# Supplementary material for: Organocatalytic desymmetrization provides access to planar chiral [2.2]paracyclophanes
Source: Nat Commun. 2024 Apr 10;15:3090. doi: 10.1038/s41467-024-47407-0 (PMC11006895; doi:10.1038/s41467-024-47407-0)
Supplement: Supplementary file 1 — Supplementary Information [file 41467_2024_47407_MOESM1_ESM.pdf]

Organocatalytic Desymmetrization Provides Access to Planar Chiral [2.2]Paracyclophanes

Vojtěch Dočekal[1], Filip Koucký[2], Ivana Císařová[2] & Jan Veselý[1*]*

[1] Department of Organic Chemistry, Faculty of Science, Charles University, Hlavova 2030/8, 128 43 Prague 2, Czech Republic, e-mail: vojtech.docekal@natur.cuni.cz, jan.vesely@natur.cuni.cz
[2] Department of Inorganic Chemistry, Faculty of Science, Charles University, Hlavova 2030/8, 128 43 Prague 2, Czech Republic

Supplementary Information

General 2

Desymmetrization reaction 2

Complete reaction optimization survey 2

General procedures for organocatalytic desymmetrization 9

Characterization data of paracyclophanes 9

Follow-up transformations and related organocatalysis 23

Mechanistic studies 28

Deuterium labeling experiments 32

Parallel kinetic isotope effect 35

General procedure for deuteration of diformyl derivatives 35

Experimental procedure for parallel kinetic isotope effect experiments 36

Stereocontrol of the process 39

Desymmetrization 39

Kinetic resolution 40

Crystallographic data 42

NMR spectra 45

Chiral HPLC 92

References 129

General

Chemicals and solvents were purchased from commercial suppliers and purified using standard techniques. Thin-layer chromatography (TLC) was performed using silica gel plates Merck 60 F_{254}. The compounds were visualized by irradiation with UV light and/or by treatment with a solution of phosphomolybdic acid (AMC) or vanillin followed by heating. Column chromatography was performed using silica gel Fluka (40-63 µm) or SiliCycle-SiliaFlash P60 (particle size: 40-63 µm, pore diameter: 60 Å. ^{1}H, ^{13}C NMR, and ^{19}F spectra were recorded with Bruker AVANCE III 400 and Bruker AVANCE III 600. Chemical shifts for protons are given in δ relative to tetramethylsilane (TMS) and referenced to residual protium in the NMR solvent (chloroform-*d*: δ_H = 7.26 ppm). Chemical shifts for carbon are referenced to the carbon of the NMR solvent (chloroform-*d*: δ_C = 77.16 ppm). The coupling constants *J* are given in hertz. IR DRIFT spectra were recorded on a Nicolet AVATAR 370 FT-IR in cm^{-1}. Chiral HPLC was performed on a LC20AD Shimadzu liquid chromatograph with an SPD-M20A diode array detector with Daicel Chiralpak® IB, Daicel Chiralpak® IC, Daicel Chiralpak® IG, and Daicel Chiralpak® ODH columns. For chiral HPLC, the samples were prepared by dissolving them in *i*-PrOH. Optical rotations were measured on AU-Tomatica polarimeter, Autopol III, and specific optical rotations are given in concentrations *c* [g/100 ml]. All melting points were measured on a Büchi melting point B-545 apparatus, in an open glass capillary, and all values are uncorrected. High-resolution mass spectra were recorded on a LCQ Fleet spectrometer. For HRMS, the samples were prepared by dissolving them in methanol.

Desymmetrization reaction

Complete reaction optimization survey

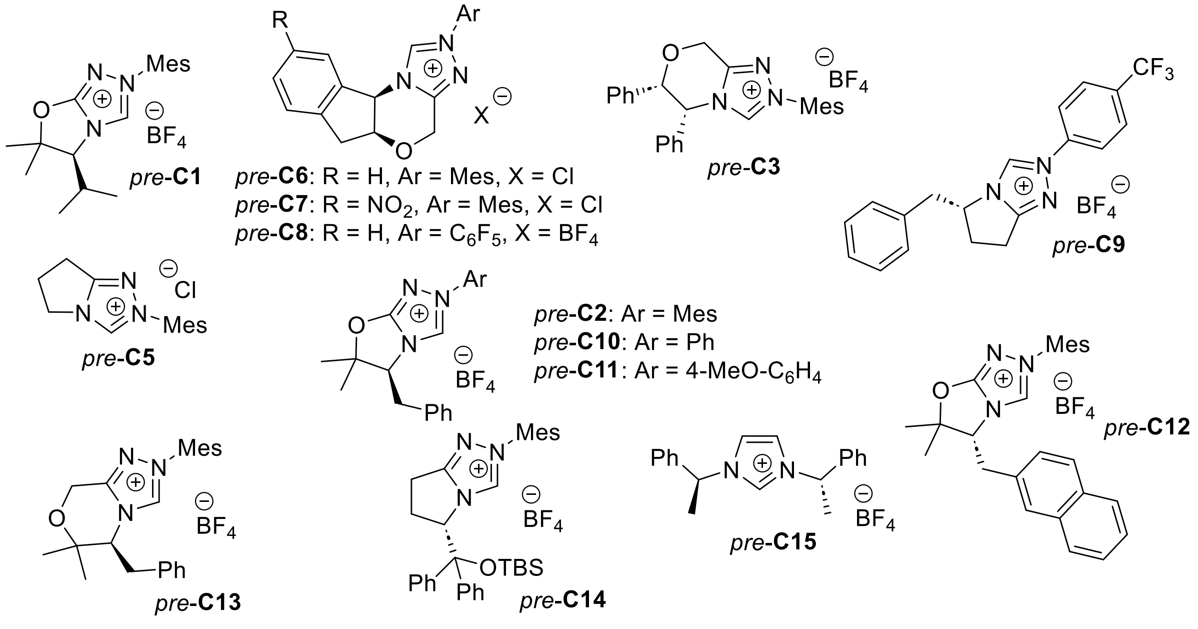

Supplementary Fig. 1. Screened precursors

Supplementary Table 1. Precursor screening

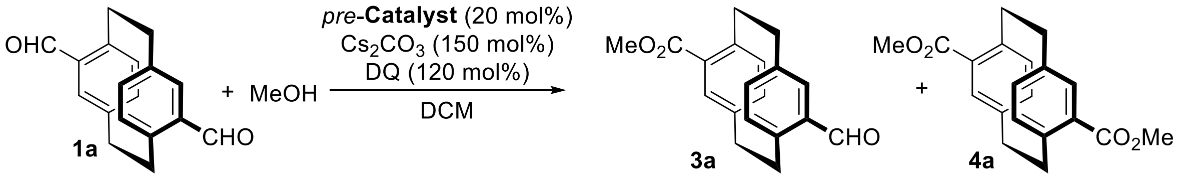

Entry[a]	*pre*-**Cat.**	Time (h)	Conversion[b]	Yield (%, 3a)[c]	Yield (%, 4a)[c]	*er* (%, 3a)[d]
1	*pre*-**C5**	1	full	15	70	50:50
2	*pre*-**C6**	72	not full	24	traces	56:44
3	*pre*-**C7**	72	not full	21	-	48:52
4	*ent-pre*-**C7**	72	not full	51	8	52:48
5	*pre*-**C8**	15	full	47	36	81:19
6	*pre*-**C3**	15	full	44	15	9:91
7	*pre*-**C9**	15	full	10	80	80:20
8	*pre*-**C1**	15	full	51	6	92:8
9	*pre*-**C2**	15	full	82	10	93:7
10	*pre*-**C10**	72	not full	18	-	38:62
11	*pre*-**C11**	72	not full	15	-	55:45
12	*pre*-**C12**	72	not full	46	17	48:52
13	*pre*-**C13**	72	not full	44	3	67:33
14	*pre*-**C14**	72	no	-	-	-
15	*pre*-**C15**	72	not full	7	-	45:55

[a] Reactions were conducted with **1a** (0.10 mmol), methanol (0.5 mmol), Cs_2CO_3 (0.15 mmol), DQ (0.12 mmol) and *pre*-**Catalyst** (20 mol%) in DCM (1.0 ml) at room temperature. [b] Determined by TLC of crude mixture. [c] Isolated yield after column chromatography. [d] Determined by chiral HPLC analysis. *Er* - enantiomeric ratio.

Supplementary Table 2. Base screening

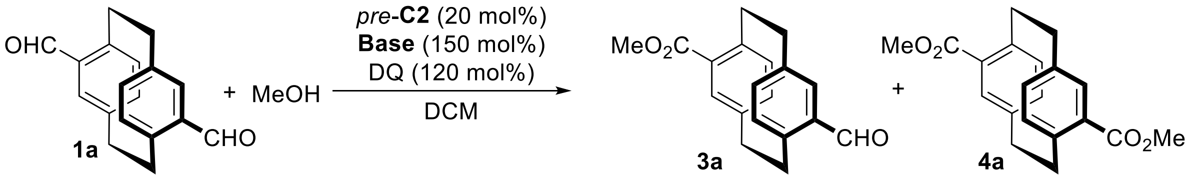

Entry[a]	Base	Time (h)	Conversion[b]	Yield (%, 3a)[c]	Yield (%, 4a)[c]	*er* (%, 3a)[d]
1	Cs_2CO_3	15	full	82	10	93:7
2	K_2CO_3	15	full	73	23	79:21
3	Na_2CO_3	15	full	70	20	79:21
4	KO*t*Bu	72	no	-	-	-
5	AcONa	72	not full	62	22	47:53
6	PhCOONa	72	not full	40	11	49:51
7	TEA	72	not full	65	25	84:16
8	DIPEA	48	full	63	22	77:23
9	DABCO	72	not full	54	21	64:36
10	DBU	72	no	-	-	-
11	pyridine	72	no	-	-	-
12	2,6-lutidine	72	no	-	-	-

[a] Reactions were conducted with **1a** (0.10 mmol), methanol (0.5 mmol), selected base (0.15 mmol), DQ (0.12 mmol) and *pre*-**C2** (20 mol%) in DCM (1.0 ml) at room temperature. [b] Determined by TLC of crude mixture. [c] Isolated yield after column chromatography. [d] Determined by chiral HPLC analysis. *Er* - enantiomeric ratio.

Supplementary Table 3. Solvent screening

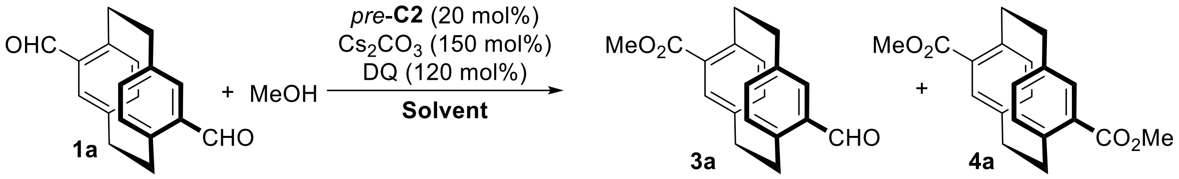

Entry[a]	Solvent	Time (h)	Conversion[b]	Yield (%, 3a)[c]	Yield (%, 4a)[c]	*er* (%, 3a)[d]
1	DCM	15	full	82	10	93:7
2	$CHCl_3$	15	full	41	37	85:15
3	1,2-DCE	72	not full	34	23	87:13
4	CCl_4	72	not full	traces	-	-
5	benzene	72	not full	traces	-	-
6	toluene	72	not full	traces	-	-
7	MeCN	72	no	-	-	-
8	EtOAc	15	full	37	34	82:18
9	MTBE	72	not ful	traces	-	-
10	THF	72	not full	41	15	52:48
11	DMSO	72	not full	traces	-	-
12	heptane	72	no	-	-	-

[a] Reactions were conducted with **1a** (0.10 mmol), methanol (0.5 mmol), Cs_2CO_3 (0.15 mmol), DQ (0.12 mmol), and *pre*-**C2** (20 mol%) in selected solvent (1.0 ml) at room temperature. [b] Determined by TLC of crude mixture. [c] Isolated yield after column chromatography. [d] Determined by chiral HPLC analysis. *Er* - enantiomeric ratio.

Supplementary Table 4. Oxidant screening

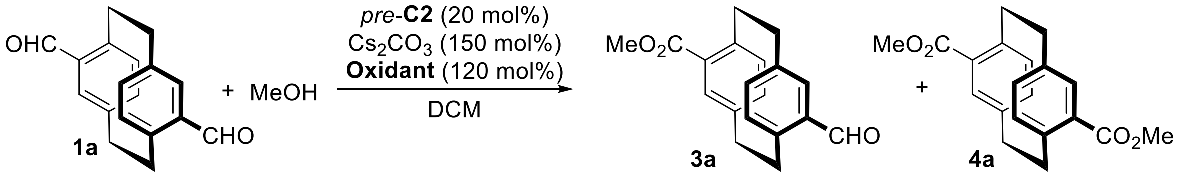

Entry[a]	Oxidant	Time (h)	Conversion[b]	Yield (%, 3a)[c]	Yield (%, 4a)[c]	*er* (%, 3a)[d]
1	DQ	15	full	82	10	93:7
2	DDQ	72	not full	traces	-	-
3	TEMPO	72	not full	21	-	88:12
4	acridine	72	no	-	-	-
5	nitrobenzene	72	not full	traces	-	-
6	MnO_2	72	not full	traces	-	-
7	electrochemical[e]	30	not full	47	-	83:17

[a] Reactions were conducted with **1a** (0.10 mmol), methanol (0.5 mmol), Cs_2CO_3 (0.15 mmol),selected oxidant (0.12 mmol), and *pre*-**C2** (20 mol%) in DCM (1.0 ml) at room temperature. [b] Determined by TLC of crude mixture. [c] Isolated yield after column chromatography. [d] Determined by chiral HPLC analysis. [e] Electrochemical oxidation (Pt cathode and anode, constant current: 1 mA, total charge: 5.44 F/mol) using TBAI (0.2 mmol) in IKA ElectraSyn 2.0 was applied. *Er* - enantiomeric ratio.

Supplementary Table 5. Precursor, base and oxidant loading

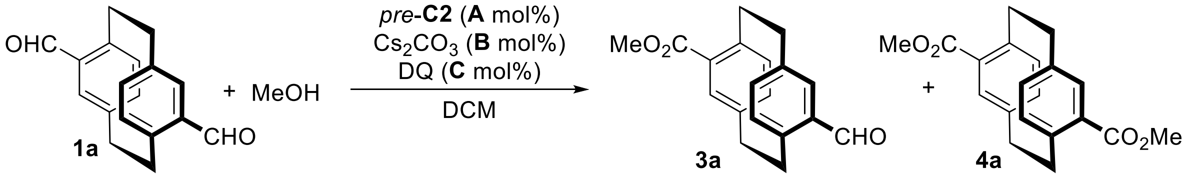

Entry[a]	A	B	C	Time (h)	Conversion[b]	Yield (%, 3a)[c]	Yield (%, 4a)[c]	*er* (%, 3a)[d]
1	20	150	120	15	full	82	10	93:7
2	10	150	120	42	full	74	24	93:7
3	5	150	120	72	not full	10	-	85:15
4	1	150	120	72	not full	traces	-	-
5	20	300	120	15	full	51	24	93:7
6	20	200	120	15	full	72	22	96:4
7	20	100	120	15	full	51	27	80:20
8	20	50	120	15	full	44	38	61:39
9	20	20	120	15	full	48	32	55:45
10	20	200	120	72	not full	65	24	91:9
11	20	200	100	72	not full	58	15	92:8

[a] Reactions were conducted with **1a** (0.10 mmol), methanol (0.5 mmol), Cs_2CO_3 (**B** mol%), DQ (**C** mol%), and *pre*-**C2** (**A** mol%) in DCM (1.0 ml) at room temperature. [b] Determined by TLC of crude mixture. [c] Isolated yield after column chromatography. [d] Determined by chiral HPLC analysis. *Er* - enantiomeric ratio.

Supplementary Table 6. Additive screening

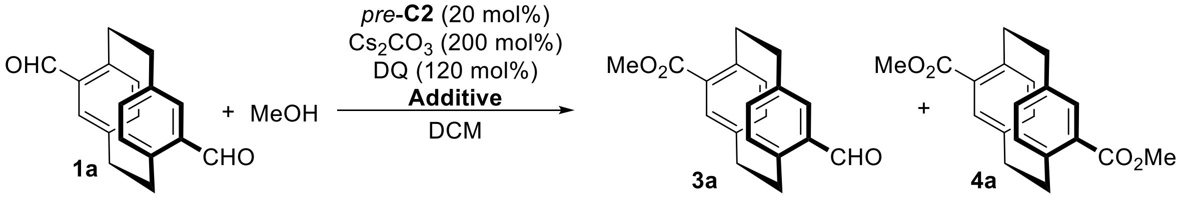

Entry[a]	Additive	Time (h)	Conversion[b]	Yield (%, 3a)[c]	Yield (%, 4a)[c]	*er* (%, 3a)[d]
1	none	15	full	72	22	96:4
2	H_2O (50 mol%)	15	full	58	6	97:3
3	H_2O (100 mol%)	24	full	56	9	94:6
4	H_2O (200 mol%)	40	full	10	-	87:13
5	MS (50mg, 3Å)	15	full	51	28	96:4

[a] Reactions were conducted with **1a** (0.10 mmol), methanol (0.5 mmol), Cs_2CO_3 (0.2 mmol), DQ (0.12 mmol), and *pre*-**C2** (20 mol%) with selected additive in DCM (1.0 ml) at room temperature. [b] Determined by TLC of crude mixture. [c] Isolated yield after column chromatography. [d] Determined by chiral HPLC analysis. *Er* - enantiomeric ratio.

Supplementary Table 7. Concentration screening

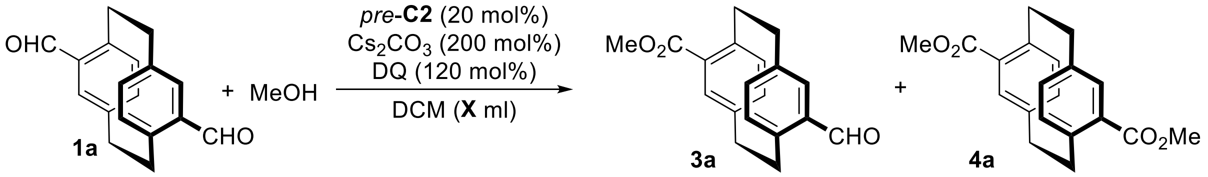

Entry[a]	X	Time (h)	Conversion[b]	Yield (%, 3a)[c]	Yield (%, 4a)[c]	*er* (%, 3a)[d]
1	1.0	15	full	72	22	96:4
2	0.5	72	not full	48	-	61:39
3	2.0	15	full	35	5	95:5
4	4.0	15	full	34	15	76:24

[a] Reactions were conducted with **1a** (0.10 mmol), methanol (0.5 mmol), Cs_2CO_3 (0.2 mmol), DQ (0.12 mmol), and *pre*-**C2** (20 mol%) in DCM (**X** ml) at room temperature. [b] Determined by TLC of crude mixture. [c] Isolated yield after column chromatography. [d] Determined by chiral HPLC analysis. *Er* - enantiomeric ratio.

Supplementary Table 8. Alcohol loading and temperature screening

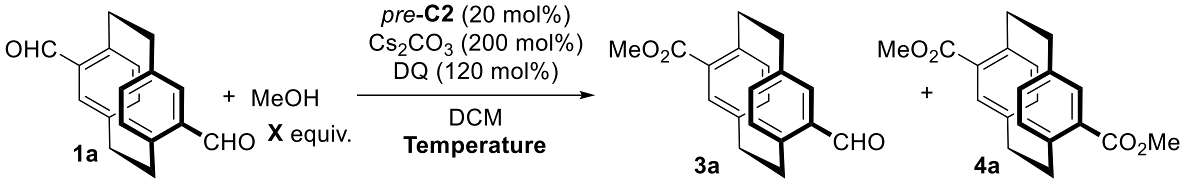

Entry[a]	X	Temp. (°C)	Time (h)	Conversion[b]	Yield (%, 3a)[c]	Yield (%, 4a)[c]	*er* (%, 3a)[d]
1	5.0	25	15	full	72	22	96:4
2	5.0	0	72	not full	13	traces	60:40
3	5.0	40	2	almost full	54	28	94:6
4	2.5	25	15	full	48	25	89:11
5	10	25	72	not full	traces	-	-
6[e]	5.0	25	15	full	87	6	99:1
7[e]	5.0	0	72	no	-	-	-

[a] Reactions were conducted with **1a** (0.10 mmol), methanol (**X** equiv.), Cs_2CO_3 (0.2 mmol), DQ (0.12 mmol), and *pre*-**C2** (20 mol%) in DCM (1.0 ml) at selected temperature. [b] Determined by TLC of crude mixture. [c] Isolated yield after column chromatography. [d] Determined by chiral HPLC analysis. [e] *Pre*-**C1** was used. *Er* - enantiomeric ratio.

Supplementary Table 9. Additional precursor screening

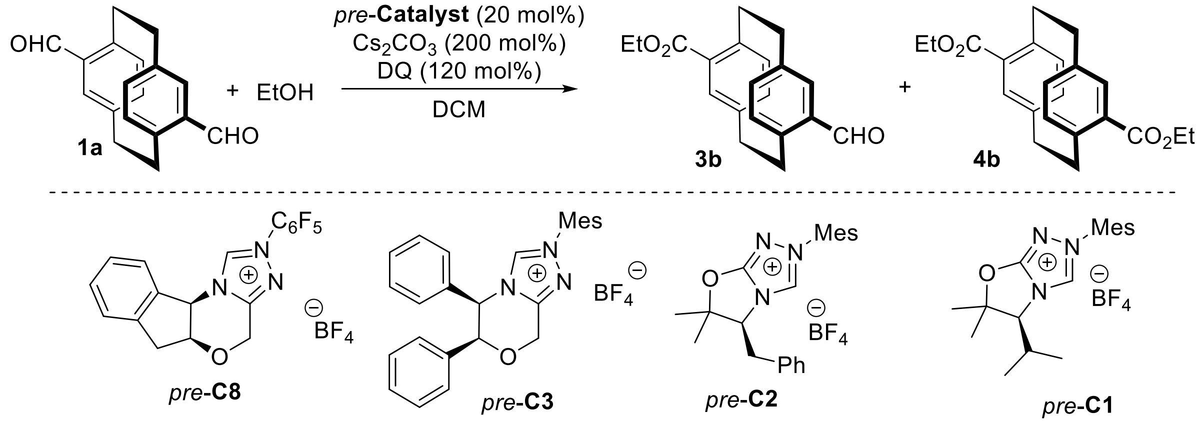

Entry[a]	*pre*-C	Time (h)	Conversion[b]	Yield (%, 3b)[c]	Yield (%, 4b)[c]	*er* (%, 3b)[d]
1	*pre*-**C8**	15	full	30	34	21:71
2	*pre*-**C3**	72	not full	30	traces	14:86
3	*pre*-**C2**	15	full	55	11	64:36
4	*pre*-**C1**	15	full	87	6	99:1

[a] Reactions were conducted with **1a** (0.10 mmol), ethanol (0.5 mmol), Cs_2CO_3 (0.2 mmol), DQ (0.12 mmol), and selected *pre*-**Catalst** (20 mol%) in DCM (1.0 ml) at room temperature. [b] Determined by TLC of crude mixture. [c] Isolated yield after column chromatography. [d] Determined by chiral HPLC analysis. *Er* - enantiomeric ratio.

Supplementary Table 10. Screening of the reaction conditions for *pseudo-gem* derivative

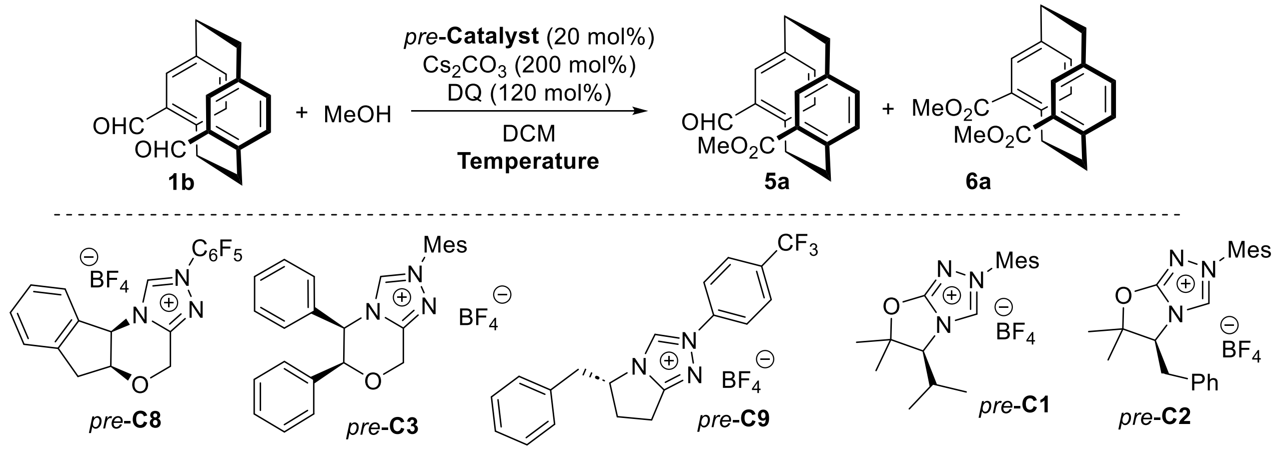

Entry[a]	*pre*-C	Temp. (°C)	Time (h)	Conversion[b]	Yield (%, 5a)[c]	Yield (%, 6a)[c]	*er* (%, 5a)[d]
1	*pre*-**C8**	r.t.	15	full	24	traces	74:26
2	*pre*-**C3**	r.t.	15	full	92	not formed	29:71
3	*pre*-**C9**	r.t.	72	not full	52	traces	50:50
4	*pre*-**C1**	0	15	full	91	not formed	99.5:0.5
5	*pre*-**C1**	20	15	full	82	traces	98:2
6	*pre*-**C1**	30	15	full	76	not formed	97:3
7	*pre*-**C1**	40	2	full	75	traces	96:4
8	*pre*-**C2**	r.t.	2	full	82	traces	96:4

[a] Reactions were conducted with **1b** (0.10 mmol), methanol (0.5 mmol), Cs_2CO_3 (0.2 mmol), DQ (0.12 mmol), and selected *pre*-**C** (20 mol%) in DCM (1.0 ml) at selected temperature. [b] Determined by TLC of crude mixture. [c] Isolated yield after column chromatography. [d] Determined by chiral HPLC analysis. *Er* - enantiomeric ratio.

General procedures for organocatalytic desymmetrization

a) using paracyclophane **1a** *as a starting material*

The vial (4 ml) was charged with **1a**[1] (26.4 mg, 0.1 mmol), *pre*-**C1**[2] (7.8 mg, 0.02 mmol), DQ (49.0 mg, 0.12 mmol), and Cs_2CO_3 (65.2 mg, 0.2 mmol), followed by DCM (1.0 ml), and the corresponding alcohol (0.5 mmol) at room temperature. The reaction was stirred for the indicated time at room temperature. Once the reaction was completed by thin-layer chromatography (TLC), the solvent was evaporated. The crude product was purified by column chromatography (eluting with hexane/EtOAc mixtures).

Note: Racemic samples were prepared in reactions with *pre*-**C5**.

b) using paracyclophane **1b** *as a starting material*

The vial (4 ml) was charged with **1b**[1] (26.4 mg, 0.1 mmol), *pre*-**C1** (7.8 mg, 0.02 mmol), DQ (49.0 mg, 0.12 mmol), and Cs_2CO_3 (65.2 mg, 0.2 mmol), followed by precooled DCM (1.0 ml, 0 °C, cryocooler), and the corresponding alcohol (0.5 mmol). The reaction was stirred for the indicated time (TLC control) at 0 °C (cryocooler). Once the reaction was completed, the solvent was evaporated. The crude product was purified by column chromatography (eluting with hexane/EtOAc mixtures).

Note: Racemic samples were prepared in reactions with *pre*-**C5**.

c) using electrochemical oxidation

The ElectraSyn vial (5 ml) was charged with **1a** (52.9 mg, 0.2 mmol), *pre*-**C2** (17.4 mg, 0.04 mmol), TBAI (110.8 mg, 0.3 mmol), and Cs_2CO_3 (97.8 mg, 0.3 mmol), followed by DCM (2.0 ml) and methanol (40 µl, 1.0 mmol). The ElectraSyn vial was equipped with anode (Pt) and cathode (Pt) and connected to Electrasyn 2.0. The mixture was electrolyzed under stirring at constant current of 1.0 mA for a total reaction time of 30 hours (total charge: 5.44 F/mol) at room temperature. After this time, traces of **1a** were still observed. Electrodes and vial were rinsed with DCM, and the solvent was evaporated. The crude product was purified by column chromatography (hexane/EtOAc - 9:1).

Characterization data of paracyclophanes

(R_p)-Methyl 4^3-Formyl-1,4(1,4)-dibenzenacyclohexaphane-1^2-yl acetate (3a)

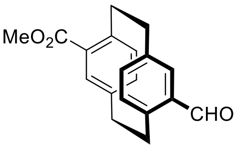

The title compound was synthesized according to the general procedure (reaction time: 15 hours), using paracyclophane **1a** (26.4 mg, 0.1 mmol) and methanol (20 µl, 0.5 mmol). The product was purified by column chromatography (hexane/EtOAc - 9:1), affording **3a** (26 mg, 87%) as a white crystalline solid. Crystals suitable for X-ray analysis were grown by dissolving **3a** in a minimal amount of boiling *i*-PrOH, followed by standing at room temperature overnight.

m.p. = 135.5-136.0 °C (*i*-PrOH). *Er* = 99:1 (*ee* = 98%), the enantiomeric excess of product **3a** was determined by high-performance liquid chromatography (HPLC) using a Chiralpak® IB column (*n*-heptane/*i*-PrOH - 80:20, flow rate = 1.0 ml/min, λ = 190 nm, t = 25 °C): t_R = 7.8 min (major), t_R = 10.1 min (minor). $[\alpha]_D^{20}$ = +8.7 (c = 0.8, $CHCl_3$). ^1H NMR (400 MHz, chloroform-*d*): δ 9.94 (s, 1H), 7.14 (d, J = 2.0 Hz, 1H), 7.04 (d, J = 2.0 Hz, 1H), 6.74 (dd, J = 7.8, 2.0 Hz, 1H), 6.58 – 6.52 (m, 2H), 6.47 (d, J = 7.8 Hz, 1H), 4.16 – 4.03 (m, 2H), 3.91 (s, 3H), 3.27 – 3.16 (m, 3H), 3.11 (ddd, J = 13.2, 10.2, 5.7 Hz, 1H), 3.05 – 2.88 (m, 2H) ppm. $^{13}C\{^1H\}$ NMR (101 MHz, chloroform-*d*): δ 192.2, 167.6, 143.0, 142.5, 141.1, 139.9, 136.9, 136.6, 136.5, 135.6, 135.4, 135.30, 135.28, 131.2, 52.1, 35.3, 34.6, 34.4, 32.8 ppm. IR (ATR): ν = 1709, 1684 (C=O, aldehyde, ester) cm^{-1}. HRMS (ESI+) *m/z*: calcd. for $C_{19}H_{19}O_3$ $[M + H]^+$: 295.1329, found: 295.1324.

(S_p)-Methyl 4^3-Formyl-1,4(1,4)-dibenzenacyclohexaphane-1^2-yl acetate (*ent*-3a)

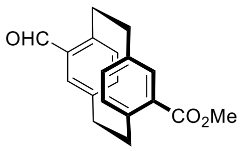

The title compound was synthesized according to the general procedure (reaction time: 15 hours), using paracyclophane **1a** (26.4 mg, 0.1 mmol) and methanol (20 µl, 0.5 mmol) and *ent-pre*-**C1** as a catalyst. The product was purified by column chromatography (hexane/EtOAc - 9:1), affording *ent*-**3a** (26 mg, 87%) as a white amorphous solid

Er = 93:7 (*ee* = 85%), the enantiomeric excess of product **3a** was determined by HPLC using a Chiralpak® IB column (*n*-heptane/*i*-PrOH - 80:20, flow rate = 1.0 ml/min, λ = 190 nm, t = 25 °C): t_R = 7.8 min (minor), t_R = 10.1 min (major). $[\alpha]_D^{20}$ = -7.9 (c = 0.6, $CHCl_3$). Other analytical data agree with the data on the opposite enantiomer (**3a**).

Dimethyl 1,4(1,4)-dibenzenacyclohexaphane-1^2,4^3-dicarboxylate (4a)

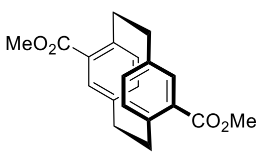

The title compound was synthesized according to the general procedure under the conditions outlined in Supplementary Table 1, Entry 1. The product was purified by column chromatography (hexane/EtOAc - 9:1), affording **4a** (23 mg, 70%) as a white amorphous solid

^{1}H NMR (400 MHz, chloroform-*d*): δ 7.14 (d, J = 2.0 Hz, 2H), 6.67 (dd, J = 7.8, 2.0 Hz, 2H), 6.49 (d, J = 7.8 Hz, 2H), 4.06 (ddd, J = 12.7, 7.1, 5.6 Hz, 2H), 3.91 (s, 6H), 3.22 – 3.11 (m, 4H), 2.91 (ddd, J = 12.9, 9.1, 7.6 Hz, 2H) ppm. $^{13}C\{^1H\}$ NMR (101 MHz, chloroform-*d*): δ 167.7 (2C), 142.4 (2C), 140.3 (2C), 135.4 (2C), 135.2 (2C), 135.0 (2C), 131.1 (2C), 52.0 (2C), 35.2 (2C), 34.5 (2C) ppm. HRMS (ESI+) *m*/*z*: calcd. for $C_{20}H_{21}O_4$ [M + H]$^+$: 325.1434, found: 325.1428.

(R_p)-Ethyl 4^3-formyl-1,4(1,4)-dibenzenacyclohexaphane-1^2-carboxylate (3b)

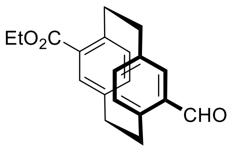

The title compound was synthesized according to the general procedure (reaction time: 15 hours), using paracyclophane **1a** (26.4 mg, 0.1 mmol) and ethanol (29 µl, 0.5 mmol). The product was purified by column chromatography (hexane/EtOAc - 10:1), affording **3b** (21 mg, 69%) as a white amorphous solid

Er = 99:1 (*ee* = 98%), the enantiomeric excess of product **3b** was determined by HPLC using a Chiralpak® IB column (*n*-heptane/*i*-PrOH - 80:20, flow rate = 1.0 ml/min, λ = 190 nm, t = 25 °C): t_R = 6.8 min (major), t_R = 8.8 min (minor). $[\alpha]_D^{20}$ = -3.8 (c = 0.7, $CHCl_3$). ^{1}H NMR (400 MHz, chloroform-*d*): δ 9.94 (s, 1H), 7.14 (d, J = 2.0 Hz, 1H), 7.04 (d, J = 2.0 Hz, 1H), 6.76 (dd, J = 7.8, 2.0 Hz, 1H), 6.55 (td, J = 4.9, 2.5 Hz, 2H), 6.47 (d, J = 7.9 Hz, 1H), 4.39 (q, J = 7.1 Hz, 2H), 4.16 – 4.05 (m, 2H), 3.29 – 3.17 (m, 3H), 3.11 (ddd, J = 13.2, 10.2, 5.7 Hz, 1H), 2.96 (dddd, J = 29.3, 13.0, 10.0, 6.4 Hz, 2H), 1.44 (t, J = 7.1 Hz, 3H) ppm. $^{13}C\{^1H\}$ NMR (101 MHz, chloroform-*d*): δ 192.2, 167.2, 143.1, 142.3, 141.1, 139.9, 136.9, 136.60, 136.55, 135.5, 135.44, 135.37, 135.3, 131.6, 60.9, 35.4, 34.6, 34.4, 32.9, 14.6 ppm. IR (ATR): ν = 1705, 1684 (C=O, aldehyde, ester) cm^{-1}. HRMS (ESI+) *m*/*z*: calcd. for $C_{20}H_{21}O_3$ [M + H]$^+$: 309.1485, found: 309.1484.

Diethyl 1,4(1,4)-dibenzenacyclohexaphane-1^2,4^3-dicarboxylate (4b)

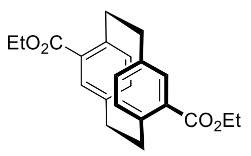

The title compound was synthesized according to the general procedure under the conditions outlined in Supplementary Table 9, Entry 1. The product was purified by column chromatography (hexane/EtOAc - 10:1), affording **4b** (12 mg, 34%) as a white amorphous solid.

^{1}H NMR (400 MHz, chloroform-*d*): δ 7.14 (d, J = 2.0 Hz, 2H), 6.68 (dd, J = 7.8, 2.0 Hz, 2H), 6.49 (d, J = 7.8 Hz, 2H), 4.39 (q, J = 7.1 Hz, 4H), 4.07 (ddd, J = 12.6, 8.1,

4.4 Hz, 2H), 3.22 – 3.05 (m, 4H), 2.90 (ddd, J = 12.9, 9.4, 7.0 Hz, 2H), 1.44 (t, J = 7.1 Hz, 6H) ppm. $^{13}C\{^1H\}$ NMR (101 MHz, chloroform-*d*): δ 167.3 (2C), 142.3 (2C), 140.2 (2C), 135.3 (2C), 135.2 (2C), 134.9 (2C), 131.4 (2C), 60.8 (2C), 35.3 (2C), 34.5 (2C), 14.6 (2C) ppm. IR (ATR): ν = 1695 (C=O, ester) cm^{-1}. HRMS (ESI+) *m*/*z*: calcd. for $C_{22}H_{25}O_4$ [M + H]$^+$, 353.1747; found, 353.1744.

(R_p)-Isopropyl 4^3-formyl-1,4(1,4)-dibenzenacyclohexaphane-1^2-carboxylate (3c)

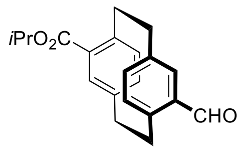

The title compound was synthesized according to the general procedure (reaction time: 72 hours, without full conversion **1a**), using paracyclophane **1a** (26.4 mg, 0.1 mmol) and isopropanol (38 µl, 0.5 mmol). The crude product was purified by column chromatography (hexane/EtOAc - 10:1), affording **3c** (14 mg, 43%) as a white amorphous solid.

Er = 94:6 (*ee* = 88%), the enantiomeric excess of product **3c** was determined by HPLC using a Chiralpak® IB column (*n*-heptane/*i*-PrOH - 80:20, flow rate = 1.0 ml/min, λ = 190 nm, t = 25 °C): t_R = 5.9 min (major), t_R = 7.6 min (minor). $[\alpha]_D^{20}$ ~ 0 (c = 0.3, $CHCl_3$). ^1H NMR (400 MHz, chloroform-*d*): δ 9.94 (s, 1H), 7.13 (d, J = 2.0 Hz, 1H), 7.04 (d, J = 2.0 Hz, 1H), 6.77 (dd, J = 7.8, 2.0 Hz, 1H), 6.59 – 6.52 (m, 2H), 6.46 (d, J = 7.8 Hz, 1H), 5.27 (p, J = 6.3 Hz, 1H), 4.18 – 4.05 (m, 2H), 3.31 – 3.05 (m, 4H), 2.96 (dddd, J = 33.2, 13.0, 10.2, 6.2 Hz, 2H), 1.43 (dd, J = 10.9, 6.3 Hz, 6H) ppm. $^{13}C\{^1H\}$ NMR (101 MHz, chloroform-*d*): δ 192.2, 166.7, 143.1, 142.2, 141.0, 139.8, 136.9, 136.57, 136.55, 135.47, 135.40, 135.33, 135.25, 132.0, 68.2, 35.5, 34.6, 34.4, 32.9, 22.3, 22.1 ppm. IR (ATR): ν = 1695, 1666 (C=O, aldehyde, ester) cm^{-1}. HRMS (ESI+) *m*/*z*: calcd. for $C_{21}H_{22}NaO_3$ [M + Na]$^+$: 345.1461, found: 345.1457.

(R_p)-4^3-(1-(Oxo-λ^3-methoxy)-1λ^5-dodecyl)-1,4(1,4)-dibenzenacyclohexaphane-1^2-carbaldehyde (3d)

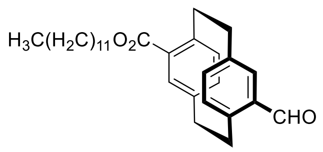

The title compound was synthesized according to the general procedure (reaction time: 15 hours), using paracyclophane **1a** (26.4 mg, 0.1 mmol) and lauryl alcohol (93 mg, 0.5 mmol). The product was purified by column chromatography (hexane/EtOAc - 15:1), affording **3d** (39 mg, 87%) as a white amorphous solid.

Er = 94:6 (*ee* = 89%), the enantiomeric excess of product **3d** was determined by HPLC using a Chiralpak® IB column (*n*-heptane/*i*-PrOH - 80:20, flow rate = 1.0 ml/min, λ = 190 nm, t = 25 °C): t_R = 5.3 min (major), t_R = 6.3 min (minor). $[\alpha]_D^{20}$ = +3.1 (c = 0.8, $CHCl_3$). ^1H NMR (400 MHz, chloroform-*d*): δ 9.94 (s, 1H), 7.14 (d, J = 2.0 Hz, 1H), 7.04 (d, J = 2.0 Hz, 1H), 6.75 (dd, J = 7.8, 2.0 Hz, 1H), 6.57 – 6.51 (m, 2H), 6.47 (d, J = 7.8 Hz, 1H), 4.32 (qt, J = 10.8, 6.7 Hz, 2H), 4.10 (tdd, J = 12.7, 5.1, 3.0 Hz, 2H), 3.30 – 3.05 (m, 4H), 2.96 (dddd, J = 27.1, 12.9, 9.9, 6.5 Hz, 2H), 1.86 – 1.76 (m, 2H), 1.54 – 1.43 (m, 2H), 1.43 – 1.19 (m, 17H), 0.90 – 0.85 (m, 3H). ppm. $^{13}C\{^1H\}$ NMR (101 MHz, chloroform-*d*): δ 192.2, 167.3, 143.0, 142.4, 141.1, 139.8, 136.9, 136.60, 136.56, 135.45, 135.42, 135.37, 135.27, 131.6, 65.1, 35.4, 34.6, 34.4, 32.9, 32.1, 29.79, 29.78, 29.74, 29.72, 29.48, 29.45, 29.0, 26.3, 22.8, 14.3 ppm. IR (ATR): ν = 1707, 1689 (C=O, aldehyde, ester) cm^{-1}. HRMS (ESI+) *m*/*z*: calcd. for $C_{30}H_{40}NaO_3$ [M + Na]$^+$: 471.2870, found: 471.2861.

(R_p)-2-Methoxyethyl 4^3-formyl-1,4(1,4)-dibenzenacyclohexaphane-1^2-carboxylate (3e)

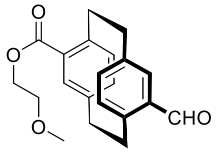

The title compound was synthesized according to the general procedure (reaction time: 15 hours), using paracyclophane **1a** (26.4 mg, 0.1 mmol) and 2-methoxyethan-1-ol (39 µl, 0.5 mmol). The product was purified by column chromatography (hexane/EtOAc - 9:1 to 6:1), affording **3e** (24 mg, 70%) as a colorless oil.

Er = 98:2 (*ee* = 96%), the enantiomeric excess of product **3e** was determined by HPLC using a Chiralpak® IB column (*n*-heptane/*i*-PrOH - 80:20, flow rate = 1.0 ml/min, λ = 190 nm, t = 25 °C): t_R = 9.7 min (major), t_R = 11.1 min (minor). $[\alpha]_D^{20}$ = -1.7 (c = 0.6, $CHCl_3$). ^{1}H NMR (400 MHz, chloroform-*d*): δ 9.94 (s, 1H), 7.17 (d, J = 2.0 Hz, 1H), 7.04 (d, J = 2.0 Hz, 1H), 6.78 (dd, J = 7.7, 2.0 Hz, 1H), 6.56 (td, J = 5.1, 2.5 Hz, 2H), 6.47 (d, J = 7.8 Hz, 1H), 4.49 (ddd, J = 4.8, 3.5, 0.7 Hz, 2H), 4.17 – 4.02 (m, 2H), 3.81 – 3.72 (m, 2H), 3.46 (s, 3H), 3.30 – 3.17 (m, 3H), 3.10 (ddd, J = 13.2, 10.3, 5.7 Hz, 1H), 3.05 – 2.86 (m, 2H) ppm. $^{13}C\{^1H\}$ NMR (101 MHz, chloroform-*d*): δ 192.2, 167.1, 143.0, 142.5, 141.1, 139.9, 136.9, 136.7, 136.5, 135.60, 135.57, 135.5, 135.3, 131.3, 70.8, 63.7, 59.1, 35.5, 34.6, 34.3, 32.8 ppm. IR (ATR): ν = 1709, 1680 (C=O, aldehyde, ester), 1072 (C-O, ether) cm^{-1}. HRMS (ESI+) *m*/*z*: calcd. for $C_{21}H_{22}NaO_4$ $[M + Na]^+$: 361.1410, found: 361.1406.

(*R_p*)-2-Bromoethyl 4³-formyl-1,4(1,4)-dibenzenacyclohexaphane-1²-carboxylate (3f)

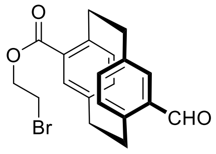

The title compound was synthesized according to the general procedure (reaction time: 15 hours), using paracyclophane **1a** (26.4 mg, 0.1 mmol) and 2-bromoethan-1-ol (36 µl, 0.5 mmol). The product was purified by column chromatography (hexane/EtOAc – 8:1), affording **3f** (29 mg, 76%) as a white amorphous solid.

Er = 82:18 (*ee* = 65%), the enantiomeric excess of product **3f** was determined by HPLC using a Chiralpak® IB column (*n*-heptane/*i*-PrOH - 80:20, flow rate = 1.0 ml/min, λ = 190 nm, t = 25 °C): t_R = 10.2 min (major), t_R = 12.6 min (minor). $[\alpha]_D^{20}$ = +2.2 (c = 0.7, $CHCl_3$). ^{1}H NMR (400 MHz, chloroform-*d*): δ 9.94 (s, 1H), 7.22 (d, J = 2.0 Hz, 1H), 7.05 (d, J = 2.1 Hz, 1H), 6.79 (dd, J = 7.7, 2.0 Hz, 1H), 6.59 (dd, J = 7.9, 2.0 Hz, 2H), 6.49 (d, J = 7.8 Hz, 1H), 4.72 (ddd, J = 11.7, 6.3, 5.3 Hz, 1H), 4.59 (ddd, J = 12.2, 6.0, 5.3 Hz, 1H), 4.19 – 4.05 (m, 2H), 3.76 – 3.67 (m, 2H), 3.28 – 3.18 (m, 3H), 3.12 (ddd, J = 13.2, 10.2, 5.8 Hz, 1H), 2.97 (dddd, J = 25.3, 13.0, 9.8, 6.8 Hz, 2H) ppm. $^{13}C\{^1H\}$ NMR (101 MHz, chloroform-*d*): δ 192.2, 166.5, 143.1, 142.9, 141.0, 140.0, 136.9, 136.7, 136.6, 135.9, 135.7, 135.5, 135.4, 130.7, 64.3, 35.4, 34.6, 34.4, 32.9, 29.5 ppm. IR (ATR): ν = 1714, 1687 (C=O, aldehyde, ester), 642 (C-Br) cm^{-1}. HRMS (ESI+) *m*/*z*: calcd. for $C_{21}H_{22}NO_2$ $[M + H]^+$: 320.1645, found: 320.1649.

(*R_p*)-(*Z*)-Hex-3-en-1-yl 4³-formyl-1,4(1,4)-dibenzenacyclohexaphane-1²-carboxylate (3g)

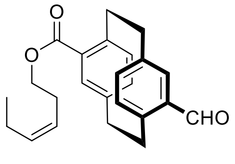

The title compound was synthesized according to the general procedure (reaction time: 15 hours), using paracyclophane **1a** (26.4 mg, 0.1 mmol) and (*Z*)-hex-3-en-1-ol (59 µl, 0.5 mmol). The product was purified by column chromatography (hexane/EtOAc –10:1), affording **3g** (26 mg, 73%) as a light-yellow oil.

Er = 96:4 (*ee* = 92%), the enantiomeric excess of product **3g** was determined by HPLC using a Chiralpak® IB column (*n*-heptane/*i*-PrOH - 80:20, flow rate = 1.0 ml/min, λ = 190 nm, t = 25 °C): t_R = 6.1 min (major), t_R = 7.3 min (minor). $[\alpha]_D^{20}$ = -16.2 (c = 0.7, $CHCl_3$). ^{1}H NMR (400 MHz, chloroform-*d*): δ 9.94 (s, 1H), 7.14 (d, J = 2.0 Hz, 1H), 7.04 (d, J = 2.0 Hz, 1H), 6.75 (dd, J = 7.8, 2.0 Hz, 1H), 6.60 – 6.50 (m, 2H), 6.46 (d, J = 7.8 Hz, 1H), 5.67 – 5.53 (m, 1H), 5.53 – 5.35 (m, 1H), 4.33 (qt, J = 10.7, 6.8 Hz, 2H), 4.10 (ddt, J = 12.8, 8.5, 3.1 Hz, 2H), 3.29 – 3.06 (m, 4H), 2.95 (dddd, J = 27.5, 12.9, 9.7, 6.7 Hz, 2H), 2.56 (qdd, J = 6.9, 1.6, 0.8 Hz, 2H), 2.22 – 2.04 (m, 2H), 1.01 (t, J = 7.5 Hz, 3H) ppm. $^{13}C\{^1H\}$ NMR (101 MHz, chloroform-*d*): δ 192.2, 167.2, 143.0, 142.4, 141.1, 139.8, 136.9, 136.7, 136.6, 135.51, 135.45, 135.4, 135.3, 134.9, 131.5, 124.1, 64.5, 35.3, 34.6, 34.4, 32.9, 27.1, 20.9, 14.4 ppm. IR (ATR): ν = 1707, 1684 (C=O, aldehyde, ester) cm^{-1}. HRMS (ESI+) *m*/*z*: calcd. for $C_{24}H_{26}NaO_3$ $[M + Na]^+$: 385.1774, found: 385.1767.

(R_p)-Pent-4-en-1-yl 4³-formyl-1,4(1,4)-dibenzenacyclohexaphane-1²-carboxylate (3h)

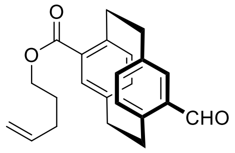

The title compound was synthesized according to the general procedure (reaction time: 15 hours), using paracyclophane **1a** (26.4 mg, 0.1 mmol) and pent-4-en-1-ol (52 µl, 0.5 mmol). The product was purified by column chromatography (hexane/EtOAc - 9:1), affording **3h** (32 mg, 93%) as a yellow oil.

Er = 88:12 (*ee* = 77%), the enantiomeric excess of product **3h** was determined by HPLC using a Chiralpak® IB column (*n*-heptane/*i*-PrOH - 80:20, flow rate = 1.0 ml/min, λ = 190 nm, t = 25 °C): t_R = 6.5 min (major), t_R = 7.7 min (minor). $[\alpha]_D^{20}$ = +2.8 (c = 0.5, $CHCl_3$). ^{1}H NMR (400 MHz, chloroform-*d*): δ 9.94 (s, 1H), 7.14 (d, J = 2.0 Hz, 1H), 7.04 (d, J = 2.0 Hz, 1H), 6.75 (dd, J = 7.7, 2.0 Hz, 1H), 6.59 – 6.51 (m, 2H), 6.47 (d, J = 7.8 Hz, 1H), 5.88 (ddt, J = 16.9, 10.2, 6.6 Hz, 1H), 5.16 – 4.98 (m, 2H), 4.45 – 4.25 (m, 2H), 4.19 – 4.00 (m, 2H), 3.28 – 3.05 (m, 4H), 2.96 (dddd, J = 26.2, 12.9, 9.9, 6.5 Hz, 2H), 2.33 – 2.18 (m, 2H), 1.91 (dq, J = 8.4, 6.8 Hz, 2H) ppm. ^{13}C{^{1}H} NMR (101 MHz, chloroform-*d*): δ 192.1, 167.1, 142.9, 142.3, 140.9, 139.7, 137.4, 136.8, 136.5, 136.4, 135.4, 135.3, 135.2, 135.2, 131.4, 115.5, 64.2, 35.2, 34.5, 34.3, 32.7, 30.3, 28.1 ppm. IR (ATR): ν = 1709, 1691 (C=O, aldehyde) cm^{-1}. HRMS (ESI+) *m*/*z*: calcd. for $C_{23}H_{24}NaO_3$ $[M + H]^+$: 371.1618, found: 371.1614.

(R_p)-Pent-3-yn-1-yl 4³-formyl-1,4(1,4)-dibenzenacyclohexaphane-1²-carboxylate (3i)

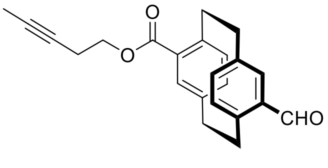

The title compound was synthesized according to the general procedure (reaction time: 15 hours), using paracyclophane **1a** (26.4 mg, 0.1 mmol) and pent-3-yn-1-ol (46 µl, 0.5 mmol). The product was purified by column chromatography (hexane/EtOAc – 8:1), affording **3i** (31 mg, 90%) as a yellow amorphous solid.

Er = 96:4 (*ee* = 91%), the enantiomeric excess of product **3i** was determined by HPLC using a Chiralpak® IB column (*n*-heptane/*i*-PrOH - 80:20, flow rate = 1.0 ml/min, λ = 190 nm, t = 25 °C): t_R = 7.4 min (major), t_R = 9.4 min (minor). $[\alpha]_D^{20}$ = -2.9 (c = 0.9, $CHCl_3$). ^{1}H NMR (400 MHz, chloroform-*d*): δ 9.94 (s, 1H), 7.19 (d, J = 2.0 Hz, 1H), 7.04 (d, J = 2.0 Hz, 1H), 6.81 (dd, J = 7.8, 2.0 Hz, 1H), 6.60 – 6.54 (m, 2H), 6.47 (d, J = 7.9 Hz, 1H), 4.47 – 4.31 (m, 2H), 4.19 – 4.06 (m, 2H), 3.29 – 3.17 (m, 3H), 3.11 (ddd, J = 13.2, 10.3, 5.8 Hz, 1H), 3.05 – 2.87 (m, 2H), 2.66 (ddt, J = 9.0, 6.6, 2.5 Hz, 2H), 1.83 (t, J = 2.5 Hz, 3H) ppm.^{13}C{^{1}H} NMR (101 MHz, chloroform-*d*): δ 192.2, 166.9, 143.0, 142.5, 141.1, 139.9, 136.9, 136.7, 136.6, 135.64, 135.61, 135.5, 135.3, 131.4, 77.5, 63.4, 35.4, 34.6, 34.3, 32.9, 19.6, 3.7 ppm. IR (KBr): ν = 1709, 1689 (C=O, aldehyde, ester) cm^{-1}. HRMS (ESI+) *m*/*z*: calcd. for $C_{23}H_{22}NaO_3$ $[M + Na]^+$: 369.1461, found: 369.1463.

(R_p)-But-3-yn-1-yl 4³-formyl-1,4(1,4)-dibenzenacyclohexaphane-1²-carboxylate (3j)

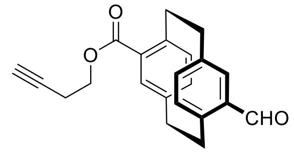

The title compound was synthesized according to the general procedure (reaction time: 15 hours), using paracyclophane **1a** (26.4 mg, 0.1 mmol) and but-3-yn-1-ol (38 µl, 0.5 mmol). The product was purified by column chromatography (hexane/EtOAc – 8:1), affording **3j** (30 mg, 91%) as a colorless oil.

Er = 92:8 (*ee* = 84%), the enantiomeric excess of product **3j** was determined by HPLC using a Chiralpak® IB column (*n*-heptane/*i*-PrOH - 80:20, flow rate = 1.0 ml/min, λ = 199 nm, t = 25 °C): t_R = 8.9 min (major), t_R = 11.5 min (minor). $[\alpha]_D^{20}$ ~ 0 (c = 0.5, $CHCl_3$). ^{1}H NMR (400 MHz, chloroform-*d*): δ 9.94 (s, 1H), 7.19 (d, J = 2.0 Hz, 1H), 7.04 (d, J = 2.0 Hz, 1H), 6.79 (dd, J = 7.8, 2.0 Hz, 1H), 6.57 (dd, J = 7.9, 1.6 Hz, 2H), 6.48 (d, J = 7.8 Hz, 1H), 4.49 (dt, J = 10.6, 6.5 Hz, 1H), 4.40 (dt, J = 10.7, 6.5 Hz, 1H), 4.17 – 4.06 (m, 2H), 3.31 – 3.17 (m, 3H), 3.11 (ddd, J = 13.2, 10.2, 5.8 Hz, 1H), 3.05 – 2.87 (m, 2H), 2.73 (td, J = 6.5, 2.7 Hz, 2H), 2.10

(t, J = 2.7 Hz, 1H) ppm. $^{13}C\{^{1}H\}$ NMR (101 MHz, chloroform-d): δ 192.2, 166.8, 143.0, 142.6, 141.0, 139.9, 136.9, 136.7, 136.6, 135.7, 135.6, 135.5, 135.3, 131.1, 80.6, 70.2, 62.7, 35.4, 34.6, 34.4, 32.9, 19.4 ppm. IR (KBr): ν = 1712, 1693 (C=O, aldehyde, ester) cm^{-1}. HRMS (ESI+) m/z: calcd. for $C_{22}H_{20}NaO_3$ [M + H]$^+$: 355.1305, found: 355.1299.

(R_p)-Benzyl 4^3-formyl-1,4(1,4)-dibenzenacyclohexaphane-1^2-carboxylate (3k)

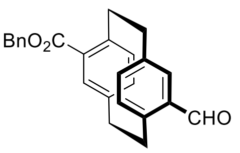

The title compound was synthesized according to the general procedure (reaction time: 48 hours), using paracyclophane **1a** (26.4 mg, 0.1 mmol) and benzyl alcohol (52 µl, 0.5 mmol). The product was purified by column chromatography (hexane/EtOAc – 8:1), affording **3k** (33 mg, 90%) as a white amorphous solid.

Er = 90:10 (ee = 80%), the enantiomeric excess of product **3k** was determined by HPLC using a Chiralpak® IB column (n-heptane/i-PrOH - 80:20, flow rate = 1.0 mL/min, λ = 190 nm, t = 25 °C): t_R = 8.5 min (major), t_R = 10.2 min (minor). $[\alpha]_D^{20}$ = +8.8 (c = 0.6, $CHCl_3$). ^{1}H NMR (400 MHz, chloroform-d): δ 9.92 (s, 1H), 7.56 – 7.48 (m, 2H), 7.47 – 7.40 (m, 2H), 7.40 – 7.35 (m, 1H), 7.19 (d, J = 2.0 Hz, 1H), 7.01 (d, J = 2.0 Hz, 1H), 6.58 (ddd, J = 12.3, 7.8, 2.1 Hz, 2H), 6.47 (dd, J = 7.8, 3.9 Hz, 2H), 5.45 – 5.30 (m, 2H), 4.09 (ddd, J = 12.7, 10.1, 2.3 Hz, 2H), 3.28 – 3.03 (m, 4H), 2.93 (dddd, J = 29.4, 12.9, 10.5, 6.0 Hz, 2H) ppm. $^{13}C\{^{1}H\}$ NMR (101 MHz, chloroform-d): δ 192.2, 166.8, 143.0, 142.7, 141.0, 139.9, 136.9, 136.6, 136.6, 136.3, 135.6, 135.5, 135.4, 135.3, 131.2, 128.8 (2C), 128.60 (2C), 128.56, 66.7, 35.4, 34.6, 34.3, 32.9 ppm. IR (ATR): ν = 1712, 1670 (C=O, aldehyde, ester) cm^{-1}. HRMS (ESI+) m/z: calcd. for $C_{25}H_{22}NaO_3$ [M + Na]$^+$: 393.1461, found: 393.1458.

(R_p)-Phenethyl 4^3-formyl-1,4(1,4)-dibenzenacyclohexaphane-1^2-carboxylate (3l)

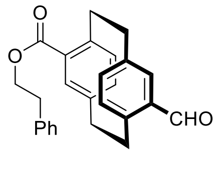

The title compound was synthesized according to the general procedure (reaction time: 15 hours), using paracyclophane **1a** (26.4 mg, 0.1 mmol) and 2-phenylethan-1-ol (60 µl, 0.5 mmol). The product was purified by column chromatography (hexane/EtOAc – 9:1), affording **3l** (31 mg, 82%) as a white amorphous solid.

Er = 92:8 (ee = 83%), the enantiomeric excess of product **3l** was determined by HPLC using a Chiralpak® IB column (n-heptane/i-PrOH - 80:20, flow rate = 1.0 ml/min, λ = 190 nm, t = 25 °C): t_R = 9.4 min (major), t_R = 11.2 min (minor). $[\alpha]_D^{20}$ = +7.5 (c = 0.4, $CHCl_3$). ^{1}H NMR (400 MHz, chloroform-d): δ 9.92 (s, 1H), 7.56 – 7.48 (m, 2H), 7.47 – 7.40 (m, 2H), 7.40 – 7.35 (m, 1H), 7.19 (d, J = 2.0 Hz, 1H), 7.01 (d, J = 2.0 Hz, 1H), 6.58 (ddd, J = 12.3, 7.8, 2.1 Hz, 2H), 6.47 (dd, J = 7.8, 3.9 Hz, 2H), 5.45 – 5.30 (m, 2H), 4.09 (ddd, J = 12.7, 10.1, 2.3 Hz, 2H), 3.28 – 3.03 (m, 4H), 2.93 (dddd, J = 29.4, 12.9, 10.5, 6.0 Hz, 2H) ppm. $^{13}C\{^{1}H\}$ NMR (101 MHz, chloroform-d): δ 192.2, 167.0, 142.9, 142.5, 141.0, 139.8, 138.1, 136.8, 136.6, 136.52, 135.53, 135.41, 135.39, 135.2, 131.3, 129.1 (2C), 128.8 (2C), 126.9, 65.2, 35.4, 35.2, 34.6, 34.3, 32.9 ppm. IR (ATR): ν = 1705, 1684 (C=O, aldehyde, ester), cm^{-1}. HRMS (ESI+) m/z: calcd. for $C_{26}H_{24}NaO_3$ [M + Na]$^+$: 407.1618, found: 407.1617.

(R_p)-2-(Ferrocen-2-yl)ethyl 4^3-formyl-1,4(1,4)-dibenzenacyclohexaphane-1^2-carboxylate (3m)

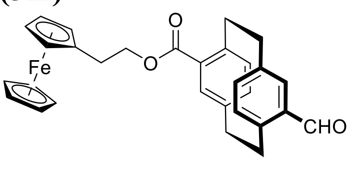

The title compound was synthesized according to the general procedure (reaction time: 15 hours), using paracyclophane **1a** (26.4 mg, 0.1 mmol) and 2-hydroxyethylferrocene[3] (115 mg, 0.5 mmol). The product was purified by column chromatography (hexane/EtOAc – 9:1), affording **3m** (39 mg, 79%) as an orange amorphous solid.

Er = 93:7 (*ee* = 86%), the enantiomeric excess of product **3m** was determined by HPLC using a Chiralpak® IB column (*n*-heptane/*i*-PrOH - 80:20, flow rate = 1.0 ml/min, λ = 194 nm, *t* = 25 °C): t_R = 13.0 min (major), t_R = 15.2 min (minor). $[\alpha]_D^{20}$ = +4.6 (*c* = 0.6, $CHCl_3$). ^{1}H NMR (400 MHz, acetonitrile-*d*): δ 9.94 (s, 1H), 7.16 (d, *J* = 2.0 Hz, 1H), 7.08 (d, *J* = 1.9 Hz, 1H), 6.71 (dd, *J* = 7.8, 2.0 Hz, 1H), 6.60 – 6.47 (m, 3H), 4.60 – 4.14 (m, 11H), 4.10 – 3.96 (m, 2H), 3.21 (tdd, *J* = 12.7, 10.6, 3.3 Hz, 2H), 3.14 – 2.88 (m, 4H), 2.74 (s, 2H). ppm.^{13}C{^{1}H} NMR (101 MHz, acetonitrile-*d*): δ 193.3, 167.7, 143.9, 143.3, 142.1, 141.0, 137.9, 137.3, 137.0, 136.4, 136.2, 136.1, 136.0, 132.5, 71.0 (10C), 65.8, 35.6, 35.0, 34.8, 33.0, 29.6 ppm. IR (ATR): ν = 1709, 1689 (C=O, aldehyde, ester) cm^{-1}. HRMS (ESI+) *m/z*: calcd. for $C_{30}H_{28}FeNaO_3$ $[M + Na]^+$: 515.1280, found: 515.1276.

(*R$_p$*)-2-(1*H*-indol-3-yl)ethyl 4^3-formyl-1,4(1,4)-dibenzenacyclohexaphane-1^2-carboxylate (3n)

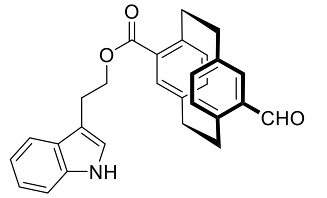

The title compound was synthesized according to the general procedure (reaction time: 48 hours), using paracyclophane **1a** (26.4 mg, 0.1 mmol) and tryptophol (81 mg, 0.5 mmol). The product was purified by column chromatography (hexane/EtOAc – 3:1), affording **3n** (36 mg, 86%) as a white amorphous solid.

Er = 91:9 (*ee* = 82%), the enantiomeric excess of product **3n** was determined by HPLC using a Chiralpak® IB column (*n*-heptane/*i*-PrOH - 60:40, flow rate = 1.0 ml/min, λ = 190 nm, *t* = 25 °C): t_R = 14.0 min (major), t_R = 18.9 min (minor). $[\alpha]_D^{20}$ = +5.3 (*c* = 0.4, $CHCl_3$). ^{1}H NMR (400 MHz, chloroform-*d*): δ 9.91 (s, 1H), 8.10 (br s, 1H), 7.75 (dt, *J* = 7.7, 1.0 Hz, 1H), 7.41 (dt, *J* = 8.1, 1.0 Hz, 1H), 7.28 – 7.17 (m, 2H), 7.16 (d, *J* = 2.4 Hz, 1H), 7.05 (d, *J* = 2.0 Hz, 1H), 6.99 (d, *J* = 2.0 Hz, 1H), 6.53 (td, *J* = 8.2, 2.0 Hz, 2H), 6.43 (d, *J* = 7.8 Hz, 1H), 6.19 (d, *J* = 7.7 Hz, 1H), 4.68 (dt, *J* = 10.7, 7.2 Hz, 1H), 4.60 (dt, *J* = 10.7, 6.6 Hz, 1H), 4.04 (dddd, *J* = 13.0, 10.3, 4.8, 2.7 Hz, 2H), 3.30 (tdd, *J* = 7.2, 3.5, 0.9 Hz, 2H), 3.21 – 2.98 (m, 4H), 2.86 (dddd, *J* = 12.3, 10.5, 6.0, 1.8 Hz, 2H) ppm. ^{13}C{^{1}H} NMR (101 MHz, chloroform-*d*): δ 192.2, 167.1, 143.0, 142.5, 141.0, 139.8, 136.8, 136.6, 136.43, 136.41, 135.46, 135.41, 135.37, 135.2, 131.4, 127.8, 122.4, 122.2, 119.7, 119.0, 112.5, 111.4, 65.0, 35.3, 34.5, 34.3, 32.7, 25.0 ppm. IR (ATR): ν = 3365 (N-H, indole), 1693, 1682 (C=O, aldehyde, ester) cm^{-1}. HRMS (ESI+) *m/z*: calcd. for $C_{28}H_{25}NNaO_3$ $[M + Na]^+$: 446.1727, found: 446.1723.

(*R$_p$*)-2-(1-(4-Chlorobenzoyl)-6-methoxy-2-methyl-1*H*-indol-3-yl)ethyl 4^3-formyl-1,4(1,4)-dibenzenacyclohexaphane-1^2-carboxylate (3o)

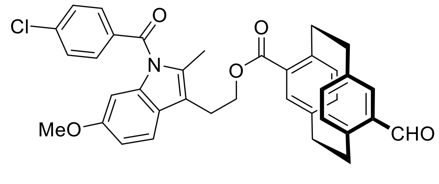

The title compound was synthesized according to the general procedure (reaction time: 48 hours), using paracyclophane **1a** (26.4 mg, 0.1 mmol) and indometacinol (172 mg, 0.5 mmol). The product was purified by column chromatography (hexane/EtOAc – 4:1), affording **3o** (47 mg, 78%) as a colorless oil. Purity was lowered due to the presence (purity: 95%, HPLC) of hardly separable diester by-product (**4o**).

Er = 92:8 (*ee* = 84%), the enantiomeric excess of product **3o** was determined by HPLC using a Chiralpak® IB column (*n*-heptane/*i*-PrOH - 50:50, flow rate = 1.0 ml/min, λ = 209 nm, *t* = 25 °C): t_R = 20.3 min (major), t_R = 46.9 min (minor). $[\alpha]_D^{20}$ = -7.7 (*c* = 0.5, $CHCl_3$). ^{1}H NMR (400 MHz, chloroform-*d*): δ 9.91 (s, 1H), 7.67 – 7.58 (m, 2H), 7.45 – 7.41 (m, 2H), 7.08 (d, *J* = 2.5 Hz, 1H), 7.02 (dd, *J* = 10.9, 2.0 Hz, 2H), 6.92 (d, *J* = 9.0 Hz, 1H), 6.71 (dd, *J* = 9.0, 2.5 Hz, 1H), 6.56 (ddd, *J* = 14.4, 7.8, 2.0 Hz, 2H), 6.44 (d, *J* = 7.9 Hz, 1H), 6.22 (d, *J* = 7.8 Hz, 1H), 4.59 – 4.44 (m, 2H), 4.09 – 4.01 (m, 2H), 3.84 (s, 3H), 3.13 (dddd, *J* = 17.8, 15.8, 8.7, 5.3 Hz, 6H), 2.96 – 2.80 (m, 2H), 2.42 (s, 3H) ppm. ^{13}C{^{1}H} NMR (101 MHz, chloroform-*d*): δ 192.2, 168.4, 167.0, 156.2, 143.0, 142.6, 141.0, 139.9, 139.4, 136.8, 136.5, 136.4, 135.6, 135.5,

135.34, 135.30, 135.28, 135.2, 134.1, 131.3 (2C), 131.2, 131.1, 129.3 (2C), 115.7, 115.2, 111.5, 101.6, 63.9, 55.9, 35.2, 34.5, 34.3, 32.7, 23.9, 13.6 ppm. IR (ATR): v = 1707, 1676, 1680 (C=O, aldehyde, ester, amide), 1261 (C-O, ether), 750 (C-Cl) cm^{-1}. HRMS (ESI+) *m*/*z*: calcd. for $C_{37}H_{32}ClNNaO_5$ $[M + H]^+$: 628.1861, found: 628.1859.

(*R*$_p$)-2-(4-Methylthiazol-5-yl)ethyl 4^3-formyl-1,4(1,4)-dibenzenacyclohexaphane-1^2-carboxylate (3p)

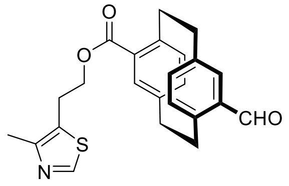

The title compound was synthesized according to the general procedure (reaction time: 15 hours), using paracyclophane **1a** (26.4 mg, 0.1 mmol) and sulfurol (60 µl, 0.5 mmol). The product was purified by column chromatography (hexane/EtOAc – 2:1), affording **3p** (38 mg, 92%) as a white amorphous solid.

Er = 97:3 (*ee* = 94%), the enantiomeric excess of product **3p** was determined by HPLC using a Chiralpak® IB column (*n*-heptane/*i*-PrOH - 60:40, flow rate = 1.0 ml/min, λ = 197 nm, *t* = 25 °C): t_R = 13.6 min (major), t_R = 14.9 min (minor). $[\alpha]_D^{20}$ = +2.6 (*c* = 1.0, $CHCl_3$). ^1H NMR (400 MHz, chloroform-*d*): δ 9.93 (s, 1H), 8.65 (s, 1H), 7.11 (d, *J* = 2.0 Hz, 1H), 7.02 (d, *J* = 2.0 Hz, 1H), 6.64 (dd, *J* = 7.8, 2.0 Hz, 1H), 6.56 (dd, *J* = 7.9, 2.0 Hz, 1H), 6.45 (dd, *J* = 10.5, 7.8 Hz, 2H), 4.51 (qt, *J* = 10.9, 6.5 Hz, 2H), 4.06 (dddd, *J* = 30.4, 12.7, 10.0, 2.5 Hz, 2H), 3.27 (t, *J* = 6.5 Hz, 2H), 3.24 – 3.06 (m, 4H), 2.94 (dddd, *J* = 27.0, 13.0, 10.4, 6.0 Hz, 2H), 2.49 (s, 3H) ppm. $^{13}C\{^1H\}$ NMR (101 MHz, chloroform-*d*): δ 192.2, 166.8, 150.2, 150.1, 143.0, 142.7, 141.0, 139.9, 136.9, 136.6, 136.5, 135.8, 135.4, 135.4, 135.3, 130.8, 64.5, 35.2, 34.6, 34.3, 32.9, 26.2, 15.2. ppm. *one qC is overlapped*. IR (ATR): v = 1709, 1684 (C=O, aldehyde, ester) cm^{-1}. HRMS (ESI+) *m*/*z*: calcd. for $C_{24}H_{23}NNaO_3S$ $[M + Na]^+$: 428.1291, found: 428.1287.

***tert*-Butyl (*R*$_p$, *S*)-2-(((4^3-formyl-1,4(1,4)-dibenzenacyclohexaphane-1^2-carbonyl)oxy)methyl)pyrrolidine-1-carboxylate (3q)**

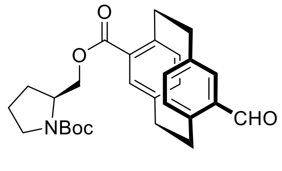

The title compound was synthesized according to the general procedure (reaction time: 72 hours, without full conversion of paracyclophane), using paracyclophane **1a** (26.4 mg, 0.1 mmol) and *N*-Boc-L-prolinol (101 mg, 0.5 mmol). The product was purified by column chromatography (hexane/EtOAc – 5:1), affording **3q** (27 mg, 59%) as a colorless oil. The diastereomeric ratio of **3q**/**3q′** = 20/1 (as determined by ^1H NMR of the crude reaction mixture).

$[\alpha]_D^{20}$ = -25.6 (*c* = 0.6, $CHCl_3$). ^1H NMR (400 MHz, chloroform-*d*): δ 10.09 (s, 1H), 7.27 (d, *J* = 2.0 Hz, 1H), 7.19 (d, *J* = 2.0 Hz, 1H), 6.89 (br s, 1H), 6.75 – 6.65 (m, 2H), 6.62 (d, *J* = 7.8 Hz, 1H), 4.59 (dd, *J* = 10.6, 3.5 Hz, 1H), 4.40 (br s, 2H), 4.32 – 4.14 (m, 3H), 3.54 (br s, 2H), 3.31 (ddd, *J* = 16.0, 7.8, 4.3 Hz, 4H), 3.22 – 2.98 (m, 2H), 2.08 (q, *J* = 7.5 Hz, 4H), 1.65 (s, 9H) ppm. $^{13}C\{^1H\}$ NMR (101 MHz, chloroform-*d*): δ 192.2, 166.9, 157.2, 143.0, 142.5, 141.0, 139.9, 136.9, 136.6, 135.6, 135.4, 135.3, 131.2, 79.8, 65.3, 55.9, 47.0, 35.3, 34.6, 34.4, 32.9, 29.3, 28.7 (3C), 28.4, 23.9, 23.3 ppm, *pyrrolidine carbon signals are broadened or split into doublet-like signals*. IR (ATR): v = 1684 (C=O, aldehyde, ester) cm^{-1}. HRMS (ESI+) *m*/*z*: calcd. for $C_{26}H_{33}NNaO_5$ $[M + Na]^+$: 486.2251, found: 486.2243.

(R_p)-(4*R*)-4-((3*R*,7*R*,8*R*,9*S*,10*S*,13*R*,14*S*,17*R*)-3,7-dihydroxy-10,13-dimethylhexadecahydro-1*H*-cyclopenta[a]phenanthren-17-yl)pentyl 4³-formyl-1,4(1,4)-dibenzenacyclohexaphane-1²-carboxylate (3r)

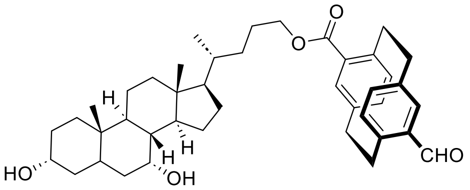

The title compound was synthesized according to the general procedure (reaction time: 48 hours), using paracyclophane **1a** (26.4 mg, 0.1 mmol) and chenodeoxycholanol[4] (190 mg, 0.5 mmol). The product was purified by column chromatography (hexane/EtOAc – 1:1), affording **3r** (43 mg, 67%) as a white amorphous solid. The diastereomeric ratio of **3r**/**3r′** = 20/1 (as determined by ^{1}H NMR of the crude reaction mixture).

$[\alpha]_D^{20}$ = +13.2 (*c* = 1.0, $CHCl_3$). ^{1}H NMR (400 MHz, chloroform-*d*): δ 9.94 (s, 1H), 7.14 (d, *J* = 2.0 Hz, 1H), 7.04 (d, *J* = 2.0 Hz, 1H), 6.75 (dd, *J* = 7.7, 2.0 Hz, 1H), 6.58 – 6.51 (m, 2H), 6.46 (d, *J* = 7.8 Hz, 1H), 4.38 – 4.22 (m, 2H), 4.10 (dtt, *J* = 12.7, 8.0, 3.5 Hz, 2H), 3.86 (q, *J* = 3.1 Hz, 1H), 3.47 (tt, *J* = 11.1, 4.5 Hz, 1H), 3.28 – 3.06 (m, 4H), 3.03-2.86 (m, 2H), 2.28 – 2.15 (m, 1H), 2.05 – 1.77 (m, 6H), 1.76 – 1.08 (m, 21H), 1.00 (d, *J* = 6.6 Hz, 3H), 0.91 (s, 3H), 0.69 (s, 3H) ppm. $^{13}C\{^1H\}$ NMR (101 MHz, chloroform-*d*): δ 192.2, 167.3, 143.0, 142.3, 141.1, 139.8, 136.9, 136.6, 136.6, 135.5, 135.4, 135.4, 135.3, 131.6, 72.2, 68.7, 65.5, 56.1, 50.7, 42.8, 41.6, 40.0, 39.8, 39.6, 35.6, 35.5, 35.3, 35.2, 34.8, 34.6, 34.4, 33.0, 32.9, 32.4, 30.8, 28.5, 25.5, 23.9, 22.9, 20.7, 18.8, 12.0 ppm. IR (ATR): *ν* = 3390 (O-H, alcohol), 1687 (C=O, aldehyde, ester) cm^{-1}. HRMS (ESI+) *m*/*z*: calcd. for $C_{42}H_{56}NaO_5$ $[M + Na]^+$: 663.4020, found: 663.4017.

(R_p)-(4*R*)-4-((3*R*,7*R*,8*R*,9*S*,10*S*,13*R*,14*S*,17*R*)-3,7-*bis*(Methoxymethoxy)-10,13-dimethylhexadecahydro-1*H*-cyclopenta[a]phenanthren-17-yl)pentyl 4³-formyl-1,4(1,4)-dibenzenacyclohexaphane-1²-carboxylate (3s)

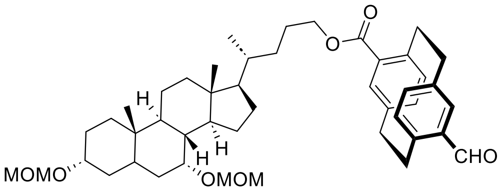

The title compound was synthesized according to the general procedure (reaction time: 48 hours), using paracyclophane **1a** (26.4 mg, 0.1 mmol) and *bis*-OMOM-chenodeoxycholanol[5] (190 mg, 0.5 mmol). The product was purified by column chromatography (hexane/EtOAc - 6:1-4:1), affording **3s** (46 mg, 66%) as an orange oil. The diastereomeric ratio of **3s**/**3s′** = 20/1 (as determined by ^{1}H NMR of the crude reaction mixture).

$[\alpha]_D^{20}$ = -6.7 (*c* = 0.5, $CHCl_3$). ^{1}H NMR (400 MHz, chloroform-*d*): δ 9.94 (s, 1H), 7.14 (d, *J* = 2.0 Hz, 1H), 7.04 (d, *J* = 2.0 Hz, 1H), 6.75 (dd, *J* = 7.8, 2.0 Hz, 1H), 6.58 – 6.50 (m, 2H), 6.46 (d, *J* = 7.8 Hz, 1H), 4.76 – 4.62 (m, 3H), 4.55 (d, *J* = 6.8 Hz, 1H), 4.29 (td, *J* = 6.7, 4.8 Hz, 2H), 4.16 – 4.03 (m, 2H), 3.61 (q, *J* = 2.7 Hz, 1H), 3.37 (s, 3H), 3.35 (s, 3H), 3.26 – 3.16 (m, 3H), 3.11 (ddd, *J* = 13.2, 10.2, 5.7 Hz, 1H), 2.95 (dddd, *J* = 23.1, 12.9, 9.7, 6.6 Hz, 2H), 2.25 (td, *J* = 13.1, 11.5 Hz, 1H), 2.05 – 1.13 (m, 25H), 1.08 (d, *J* = 6.8 Hz, 1H), 1.00 (d, *J* = 6.5 Hz, 3H), 0.91 (s, 3H), 0.67 (s, 3H) ppm. $^{13}C\{^1H\}$ NMR (101 MHz, chloroform-*d*): δ 192.2, 167.3, 143.0, 142.3, 141.1, 139.8, 136.9, 136.61, 136.55, 135.5, 135.42, 135.35, 135.3, 131.6, 96.7, 94.7, 77.4, 74.8, 65.6, 56.3, 56.0, 55.2, 50.0, 42.7, 41.8, 39.6, 36.3, 35.74, 35.71, 35.5, 35.3, 34.6, 34.4, 33.1, 32.9, 32.4, 31.0, 28.5, 27.9, 25.7, 25.6, 23.9, 23.0, 20.8, 18.8, 11.9 ppm. IR (ATR): *ν* = 1709, 1687 (C=O, aldehyde, ester) cm^{-1}. HRMS (ESI+) *m*/*z*: calcd. for $C_{46}H_{64}NaO_7$ $[M + Na]^+$: 751.4544, found: 751.4558.

(R_p)-((2R,3R,4S,5R,6S)-3,4,5,6-Tetramethoxytetrahydro-2H-pyran-2-yl)methyl 4³-formyl-1,4(1,4)-dibenzenacyclohexaphane-1²-carboxylate (3t)

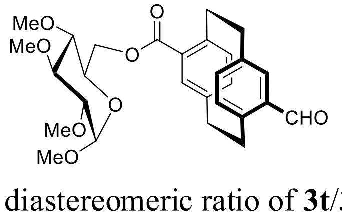

The title compound was synthesized according to the general procedure (reaction time: 48 hours), using paracyclophane **1a** (26.4 mg, 0.1 mmol) and *per*-Me-glucose[6] (118 mg, 0.5 mmol). The product was purified by column chromatography (hexane/EtOAc – 2:1), affording **3t** (36 mg, 72%) as a white amorphous solid. The diastereomeric ratio of **3t**/**3t′** = 9/1 (as determined by ^{1}H NMR of the crude reaction mixture).

$[\alpha]_D^{20}$ = +46.4 (c = 0.8, $CHCl_3$). ^{1}H NMR (only major diastereomer, 400 MHz, chloroform-d): δ 9.94 (s, 1H), 7.16 (d, J = 2.0 Hz, 1H), 7.05 (d, J = 2.0 Hz, 1H), 6.77 (dd, J = 7.8, 2.0 Hz, 1H), 6.57 (dd, J = 7.8, 2.0 Hz, 1H), 6.51 (d, J = 7.8 Hz, 1H), 6.47 (d, J = 7.8 Hz, 1H), 4.85 (d, J = 3.6 Hz, 1H), 4.58 (dd, J = 11.9, 2.2 Hz, 1H), 4.46 (dd, J = 11.8, 5.1 Hz, 1H), 4.14 – 4.06 (m, 2H), 3.89 – 3.81 (m, 1H), 3.66 (s, 3H), 3.61 (s, 3H), 3.54 (s, 3H), 3.45 (s, 3H), 3.22 (ddd, J = 12.7, 7.9, 2.4 Hz, 5H), 3.17 – 2.85 (m, 4H) ppm. $^{13}C\{^1H\}$ NMR (only major diastereomer, 101 MHz, chloroform-d): δ 192.2, 167.0, 160.9, 143.0, 142.5, 141.1, 139.9, 136.9, 136.6, 136.6, 135.7, 135.4, 135.4, 131.1, 97.7, 83.6, 82.0, 80.3, 68.8, 63.9, 61.2, 60.8, 59.2, 55.5, 35.5, 34.6, 34.4, 32.9 ppm. IR (ATR): v = 1697, 1682 (C=O, aldehyde, ester), 1036 (C-O, ether) cm^{-1}. HRMS (ESI+) m/z: calcd. for $C_{28}H_{34}NaO_8$ [M + Na]$^+$: 521.2146, found: 521.2148.

(R_p)-5-((3aS,4S,6aR)-1,3-Dibenzyl-2-oxohexahydro-1H-thieno[3,4-d]imidazol-4-yl)pentyl 4³-formyl-1,4(1,4)-dibenzenacyclohexaphane-1²-carboxylate (3u)

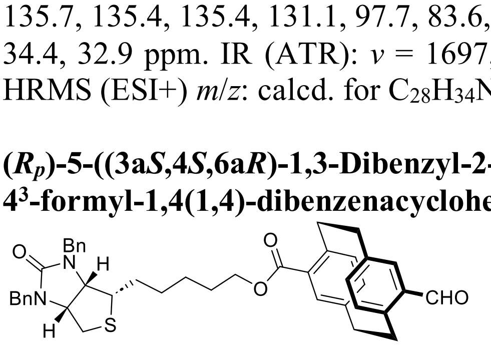

The title compound was synthesized according to the general procedure (reaction time: 15 hours), using paracyclophane **1a** (26.4 mg, 0.1 mmol) and N-Bn-biotinol[7] (205 mg, 0.5 mmol). The product was purified by column chromatography (hexane/EtOAc – 3:1-2:1), affording **3u** (56 mg, 83%) as a white amorphous solid. The diastereomeric ratio of **3u**/**3u′** = 20/1 (as determined by ^{1}H NMR of the crude reaction mixture).

$[\alpha]_D^{20}$ = -23.6 (c = 0.6, $CHCl_3$). ^{1}H NMR (400 MHz, chloroform-d): δ 9.94 (s, 1H), 7.37 – 7.20 (m, 10H), 7.15 (d, J = 2.0 Hz, 1H), 7.05 (d, J = 2.0 Hz, 1H), 6.76 (dd, J = 7.8, 2.0 Hz, 1H), 6.59 – 6.53 (m, 2H), 6.47 (d, J = 7.8 Hz, 1H), 5.08 (d, J = 15.1 Hz, 1H), 4.75 (d, J = 15.2 Hz, 1H), 4.33 (td, J = 6.7, 2.3 Hz, 2H), 4.19 – 4.05 (m, 3H), 4.01 – 3.93 (m, 2H), 3.87 (dd, J = 9.5, 5.5 Hz, 1H), 3.29 – 3.17 (m, 3H), 3.17 – 3.06 (m, 2H), 2.97 (dddd, J = 29.1, 12.8, 9.8, 6.4 Hz, 2H), 2.80 – 2.62 (m, 2H), 1.88 – 1.75 (m, 2H), 1.66 (td, J = 11.5, 9.6, 5.7 Hz, 3H), 1.60 – 1.34 (m, 4H) ppm. $^{13}C\{^1H\}$ NMR (101 MHz, chloroform-d): δ 192.2, 167.2, 161.2, 143.1, 142.4, 141.0, 139.9, 137.1, 137.0, 136.9, 136.6, 136.5, 135.5, 135.4, 135.31, 135.29, 131.5, 128.9 (2C), 128.8 (2C), 128.4 (4C), 127.8, 127.8, 64.8, 62.8, 61.3, 54.5, 48.1, 46.8, 35.4, 34.9, 34.6, 34.4, 32.8, 29.0, 28.9, 28.9, 26.2 ppm. IR (ATR): v = 1687 (C=O, aldehyde, ester, amide) cm^{-1}. HRMS (ESI+) m/z: calcd. for $C_{42}H_{44}N_2NaO_4S$ [M + Na]$^+$: 695.2914, found: 695.2919.

(R_p,)-(S)-3,7-Dimethyloct-6-en-1-yl 4³-formyl-1,4(1,4)-dibenzenacyclohexaphane-1²-carboxylate (3v)

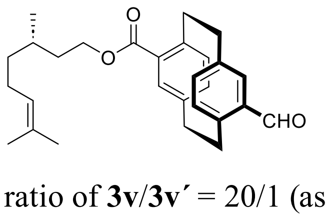

The title compound was synthesized according to the general procedure (reaction time: 15 hours), using paracyclophane **1a** (26.4 mg, 0.1 mmol) and *(-)-β*-citronellol (91 µl, 0.5 mmol). The product was purified by column chromatography (hexane/EtOAc – 16:1), affording **3v** (36 mg, 84%) as a yellow oil. The diastereomeric ratio of **3v**/**3v′** = 20/1 (as determined by ^{1}H NMR of the crude reaction mixture).

$[\alpha]_D^{20}$ = +3.4 (c = 0.9, $CHCl_3$). ^{1}H NMR (400 MHz, chloroform-d): δ 9.94 (s, 1H), 7.13 (d, J = 2.1 Hz, 1H), 7.04 (d, J = 2.0 Hz, 1H), 6.75 (dd, J = 7.8, 2.0 Hz, 1H), 6.58 – 6.51 (m, 2H), 6.47

(d, J = 7.8 Hz, 1H), 5.13 (dddd, J = 7.1, 5.7, 2.9, 1.5 Hz, 1H), 4.46 – 4.28 (m, 2H), 4.17 – 4.03 (m, 2H), 3.29 – 3.06 (m, 4H), 2.95 (dddd, J = 26.3, 13.0, 10.0, 6.5 Hz, 2H), 2.05 (tq, J = 15.0, 7.5 Hz, 2H), 1.95 – 1.76 (m, 1H), 1.74 – 1.53 (m, 8H), 1.45 (dddd, J = 14.6, 9.4, 6.6, 5.2 Hz, 1H), 1.27 (dddd, J = 13.7, 9.3, 7.7, 6.1 Hz, 1H), 1.01 (d, J = 6.4 Hz, 3H) ppm. $^{13}C\{^1H\}$ NMR (101 MHz, chloroform-d): δ 192.2, 167.3, 143.0, 142.4, 141.1, 139.8, 136.9, 136.60, 136.57, 135.46, 135.43, 135.37, 135.3, 131.7, 124.6, 63.5, 37.2, 35.9, 35.4, 34.6, 34.4, 32.9, 29.8, 25.9, 25.6, 19.6, 17.8 ppm. IR (ATR): ν = 1707, 1689 (C=O, aldehyde, ester) cm^{-1}. HRMS (ESI+) m/z: calcd. for $C_{28}H_{34}NaO_3$ [M + Na]$^+$: 441.2400, found: 441.2394.

(R_p)-*S*-Ethyl 4^3-formyl-1,4(1,4)-dibenzenacyclohexaphane-1^2-carbothioate (3w)

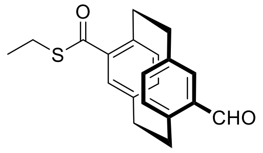

The title compound was synthesized according to the general procedure (reaction time: 15 hours), using paracyclophane **1a** (26.4 mg, 0.1 mmol) and ethanethiol (36 µl, 0.5 mmol). The product was purified by column chromatography (hexane/EtOAc – 9:1), affording **3w** (18 mg, 56%) as a white amorphous solid.

Er = 78:22 (*ee* = 57%), the enantiomeric excess of product **3w** was determined by HPLC using a Chiralpak® IB column (n-heptane/i-PrOH - 80:20, flow rate = 1.0 ml/min, λ = 190 nm, t = 25 °C): t_R = 6.8 min (major), t_R = 8.3 min (minor). $[\alpha]_D^{20}$ = +9.5 (c = 0.5, $CHCl_3$). ^1H NMR (400 MHz, chloroform-d): δ 9.95 (s, 1H), 7.05 (dd, J = 6.1, 1.9 Hz, 2H), 6.88 (dd, J = 7.8, 2.0 Hz, 1H), 6.58 – 6.50 (m, 2H), 6.46 (d, J = 7.8 Hz, 1H), 4.11 (ddd, J = 12.9, 10.2, 2.4 Hz, 1H), 3.82 (ddd, J = 13.0, 10.3, 2.8 Hz, 1H), 3.31 – 3.10 (m, 4H), 3.09 – 2.88 (m, 4H), 1.38 (t, J = 7.4 Hz, 3H) ppm. $^{13}C\{^1H\}$ NMR (101 MHz, chloroform-d): δ 193.6, 192.2, 142.9, 141.1, 140.2, 139.7, 138.1, 136.73, 136.66, 136.65, 135.7, 135.6, 135.4, 133.0, 34.7 (2C), 34.5, 32.9, 24.1, 15.0 ppm. IR (ATR): ν = 1676, 1660 (C=O, aldehyde, thioester) cm^{-1}. HRMS (ESI+) m/z: calcd. for $C_{20}H_{20}NaO_2S$ [M + Na]$^+$: 347.1076, found: 347.1079.

(R_p)-*S*-Phenethyl 4^3-formyl-1,4(1,4)-dibenzenacyclohexaphane-1^2-carbothioate (3x)

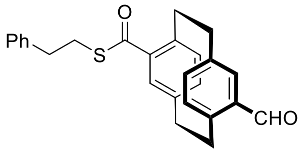

The title compound was synthesized according to the general procedure (reaction time: 15 hours), using paracyclophane **1a** (26.4 mg, 0.1 mmol) and 2-phenylethane-1-thiol (67 µl, 0.5 mmol). The product was purified by column chromatography (hexane/EtOAc – 10:1), affording **3x** (25 mg, 62%) as a white amorphous solid.

Er = 75:25 (*ee* = 50%), the enantiomeric excess of product **3x** was determined by HPLC using a Chiralpak® IB column (n-heptane/i-PrOH - 80:20, flow rate = 1.0 ml/min, λ = 190 nm, t = 25 °C): t_R = 9.4 min (major), t_R = 10.9 min (minor). $[\alpha]_D^{20}$ = +8.5 (c = 0.7, $CHCl_3$). ^1H NMR (400 MHz, chloroform-d): δ 9.94 (s, 1H), 7.38 – 7.29 (m, 4H), 7.28 – 7.23 (m, 1H), 7.07 – 7.01 (m, 2H), 6.80 (dd, J = 7.8, 2.0 Hz, 1H), 6.55 (dd, J = 7.8, 1.9 Hz, 1H), 6.47 (dd, J = 17.4, 7.8 Hz, 2H), 4.11 (ddd, J = 12.9, 10.2, 2.4 Hz, 1H), 3.78 (ddd, J = 13.0, 10.2, 2.9 Hz, 1H), 3.36 – 3.29 (m, 2H), 3.28 – 2.85 (m, 8H) ppm. $^{13}C\{^1H\}$ NMR (101 MHz, chloroform-d): δ 193.0, 192.2, 142.9, 141.0, 140.2, 140.1, 139.7, 138.0, 136.70, 136.66, 136.64, 135.8, 135.6, 135.3, 133.0, 128.9 (2C), 128.7 (2C), 126.7, 36.1, 34.7 (2C), 34.4, 32.9, 30.9 ppm. IR (ATR): ν = 1684. 1649 (C=O, aldehyde, thioester) cm^{-1}. HRMS (ESI+) m/z: calcd. for $C_{26}H_{24}NaO_2S$ [M + Na]$^+$: 423.1389, found: 423.1381.

(R_p)-Methyl 4²-formyl-1,4(1,4)-dibenzenacyclohexaphane-1²-carboxylate (5a)

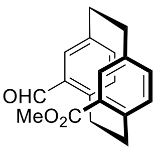

The title compound was synthesized according to the general procedure (reaction time: 15 hours), using paracyclophane **1b** (26.4 mg, 0.1 mmol) and methanol (20 µl, 0.5 mmol). The product was purified by column chromatography (hexane/EtOAc - 9:1), affording **5a** (27 mg, 91%) as a white crystalline solid. Crystals suitable for X-ray analysis were grown by the dissolution of **5a** in a minimal amount of boiling *i*-PrOH, followed by standing at room temperature overnight.

m.p. = 189.6-190.8 °C (*i*-PrOH). *Er* = 99.5:0.5 (*ee* = 99%), the enantiomeric excess of product **5a** was determined by HPLC using a Chiralpak® IB column (*n*-heptane/*i*-PrOH - 80:20, flow rate = 1.0 ml/min, λ = 190 nm, *t* = 25 °C): t_R = 9.3 min (major), t_R = 14.3 min (minor). $[\alpha]_D^{20}$ = +82.2 (*c* = 0.9, $CHCl_3$). ^1H NMR (400 MHz, chloroform-*d*): δ 9.92 (s, 1H), 7.07 (t, *J* = 2.1 Hz, 2H), 6.71 (ddd, *J* = 7.8, 4.7, 2.0 Hz, 2H), 6.63 (dd, *J* = 12.5, 7.8 Hz, 2H), 4.21 – 4.05 (m, 2H), 3.82 (s, 3H), 3.18 – 2.96 (m, 6H) ppm. $^{13}C\{^1H\}$ NMR (101 MHz, chloroform-*d*): δ 190.8, 167.2, 143.7, 142.3, 140.3, 139.9, 138.3, 136.7, 136.3, 136.2, 135.9, 134.6, 133.9, 131.0, 52.1, 35.2, 34.9, 34.8, 31.3 ppm. IR (KBr): ν = 1709, 1674 (C=O, aldehyde, ester) cm^{-1}. HRMS (ESI+) *m*/*z*: calcd. for $C_{19}H_{18}O_3Na$ $[M + Na]^+$: 317.1148, found: 317.1155.

(S_p)-Methyl 4²-formyl-1,4(1,4)-dibenzenacyclohexaphane-1²-carboxylate (*ent*-5a)

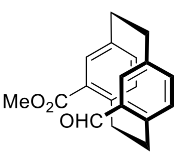

The title compound was synthesized according to the general procedure (reaction time: 15 hours), using paracyclophane **1b** (26.4 mg, 0.1 mmol), methanol (20 µl, 0.5 mmol), and *ent*-*pre*-**C1** as a catalyst. The product was purified by column chromatography (hexane/EtOAc - 9:1), affording *ent*-**5a** (26 mg, 89%) as a white crystalline solid. Crystals suitable for X-ray analysis were grown by dissolving *ent*-**5a** in a minimal amount of boiling *i*-PrOH, followed by standing at room temperature overnight.

m.p. = 188.0-190.0 °C (*i*-PrOH). *Er* = 99:1 (*ee* = 97%), the enantiomeric excess of product *ent*-**5a** was determined by HPLC using a Chiralpak® IB column (*n*-heptane/*i*-PrOH - 80:20, flow rate = 1.0 ml/min, λ = 190 nm, *t* = 25 °C): t_R = 9.3 min (minor), t_R = 14.3 min (major). $[\alpha]_D^{20}$ = -75.7 (*c* = 0.9, $CHCl_3$). Other analytical data agree with data on the opposite enantiomer (**5a**).

(R_p)-Ethyl 4²-formyl-1,4(1,4)-dibenzenacyclohexaphane-1²-carboxylate (5b)

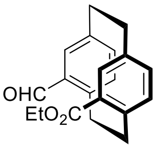

The title compound was synthesized according to the general procedure (reaction time: 15 hours), using paracyclophane **1b** (26.4 mg, 0.1 mmol) and ethanol (29 µl, 0.5 mmol). The product was purified by column chromatography (hexane/EtOAc - 8:1), affording **5b** (28 mg, 92%) as a white amorphous solid. *Er* = 99.5:0.5 (*ee* = 99%), the enantiomeric excess of product **5b** was determined by HPLC using a Chiralpak® IB column (*n*-heptane/*i*-PrOH - 80:20, flow rate = 1.0 ml/min, λ = 196 nm, *t* = 25 °C): t_R = 7.6 min (major), t_R = 10.9 min (minor). $[\alpha]_D^{20}$ = +62.7 (*c* = 1.0, $CHCl_3$). ^1H NMR (400 MHz, chloroform-*d*): δ 9.92 (s, 1H), 7.06 (dd, *J* = 4.5, 2.0 Hz, 2H), 6.70 (ddd, *J* = 7.9, 2.1, 1.0 Hz, 2H), 6.62 (dd, *J* = 9.5, 7.8 Hz, 2H), 4.39 – 4.21 (m, 2H), 4.21 – 4.05 (m, 2H), 3.25 – 2.92 (m, 6H), 1.39 (t, *J* = 7.1 Hz, 3H) ppm. $^{13}C\{^1H\}$ NMR (101 MHz, chloroform-*d*): δ 190.8, 166.8, 143.6, 142.1, 140.3, 139.8, 138.3, 136.7, 136.3, 136.1, 135.9, 134.5, 134.1, 131.3, 61.1, 35.2, 34.9, 34.8, 31.4, 14.3 ppm. IR (ATR): ν = 1701, 1682 (C=O, aldehyde, ester) cm^{-1}. HRMS (ESI+) *m*/*z*: calcd. for $C_{20}H_{20}NaO_3$ $[M + Na]^+$: 331.1305, found: 331.1298.

(R_p)-Isopropyl 4^2-formyl-1,4(1,4)-dibenzenacyclohexaphane-1^2-carboxylate (5c)

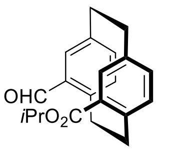

The title compound was synthesized according to the general procedure (reaction time: 72 hours, without full conversion **1b**), using paracyclophane **1b** (26.4 mg, 0.1 mmol) and isopropanol (38 µl, 0.5 mmol). The product was purified by column chromatography (hexane/EtOAc - 9:1), affording **5c** (19 mg, 56%) as a yellow oil.

Er = 98:2 (*ee* = 97%), the enantiomeric excess of product **5c** was determined by HPLC using a Chiralpak® IB column (*n*-heptane/*i*-PrOH - 80:20, flow rate = 1.0 ml/min, λ = 215 nm, *t* = 25 °C): t_R = 6.3 min (major), t_R = 7.8 min (minor). $[\alpha]_D^{20}$ = +36.4 (*c* = 0.2, $CHCl_3$). ^1H NMR (400 MHz, chloroform-*d*): δ 9.94 (s, 1H), 7.04 (dd, *J* = 12.7, 2.0 Hz, 2H), 6.70 (ddd, *J* = 8.3, 6.4, 2.0 Hz, 2H), 6.62 (dd, *J* = 7.8, 3.4 Hz, 2H), 5.14 (p, *J* = 6.3 Hz, 1H), 4.23 – 4.04 (m, 2H), 3.20 – 2.89 (m, 6H), 1.36 (dd, *J* = 10.4, 6.3 Hz, 6H) ppm. $^{13}C\{^1H\}$ NMR (101 MHz, chloroform-*d*): δ 190.9, 166.5, 143.6, 142.0, 140.3, 139.8, 138.2, 136.8, 136.3, 136.0, 135.9, 134.4, 134.2, 131.7, 68.6, 35.1, 34.9, 34.8, 31.6, 22.0, 21.9 ppm. IR (ATR): ν = 1710, 1682 (C=O, aldehyde, ester) cm^{-1}. HRMS (ESI+) *m*/*z*: calcd. for $C_{21}H_{22}NaO_3$ $[M + Na]^+$: 345.1461, found: 345.1457.

(R_p)-2-Methoxyethyl 4^2-formyl-1,4(1,4)-dibenzenacyclohexaphane-1^2-carboxylate (5d)

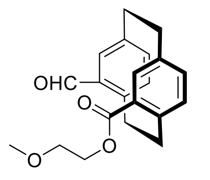

The title compound was synthesized according to the general procedure (reaction time: 15 hours), using paracyclophane **1b** (26.4 mg, 0.1 mmol) and 2-methoxyethan-1-ol (40 µl, 0.5 mmol). The product was purified by column chromatography (hexane/EtOAc - 9:1), affording **5d** (34 mg, 86%) as a yellow oil.

Er = 99.5:0.5 (*ee* = 99%), the enantiomeric excess of product **5d** was determined by HPLC using a Chiralpak® IB column (*n*-heptane/*i*-PrOH - 80:20, flow rate = 1.0 ml/min, λ = 215 nm, *t* = 25 °C): t_R = 17.4 min (major), t_R = 23.4 min (minor). $[\alpha]_D^{20}$ = +48.7 (*c* = 1.1, $CHCl_3$). ^1H NMR (400 MHz, chloroform-*d*): δ 9.92 (s, 1H), 7.07 (dd, *J* = 8.4, 2.0 Hz, 2H), 6.70 (ddd, *J* = 7.8, 1.9, 1.2 Hz, 2H), 6.62 (t, *J* = 8.0 Hz, 2H), 4.41 (ddd, *J* = 12.1, 5.8, 4.1 Hz, 1H), 4.32 (ddd, *J* = 12.0, 5.4, 3.8 Hz, 1H), 4.19 – 4.07 (m, 2H), 3.77 – 3.67 (m, 2H), 3.45 (s, 3H), 3.17 – 2.95 (m, 6H) ppm. $^{13}C\{^1H\}$ NMR (101 MHz, chloroform-*d*): δ 191.2, 166.7, 143.6, 142.3, 140.3, 139.9, 138.3, 136.7, 136.33, 136.27, 135.9, 134.7, 134.2, 130.9, 70.6, 64.0, 59.0, 35.2, 34.9, 34.8, 31.4 ppm. IR (ATR): ν = 1701, 1682 (C=O, aldehyde, ester), 1026 (C-O, ether) cm^{-1}. HRMS (ESI+) *m*/*z*: calcd. for $C_{21}H_{22}NaO_4$ $[M + Na]^+$: 361.1410, found: 361.1406.

(R_p)-Pent-3-yn-1-yl 4^2-formyl-1,4(1,4)-dibenzenacyclohexaphane-1^2-carboxylate (5e)

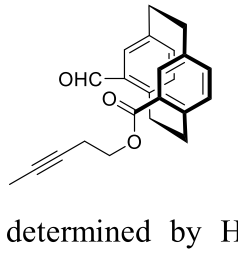

The title compound was synthesized according to the general procedure (reaction time: 15 hours), using paracyclophane **1b** (26.4 mg, 0.1 mmol) and pent-3-yn-1-ol (46 µl, 0.5 mmol). The product was purified by column chromatography (hexane/EtOAc - 8:1), affording **5e** (33 mg, 96%) as a yellow amorphous solid.

Er = 99.5:0.5 (*ee* = 99%), the enantiomeric excess of product **5e** was determined by HPLC using a Chiralpak® IB column (*n*-heptane/*i*-PrOH - 80:20, flow rate = 1.0 ml/min, λ = 201 nm, *t* = 25 °C): t_R = 9.3 min (major), t_R = 12.4 min (minor). $[\alpha]_D^{20}$ = +38.5 (*c* = 0.9, $CHCl_3$). ^1H NMR (400 MHz, chloroform-*d*): δ 9.92 (s, 1H), 7.07 (dd, *J* = 9.5, 2.0 Hz, 2H), 6.70 (ddd, *J* = 7.8, 3.5, 2.0 Hz, 2H), 6.63 (dd, *J* = 10.5, 7.8 Hz, 2H), 4.35 – 4.22 (m, 2H), 4.22 – 4.08 (m, 2H), 3.21 – 2.94 (m, 6H), 2.66 – 2.57 (m, 2H), 1.82 (t, *J* = 2.5 Hz, 3H) ppm. $^{13}C\{^1H\}$ NMR (101 MHz, chloroform-*d*): δ 191.0, 166.6, 143.6, 142.2, 140.3, 139.9, 138.2, 136.8, 136.3, 136.3, 135.9, 134.7, 134.3, 131.0, 77.4, 75.3, 63.6, 35.2, 34.9, 34.8, 31.4, 19.3, 3.7 ppm. IR (KBr): ν = 1699, 1684 (C=O, aldehyde, ester) cm^{-1}. HRMS (ESI+) *m*/*z*: calcd. for $C_{23}H_{22}NaO_3$ $[M + Na]^+$: 369.1461, found: 369.1457.

(R_p)-Phenethyl 4[2]-formyl-1,4(1,4)-dibenzenacyclohexaphane-1[2]-carboxylate (5f)

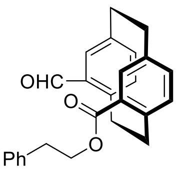

The title compound was synthesized according to the general procedure (reaction time: 15 hours), using paracyclophane **1b** (26.4 mg, 0.1 mmol) and 2-phenylethan-1-ol (60 µl, 0.5 mmol). The product was purified by column chromatography (hexane/EtOAc - 8:1), affording **5f** (36 mg, 94%) as a yellow amorphous solid.

Er = 99.5:0.5 (*ee* = 99%), the enantiomeric excess of product **5f** was determined by HPLC using a Chiralpak® IB column (*n*-heptane/*i*-PrOH - 80:20, flow rate = 1.0 ml/min, λ = 213 nm, t = 25 °C): t_R = 10.2 min (major), t_R = 13.0 min (minor). $[\alpha]_D^{20}$ = +46.1 (c = 1.2, $CHCl_3$). ^1H NMR (400 MHz, chloroform-*d*): δ 9.77 (s, 1H), 7.42 – 7.32 (m, 4H), 7.31 – 7.21 (m, 1H), 6.97 (dd, J = 4.5, 2.0 Hz, 2H), 6.69 (ddd, J = 7.1, 4.7, 1.9 Hz, 2H), 6.60 (dd, J = 12.6, 7.8 Hz, 2H), 4.44 (q, J = 6.8 Hz, 2H), 4.15 – 3.98 (m, 2H), 3.20 – 2.86 (m, 8H) ppm. $^{13}C\{^1H\}$ NMR (101 MHz, chloroform-*d*): δ 191.0, 166.6, 143.6, 142.2, 140.2, 139.8, 138.3, 138.2, 136.7, 136.3, 136.2, 135.9, 134.6, 134.2, 131.1, 129.2 (2C), 128.7 (2C), 126.7, 65.5, 35.1, 35.0, 34.9, 34.8, 31.4 ppm. IR (KBr): ν = 1703, 1684 (C=O, aldehyde, ester) cm^{-1}. HRMS (ESI+) *m/z*: calcd. for $C_{26}H_{24}NaO_3$ $[M + Na]^+$: 407.1618, found: 407.1616.

(R_p)-2-(4-Methylthiazol-5-yl)ethyl 4[2]-formyl-1,4(1,4)-dibenzenacyclohexaphane-1[2]-carboxylate (5g)

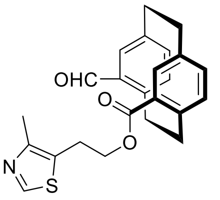

The title compound was synthesized according to the general procedure (reaction time: 15 hours), using paracyclophane **1b** (26.4 mg, 0.1 mmol) and sulfurol (60 µl, 0.5 mmol). The product was purified by column chromatography (hexane/EtOAc - 3:1-1:1), affording **5g** (38 mg, 95%) as a colorless oil.

Er = 99:1 (*ee* = 98%), the enantiomeric excess of product **5g** was determined by HPLC using a Chiralpak® IB column (*n*-heptane/*i*-PrOH - 60:40, flow rate = 1.0 ml/min, λ = 252 nm, t = 25 °C): t_R = 10.1 min (major), t_R = 13.1 min (minor). $[\alpha]_D^{20}$ = +40.1 (c = 1.6, $CHCl_3$). ^1H NMR (400 MHz, chloroform-*d*): δ 9.85 (s, 1H), 8.62 (s, 1H), 7.03 (dd, J = 6.9, 2.0 Hz, 2H), 6.70 (ddd, J = 7.8, 5.0, 2.0 Hz, 2H), 6.62 (dd, J = 11.7, 7.9 Hz, 2H), 4.37 (t, J = 6.8 Hz, 2H), 4.15 – 4.02 (m, 2H), 3.23 (t, J = 6.7 Hz, 2H), 3.19 – 2.92 (m, 6H), 2.48 (s, 3H) ppm. $^{13}C\{^1H\}$ NMR (101 MHz, chloroform-*d*): δ 190.8, 166.4, 150.1, 150.0, 143.6, 142.4, 140.3, 139.9, 138.3, 136.6, 136.4, 136.4, 135.9, 134.6, 133.9, 130.6, 127.2, 64.6, 35.0, 34.9, 34.8, 31.4, 25.9, 15.1 ppm. IR (ATR): ν = 1714, 1676 (C=O, aldehyde, ester) cm^{-1}. HRMS (ESI+) *m/z*: calcd. for $C_{24}H_{23}NNaO_3S$ $[M + Na]^+$: 428.1291, found: 428.1290.

(R_p)-*S*-Ethyl 4[2]-formyl-1,4(1,4)-dibenzenacyclohexaphane-1[2]-carbothioate (5h)

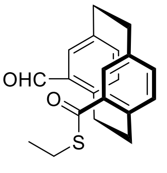

The title compound was synthesized according to the general procedure (reaction time: 72 hours, without full conversion **1b**), using paracyclophane **1b** (26.4 mg, 0.1 mmol) and ethanethiol (36 µl, 0.5 mmol). The product was purified by column chromatography (hexane/EtOAc - 8:1), affording **5h** (23 mg, 71%) as a white amorphous solid.

Er = 86:14 (*ee* = 73%), the enantiomeric excess of product **5h** was determined by HPLC using a Chiralpak® IB column (*n*-heptane/*i*-PrOH - 80:20, flow rate = 1.0 ml/min, λ = 211 nm, t = 25 °C): t_R = 8.5 min (major), t_R = 14.3 min (minor). $[\alpha]_D^{20}$ = +40.4 (c = 0.8, $CHCl_3$). ^1H NMR (400 MHz, chloroform-*d*): δ 10.01 (s, 1H), 7.08 (d, J = 2.0 Hz, 1H), 6.98 (d, J = 1.9 Hz, 1H), 6.72 (ddd, J = 12.1, 7.8, 2.0 Hz, 2H), 6.62 (dd, J = 13.2, 7.8 Hz, 2H), 4.13 (ddd, J = 12.7, 10.4, 4.5 Hz, 1H), 3.91 (ddd, J = 13.1, 10.4, 2.8 Hz, 1H), 3.24 – 2.83 (m, 8H), 1.34 (t, J = 7.4 Hz, 3H) ppm. $^{13}C\{^1H\}$ NMR (101 MHz, chloroform-*d*): δ 193.4, 190.2, 143.8, 140.3, 140.1, 139.4, 138.2, 137.3, 136.6, 136.43, 136.39, 135.8, 132.9, 132.6, 34.94, 34.90, 34.8, 31.7, 24.2, 14.7

ppm. IR (ATR): ν = 1674, 1645 (C=O, aldehyde, thioester) cm^{-1}. HRMS (ESI+) *m*/*z*: calcd. for $C_{20}H_{20}NaO_2S$ $[M + Na]^+$: 347.1076, found: 347.1080.

Follow-up transformations and related organocatalysis

(*R*$_p$)-Methyl 4^2-formyl-1,4(1,4)-dibenzenacyclohexaphane-1^2-carboxylate (5a)

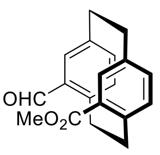

The round bottom flask (100 ml) was charged with **1b** (1000 mg, 3.8 mmol, 1.0 equiv.), *pre*-**C1** (294 mg, 0.8 mmol, 0.2 equiv.), DQ (1855 mg, 4.5 mmol, 1.2 equiv.), and Cs_2CO_3 (2467 mg, 7.6 mmol, 2.0 equiv.), followed by precooled DCM (38 ml, 0 °C, cryocooler) and methanol (0.8 ml, 18.9 mmol, 5.0 equiv.). The reaction was stirred for 48 hours (TLC control) at 0 °C (cryocooler). Once the reaction was completed, the heterogeneous mixture was filtered through a short pad of Celite and washed with DCM (3 × 10 ml). The filtrate was evaporated under reduced pressure. The crude product was purified by column chromatography (eluting by hexane/EtOAc – 9:1), affording **5a** (976 mg, 88%) as a white crystalline solid.
Er = 99:1 (98% *ee*).
All analytical data agree with data on this compound prepared on a 0.1-mmol scale.

(*S*$_p$)-Methyl 4^2-((ethylthio)carbonyl)-1,4(1,4)-dibenzenacyclohexaphane-1^2-carboxylate (7)

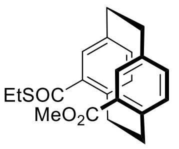

The vial (4 ml) was charged with **5a** (29.4 mg, 0.1 mmol, 1.0 equiv., 99.5:0.5 *er*), *pre*-**C4** (7.3 mg, 0.02 mmol, 0.2 equiv.), DQ (49.0 mg, 0.12 mmol, 1.2 equiv.), and Cs_2CO_3 (48.9 mg, 0.15 mmol, 1.5 equiv.), followed by DCM (1.0 ml), and ethanethiol (36 µl, 0.5 mmol, 5.0 equiv.) at room temperature. The mixture was heated to 40 °C and stirred at this temperature overnight (15 hours). Once the aldehyde was no longer detected (TLC), the reaction mixture was cooled to room temperature, and the solvent was evaporated. The crude product was purified by column chromatography (eluting with hexane/EtOAc- 12:1), affording **7** (31 mg, 87%) as a white amorphous solid.
Er = 99:1 (*ee* = 98%), the enantiomeric excess of product **7** was determined by HPLC using a Chiralpak® IC column (*n*-heptane/*i*-PrOH - 80:20, flow rate = 1.0 ml/min, λ = 212 nm, *t* = 25 °C): t_R = 7.6 min (major), t_R = 16.7 min (minor). $[\alpha]_D^{20}$ = +47.5 (*c* = 1.3, $CHCl_3$). ^1H NMR (400 MHz, chloroform-*d*): δ 7.15 (d, *J* = 1.9 Hz, 1H), 7.06 (d, *J* = 1.8 Hz, 1H), 6.67 (ddd, *J* = 9.2, 7.8, 1.9 Hz, 2H), 6.61 (dd, *J* = 7.8, 2.7 Hz, 2H), 4.20 (ddd, *J* = 11.4, 10.3, 3.2 Hz, 1H), 3.98 – 3.88 (m, 1H), 3.83 (s, 3H), 3.19 – 2.88 (m, 8H), 1.35 (t, *J* = 7.4 Hz, 3H) ppm. $^{13}C\{^1H\}$ NMR (101 MHz, chloroform-*d*): δ 192.9, 167.3, 142.5, 140.0 (2C, *overlapped*), 139.4, 137.4, 136.5, 136.3, 136.1, 136.0, 134.2, 132.1, 130.7, 51.8, 34.87, 34.84 (2C, *overlapped*), 34.6, 24.0, 14.8 ppm. IR (ATR): ν = 1709, 1651 (C=O, aldehyde, thioester) cm^{-1}. HRMS (ESI+) *m*/*z*: calcd. for $C_{21}H_{22}O_3SNa$ $[M + Na]^+$: 377.1182, found: 377.1181.

(*R*$_p$)-Methyl (*E*)-4^2-(3-ethoxy-3-oxoprop-1-en-1-yl)-1,4(1,4)-dibenzenacyclohexaphane-1^2-carboxylate (8)

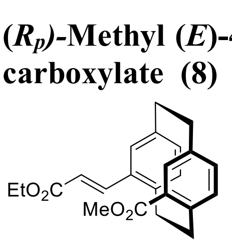

The vial (4 ml) was charged with **5a** (29.4 mg, 0.1 mmol, 1.0 equiv., 99.5:0.5 *er*), ethyl 2-(triphenyl-λ^5-phosphaneylidene)acetate (104.4 mg, 0.3 mmol, 3.0 equiv.), followed by DCM (1.0 ml) at room temperature. The mixture was heated to 60 °C and stirred at this temperature overnight (15 hours). Once the aldehyde was no longer detected (by TLC), the reaction mixture was cooled to room temperature, and the solvent was evaporated. The crude product was purified by column chromatography (eluting with hexane/EtOAc- 9:1), affording **8** (36 mg,

98%) as a white amorphous solid. The ratio of *E*/*Z* isomers of **8** ≥ 20/1 (as determined by ^{1}H NMR of the crude reaction mixture).
Er = 99.5:0.5 (*ee* = 99%), the enantiomeric excess of product **8** was determined by HPLC using a Chiralpak® IC column (*n*-heptane/*i*-PrOH - 80:20, flow rate = 1.0 ml/min, λ = 262 nm, *t* = 25 °C): t_R = 13.0 min (minor), t_R = 20.6 min (major). $[\alpha]_D^{20}$ = +250.6 (*c* = 1.7, $CHCl_3$). ^{1}H NMR (400 MHz, chloroform-*d*): δ 7.74 (d, *J* = 15.9 Hz, 1H), 7.22 (d, *J* = 2.0 Hz, 1H), 6.74 (d, *J* = 1.7 Hz, 1H), 6.69 (dd, *J* = 7.8, 2.0 Hz, 1H), 6.61 (d, *J* = 7.8 Hz, 1H), 6.59 – 6.50 (m, 2H), 6.16 (d, *J* = 15.9 Hz, 1H), 4.32 – 4.19 (m, 3H), 3.76 (s, 3H), 3.70 – 3.60 (m, 1H), 3.17 – 2.92 (m, 6H), 1.35 (t, *J* = 7.1 Hz, 3H) ppm. $^{13}C\{^1H\}$ NMR (101 MHz, chloroform-*d*): δ 167.2, 166.7, 142.8, 141.8, 140.8, 140.0, 139.6, 136.5, 136.4, 135.1, 135.1, 134.9, 134.6, 130.3, 129.5, 118.6, 60.5, 51.7, 35.2, 35.0, 35.0, 32.3, 14.5 ppm. IR (ATR): ν = 1717, 1697 (C=O, ester) cm^{-1}. HRMS (ESI+) *m*/*z*: calcd. for $C_{23}H_{24}O_4Na$ $[M + Na]^+$: 387.1567, found: 387.1568.

(*R$_p$*)-Methyl 4^2-((benzylamino)methyl)-1,4(1,4)-dibenzenacyclohexaphane-1^2-carboxylate (9)

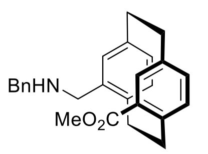

The title compound was prepared under modified reaction conditions, as previously reported.[8]
The round-bottom flask (25 ml) was charged with a magnetic stirrer and **5a** (29.4 mg, 0.1 mmol, 1.0 equiv., 99.5:0.5 *er*), followed by DCM (1.0 ml) and MeOH (1.0 ml) at room temperature. Then, benzylamine (55 µl, 0.5 mmol, 5.0 equiv.), followed by conc. HCl (25 µl, 0.2 mmol, 2.0 equiv.) were added in one portion at room temperature. At this temperature, the mixture was stirred for 30 minutes. After this time, $NaBH_3CN$ (12.6 mg, 0.2 mmol, 2.0 equiv.) was added in one portion. The reaction mixture was stirred for 2 hours, followed by benzylamine (55 µl, 0.5 mmol, 5.0 equiv.), conc. HCl (25 µl, 0.2 mmol, 2.0 equiv.), and $NaBH_3CN$ (12.6 mg, 0.2 mmol, 2.0 equiv.) addition. The reaction mixture was stirred for 2 hours at room temperature. Once the aldehyde was no longer detected (by TLC), the reaction was quenched by slowly adding an aqueous solution of NaOH (1M, 20 ml), and diluted with DCM (10 ml). The organic phase was separated. The water phase was extracted with DCM (3 × 10 ml). The collected organic phases were washed with brine (1 × 10 ml) and dried under anhydrous $MgSO_4$. After filtration of the solid, the filtrate was concentrated under reduced pressure. The crude product was purified by column chromatography (eluting by hexane/EtOAc - 1:1), affording **9** (26 mg, 66%) as a colorless oil.
Er = 99:1 (*ee* = 99%), the enantiomeric excess of product **9** was determined by HPLC using a Chiralpak® IG column (*n*-heptane/*i*-PrOH - 80:20, flow rate = 1.0 ml/min, λ = 209 nm, *t* = 25 °C): t_R = 12.4 min (minor), t_R = 13.5 min (major). $[\alpha]_D^{20}$ = -25.7 (*c* = 1.1, $CHCl_3$). ^{1}H NMR (400 MHz, chloroform-*d*): δ 7.38 – 7.22 (m, 5H), 7.19 (d, *J* = 2.0 Hz, 1H), 6.67 (dd, *J* = 7.7, 2.0 Hz, 1H), 6.59 – 6.52 (m, 2H), 6.48 (dd, *J* = 7.7, 1.9 Hz, 1H), 6.39 (d, *J* = 1.9 Hz, 1H), 4.21 – 4.10 (m, 1H), 3.78 (s, 3H), 3.75 – 3.61 (m, 3H), 3.43 – 3.34 (m, 1H), 3.29 (dd, *J* = 13.1, 0.7 Hz, 1H), 3.16 – 2.90 (m, 6H), 1.44 (br s, 1H) ppm. $^{13}C\{^1H\}$ NMR (101 MHz, chloroform-*d*): δ 167.4, 142.8, 140.49, 140.48, 139.6 (2C, *overlapped*), 138.1, 137.0, 136.4, 134.7, 134.5, 132.9, 128.7, 128.5, 128.5 (2C), 128.3 (2C), 127.0, 53.5, 51.7, 51.3, 35.0, 34.9, 34.2, 32.3 ppm. IR (ATR): ν = 3338 (N-H, amine), 1697 (C=O, ester) cm^{-1}. HRMS (ESI+) *m*/*z*: calcd. for $C_{26}H_{28}O_2$ $[M + H]^+$: 386.2115, found: 386.2113.

(*R$_p$*)-Methyl 4^2-(hydroxymethyl)-1,4(1,4)-dibenzenacyclohexaphane-1^2-carboxylate (10)

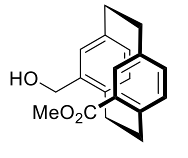

The round-bottom flask (25 ml) was charged with a magnetic stirrer and **5a** (29.4 mg, 0.1 mmol, 1.0 equiv., 99.5:0.5 *er*), followed by THF (1.0 ml) and MeOH (0.5 ml) at room temperature. Then, $NaBH_4$ (11.4 mg, 0.3 mmol, 3.0 equiv.) was added to the mixture. The reaction mixture was stirred for 1 hour at room temperature. Once the aldehyde was no longer detected (by TLC), the

reaction was quenched by slowly adding diluted hydrochloric acid (1M, 1 ml) and diluted with EtOAc (10 ml). The mixture was intensively stirred for 15 minutes. Then, the organic phase was separated. Water phase was extracted with EtOAc (3 × 10 ml). The collected organic phases were washed with brine (1 × 10 ml) and dried under anhydrous $MgSO_4$. After filtration of the solid, the filtrate was concentrated under reduced pressure. The crude product was purified by column chromatography (eluting by hexane/EtOAc - 3:1), affording **10** (24 mg, 81%) as a white amorphous.

Er = 99.5:0.5 (*ee* = 99%), the enantiomeric excess of product **10** was determined by HPLC using a Chiralpak® IC column (*n*-heptane/*i*-PrOH - 80:20, flow rate = 1.0 ml/min, λ = 232 nm, t = 25 °C): t_R = 13.6 min (major), t_R = 16.9 min (minor). $[\alpha]_D^{20}$ = -33.6 (c = 1.2, $CHCl_3$). ^1H NMR (400 MHz, chloroform-*d*): δ 7.13 (d, J = 2.0 Hz, 1H), 6.68 (dd, J = 7.8, 2.0 Hz, 1H), 6.59 – 6.54 (m, 2H), 6.52 (d, J = 1.1 Hz, 2H), 4.51 (dd, J = 14.0, 0.8 Hz, 1H), 4.28 (dd, J = 14.0, 0.8 Hz, 1H), 4.05 (ddd, J = 13.1, 10.2, 3.5 Hz, 1H), 3.90 (s, 3H), 3.41 – 3.31 (m, 1H), 3.19 – 2.93 (m, 6H), 1.97 (br s, 1H) ppm. $^{13}C\{^1H\}$ NMR (101 MHz, chloroform-*d*): δ 168.7, 142.0, 141.1, 140.0, 139.8, 137.1, 136.5, 136.3, 134.8, 134.4, 131.9, 130.3, 129.1, 63.1, 52.2, 35.0, 34.9, 33.8, 32.0 ppm. IR (ATR): ν = 3450, 3400 (O-H, alcohol), 1709 (C=O, ester) cm^{-1}. HRMS (ESI+) *m*/*z*: calcd. for $C_{19}H_{20}O_3Na$ $[M + Na]^+$: 319.1305, found: 319.1307.

(S_p)-4²-(Methoxycarbonyl)-1,4(1,4)-dibenzenacyclohexaphane-1²-carboxylic acid (11)

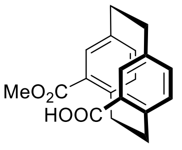

The round-bottom flask (25 ml) was charged with a magnetic stirrer and *ent*-**5a** (50 mg, 0.17 mmol, 1.0 equiv., 99:1 *er*) followed by acetone (3.5 ml), DMSO (1.4 ml), and water (2.5 ml) addition. Then, KH_2PO_4 (116 mg, 0.85 mmol, 5.0 equiv.) and $NaClO_2$ (80%, 96 mg, 0.85 mmol, 5.0 equiv.) were added in one portion at room temperature. The mixture was stirred at room temperature for 48 hours. Once the starting material was no longer detected by (TLC), the volatile solvents were concentrated under reduced pressure. The resulting mixture was diluted with water (10 ml) and extracted with EtOAc (3 × 10 ml). The collected organic phases were washed with brine (2 × 10 ml) and dried under anhydrous $MgSO_4$. After filtration of the solid, the filtrate was concentrated under reduced pressure. The crude product was purified by column chromatography (eluting by hexane/*i*-PrOH - 10:1), affording **11** (44 mg, 84%) as a white amorphous solid.

Er = 99:1 (*ee* = 98%), the enantiomeric excess of product **11** was determined by HPLC using a Chiralpak® IC column (*n*-heptane/*i*-PrOH - 40:60, flow rate = 1.0 ml/min, λ = 238 nm, t = 25 °C): t_R = 16.7 min (minor), t_R = 29.0 min (major). $[\alpha]_D^{20}$ = -8.5 (c = 1.2, MeOH). ^1H NMR (400 MHz, methanol-d_4): δ 7.13 (dd, J = 9.2, 2.0 Hz, 2H), 6.78 – 6.62 (m, 4H), 4.20 – 4.07 (m, 2H), 3.83 (s, 3H), 3.16 – 3.07 (m, 4H), 3.06 – 2.95 (m, 2H) ppm. $^{13}C\{^1H\}$ NMR (101 MHz, methanol-d_4): δ 170.3, 168.9, 143.9, 143.8, 141.1, 140.9, 137.8, 137.7, 137.23, 137.21, 135.3, 135.2, 131.9, 131.7, 52.1, 35.6, 35.6, 35.4, 35.1 ppm. IR (ATR): ν = 2931(O-H, acid), 1718, 1668 (C=O, ester, acid) cm^{-1}. HRMS (ESI+) *m*/*z*: calcd. for $C_{19}H_{18}O_4Na$ $[M + Na]^+$: 333.1097, found: 333.1101.

(S_p)-4²-Hydroxy-1,4(1,4)-dibenzenacyclohexaphane-1²-carbaldehyde (12)

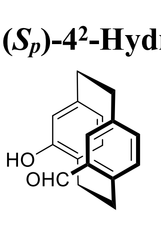

The title compound was prepared under previously reported modified reaction conditions.[9]

The round-bottom flask (100 ml) was charged with a magnetic stirrer and **5a** (500 mg, 1.7 mmol, 1.0 equiv., 99:1 *er*). The flask was evacuated and refilled with argon followed by the addition of anhydrous DCM (13 ml). *m*CBPA was dried under reduced pressure to dryness. Then, dry *m*CBPA (approx. 77%, 760 mg, 3.4 mmol, 2.0 equiv.) was added in one portion to a stirred solution of aldehyde at room temperature. The reaction was stirred overnight (15 hours) at room temperature (forming solids). Once the aldehyde was no longer detected (by TLC), the mixture was diluted with DCM (30 ml) and

washed with saturated aqueous solutions of Na_2SO_3 (1 × 20 ml), and $NaHCO_3$ (1 × 20 ml). Collected water phases were extracted with DCM (3 × 25 ml). Collected organic phases were washed with brine (1 × 25 ml) and dried under anhydrous $MgSO_4$. After filtration of the solid, the filtrate was concentrated under reduced pressure. The crude product was purified by filtration through a short pad of silica, eluting with hexane/EtOAc - 5:1. The resulting formate (282 mg, 54% yield) was used directly in a second step.
The round bottom flask (100 ml) was charged with a magnetic stirrer and formate (282 mg, 0.9 mmol, 1.0 equiv.). The flask was evacuated and refilled with argon, subsequently adding anhydrous THF (25 ml). $LiAlH_4$ (145 mg, 3.8 mmol, 4.2 equiv.) was added portionwise to a stirred solution of formate at room temperature (gas evolution). The mixture was heated up to 60 °C (oil bath) and left to stir at this temperature for 6 hours. Once the formate was no longer detected (by TLC), the mixture was cooled to room temperature and carefully quenched (gas evolution) by dropwise addition of wet EtOAc (5 ml), followed by diluted hydrochloric acid (2M approx. 15 ml) addition, yielding a homogenous mixture. The mixture was extracted with EtOAc (3 × 30 ml). Collected organic phases were washed with brine (1 × 30 ml) and dried under anhydrous $MgSO_4$. After filtration of the solid, the filtrate was concentrated under reduced pressure. The resulting alcohol (224 mg, 97% yield) was used directly to the next step without any purification.
The round-bottom flask (100 ml) was charged with a magnetic stirrer and alcohol (224 mg, 0.9 mmol, 1.0 equiv.). The flask was evacuated and refilled with argon followed by 1,4-dioxane (20 ml) addition. DDQ (200 mg, 0.9 mmol, 1.0 equiv.) was added in one portion at room temperature. At this temperature, the mixture was left to stir for 2 hours (forming solids). Once the alcohol was no longer detected (by TLC), the heterogenous mixture was filtered, and washed with DCM (2 × 5 ml). The filtrate was concentrated under reduced pressure. The crude product was purified by column chromatography (eluting by hexane/EtOAc - 4:1), affording **12** (180 mg, 81%, last step) as a white amorphous solid.
Er = 99:1 (*ee* = 97%), the enantiomeric excess of product **12** was determined by HPLC using a Chiralpak® IB column (*n*-heptane/*i*-PrOH - 80:20, flow rate = 1.0 ml/min, λ = 257 nm, t = 25 °C): t_R = 7.4 min (minor), t_R = 8.4 min (major). $[\alpha]_D^{20}$ = -339.0 (c = 0.5, $CHCl_3$). ^1H NMR (400 MHz, chloroform-*d*): δ 10.36 (s, 1H), 6.82 (dd, J = 7.8, 2.0 Hz, 1H), 6.68 (br s, 1H), 6.46 (dd, J = 7.8, 2.2 Hz, 2H), 6.33 (dd, J = 7.7, 1.7 Hz, 1H), 5.71 (d, J = 1.8 Hz, 1H), 4.05 (ddd, J = 13.3, 10.3, 4.3 Hz, 1H), 3.54 (ddd, J = 13.5, 10.3, 3.3 Hz, 1H), 3.22 – 2.91 (m, 5H), 2.75 (ddd, J = 13.3, 10.9, 4.3 Hz, 1H) ppm. $^{13}C\{^1H\}$ NMR (101 MHz, chloroform-*d*): δ 192.0, 155.0, 144.9, 141.8, 139.9, 137.8, 136.2, 135.3, 134.2, 132.2, 125.3, 124.0, 122.0, 35.0, 34.7, 30.5, 30.0 ppm. IR (ATR): ν = 3317 (O-H, phenol), 1655 (C=O, aldehyde) cm^{-1}. HRMS (ESI+) *m/z*: calcd. for $C_{17}H_{16}O_2Na$ $[M + Na]^+$: 275.1043, found: 275.1042. Our physical and spectroscopic data matched previously reported data.[9]

(S_p)-4²-Hydroxy-1,4(1,4)-dibenzenacyclohexaphane-1²-carboxylic acid (13)

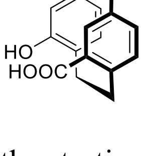

The round-bottom flask (100 ml) was charged with a magnetic stirrer and **12** (60 mg, 0.24 mmol, 1.0 equiv., *er* = 99:1) followed by acetone (10 ml), DMSO (4 ml), and water (7 ml) addition. Then, KH_2PO_4 (163 mg, 1.20 mmol, 5.0 equiv.) and $NaClO_2$ (80%, 136 mg, 1.20 mmol, 5.0 equiv.) were added in one portion at room temperature. The mixture was stirred at room temperature for 48 hours. Once the starting material was no longer detected by (TLC), the volatile solvents were concentrated under reduced pressure. The resulting mixture was diluted with brine (15 ml) and extracted with DCM (5 × 10 ml). Collected organic phases were washed with brine (2 × 15 ml) and dried under anhydrous $MgSO_4$. After filtration of the solid, the filtrate was concentrated under reduced pressure. The crude product was purified by column chromatography (eluting by hexane/*i*-PrOH - 12:1), affording **13** (44 mg, 68%) as a white amorphous solid.

Er = 99:1 (*ee* = 98%), the enantiomeric excess of product **13** was determined by HPLC using a Chiralpak® IC column (*n*-heptane/*i*-PrOH - 60:40, flow rate = 1.0 ml/min, λ = 208 nm, *t* = 25 °C): t_R = 6.4 min (minor), t_R = 12.7 min (major). $[\alpha]_D^{20}$ = +86.3 (*c* = 0.4, MeOH). ^{1}H NMR (400 MHz, methanol-d_4): δ 7.28 (d, *J* = 2.0 Hz, 1H), 6.68 (dd, *J* = 7.8, 2.0 Hz, 1H), 6.42 (dd, *J* = 16.7, 7.7 Hz, 2H), 6.19 (dd, *J* = 7.6, 1.8 Hz, 1H), 5.64 (d, *J* = 1.7 Hz, 1H), 4.27 (ddd, *J* = 12.9, 9.9, 4.2 Hz, 1H), 3.45 (ddd, *J* = 13.0, 9.9, 4.2 Hz, 1H), 3.17 – 3.01 (m, 1H), 2.92 (dddd, *J* = 21.3, 10.3, 4.5, 2.9 Hz, 4H), 2.61 (ddd, *J* = 13.0, 10.4, 4.2 Hz, 1H) ppm. ^{13}C{^{1}H} NMR (101 MHz, methanol-d_4): δ 171.4, 157.5, 144.5, 142.3, 139.9, 137.4, 136.8, 135.8, 135.6, 129.4, 126.8, 124.6, 122.0, 35.8, 35.6, 34.1, 31.0 ppm. IR (ATR): ν = 3284 (O-H, phenol), 2642 (O-H, carboxylic acid), 1664 (C=O, carboxylic acid) cm^{-1}. HRMS (ESI+) *m*/*z*: calcd. for $C_{17}H_{16}O_3Na$ [M + Na]$^+$: 291.0992, found: 291.0992.

3-Isobutyl-3,4-dihydro-2*H*-benzo[*e*][1,2,4]thiadiazine 1,1-dioxide (16)

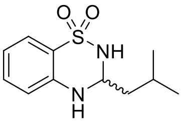

The vial (4 ml) was charged with a stirring bar, sulfonamide **14** (17.2 mg, 0.1 mmol, 1.0 equiv.), molecular sieves (4 Å, 15 mg), and acid **13** (2.7 mg, 0.01 mmol, 0.1 equiv.) followed by anhydrous toluene (0.5 ml) addition. Then, isovaleraldehyde (12 µl, 0.11 mmol, 1.1 equiv.). The reaction was stirred for 24 hours at room temperature. Once the reaction was completed (as shown by TLC), sieves were filtered, and the solvent was evaporated. The crude product was purified by column chromatography (eluting by hexane/EtOAc- 3:1), affording **16** (16 mg, 67%) as a white amorphous solid.

Er = 53:47 (*ee* = 5%), the enantiomeric excess of product **16** was determined by HPLC using a Chiralpak® ODH column (*n*-heptane/*i*-PrOH - 80:20, flow rate = 1.0 ml/min, λ = 249 nm, *t* = 25 °C): t_R = 7.0 min (minor), t_R = 14.8 min (major). ^{1}H NMR (400 MHz, chloroform-*d*): δ 7.64 (dd, *J* = 7.9, 1.5 Hz, 1H), 7.32 – 7.26 (m, 1H), 6.85 (ddd, *J* = 8.2, 7.2, 1.1 Hz, 1H), 6.67 (dd, *J* = 8.3, 1.1 Hz, 1H), 4.99 (t, *J* = 6.8 Hz, 1H), 4.42 (s, 1H), 4.29 (br s, 1H), 1.91 (ddt, *J* = 13.2, 7.9, 6.6 Hz, 1H), 1.73 (ddd, *J* = 14.1, 7.7, 6.5 Hz, 1H), 1.59 (ddd, *J* = 13.6, 7.8, 5.7 Hz, 1H), 1.02 (d, *J* = 3.0 Hz, 3H), 1.00 (d, *J* = 3.1 Hz, 3H) ppm. ^{13}C{^{1}H} NMR (101 MHz, chloroform-*d*): δ 142.6, 133.4, 125.0, 122.9, 119.1, 116.3, 65.0, 43.9, 24.3, 22.7, 22.5 ppm. IR (KBr): ν = 1298, 1153 (S=O, sulfonamide) cm^{-1}. HRMS (ESI+) *m*/*z*: calcd. for $C_{11}H_{17}N_2O_2S$ [M + H]$^+$: 241.1005, found: 241.1003. Our physical and spectroscopic data matched previously reported data.[10]

2-Nitro-1-phenylethan-1-ol (19)

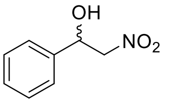

The vial (4 ml) was charged with stirring bar, *p*-nitrobenzaldehyde (15.1 mg, 0.1 mmol, 1.0 equiv.), and acid **13** (2.7 mg, 0.01 mmol, 0.1 equiv.), followed by anhydrous THF (0.5 ml), DIPEA (3.5 µl, 0.02 mmol, 0.2 equiv.), and nitromethane (54 µl, 1.0 mmol, 10.0 equiv.) addition. The reaction was stirred for 48 hours at room temperature. Once the reaction was completed (as shown by TLC), the mixture was diluted with EtOAc (10 ml) and washed by saturated solution of NH_4Cl (1 × 5 ml). The organic phase was dried under anhydrous $MgSO_4$. After filtration of the solid, the filtrate was concentrated under reduced pressure. The crude product was purified by column chromatography (eluting by hexane/EtOAc - 3:1), affording **19** (17 mg, 95%) as a colorles oil.

Er = 51:49 (*ee* = 3%), the enantiomeric excess of product **19** was determined by HPLC using a Chiralpak® ODH column (*n*-heptane/*i*-PrOH - 80:20, flow rate = 1.0 ml/min, λ = 212 nm, *t* = 25 °C): t_R = 10.0 min (major), t_R = 12.0 min (minor). ^{1}H NMR (400 MHz, chloroform-*d*): δ 8.27 (d, *J* = 8.7 Hz, 2H), 7.71 – 7.55 (m, 2H), 5.61 (dt, *J* = 8.3, 4.1 Hz, 1H), 4.65 – 4.52 (m, 2H), 3.14 (d, *J* = 4.1 Hz, 1H) ppm. ^{13}C{^{1}H} NMR (101 MHz, chloroform-*d*): δ 148.3, 145.0, 127.1 (2C), 124.4 (2C), 80.7, 70.1 ppm. IR (ATR): ν = 3514 (O-H, alcohol), 1514, 1342 (N-O, nitro)

cm^{-1}. HRMS (ESI-) *m/z*: calcd. for $C_7H_4NO_4$ $[M - H]^-$: 166.0146, found: 166.0146. Our physical and spectroscopic data matched previously reported data.[11]

Mechanistic studies

a) for pseudo-para derivative

Based on our mechanistic experimental study, we proposed a catalytic cycle together with the proposed rate-determining and stereo-divergent steps (Supplementary Fig. 2). At the beginning, carbene **I** is formed in situ by base deprotonation of the corresponding azolium salt (*pre*-**C**). Then, the nucleophilic carbene attacks (process A) the aldehyde carbon, yielding a tetrahedral intermediate **II**. This intermediate undergoes a 1,2-hydride shift (process B), generating a Breslow intermediate (**III**). In the presence of an oxidant (DQ), the Breslow intermediate can be oxidized (process B) into an acyl azolium intermediate (**IV**). The acyl azolium is electrophilic at the carbonyl carbon and thus undergoes acyl substitutions (process D) with alcohol **2** (or alkoxide). These acyl substitutions regenerate the carbene back to catalytic cycle, yielding ester **3**.

The deuterium-labeling experiment showed significant deuteration of aldehyde in the model reaction conducted with deuterated methanol, which indicated the reversibility of the formation of Breslow intermediate. Moreover, a parallel study of the kinetic isotope effect showed *KIE* = 2.8, revealing a normal isotope effect,[12] which is consistent with values previously observed in various NHC-catalyzed processes, such us intramolecular Stetter reaction.[13,14] Based on these observations, we suggested proton transfer as the turnover-limiting step (process B). To elaborate on the origin of asymmetric induction, we performed the desymmetrization reaction **1a** with methanol under optimized reaction conditions with a lowered amount of oxidant, which produced the expected product **3a** with a lower level of optical purity (88.3/11.7 *er*). This result indicates that desymmetrization is one of the enantio-divergent steps. We hypothesized, that desymmetrization is followed by kinetic resolution, which was confirmed by the formation of highly enantioenriched product **3a** in the kinetic resolution reaction of *rac*-**3a**. Therefore, we proposed desymmetrization and kinetic resolution as enantiodivergent steps.

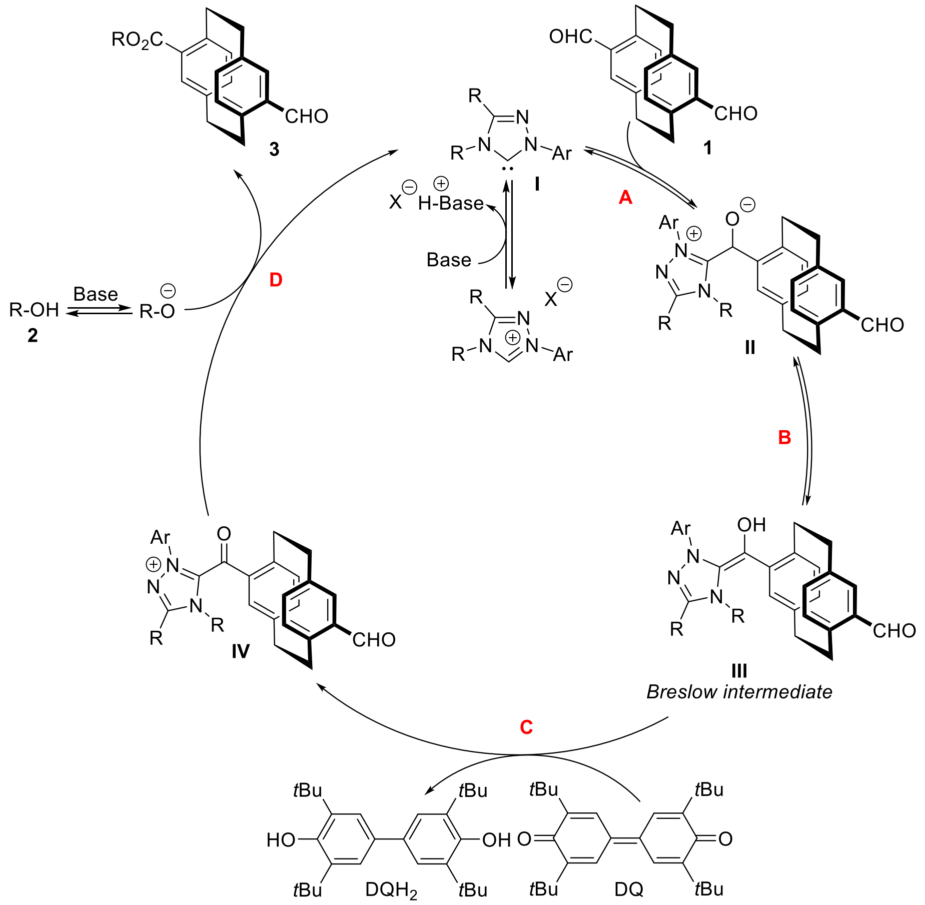

Supplementary Fig. 2. Proposed reaction mechanism for enantioselective desymmetrization of *pseudo-para* derivative (**1a**).

To rationalize the stereochemical outcome observed in the desymmetrization reaction, we proposed putative transition states (Supplementary Fig. 3). The main enantiodiscrimination step is determined by steric hindrance of the chiral catalyst during an initial nucleophilic attack to the aldehyde group of starting material **1a**. Steric hindrance of the isopropyl group from the catalyst effectively shields one face of the catalyst. The less-shielded face is non-planar due to the presence of one of the methyl groups, which sterically disfavors the formation of one enantiomer, in both processes (desymmetrization and kinetic resolution).

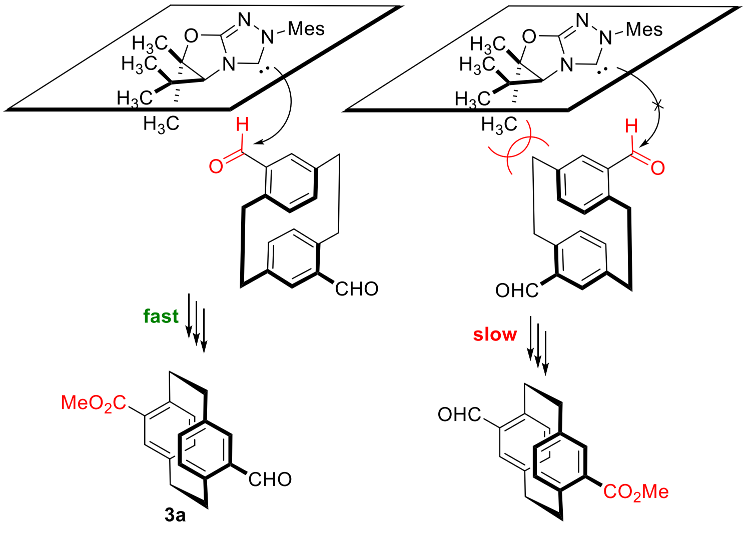

Supplementary Fig. 3. Plausible transition states of enantiodiscrimination step for **1a**.

b) for pseudo-gem derivative

In the desymmetrization process of the *pseudo-gem* derivative, differences were found in both the rate-determining and the stereo-divergent steps (Supplementary Fig. 4). For example, the Breslow intermediate was irreversibly formed under optimal reaction conditions (according to deuterium labeling experiment). In addition, we observed the inverse KIE, where $k_H/k_D < 1$ was interpreted as arising from an increase in the aldehydic C−H(D) force constant of out-of-plane bending from the ground state (sp^2) to the zwitterionic intermediate (sp^3).[15] In this process, asymmetric induction was straightforward, with an excellent level of enantiomeric purity of product **5a** in the desymmetrization reaction performed with a lower amount of oxidant. Furthermore, kinetic resolution of *rac*-**5a** was almost ineffective.

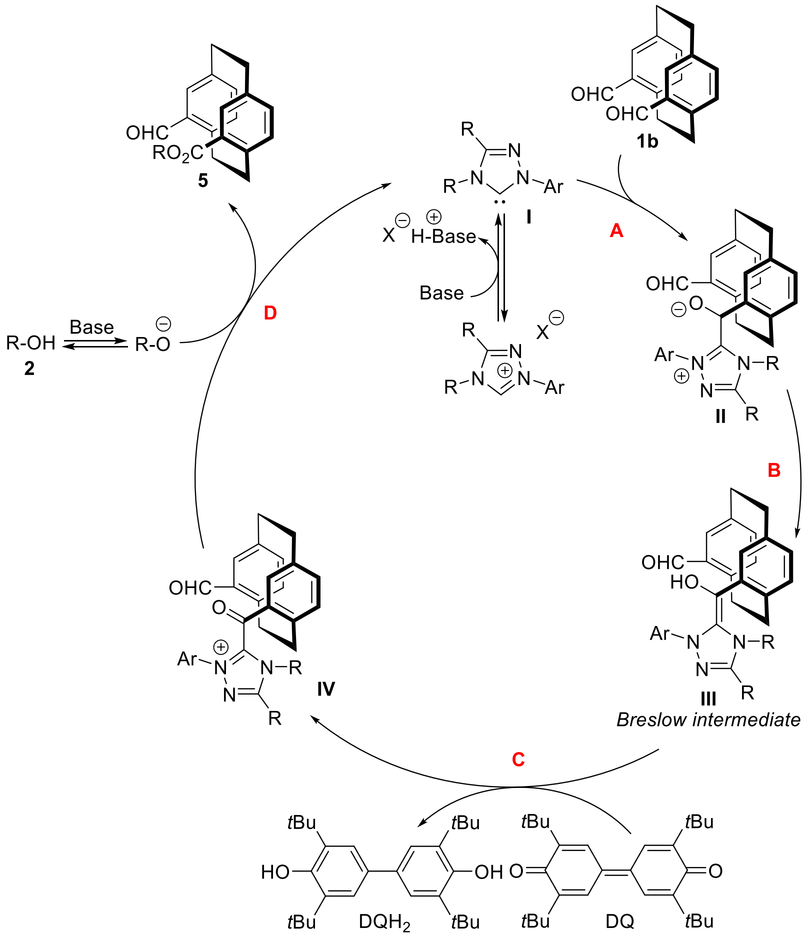

Supplementary Fig. 4. Proposed reaction mechanism for enantioselective desymmetrization of *pseudo-gem* derivative (**1b**).

As in the desymmetrization process for the *pseudo-para* derivative (**1a**), we proposed putative transition states for the *pseudo-gem* derivative (Supplementary Fig. 5). The main enantiodiscrimination step is determined by steric hindrance of the chiral catalyst during an initial nucleophilic attack to the aldehyde group of starting material **1b**. Steric hindrance of the isopropyl group from the catalyst effectively shields one face of the catalyst. The less-shielded face is non-planar due to the presence of one of the methyl groups, which sterically disfavors the formation of one enantiomer, especially in the desymmetrization reaction.

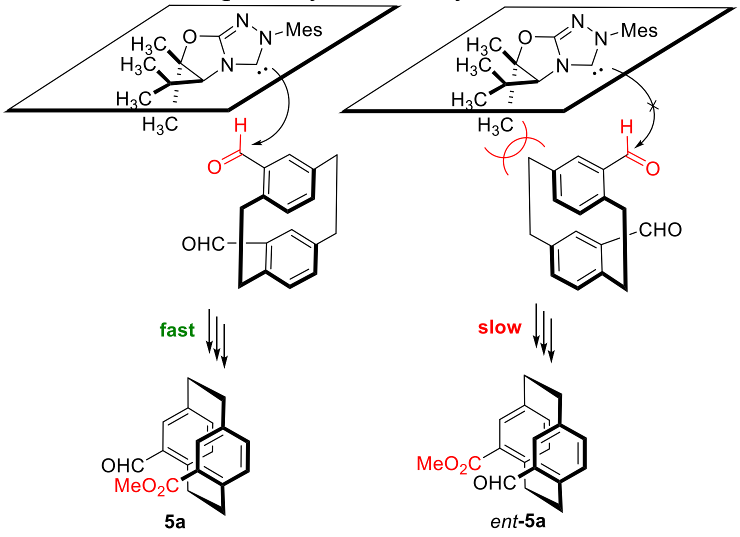

Supplementary Fig. 5. Plausible transition states of enantiodiscrimination step for **1b**.

Deuterium labeling experiments

a) for pseudo-para derivative

The vial (4 ml) was charged with **1a** (26.4 mg, 0.1 mmol, 1.0 equiv.), *pre*-**C1** (7.6 mg, 0.02 mmol, 0.2 equiv.), DQ (49.0 mg, 0.12 mmol, 1.2 equiv.), and Cs_2CO_3 (65.2 mg, 0.2 mmol, 2.0 equiv.), followed by DCM (1.0 ml), MeOD-d_4 (20 µl, 0.5 mmol, 5.0 equiv.) at room temperature. The reaction was stirred for 15 hours at room temperature. Once the reaction was completed (as shown by TLC), the solvent was evaporated. The crude product was purified by column chromatography (eluting by hexane/EtOAc - 9:1), affording **3a**-d_3 (19 mg, 64%) as a white amorphous solid.

The product **3a**-d_3 showed deuterium incorporation to aldehyde with 39% deuterium incorporation (Supplementary Fig. 6), incorporation was confirmed by ^{2}H NMR (Supplementary Fig. 7).

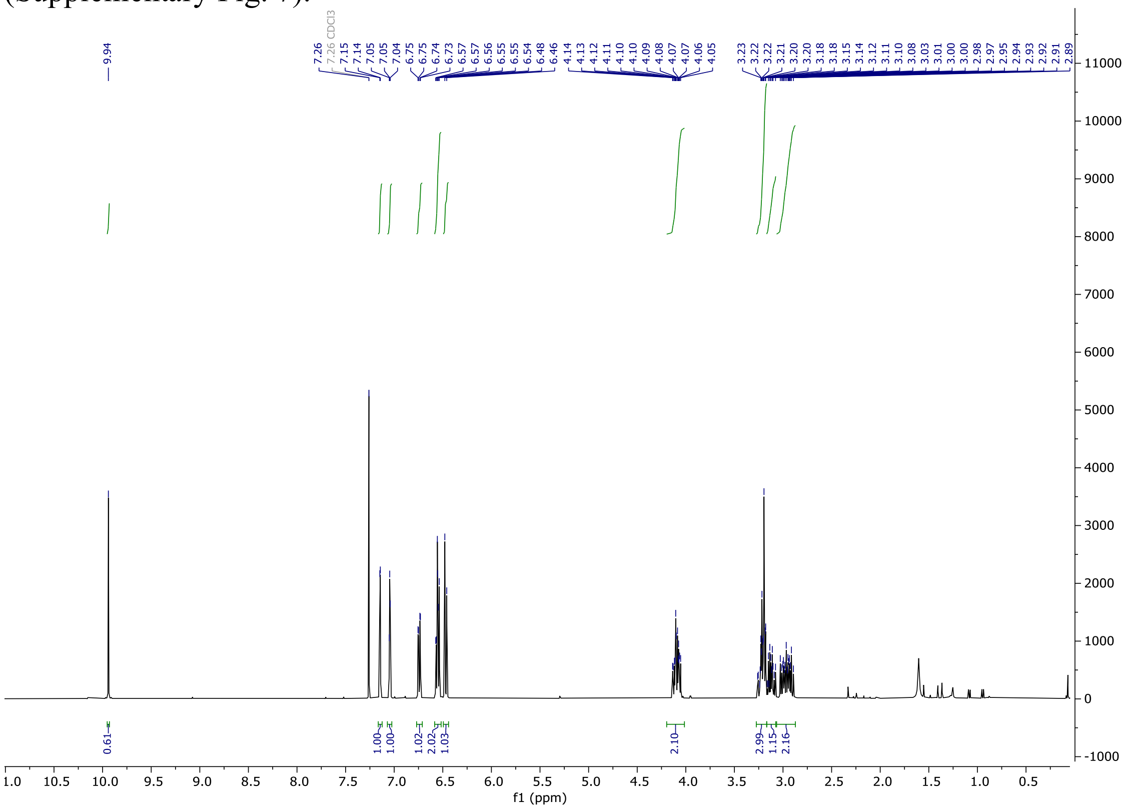

Supplementary Fig. 6. ^{1}H NMR of **3a**-d_3 (400 MHz, $CDCl_3$)

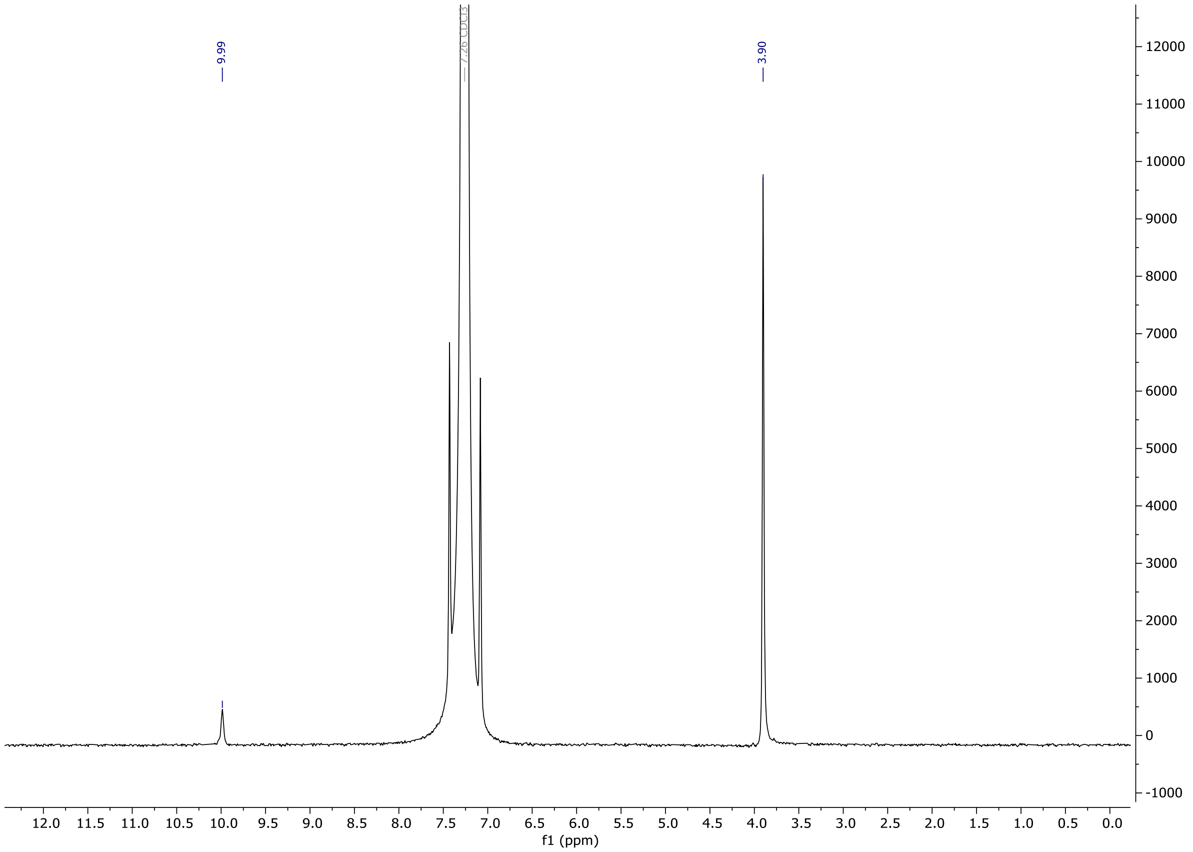

Supplementary Fig. 7. ^{2}H NMR of **3a**-*d*$_3$ (92 MHz, $CDCl_3$)

a) for pseudo-gem derivative

The vial (4 ml) was charged with **1b** (26.4 mg, 0.1 mmol, 1.0 equiv.), *pre*-**C1** (7.6 mg, 0.02 mmol, 0.2 equiv.), DQ (49.0 mg, 0.12 mmol, 1.2 equiv.), and Cs_2CO_3 (65.2 mg, 0.2 mmol, 2.0 equiv.), followed by precooled DCM (0°C,1.0 ml), MeOD-*d*$_4$ (20 µl, 0.5 mmol, 5.0 equiv.) addition at room temperature. The reaction was stirred for 15 hours at 0 °C (crycooler). Once the reaction was completed (as shown by TLC), the solvent was evaporated. The crude product was purified by column chromatography (eluting by hexane/EtOAc – 8:1), affording **5a**-*d*$_3$ (24 mg, 81%) as a white amorphous solid. The product **5a**-*d*$_3$ did not show deuterated aldehyde (Supplementary Fig. 8), as confirmed by ^{2}H NMR (Supplementary Fig. 9).

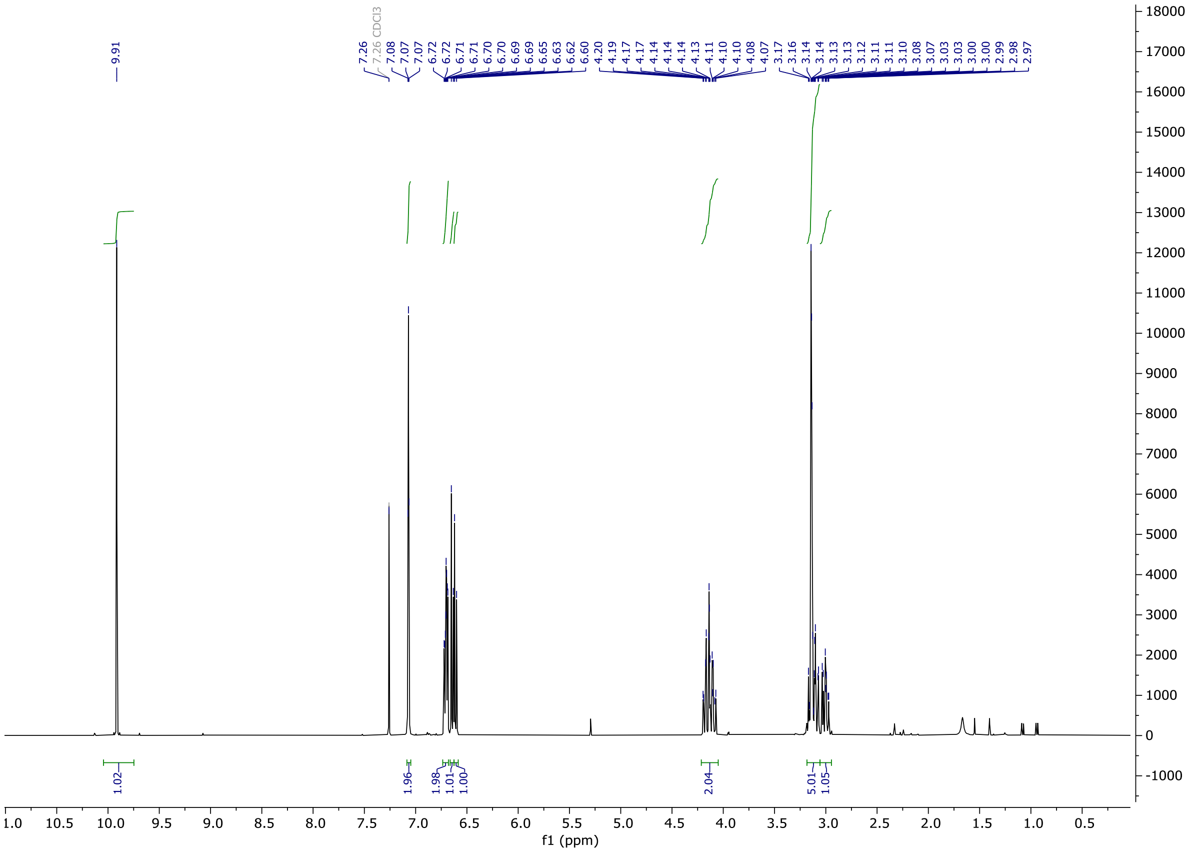

Supplementary Fig. 8. ^{1}H NMR of **5a**-*d*$_3$ (400 MHz, $CDCl_3$)

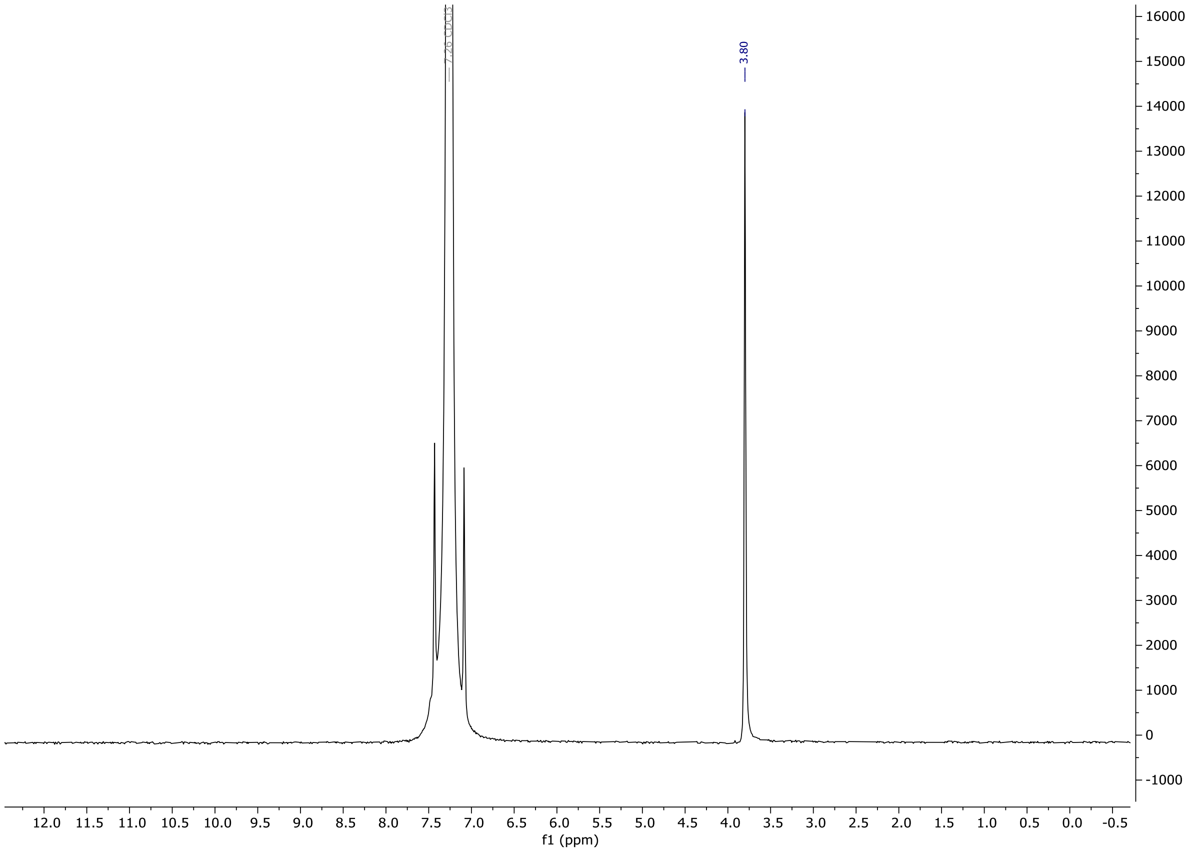

Supplementary Fig. 9. ^{2}H NMR of **5a**-*d*$_3$ (92 MHz, $CDCl_3$)

Parallel kinetic isotope effect

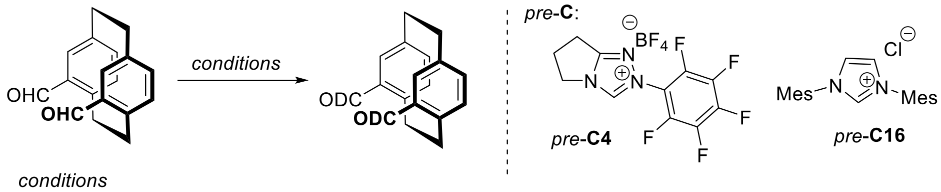

conditions

D_2O, *pre*-**C16** (20 mol%), Na_2CO_3, toluene, reflux: 26%D (low solubility of paracyclophane), not isolated

D_2O, *pre*-**C16** (20 mol%), K_2CO_3, DCM, 40 °C: 27%D, not isolated

D_2O, *pre*-**C4** (20 mol%), K_2CO_3, DCM, 40 °C: 98%D, 60% yield

Supplementary Fig. 10. Optimization of reaction conditions for deuteration

General procedure for deuteration of diformyl derivatives

The round-bottom flask (25 ml) was charged with a magnetic stirrer and the corresponding diformyl derivative **1** (200 mg, 0.76 mmol, 1.0 equiv.) and *pre*-**C4** (55 mg, 0.15 mmol, 0.2 equiv.), followed by DCM (1 ml) and D_2O (4 ml) addition. Then, K_2CO_3 (105 mg, 0.76 mmol, 1.0 equiv.) was added to a stirred biphasic solution. The resulting reaction mixture was heated up to 40 °C (oil bath) and stirred for 24 hours at this temperature. After this time, the reaction mixture was cooled to room temperature and diluted with DCM (20 ml). The organic phase was separated, and the water phase was extracted with DCM (3 × 20 ml). After filtration of the solid, the filtrate was concentrated under reduced pressure. The crude product was purified by column chromatography.

1,4(1,4)-Dibenzenacyclohexaphane-1^2,4^3-dicarbaldehyde-d_2 (1a-d_2)

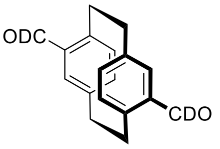

The title compound was synthesized according to the general procedure. The product was purified by column chromatography (eluting by DCM/acetone - 25:1), affording **1a**-d_2 (153 mg, 77%) as a white amorphous solid.

96% Deuterium incorporation. ^{1}H NMR (400 MHz, chloroform-*d*): δ 9.94 (s, 0.07H), 7.05 (d, J = 2.0 Hz, 2H), 6.63 (dd, J = 7.8, 2.0 Hz, 2H), 6.52 (d, J = 7.8 Hz, 2H), 4.13 (ddd, J = 13.0, 10.3, 2.4 Hz, 2H), 3.29 (ddd, J = 13.2, 10.5, 2.4 Hz, 2H), 3.16 (ddd, J = 13.3, 10.3, 5.8 Hz, 2H), 3.02 (ddd, J = 13.2, 10.6, 5.8 Hz, 2H) ppm. ^{2}H NMR (92 MHz, chloroform-*d*): δ 9.97 (s) ppm. ^{13}C{^{1}H} NMR (101 MHz, chloroform-*d*): δ 191.7 (t, J = 26.5 Hz, 2C), 143.1 (2C), 140.7 (2C), 137.1 (2C), 137.0-136.8 (m, 2C), 136.6 (2C), 135.4 (2C), 34.5 (2C), 32.9 (2C) ppm. IR (ATR): ν = 1653 (C=O, aldehyde) cm^{-1}. HRMS (ESI+) *m*/*z*: calcd. for $C_{18}H_{14}D_2NaO_3$ [M + Na]$^+$: 289.1168, found: 289.1167.

1,4(1,4)-Dibenzenacyclohexaphane-1^2,4^2-dicarbaldehyde-d_2 (1b-d_2)

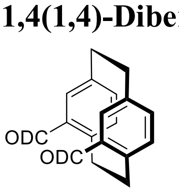

The title compound was synthesized according to the general procedure. The product was purified by column chromatography (eluting by hexane/EtOAc - 5:1), affording **1a**-d_2 (120 mg, 60%) as a white amorphous solid.

98% Deuterium incorporation. ^{1}H NMR (400 MHz, chloroform-*d*): δ 9.80 (s, 0.04H), 6.99 (d, J = 2.0 Hz, 2H), 6.75 (dd, J = 7.8, 2.0 Hz, 2H), 6.66 (d, J = 7.8 Hz, 2H), 4.17 – 4.08 (m, 2H), 3.22 – 3.07 (m, 6H) ppm. ^{2}H NMR (92 MHz, chloroform-*d*): δ 9.84 (s) ppm. ^{13}C{^{1}H} NMR (101 MHz, chloroform-*d*): δ 191.7 (t, J = 26.6 Hz, 2C), 142.9 (2C), 140.6 (2C), 138.1 (2C), 137.2-136.9 (m, 2C), 136.2 (2C), 134.7 (2C), 34.9 (2C), 32.1 (2C) ppm. IR (ATR): ν = 1655 (C=O, aldehyde) cm^{-1}. HRMS (ESI+) *m*/*z*: calcd. for $C_{18}H_{14}D_2NaO_3$ [M + Na]$^+$: 289.1168, found: 289.1167.

Experimental procedure for parallel kinetic isotope effect experiments

a) for pseudo-para diformyl derivative

The vial (4 ml) was charged with **1a** (26.4 mg, 0.1 mmol, 1.0 equiv.), *pre*-**C1** (7.6 mg, 0.02 mmol, 0.2 equiv.), DQ (49.0 mg, 0.12 mmol, 1.2 equiv.), and Cs_2CO_3 (65.2 mg, 0.2 mmol, 2.0 equiv.), followed by DCM (1.0 ml) and methanol (20 µl, 0.5 mmol, 5 equiv.) addition at room temperature. The reaction was left to stir for 1 hour. Then, the mixture was filtered through a short pad of Celite and washed with DCM (1 ml) before evaporating the solvents. The reaction mixture was dissolved in $CDCl_3$ (0.6 ml) and dibromomethane (7.0 µl, 0.1 mmol) and analyzed by ^1H NMR. The NMR yield of **3a** was 41% (Supplementary Fig. 11).

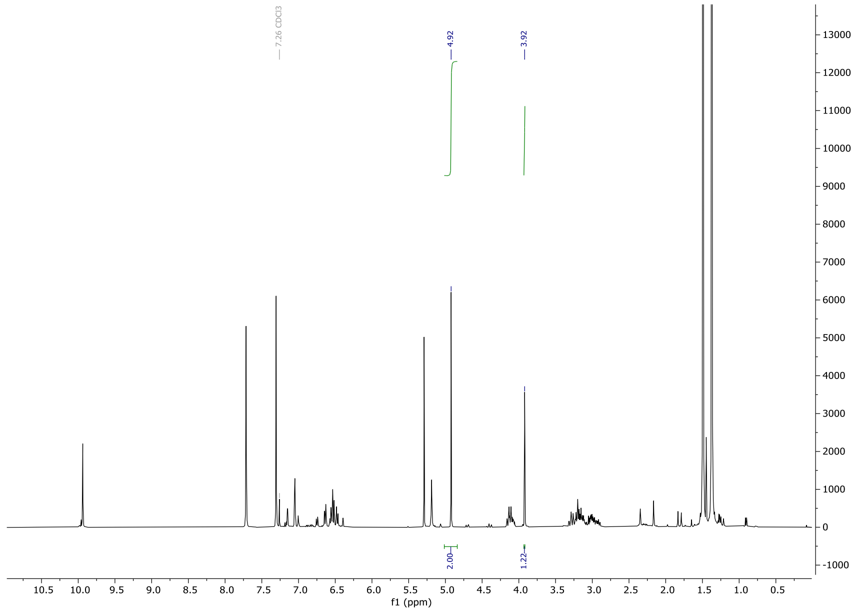

Supplementary Fig. 11. NMR yield determination for **3a**

The vial (4 ml) was charged with **1a**-d_2 (26.6 mg, 0.1 mmol, 1.0 equiv.), *pre*-**C1** (7.6 mg, 0.02 mmol, 0.2 equiv.), DQ (49.0 mg, 0.12 mmol, 1.2 equiv.), and Cs_2CO_3 (65.2 mg, 0.2 mmol, 2.0 equiv.), followed by addition DCM (1.0 ml), and methanol (20 µl, 0.5 mmol, 5 equiv.) at room temperature. Reaction was left to stir for 1 hour. Then, mixture was filtered through a short pad of Celite and washed with DCM (1 ml) before evaporating the solvents. The reaction mixture was dissolved in $CDCl_3$ (0.6 ml) and dibromomethane (7.0 µl, 0.1 mmol) and analyzed by ^1H NMR. The NMR yield of **3a**-d_1 was 15% (Supplementary Fig. 12).

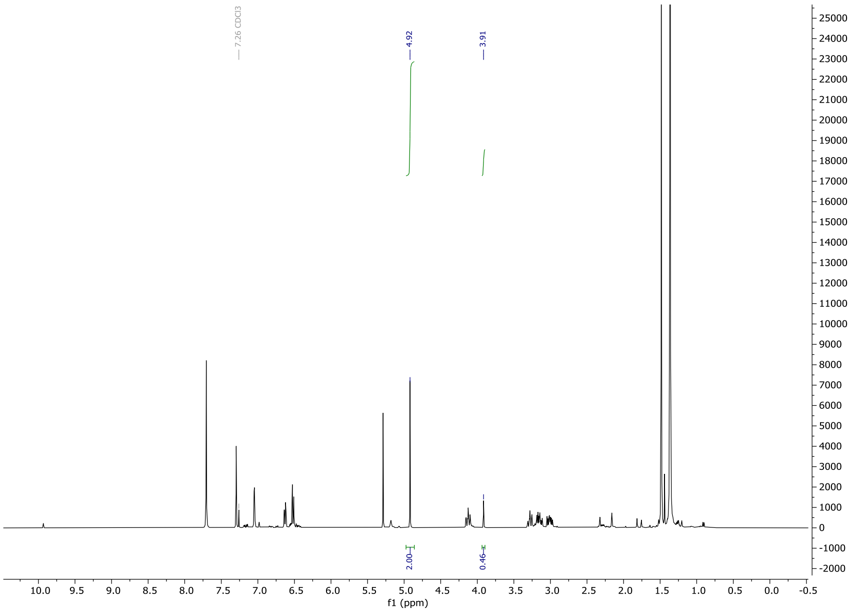

Supplementary Fig. 12. NMR yield determination for **3a-**d_1

Supplementary equation

$$KIE = \frac{k_{\mathrm{H}}}{k_{\mathrm{D}}} = \frac{NMR\ yield_{\mathrm{H}}}{NMR\ yield_{\mathrm{D}}} \qquad (1)$$

where k_{H} and k_{D} represent the rate constants of non-labeled reaction and deuterium-labeled reaction

$$KIE = \frac{41}{15} = 2.73 \qquad (2)$$

b) for pseudo-gem derivative

The vial (4 ml) was charged with **1b** (26.4 mg, 0.1 mmol, 1.0 equiv.), *pre*-**C1** (7.6 mg, 0.02 mmol, 0.2 equiv.), DQ (49.0 mg, 0.12 mmol, 1.2 equiv.), and Cs_2CO_3 (65.2 mg, 0.2 mmol, 2.0 equiv.), followed by precooled DCM (0°C, 1.0 ml) and methanol (20 µl, 0.5 mmol, 5 equiv.) addition at 0 °C (cryocooler). The reaction was stirred for 1 hour at this temperature. Then, the mixture was filtered through a short pad of Celite, washed with DCM (1 ml) and solvents were evaporated. The reaction mixture was dissolved in $CDCl_3$ (0.6 ml) and dibromomethane (7.0 µl, 0.1 mmol) and analyzed by ^{1}H NMR. The NMR yield of **5a** was 9% (
Supplementary Fig. **13**).

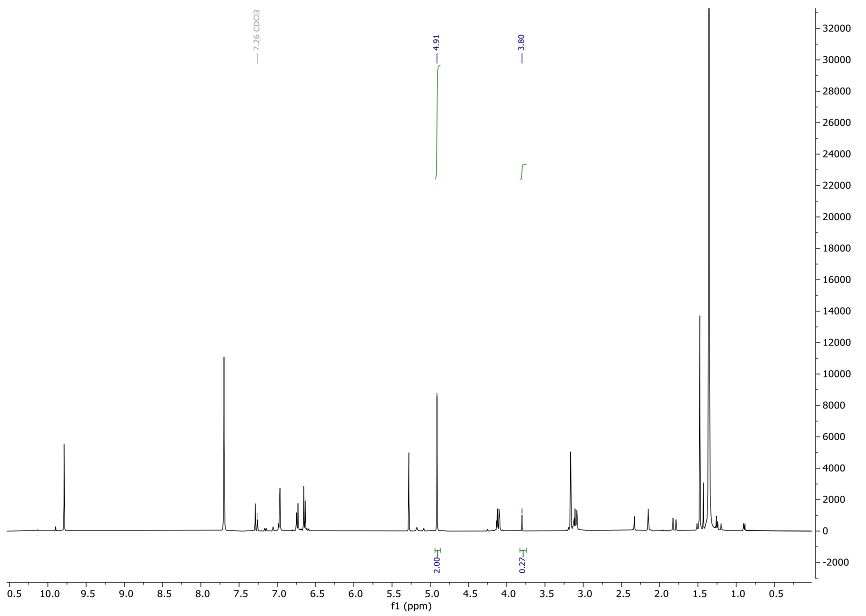

Supplementary Fig. 13. NMR yield determination for **5a**

The vial (4 ml) was charged with **1b-*d*$_2$** (26.6 mg, 0.1 mmol, 1.0 equiv.), *pre*-**C1** (7.6 mg, 0.02 mmol, 0.2 equiv.), DQ (49.0 mg, 0.12 mmol, 1.2 equiv.), and Cs_2CO_3 (65.2 mg, 0.2 mmol, 2.0 equiv.), followed by precooled DCM (0°C, 1.0 ml) and methanol (20 µl, 0.5 mmol, 5 equiv.) addition at 0 °C (cryocooler). Reaction was left to stir for 1 hour at this temperature. Then, mixture was filtered through short pad of Celite, washed with DCM (1 ml) and solvents were evaporated. Reaction mixture was dissolved in $CDCl_3$ (0.6 ml) and dibromomethane (7.0 µl, 0.1 mmol) and analyzed by ^1H NMR. The NMR yield of **5a-*d*$_1$** was 20% (Supplementary Fig. 14).

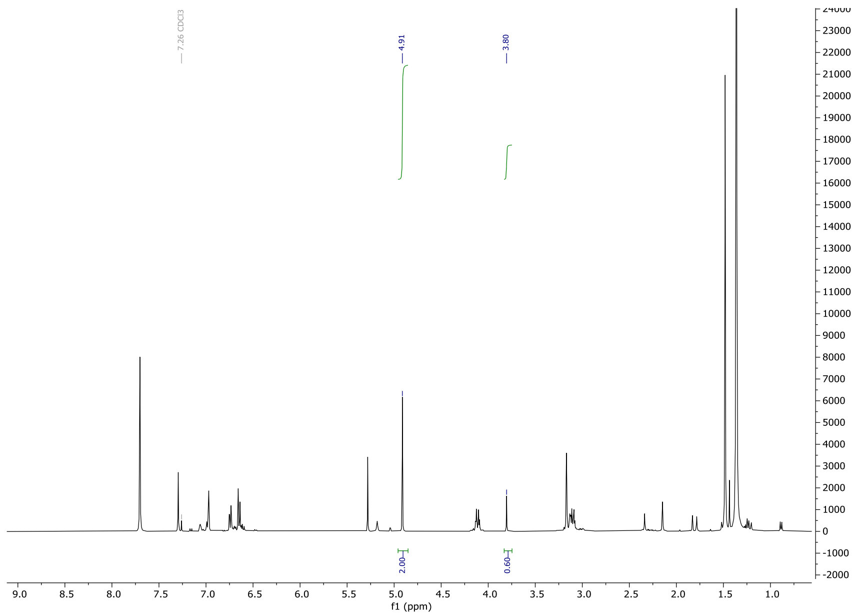

Supplementary Fig. 14. NMR yield determination for **5a-*d*$_1$**.

Supplementary equation

$$KIE = \frac{9}{20} = 0.45 \qquad (3)$$

Stereocontrol of the process

Desymmetrization

a) for pseudo-para derivative

The vial (4 ml) was charged with **1a** (26.4 mg, 0.1 mmol, 1.0 equiv.), *pre*-**C1** (7.6 mg, 0.02 mmol, 0.2 equiv.), DQ (22.5 mg, 0.055 mmol, 0.55 equiv.), and Cs_2CO_3 (65.2 mg, 0.2 mmol, 2.0 equiv.), and evacuated and refilled with argon. Then, DCM (1.0 ml), and MeOH (20 µl, 0.5 mmol, 5.0 equiv.) were added at room temperature. The reaction was stirred for 15 hours at room temperature under argon atmosphere. After that time, the solvent was evaporated. The crude product was purified by column chromatography (eluting with hexane/EtOAc – 9:1), affording **3a** (10 mg, 35%) as a white amorphous solid.

Er = 88.3/11.7, the enantiomeric excess of product **3a** was determined by HPLC using a Chiralpak® IB column (*n*-heptane/*i*-PrOH - 80:20, flow rate = 1.0 ml/min, λ = 190 nm, t = 25 °C): t_R = 7.8 min (major), t_R = 10.3 min (minor).

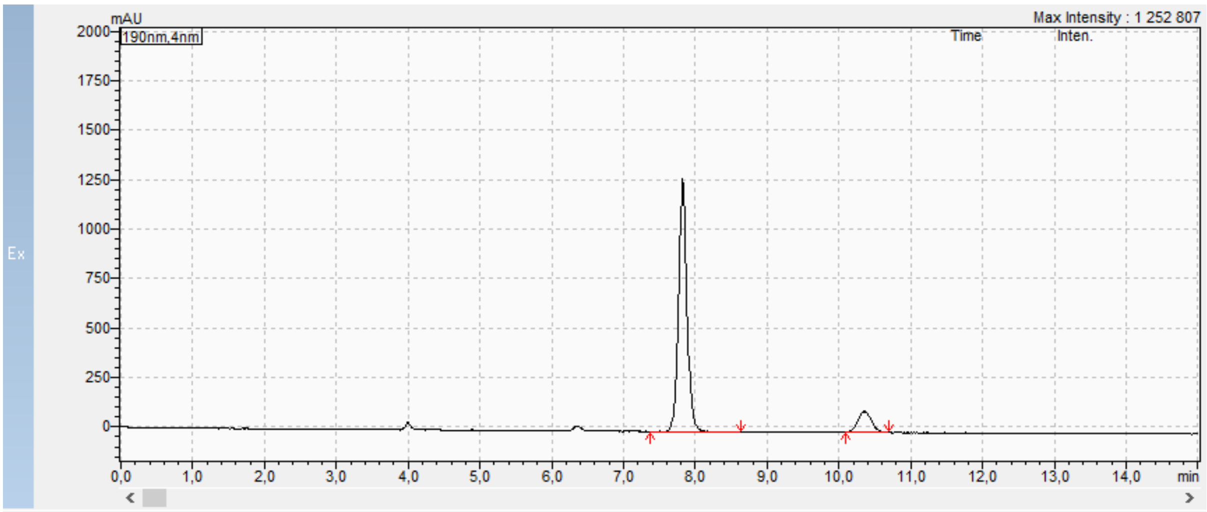

Results View - Peak Table

Peak Table | Compound | Group | Calibration Curve

Peak#	Ret. Time	Area	Height	Peak Start	Peak End	Mark	Conc.	Unit	Area%
1	7,821	10382832	1276159	7,360	8,640	M	88,308		88,308
2	10,355	1374673	105506	10,091	10,699	M	11,692		11,692
Total		11757505	1381666				100,000		100,000

Supplementary Fig. 15. Chiral HPLC trace of **3a** obtained in the desymmetrization control experiment.

Supplementary equation
Desymmetrization rate

$$k_R/k_S = \frac{R}{S} \tag{4}$$

where *R* and *S* represent the amount of obtained enantiomers (%)

$$k_R/k_S = \frac{88.31}{11.69} = \frac{7.55}{1} \tag{5}$$

b) for pseudo-gem derivative

The vial (4 ml) was charged with **1b** (26.4 mg, 0.1 mmol, 1.0 equiv.), *pre*-**C1** (7.6 mg, 0.02 mmol, 0.2 equiv.), DQ (22.5 mg, 0.055 mmol, 0.55 equiv.), and Cs_2CO_3 (65.2 mg, 0.2 mmol, 2.0 equiv.), and evacuated and refilled with argon. Then, precooled DCM (0°C, 1.0 ml), MeOH (20 µl, 0.5 mmol, 5.0 equiv.) were added. The reaction was stirred for 15 hours at 0 °C (crycooler). After that time, the solvent was evaporated. The crude product was purified by

column chromatography (eluting with hexane/EtOAc – 8:1), affording **5a** (16 mg, 53%) as a white amorphous solid.
Er = 99.8/0.2, the enantiomeric excess of product **5a** was determined by HPLC using a Chiralpak® IB column (*n*-heptane/*i*-PrOH - 80:20, flow rate = 1.0 ml/min, λ = 190 nm, t = 25 °C): t_R = 9.3 min (major), t_R = 14.3 min (minor).

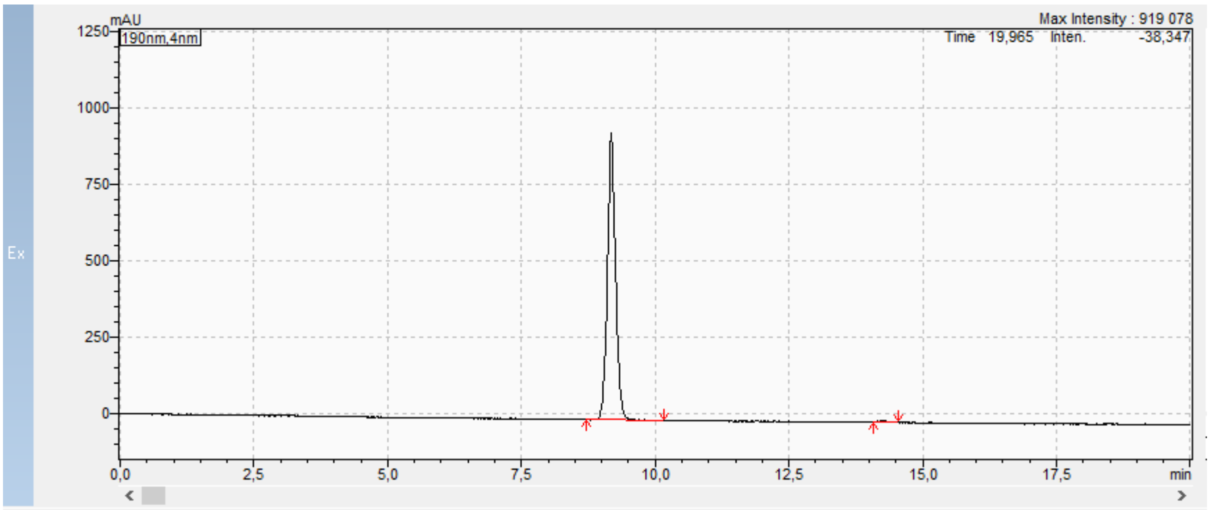

Peak#	Ret. Time	Area	Height	Peak Start	Peak End	Mark	Conc.	Unit	Area%
1	9,177	9892986	940226	8,715	10,155	M	99,773		99,773
2	14,236	22539	2994	14,069	14,539	M	0,227		0,227
Total		9915525	943219				100,000		100,000

Supplementary Fig. 16. Chiral HPLC trace of **5a** obtained in the desymmetrization control experiment.

Supplementary equation

Desymmetrization rate

$$k_R/k_S = \frac{99.77}{0.23} = \frac{433.8}{1} \quad (6)$$

Kinetic resolution

a) for pseudo-para derivative

The vial (4 ml) was charged with *rac*-**3a** (29.4 mg, 0.1 mmol, 1.0 equiv.), *pre*-**C1** (7.6 mg, 0.02 mmol, 0.2 equiv.), DQ (22.5 mg, 0.055 mmol, 0.55 equiv.), and Cs_2CO_3 (65.2 mg, 0.2 mmol, 2.0 equiv.), and evacuated and refilled with argon. Then, DCM (1.0 ml), and MeOH (20 µl, 0.5 mmol, 5.0 equiv.) were added at room temperature. The reaction was stirred for 15 hours at room temperature under argon atmosphere. After that time, the solvent was evaporated. The crude product was purified by column chromatography (eluting by hexane/EtOAc – 9:1), , affording **3a** (10 mg, 35%) as a white amorphous solid and **4a** (16 mg, 50%) as a white amorphous solid.
Er = 84.4/15.6, the enantiomeric excess of product **3a** was determined by HPLC using a Chiralpak® IB column (*n*-heptane/*i*-PrOH - 80:20, flow rate = 1.0 ml/min, λ = 190 nm, t = 25 °C): t_R = 7.8 min (major), t_R = 10.3 min (minor).

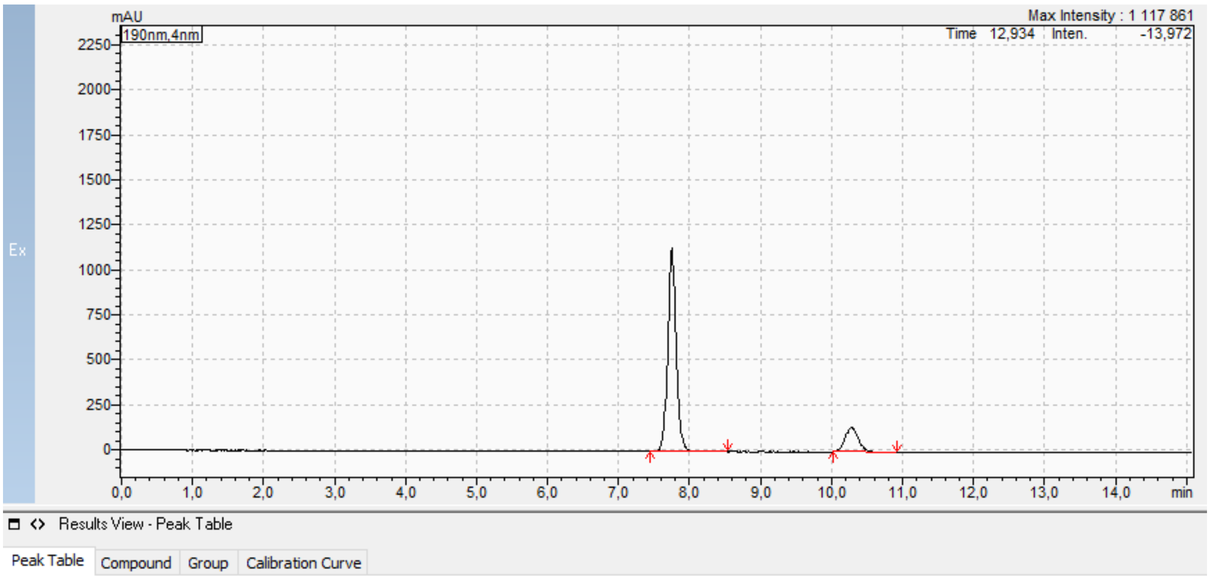

Peak#	Ret. Time	Area	Height	Peak Start	Peak End	Mark	Conc.	Unit	Area%
1	7,752	9268984	1126804	7,435	8,533	M	84,377		84,377
2	10,274	1716193	132778	10,027	10,912	M	15,623		15,623
Total		10985177	1259581				100,000		100,000

Supplementary Fig. 17. Chiral HPLC trace of **3a** obtained in the kinetic resolution control experiment.

Supplementary equations
Resolution rate

$$V_{\mathrm{R}}/V_{\mathrm{S}} = \frac{R}{S} \qquad (7)$$

where *R* and *S* represent the amount of obtained enantiomers (%)

$$V_{\mathrm{R}}/V_{\mathrm{S}} = \frac{84.38}{15.62} = \frac{5.40}{1} \qquad (8)$$

Selectivity factor

$$s = \frac{\ln\left[\left(1-\frac{C}{100}\right)\left(1-\frac{ee}{100}\right)\right]}{\ln\left[\left(1-\frac{C}{100}\right)\left(1+\frac{ee}{100}\right)\right]} \qquad (9)$$

where *C* represents conversion, defined as: $C = 100 - \mathrm{yield}(\mathbf{3a}\text{ or }\mathbf{5a})$

$$s = \frac{\ln\,[(1-0.65)(1-0.69)]}{\ln\,[(1-0.65)(1+069)]} = 4.23 \qquad (10)$$

b) for pseudo-gemderivative

The vial (4 ml) was charged with *rac*-**5a** (29.4 mg, 0.1 mmol, 1.0 equiv.), *pre*-**C1** (7.6 mg, 0.02 mmol, 0.2 equiv.), DQ (22.5 mg, 0.055 mmol, 0.55 equiv.), and Cs_2CO_3 (65.2 mg, 0.2 mmol, 2.0 equiv.), and evacuated and refilled with argon. Then, precooled DCM (0 °C,1.0 ml), MeOH (20 µl, 0.5 mmol, 5.0 equiv.) were added. The reaction was stirred for 15 hours at 0 °C (crycooler). After that time, the solvent was evaporated. The crude product was purified by column chromatography (eluting with hexane/EtOAc – 8:1), affording **5a** (26 mg, 89%) as a white amorphous solid.

Er = 53.2/46.7, the enantiomeric excess of product **5a** was determined by HPLC using a Chiralpak® IB column (*n*-heptane/*i*-PrOH - 80:20, flow rate = 1.0 ml/min, λ = 190 nm, *t* = 25 °C): t_R = 9.3 min (major), t_R = 14.3 min (minor).

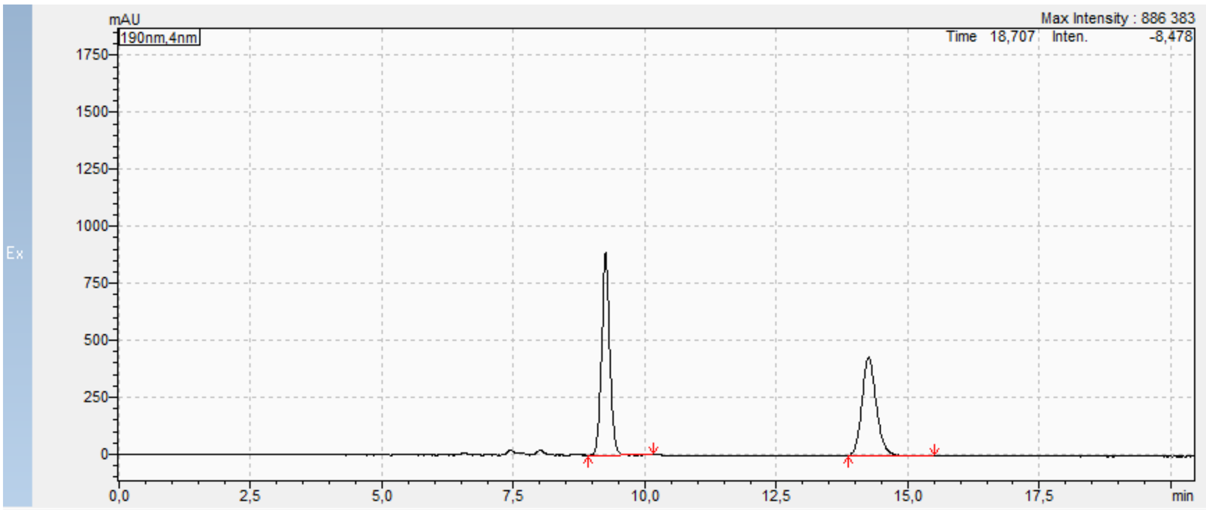

Results View - Peak Table

Peak Table | Compound | Group | Calibration Curve

Peak#	Ret. Time	Area	Height	Peak Start	Peak End	Mark	Conc.	Unit	Area%
1	9,248	9333324	888920	8,907	10,165	M	53,306		53,306
2	14,259	8175575	432432	13,856	15,509	M	46,694		46,694
Total		17508899	1321352				100,000		100,000

Supplementary Fig. 18. Chiral HPLC trace of **5a** obtained in the kinetic resolution control experiment.

Supplementary equations
Resolution rate

$$V_R/V_S = \frac{53.31}{46.69} = \frac{1.14}{1} \quad (11)$$

Selectivity factor

$$s = \frac{\ln\,[(1 - 0.11)(1 - 0.07)]}{\ln\,[(1 - 0.11)(1 + 0.06)]} = 0.09 \quad (12)$$

Crystallographic data

The x-ray experiments were performed on a Bruker D8 VENTURE Kappa Duo PHOTONIII diffractometer with a IµS micro-focus sealed tube CuKα (λ = 1.54178 Å) at a temperature of 120(2) K. The structure was solved using direct methods (XT)[16] and refined by full matrix least squares based on F^2 (SHELXL2019).[17] The hydrogen atoms on carbon were fixed to idealized positions (riding model) and assigned temperature factors using either H_{iso}(H) = 1.2 U_{eq}(pivot atom) or H_{iso}(H) = 1.5 U_{eq} (pivot atom) for the methyl moiety. The absolute structure determination was based on anomalous dispersion of oxygen atoms.

The crystal of **5a** and its enantiomer *ent*-**5a** were refined as two component non-merohedral twin with the same twin matrix: -1 0 -0.0857; 0 -1 0; 0 0 1; the volume ration of twins parts were refined 0.52:0.48 and 0.51:0.49 for **3a** and *ent*-**5a** respectively.

Crystal data for **3a**: $C_{19}H_{18}O_3$, M_r = 294.33; Monoclinic , P 2_1, (No 4), a = 7.4955 (4) Å, b = 11.5740 (6) Å, c = 16.8669 (8) Å , β = 95.163 (1)°,V = 1457.32 (13) Å^3, Z = 4, D_x = 1.342 Mg m^{-3}, Plate, colourless of dimensions 0.43 × 0.21 × 0.06 mm, multi-scan absorption correction (µ = 0.72 mm^{-1}) T_{min} = 0.84, T_{max} = 0.96; a total of 32311 measured reflections (θ_{max} = 79.5°), from which 6028 were unique (R_{int} = 0.036) and 5968 observed according to the

I > 2σ(I) criterion. The refinement converged (Δ/$σ_{max}$= 0.001) to R = 0.033 for observed reflections and wR(F^2) = 0.089, GOF = 1.07 for 403 parameters and all 6028 reflections. The final difference map displayed no peaks of chemical significance (Δ$ρ_{max}$ = 0.29, Δ$ρ_{min}$ -0.25 e.Å^{-3}). Absolute structure parameter: -0.03 (4)[18]

Crystal data for **5a**: $C_{19}H_{18}O_3$, M_r = 294.33; Monoclinic , P 2_1, (No 4), a = 11.9456 (4) Å, b = 7.4176 (2) Å, c = 16.3431 (6) Å , $β$ = 93.368 (2)°, V = 1445.62 (8) Å^3, Z = 4, D_x = 1.352 Mg m^{-3}, Prism, colourless of dimensions 0.21 × 0.16 × 0.06 mm, multi-scan absorption correction (μ = 0.73 mm^{-1}) T_{min} = 0.68, T_{max} = 0.75; a total of 40674 measured reflections ($θ_{max}$ = 79.0°), from which 10239 were unique (R_{int} = 0.053) and 9879 observed according to the I > 2σ(I) criterion. The refinement converged (Δ/$σ_{max}$= 0.001) to R = 0.039 for observed reflections and wR(F^2) = 0.103, GOF = 1.03 for 400 parameters and all 10239 reflections. The final difference map displayed no peaks of chemical significance (Δ$ρ_{max}$ = 0.23, Δ$ρ_{min}$ -0.21 e.Å^{-3}). Absolute structure parameter: -0.03 (9)[18]

Crystal data for *ent*-**5a**: $C_{19}H_{18}O_3$, M_r = 294.33; Monoclinic , P 2_1, (No 4), a = 11.9498 (4) Å, b = 7.4217 (3) Å, c = 16.3330 (6) Å , $β$ = 93.365 (1)°, V = 1446.04 (9) Å^3, Z = 4, D_x = 1.352 Mg m^{-3}, Prism, colourless of dimensions 0.22 × 0.22 × 0.20 mm, multi-scan absorption correction (μ = 0.73 mm^{-1}) T_{min} = 0.79, T_{max} = 0.87; a total of 43901 measured reflections ($θ_{max}$ = 77.4°), from which 10289 were unique (R_{int} = 0.031) and 10072 observed according to the I > 2σ(I) criterion. The refinement converged (Δ/$σ_{max}$= 0.001) to R = 0.031 for observed reflections and wR(F^2) = 0.085, GOF = 1.03 for 400 parameters and all 10289 reflections. The final difference map displayed no peaks of chemical significance (Δ$ρ_{max}$ = 0.19, Δ$ρ_{min}$ -0.18 e.Å^{-3}). Absolute structure parameter: 0.01 (6)[18]

CheckCIF files provided during the submission process do not reveal any A- or B-level alerts except comprehensible publication errors regarding missing references to published work.

X-ray crystallographic data were deposited with the Cambridge Crystallographic Data Centre (CCDC) under deposition number 2302458, 2302459 and 2309999 for **3a**, **5a**, and *ent*-**5a**, respectively and can be obtained free of charge from the Centre via its website (https://www.ccdc.cam.ac.uk/structures/).

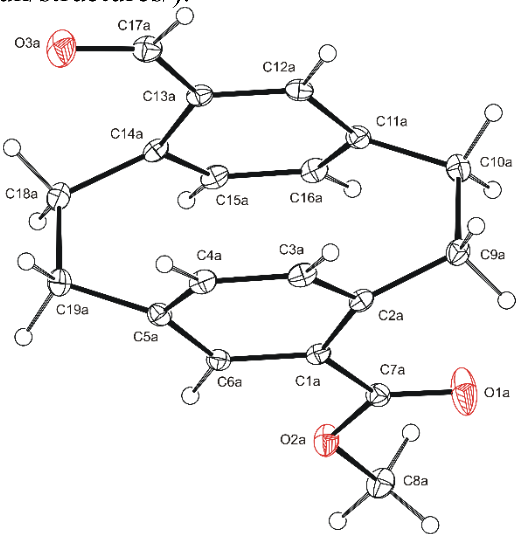

Supplementary Fig. 19. View on one of two symmetrically independent molecule of **3a** with atom numbering schema; the displacement ellipsoid is drawn on 30% probability level (CCDC: 2302458).

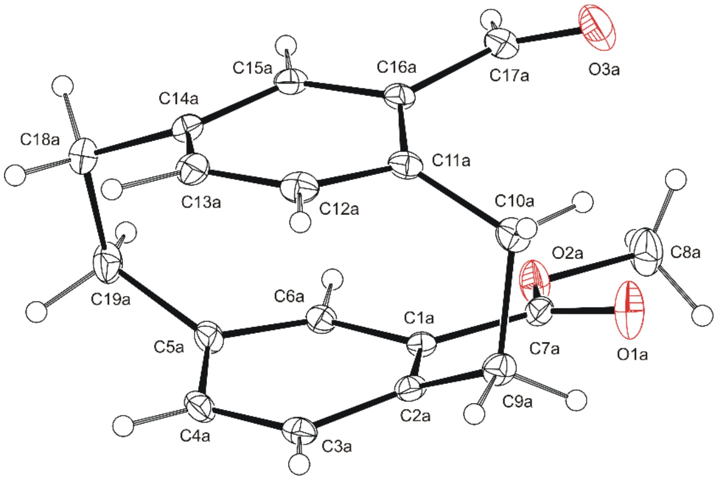

Supplementary Fig. 20. View on one of two symmetrically independent molecule of **5a** with atom numbering schema; the displacement ellipsoid is drawn on 30% probability level (CCDC: 2302459).

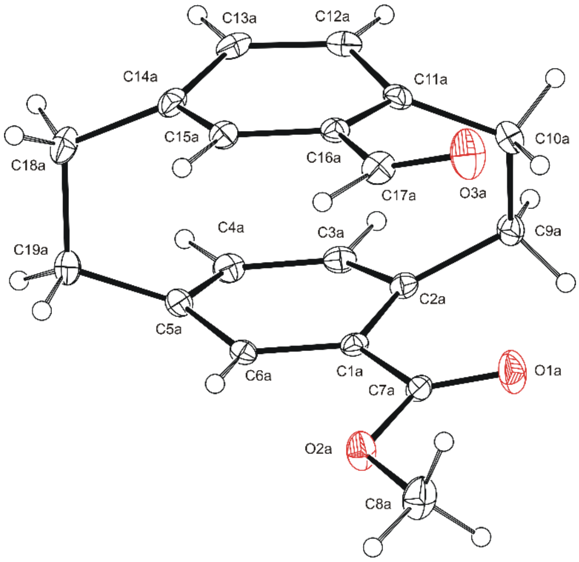

Supplementary Fig. 21. View on one of two symmetrically independent molecule of *ent*-**5a** with atom numbering schema; the displacement ellipsoid is drawn on 30% probability level (CCDC: 2309999).

NMR spectra

(R_p)-4^3-Formyl-1,4(1,4)-dibenzenacyclohexaphane-1^2-yl acetate (3a)

^{1}H NMR of **3a** (400 MHz, $CDCl_3$)

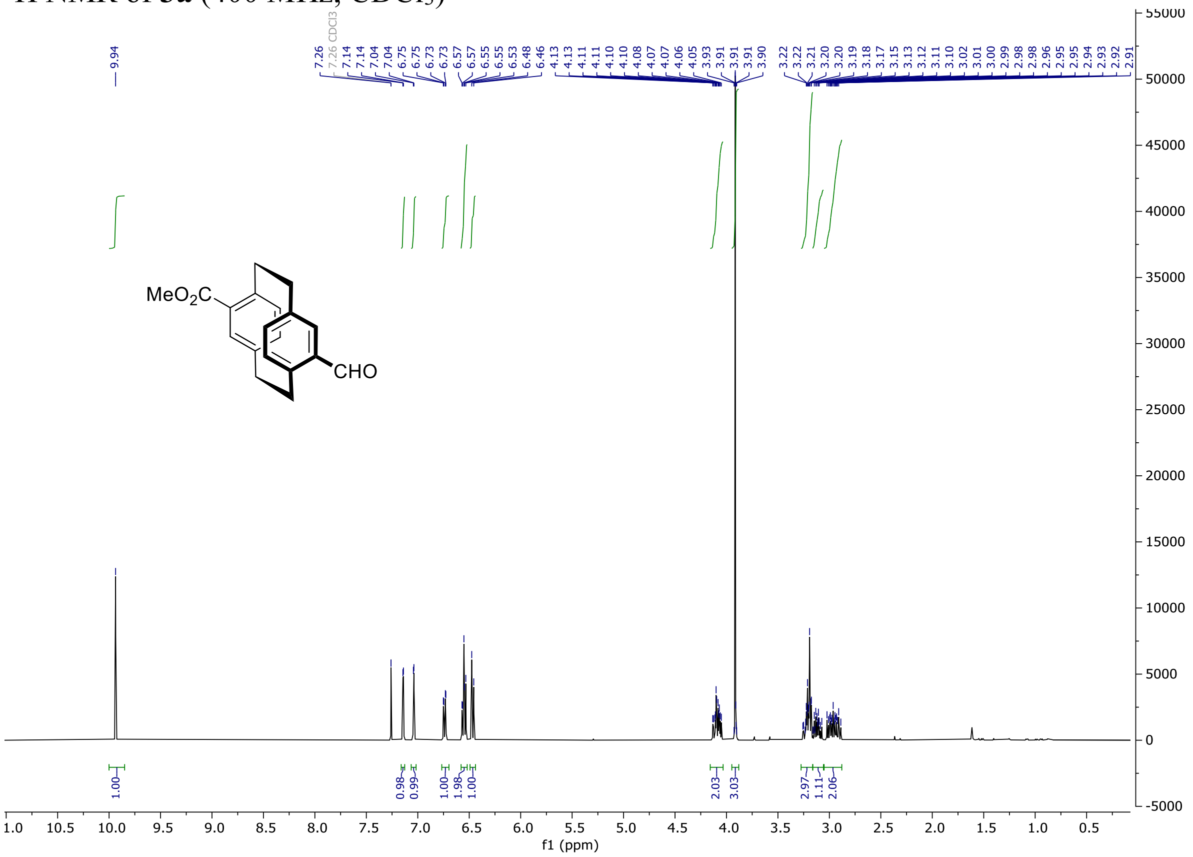

^{13}C{^{1}H} NMR of **3a** (101 MHz, $CDCl_3$)

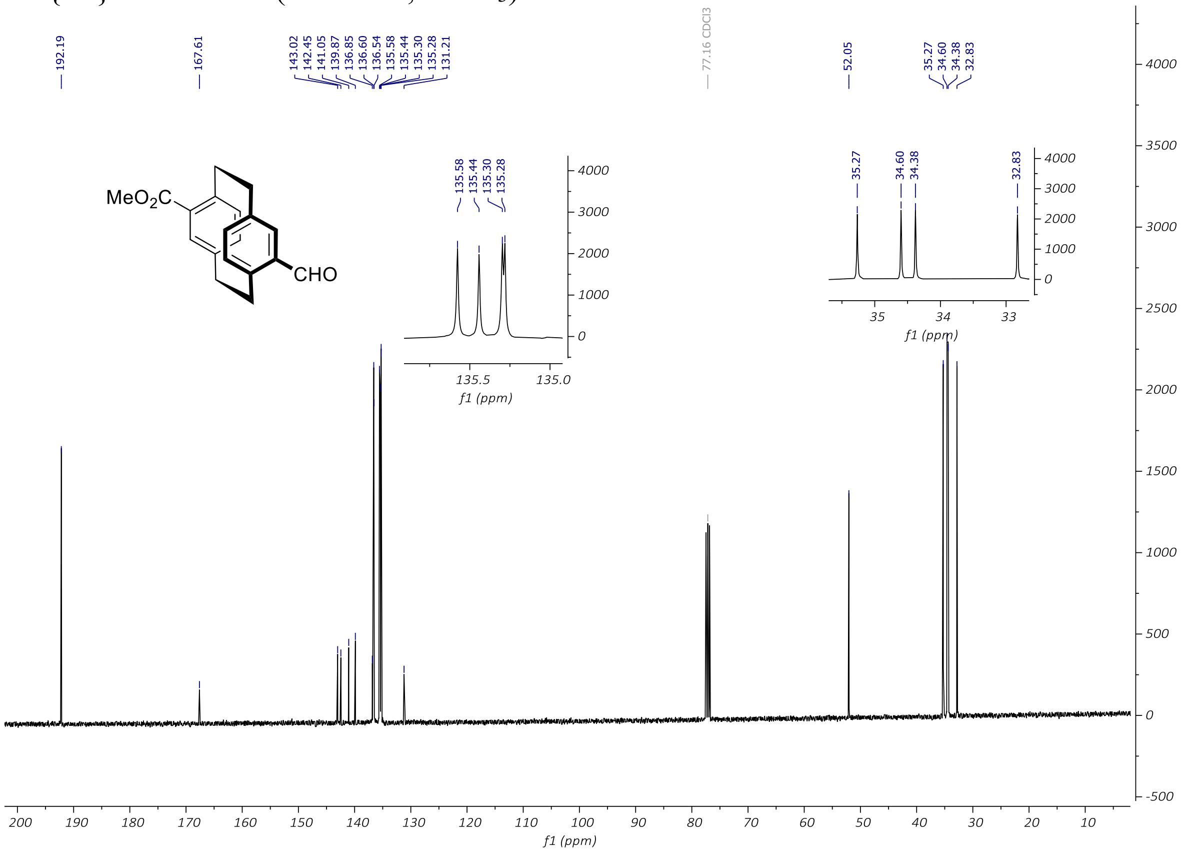

Dimethyl 4^3-acetoxy-1,4(1,4)-dibenzenacyclohexaphane-1^2-carboxylate (4a)

^{1}H NMR of **4a** (400 MHz, $CDCl_3$)

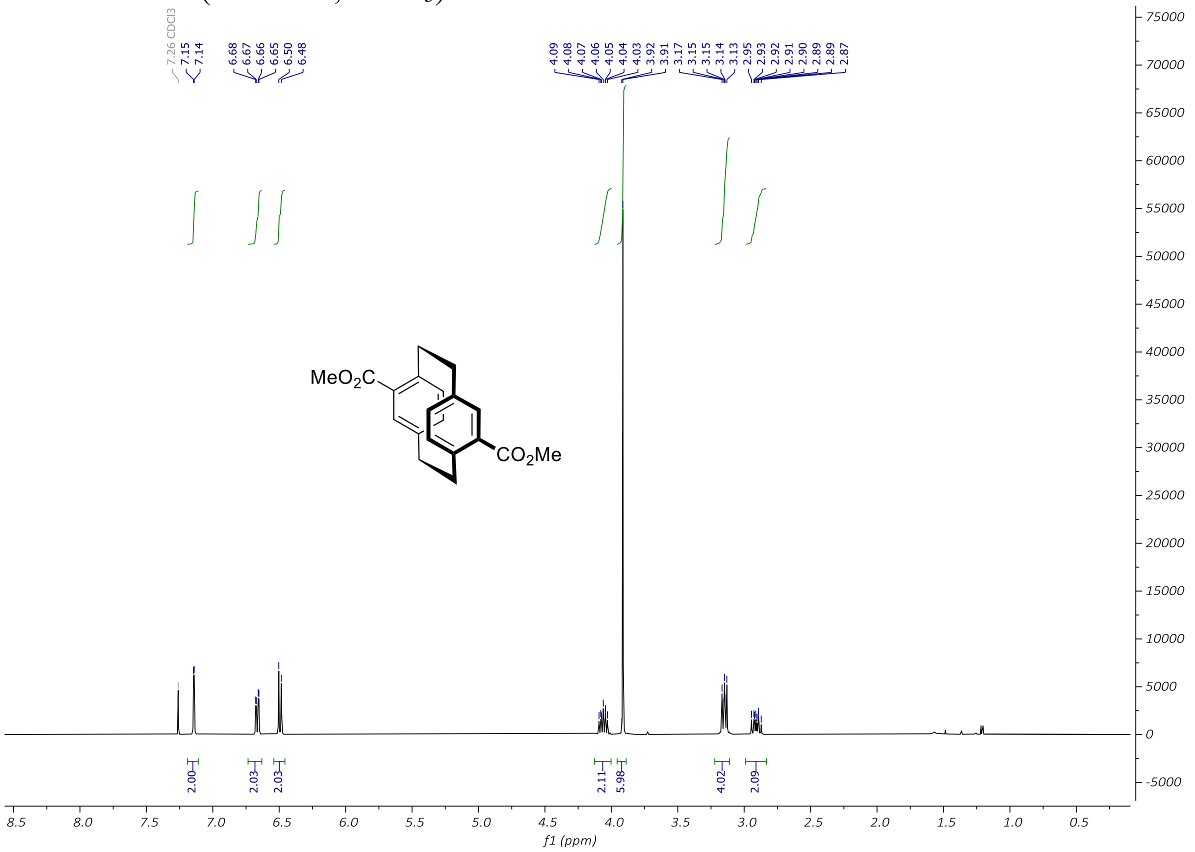

$^{13}C\{^1H\}$ NMR of **4a** (101 MHz, $CDCl_3$)

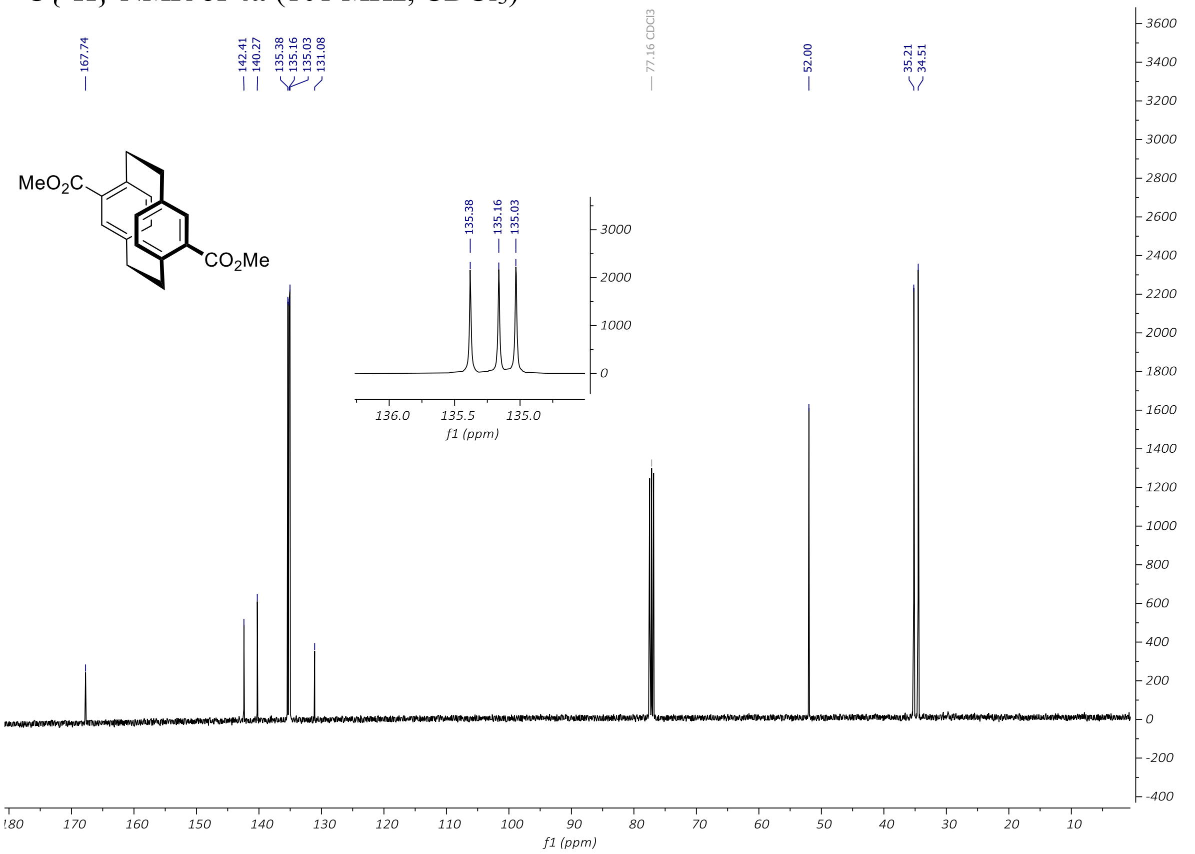

(R_p)-Ethyl 4^3-formyl-1,4(1,4)-dibenzenacyclohexaphane-1^2-carboxylate (3b)

^{1}H NMR of **3b** (400 MHz, $CDCl_3$)

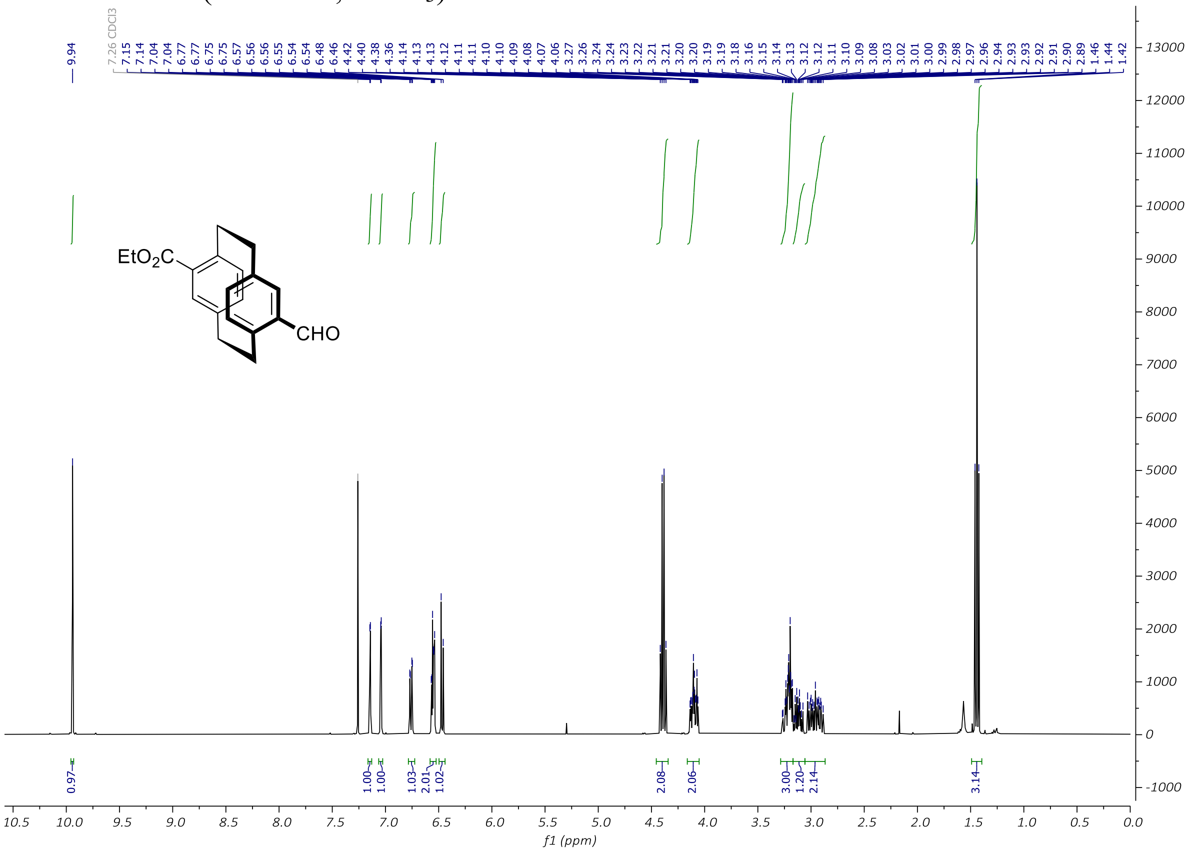

^{13}C{^{1}H} NMR of **3b** (101 MHz, $CDCl_3$)

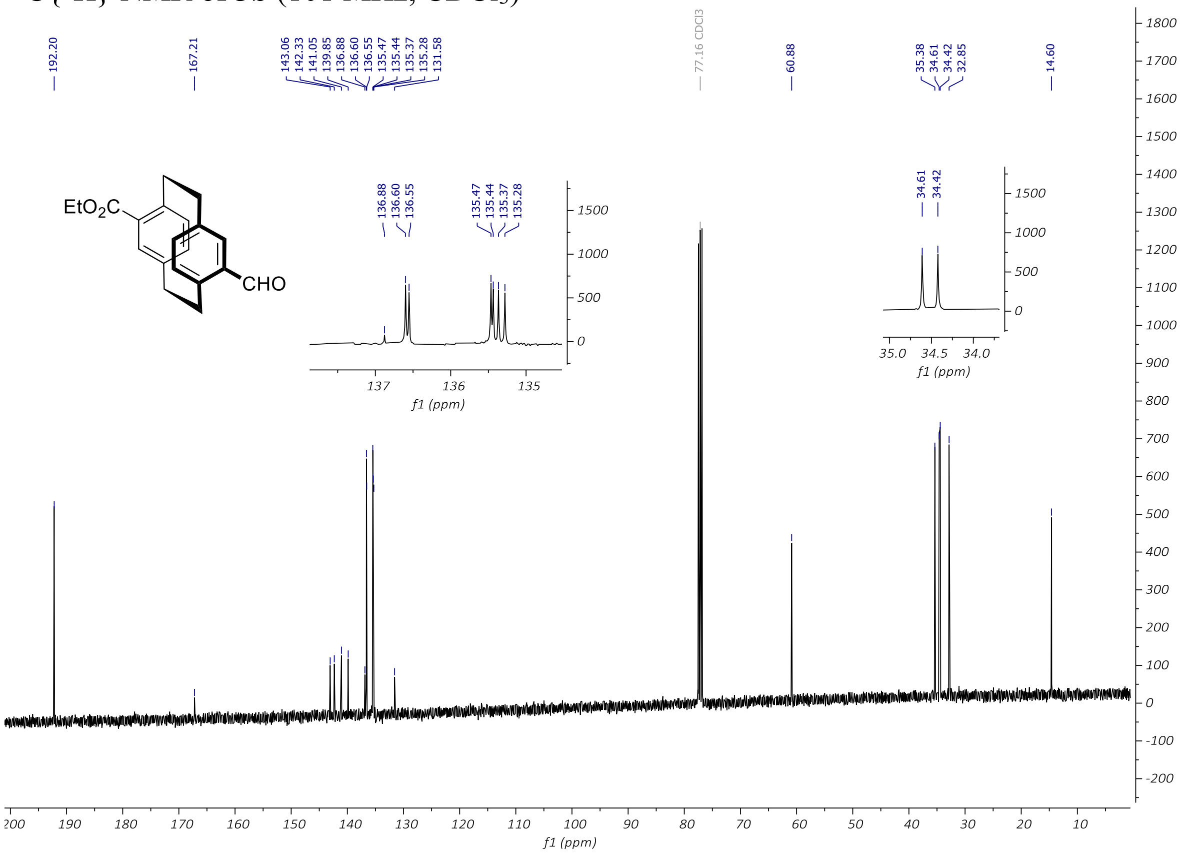

Diethyl 1,4(1,4)-dibenzenacyclohexaphane-1^2,4^3-dicarboxylate (4b)

^{1}H NMR of **4b** (400 MHz, $CDCl_3$)

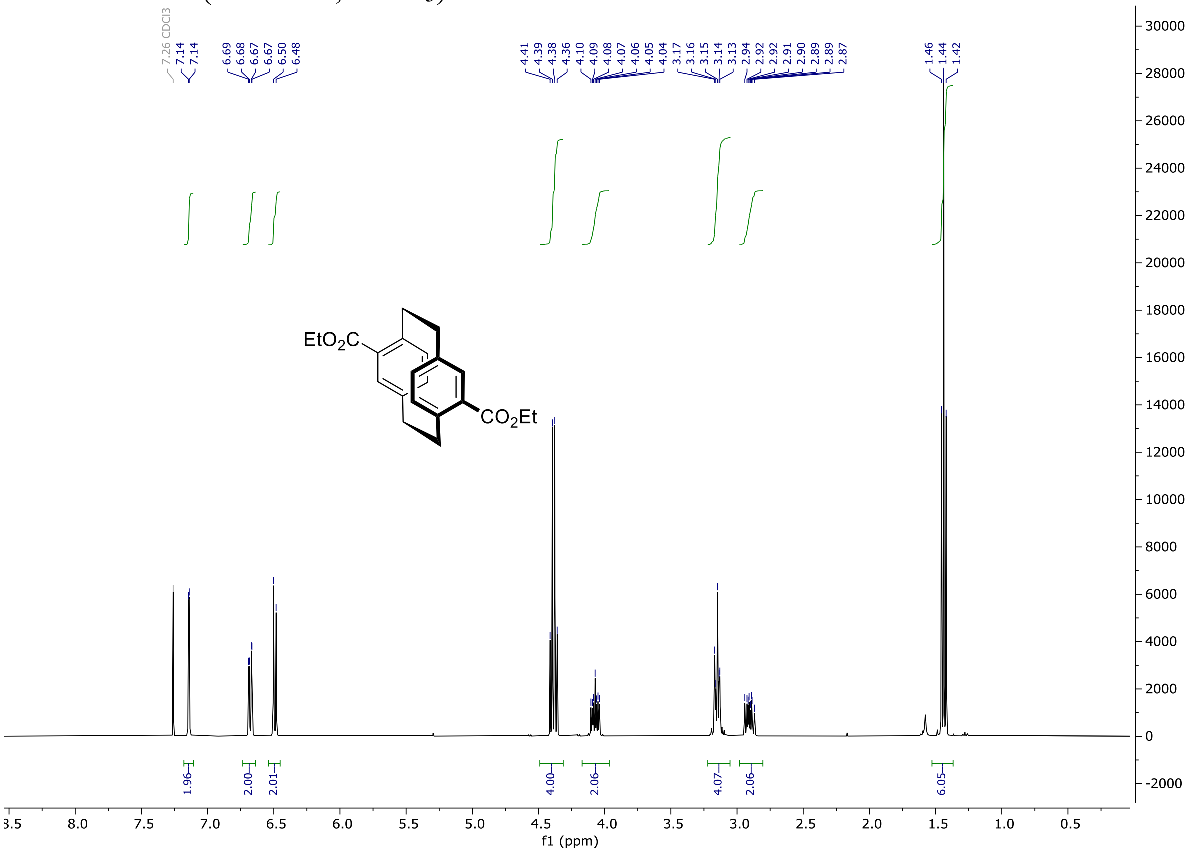

$^{13}C\{^1H\}$ NMR of **4b** (101 MHz, $CDCl_3$)

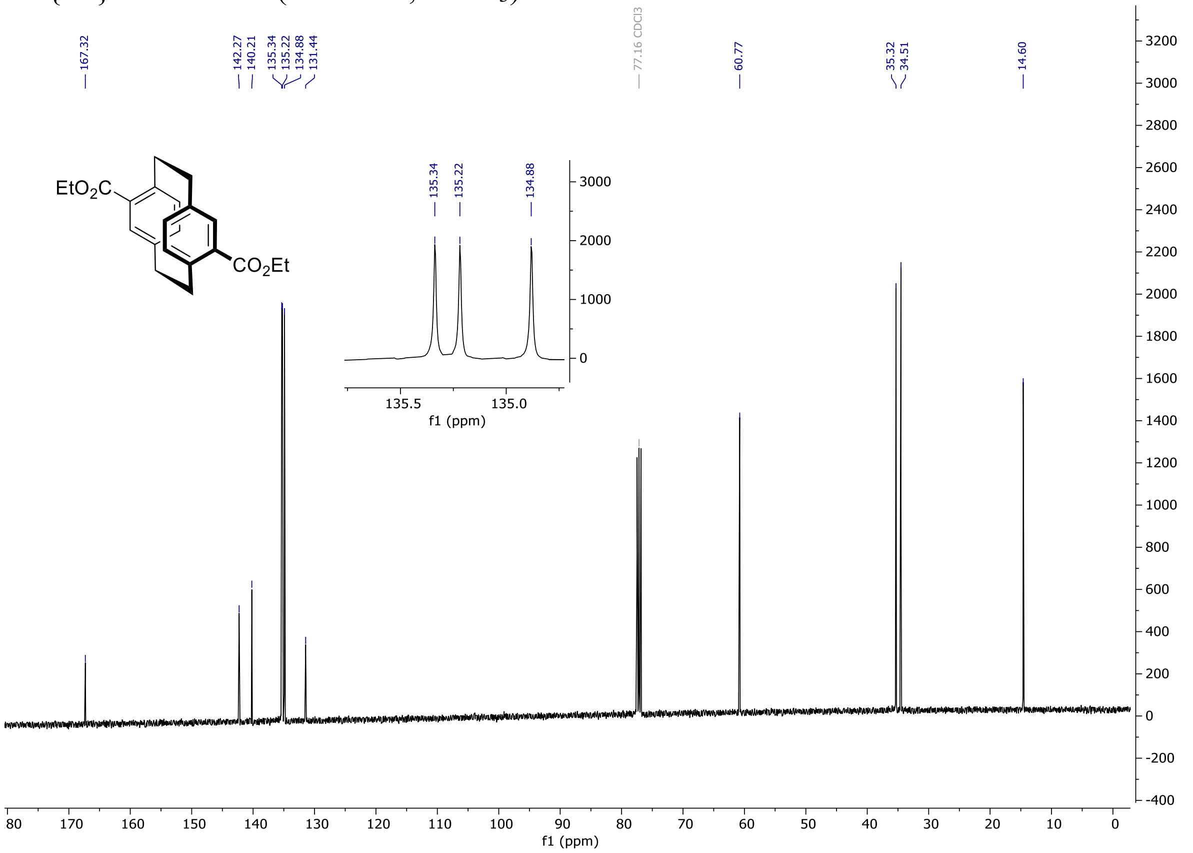

(R_p)-Isopropyl 4^3-formyl-1,4(1,4)-dibenzenacyclohexaphane-1^2-carboxylate (3c)

^{1}H NMR of **3c** (400 MHz, $CDCl_3$)

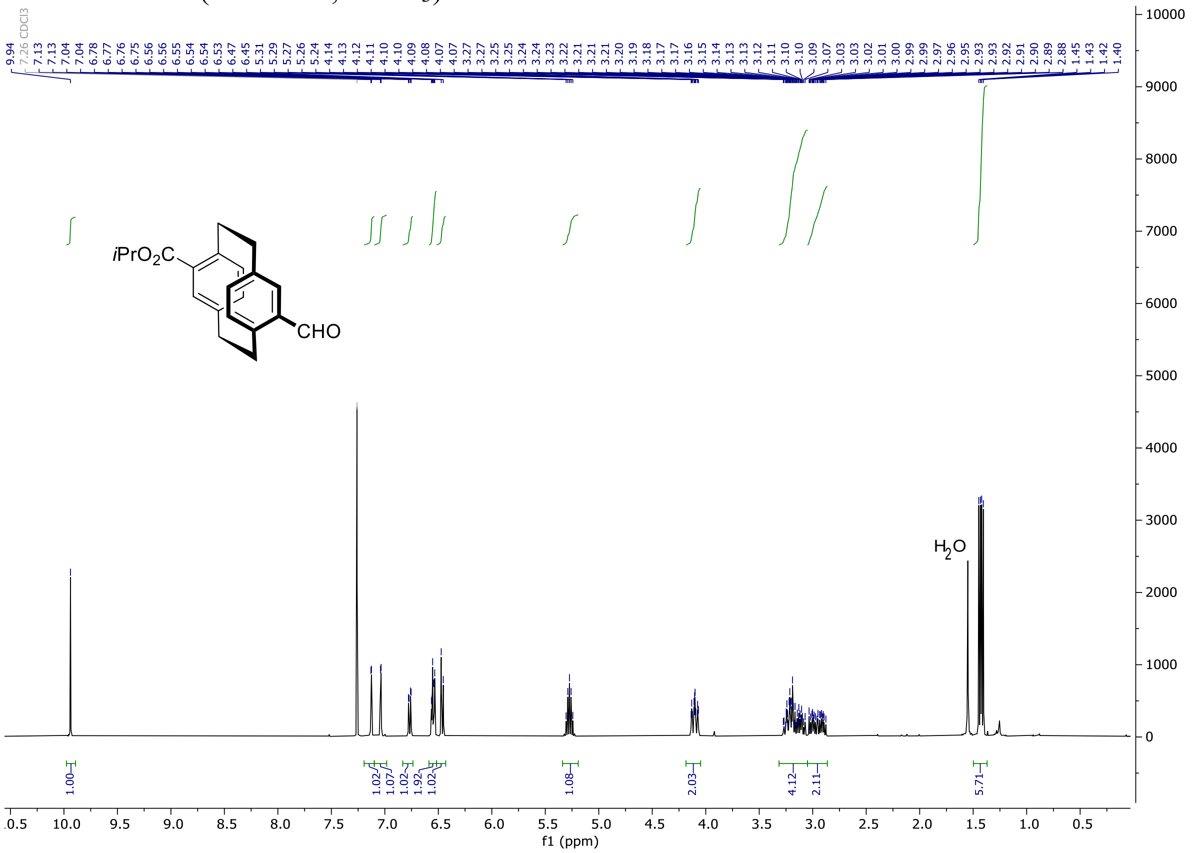

^{13}C{^{1}H} NMR of **3c** (101 MHz, $CDCl_3$)

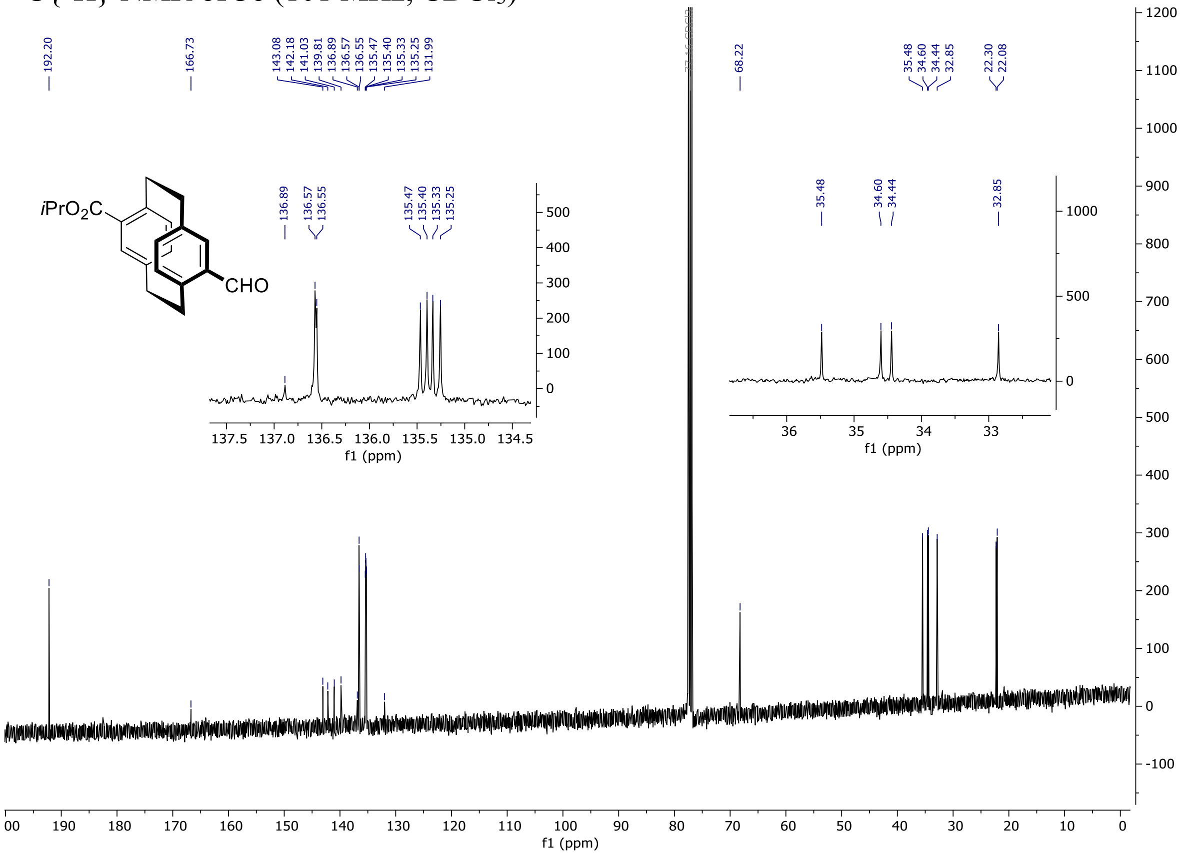

(R_p)-4^3-(1-(Oxo-λ^3-methoxy)-1λ^5-dodecyl)-1,4(1,4)-dibenzenacyclohexaphane-1^2-carbaldehyde (3d)

^{1}H NMR of **3d** (400 MHz, $CDCl_3$)

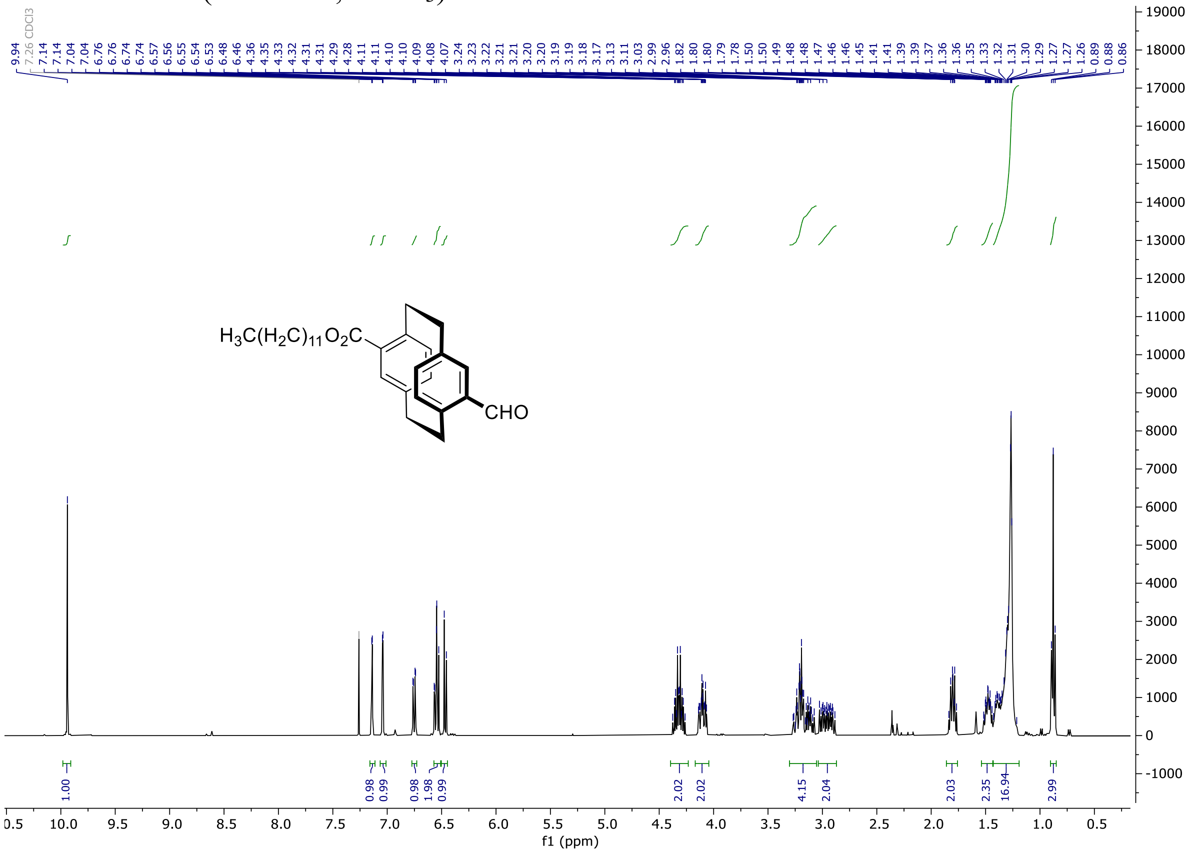

^{13}C{^{1}H} NMR of **3d** (101 MHz, $CDCl_3$)

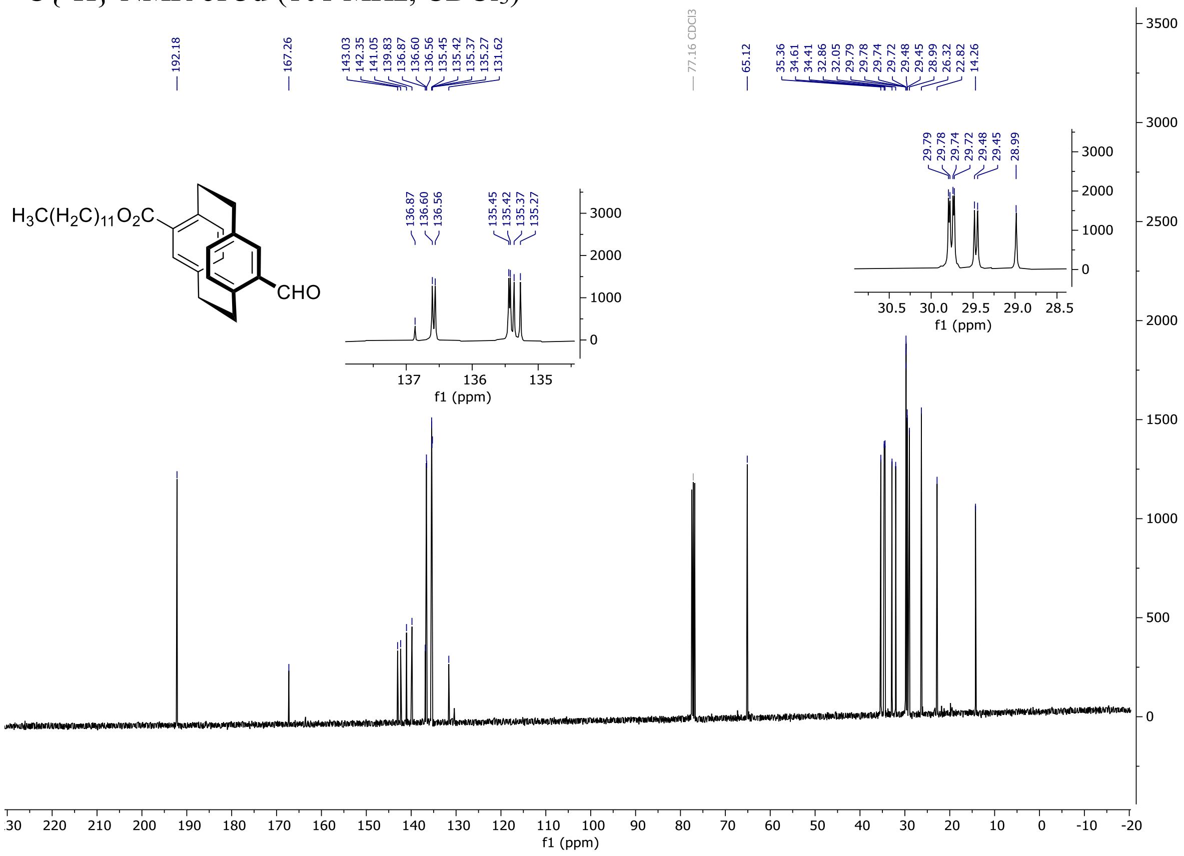

(R_p)-2-Methoxyethyl 4[3]-formyl-1,4(1,4)-dibenzenacyclohexaphane-1[2]-carboxylate (3e)

^{1}H NMR of **3e** (400 MHz, $CDCl_3$)

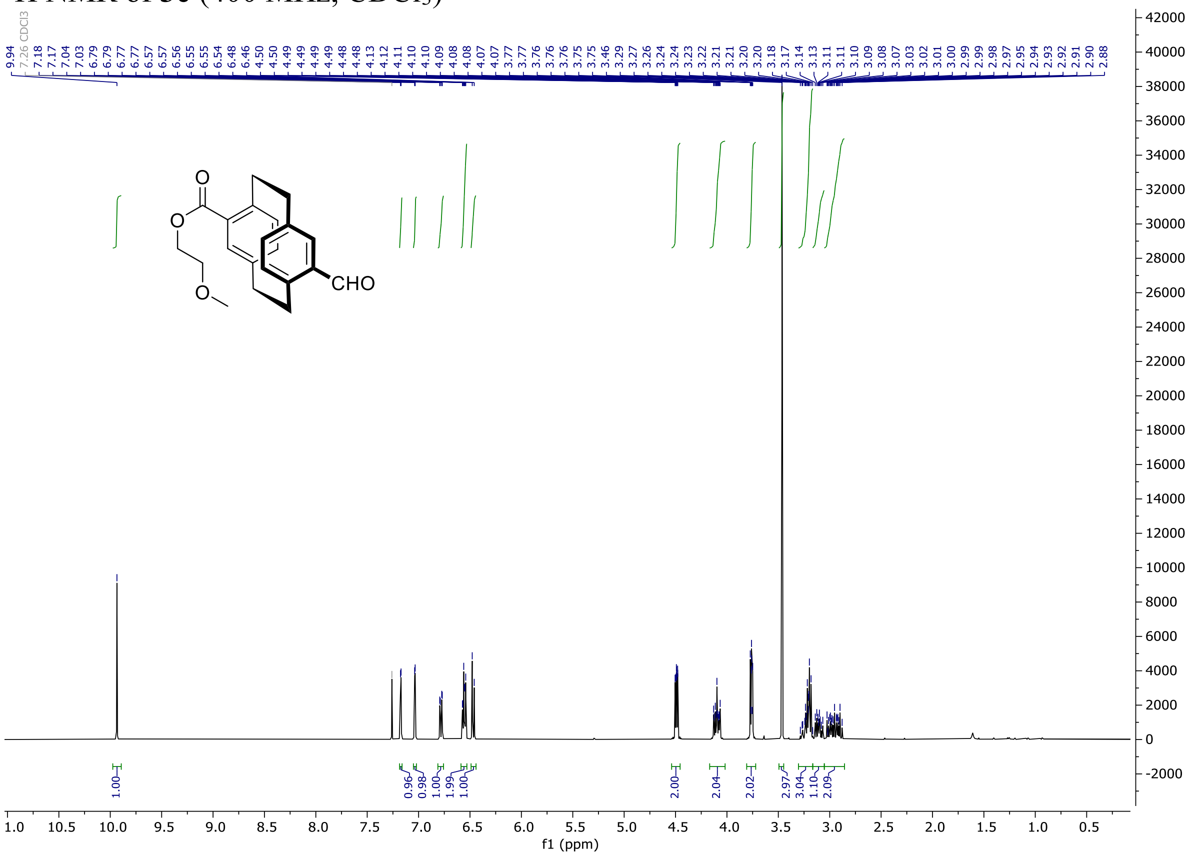

^{13}C{^{1}H} NMR of **3e** (101 MHz, $CDCl_3$)

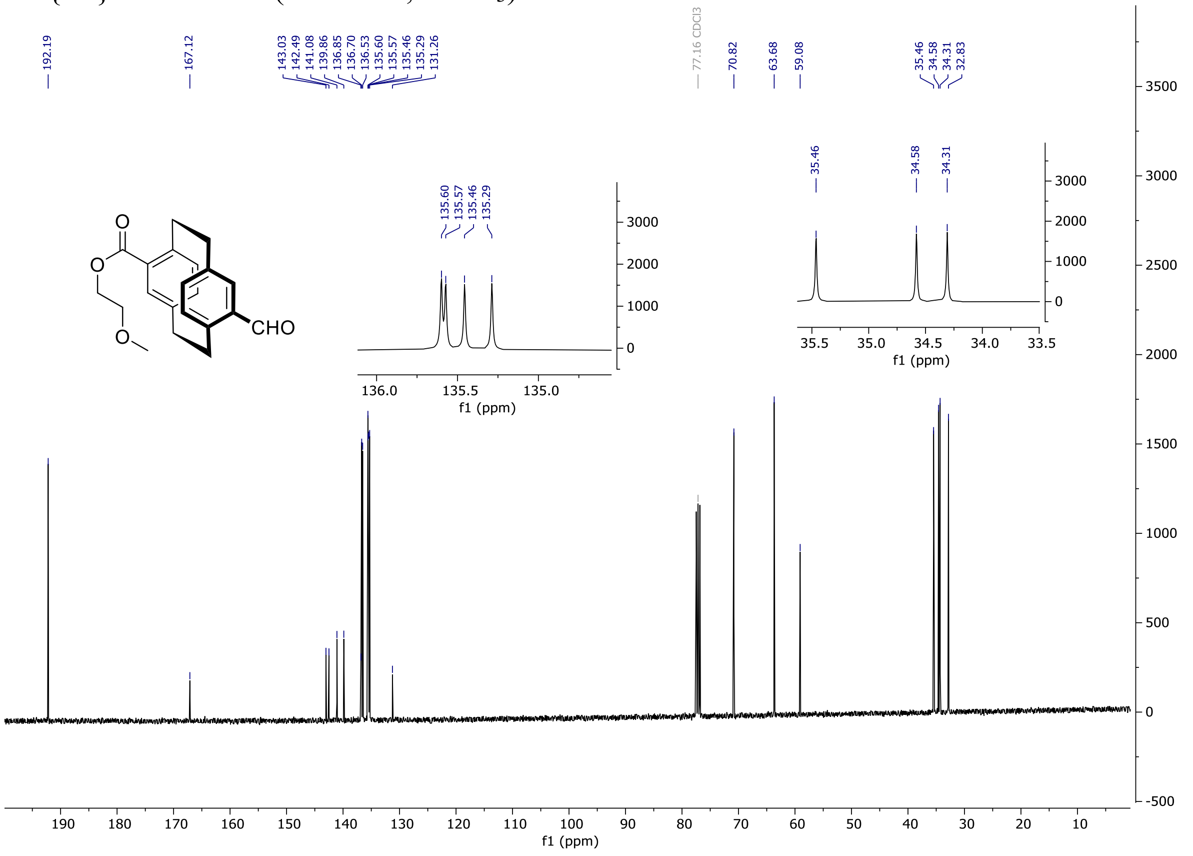

(R_p)-2-Bromoethyl 4[3]-formyl-1,4(1,4)-dibenzenacyclohexaphane-1[2]-carboxylate (3f)

^{1}H NMR of **3f** (400 MHz, $CDCl_3$)

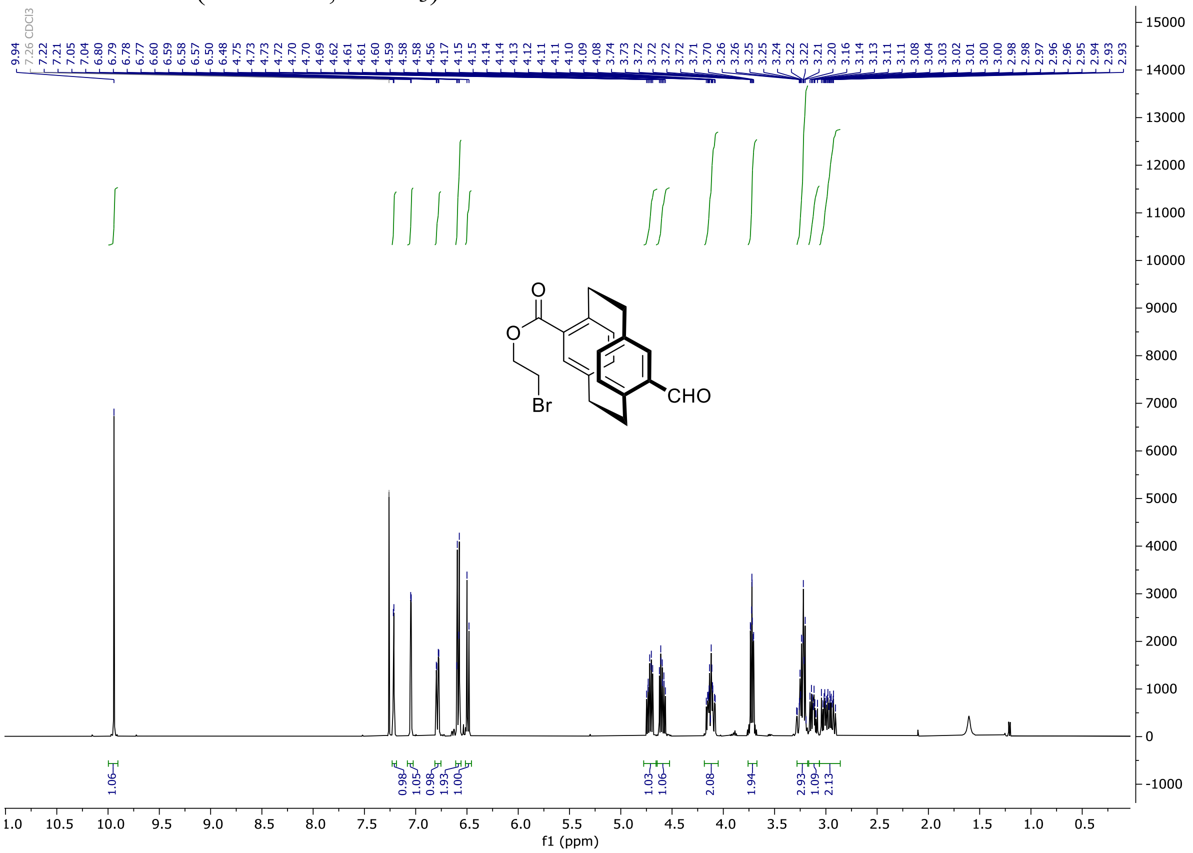

^{13}C{^{1}H} NMR of **3f** (101 MHz, $CDCl_3$)

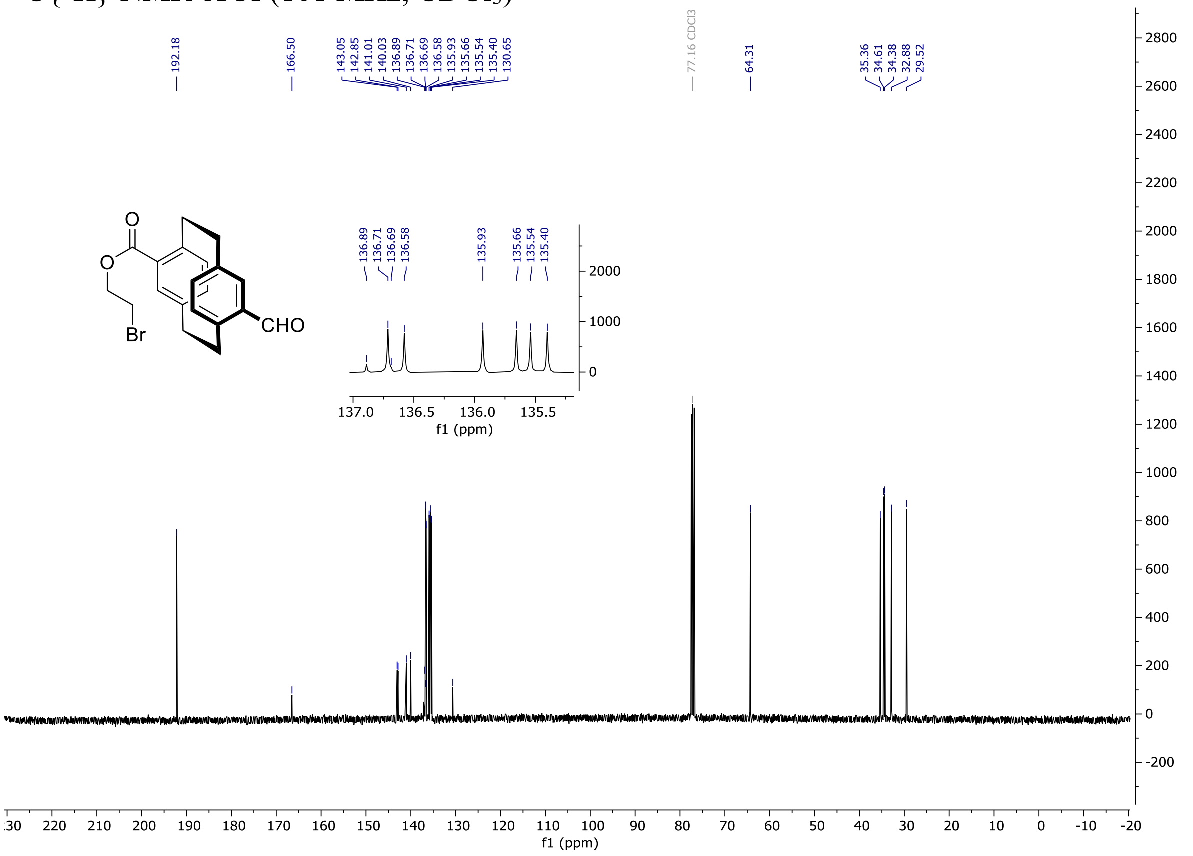

(R_p)-(Z)-Hex-3-en-1-yl 4³-formyl-1,4(1,4)-dibenzenacyclohexaphane-1²-carboxylate (3g)

^{1}H NMR of **3g** (400 MHz, CDCl$_3$)

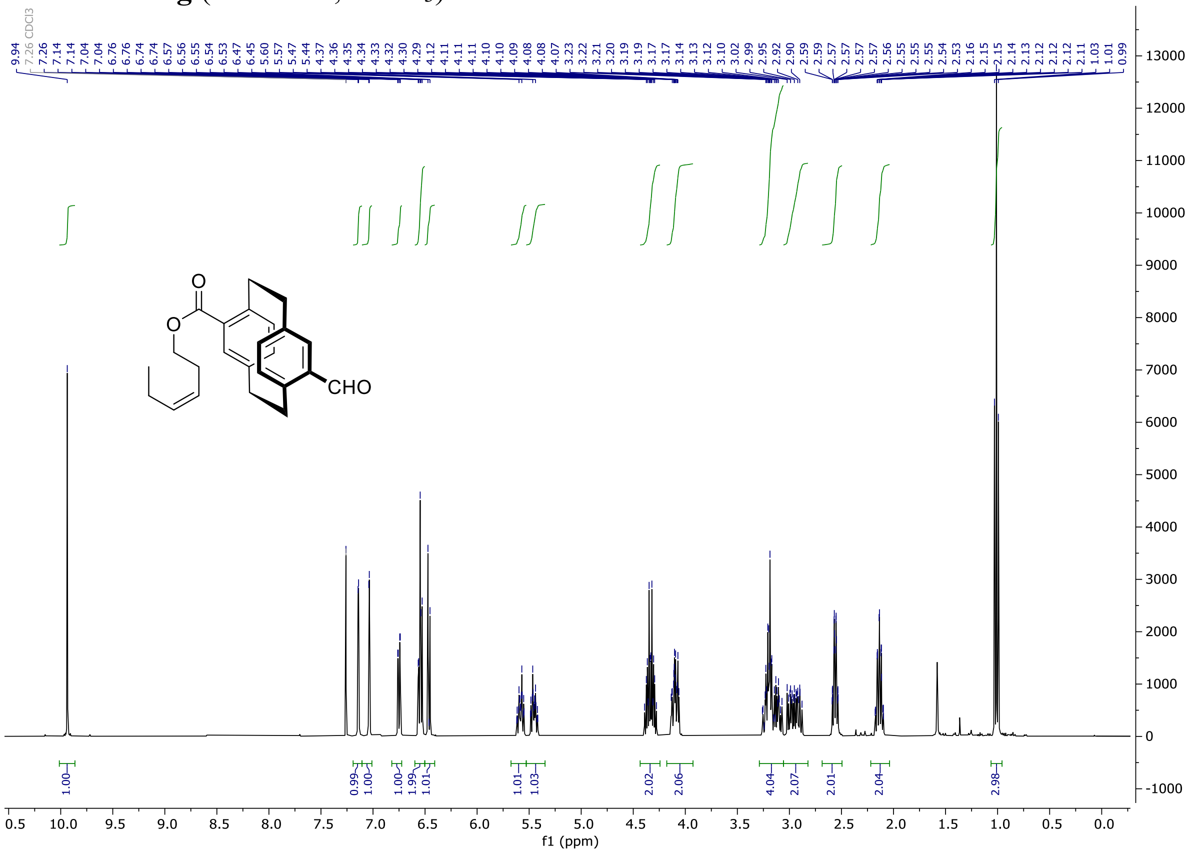

^{13}C{^{1}H} NMR of **3g** (101 MHz, CDCl$_3$)

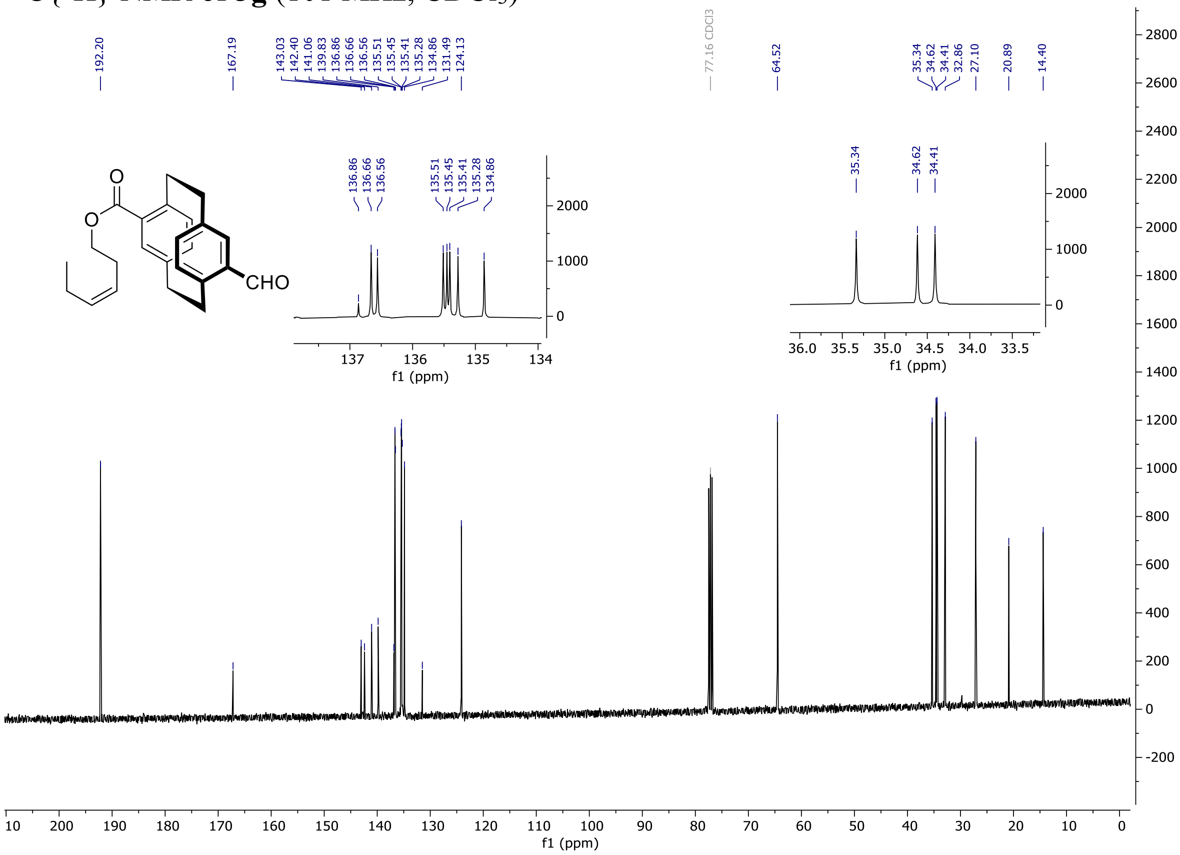

(R_p)-Pent-4-en-1-yl 4[3]-formyl-1,4(1,4)-dibenzenacyclohexaphane-1[2]-carboxylate (3h)

^{1}H NMR of **3h** (400 MHz, $CDCl_3$)

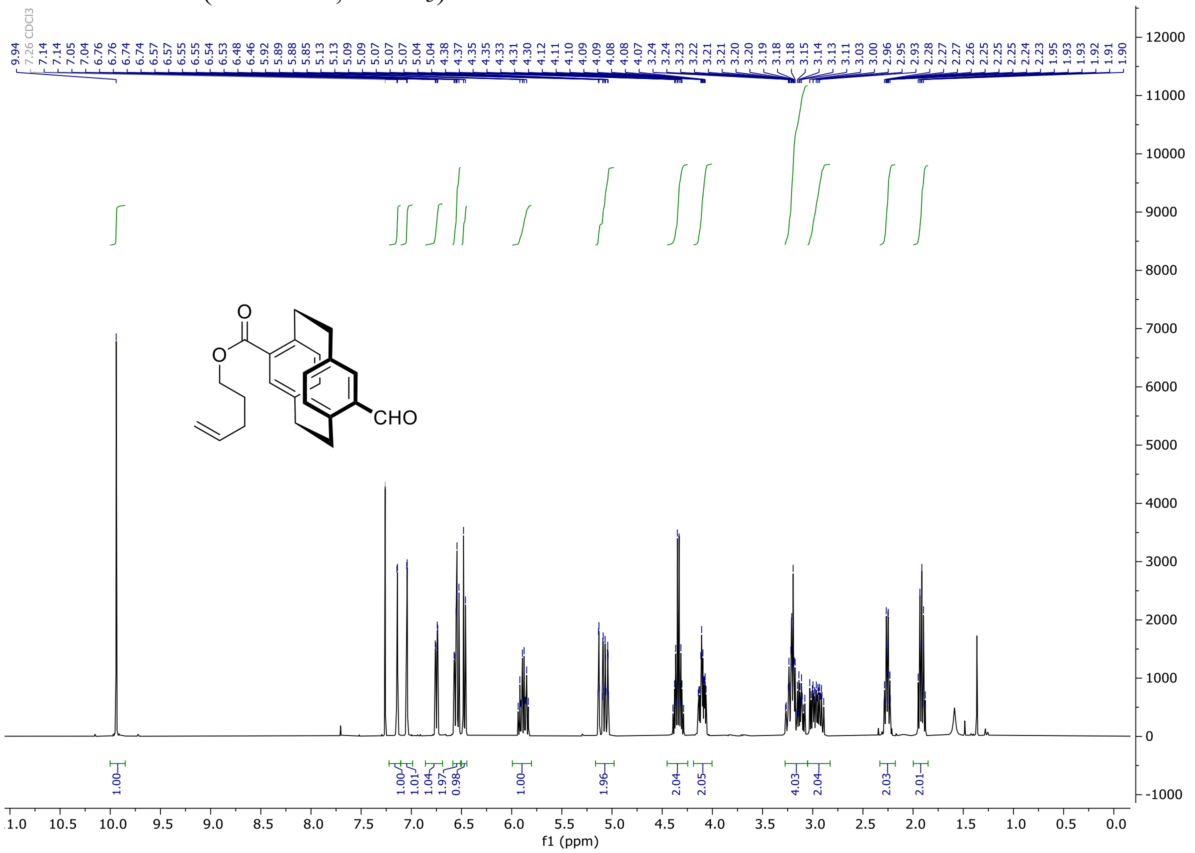

^{13}C{^{1}H} NMR of **3h** (101 MHz, $CDCl_3$)

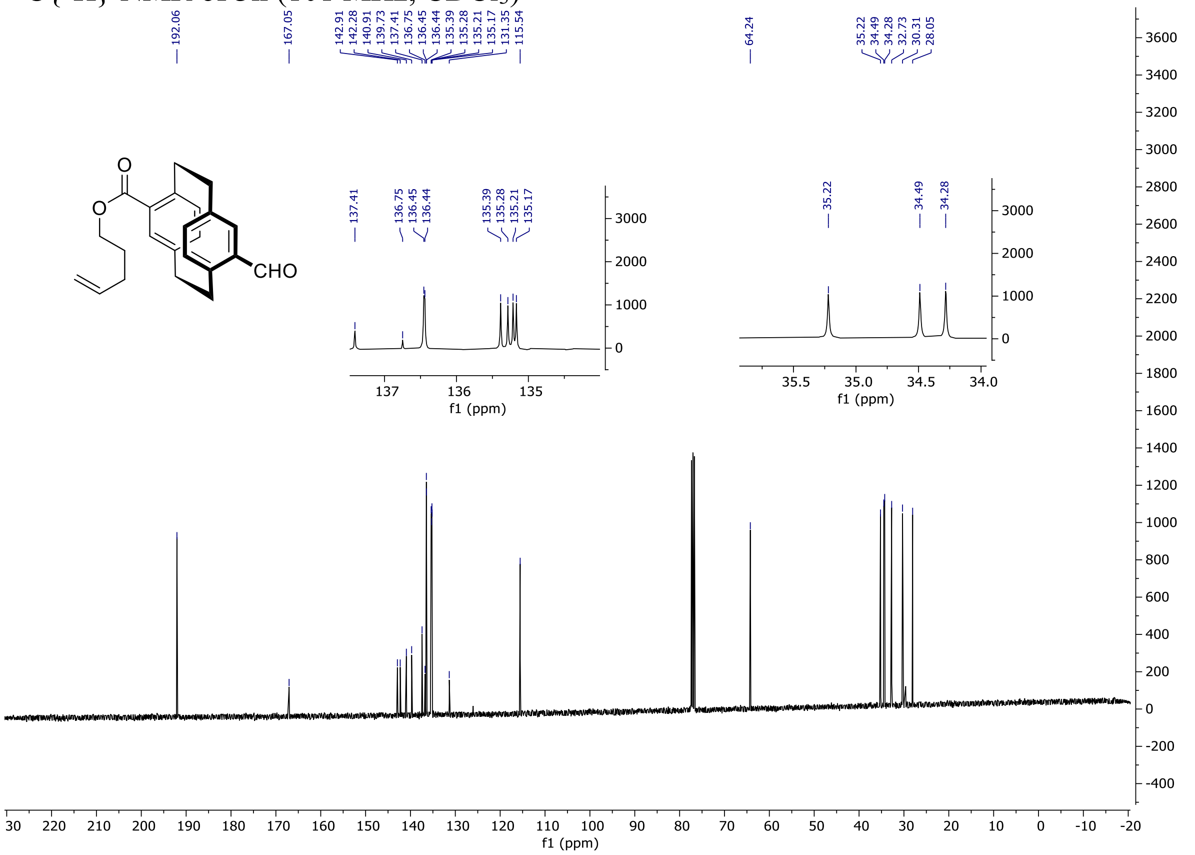

(R_p)-Pent-3-yn-1-yl 4[3]-formyl-1,4(1,4)-dibenzenacyclohexaphane-1[2]-carboxylate (3i)

^{1}H NMR of **3i** (400 MHz, $CDCl_3$)

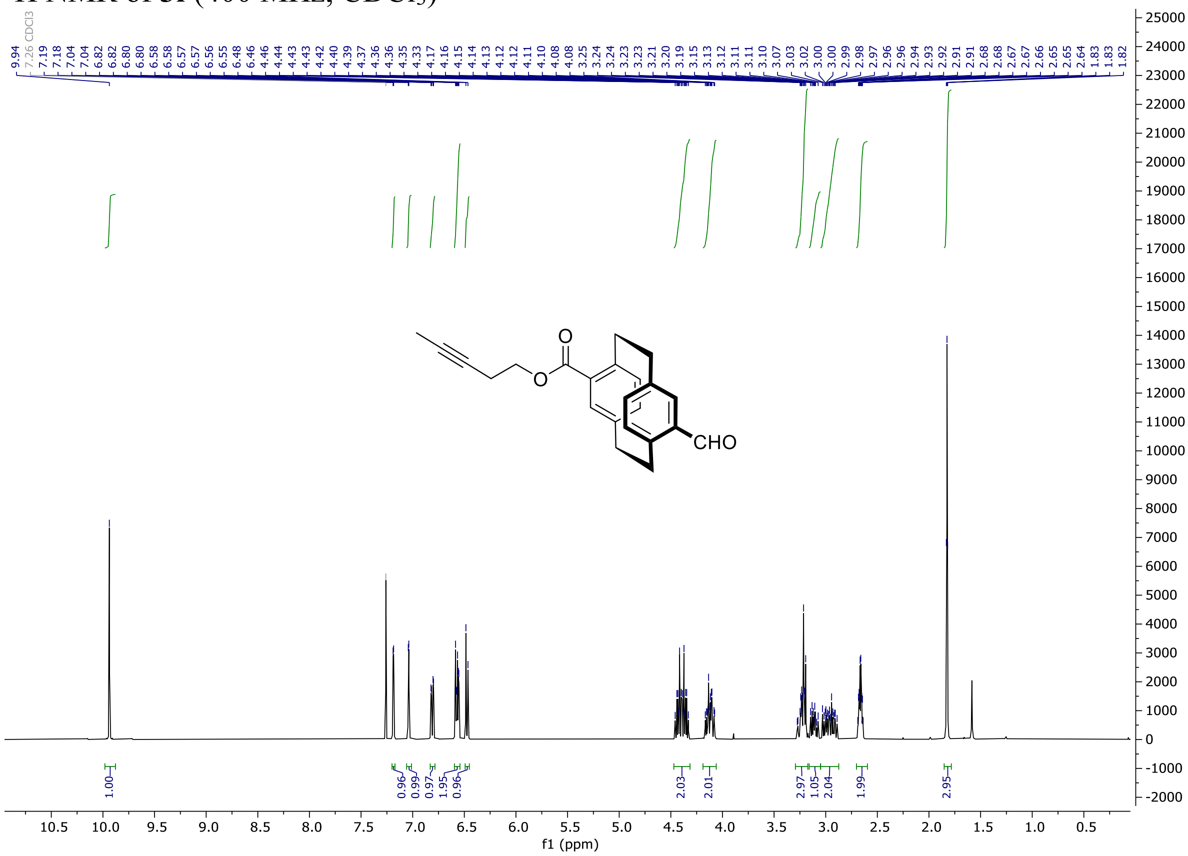

^{13}C{^{1}H} NMR of **3i** (101 MHz, $CDCl_3$)

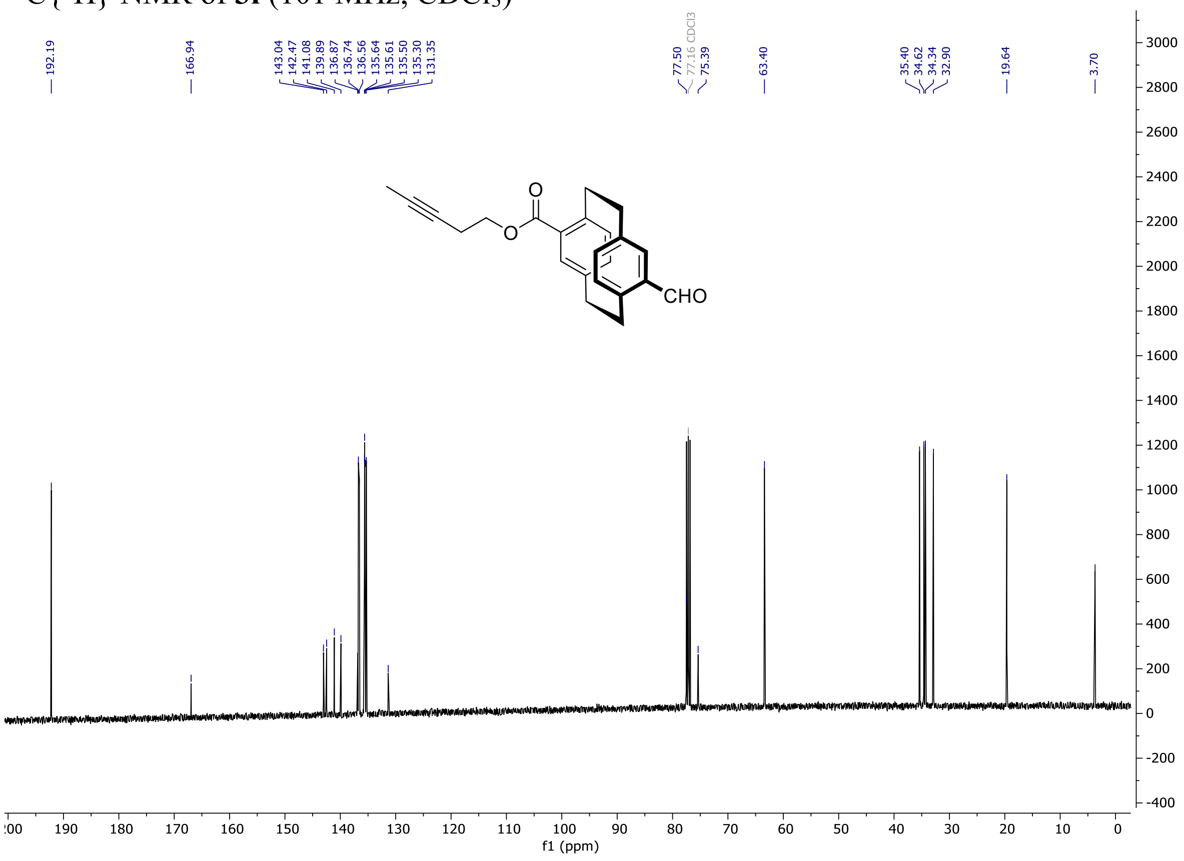

(R_p)-But-3-yn-1-yl 4³-formyl-1,4(1,4)-dibenzenacyclohexaphane-1²-carboxylate (3j)

^{1}H NMR of **3j** (400 MHz, $CDCl_3$)

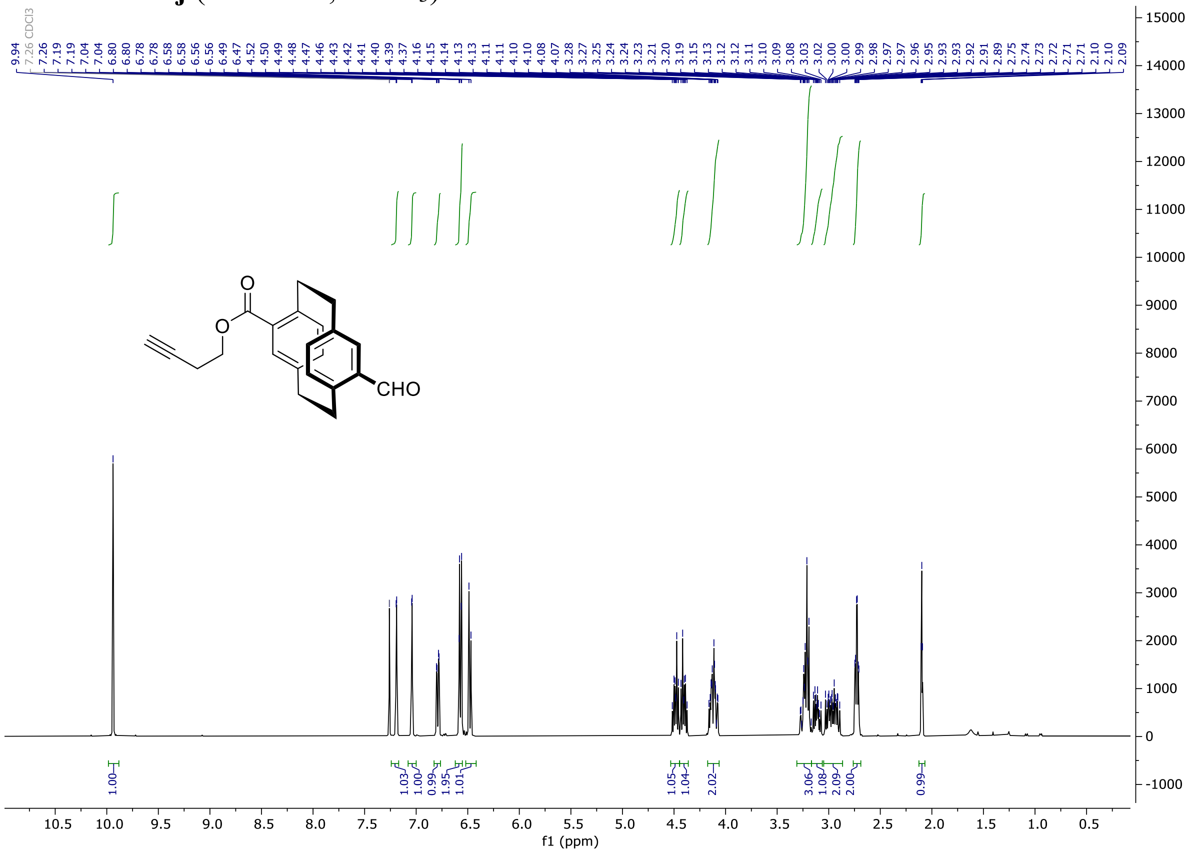

^{13}C{^{1}H} NMR of **3j** (101 MHz, $CDCl_3$)

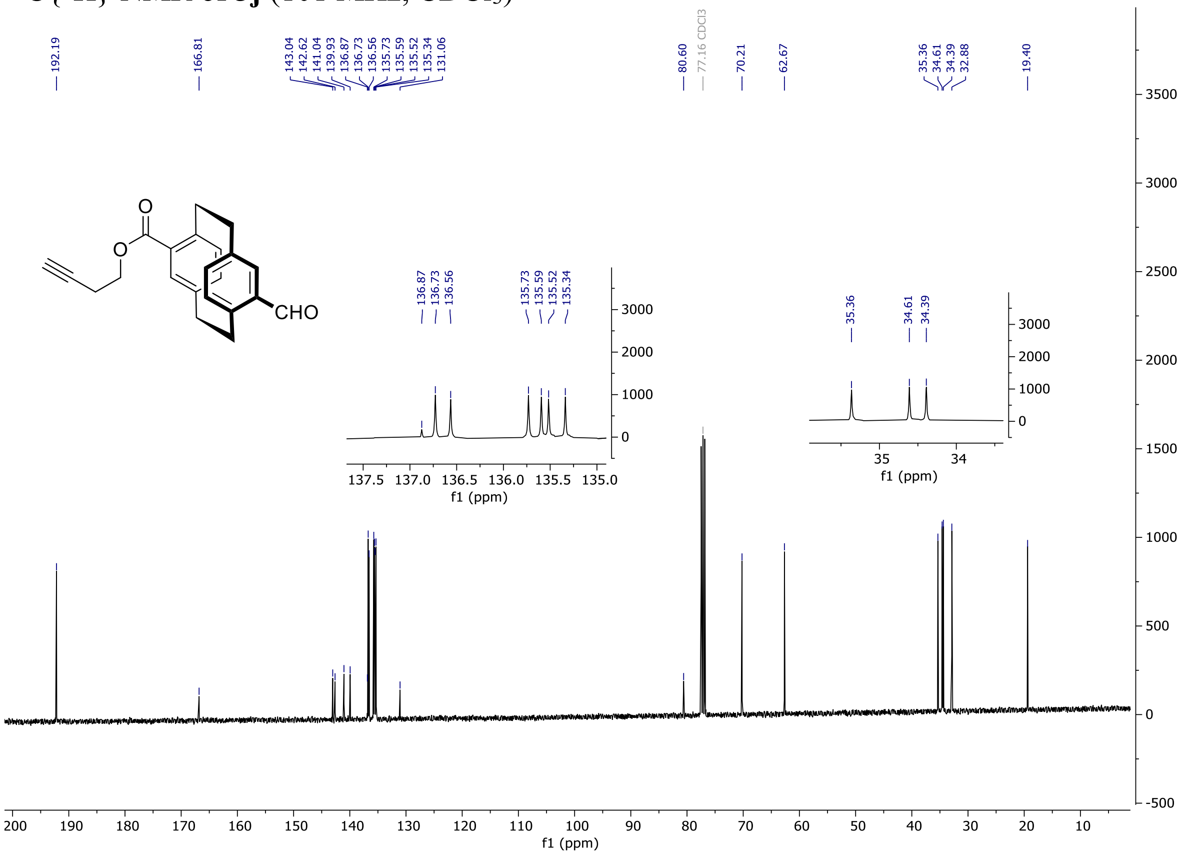

(R_p)-Benzyl 4^3-formyl-1,4(1,4)-dibenzenacyclohexaphane-1^2-carboxylate (3k)

^{1}H NMR of **3k** (400 MHz, $CDCl_3$)

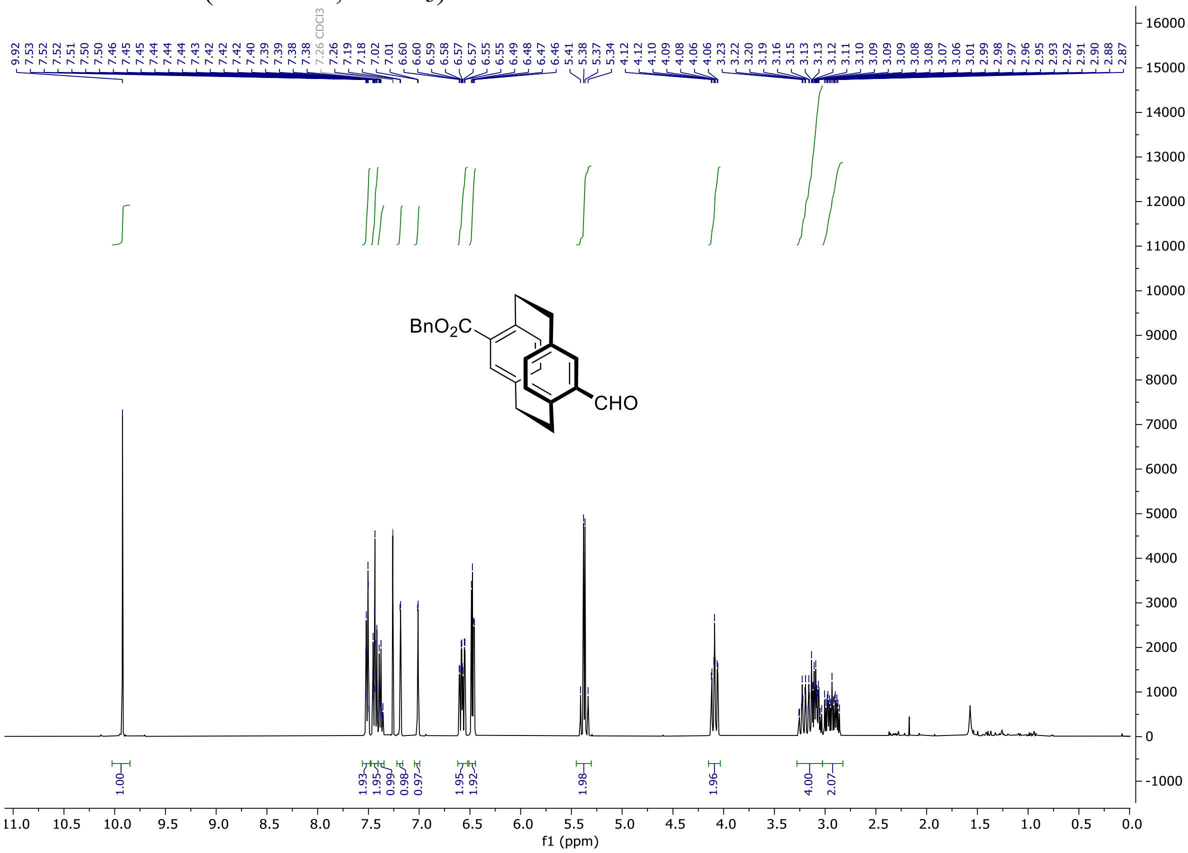

$^{13}C\{^1H\}$ NMR of **3k** (101 MHz, $CDCl_3$)

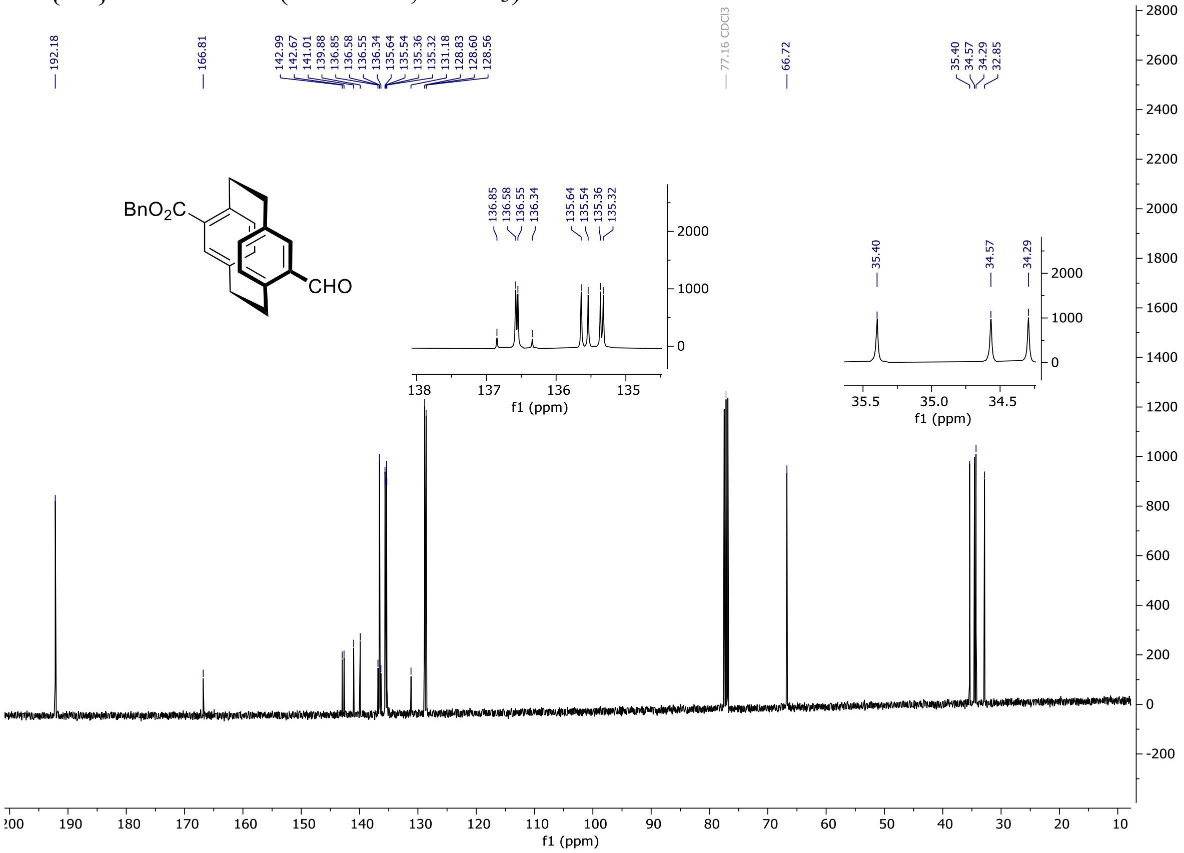

(R_p)-Phenethyl 4[3]-formyl-1,4(1,4)-dibenzenacyclohexaphane-1[2]-carboxylate (3l)

^{1}H NMR of **3l** (400 MHz, $CDCl_3$)

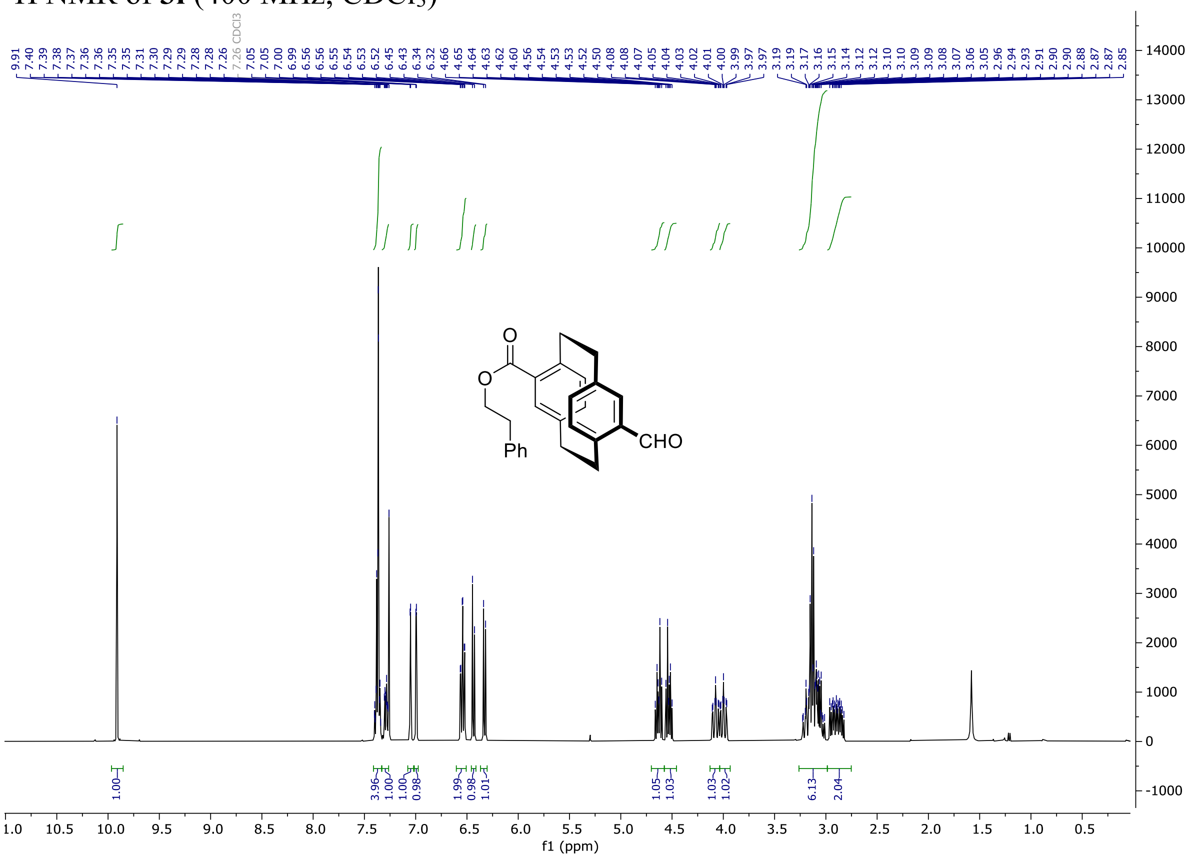

$^{13}C\{^1H\}$ NMR of **3l** (101 MHz, $CDCl_3$)

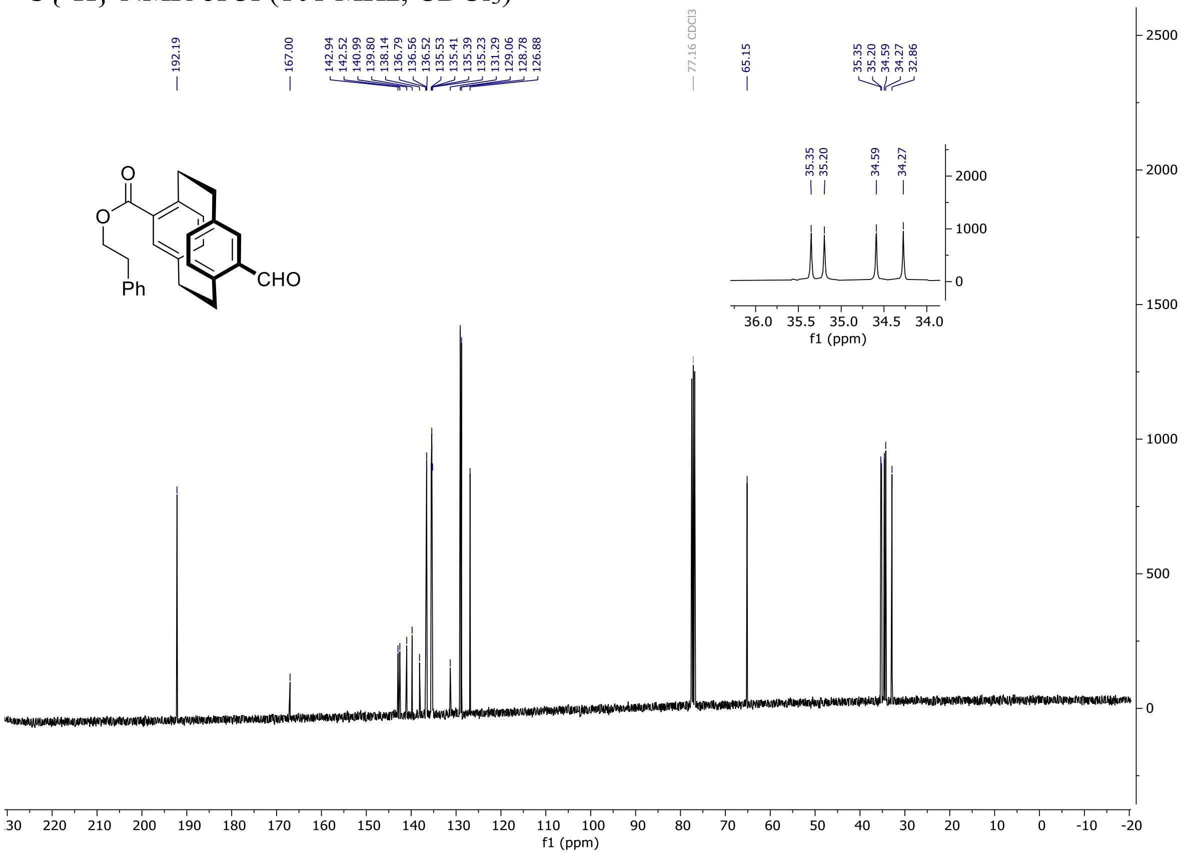

(R_p)-2-(Ferrocen-1-yl)ethyl 4³-formyl-1,4(1,4)-dibenzenacyclohexaphane-1²-carboxylate (3m)

^{1}H NMR of **3m** (400 MHz, CD_3CN)

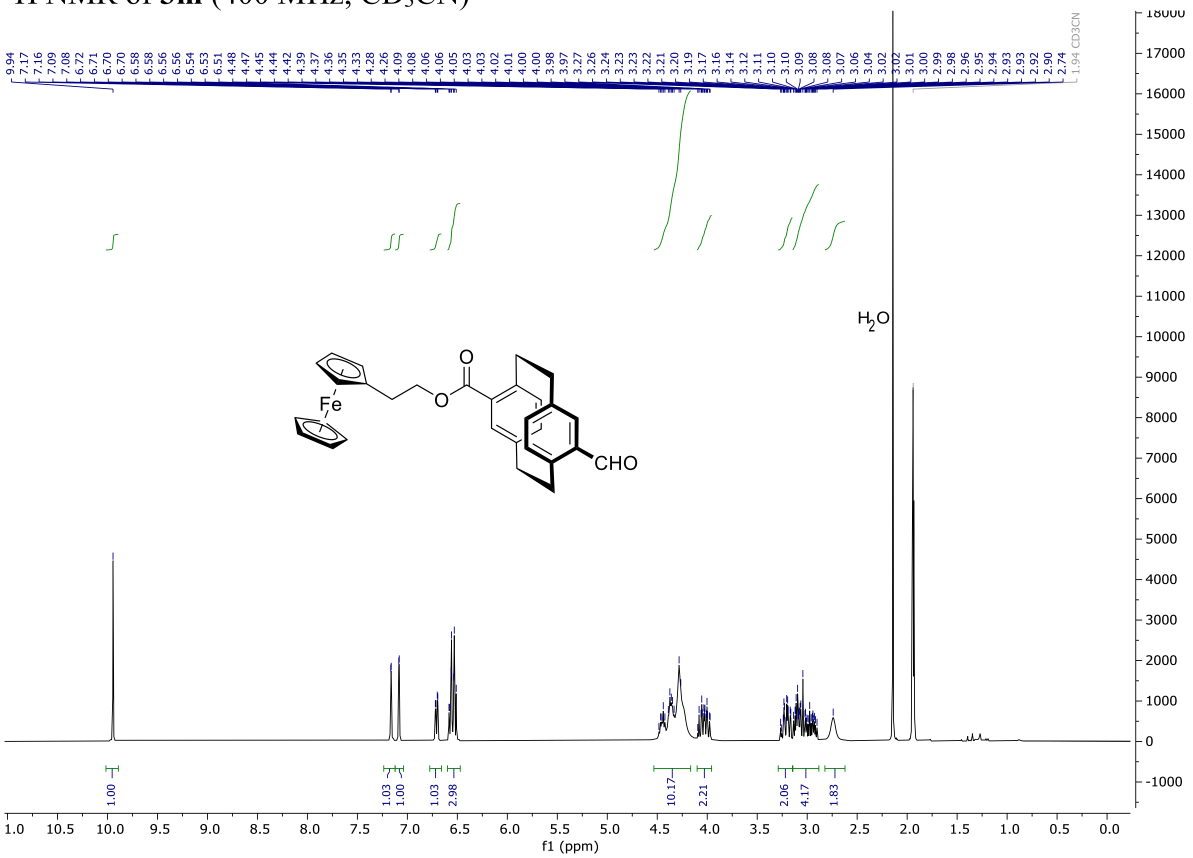

$^{13}C\{^1H\}$ NMR of **3m** (101 MHz, CD_3CN)

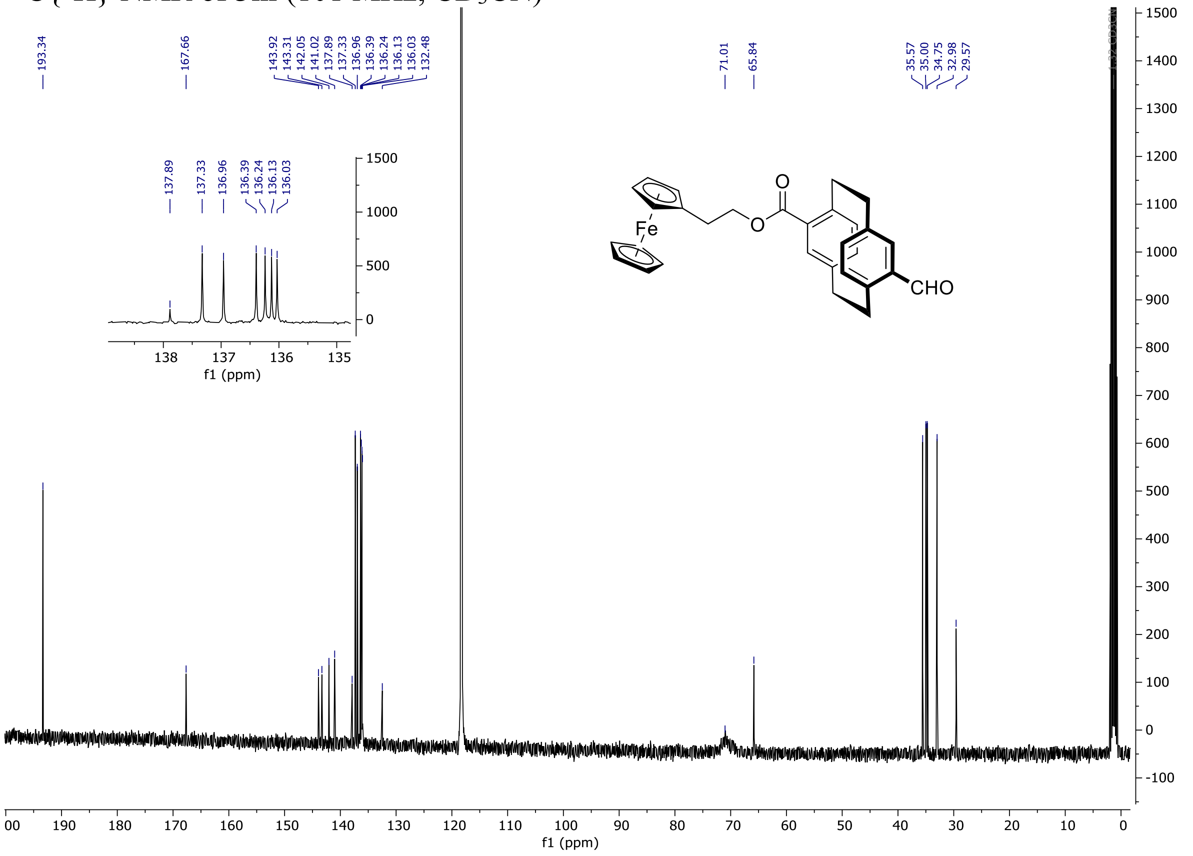

(R_p)-2-(1*H*-indol-3-yl)ethyl 4^3-formyl-1,4(1,4)-dibenzenacyclohexaphane-1^2-carboxylate (3n)

^{1}H NMR of **3n** (400 MHz, $CDCl_3$)

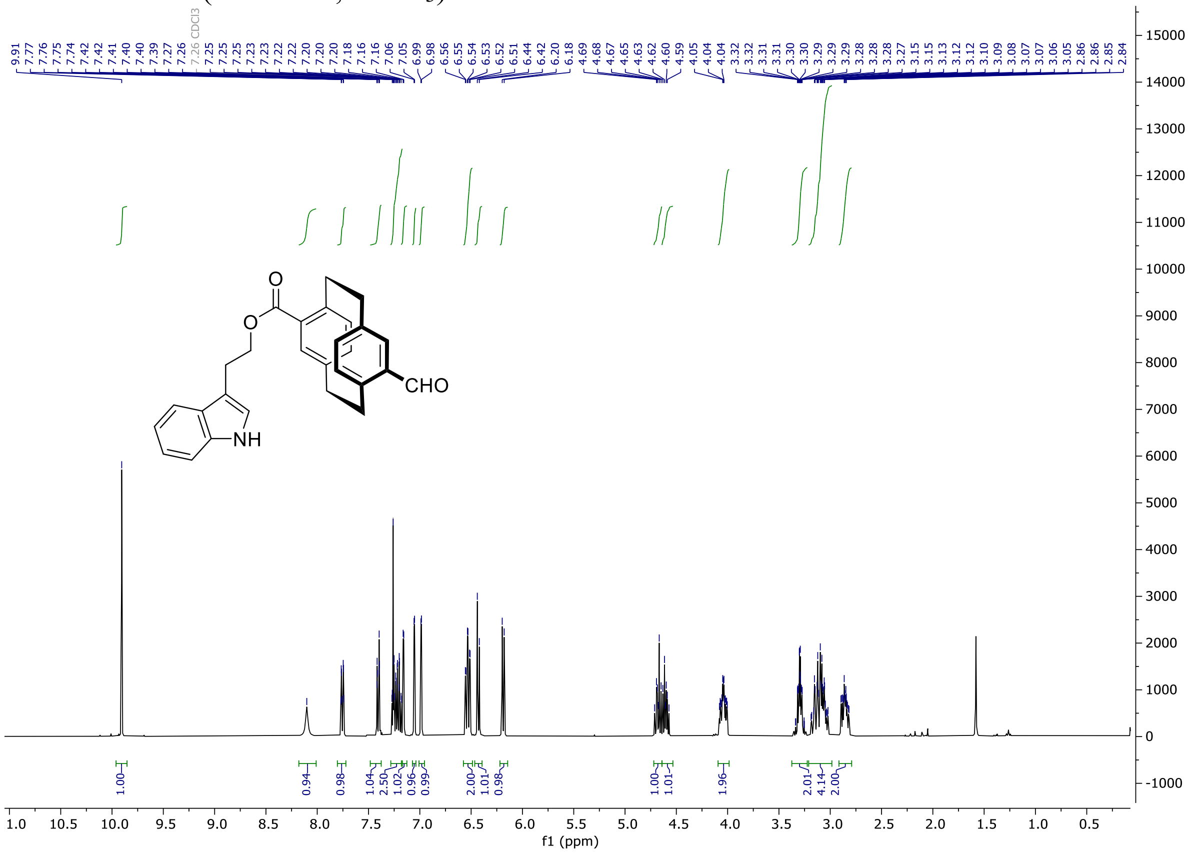

$^{13}C\{^1H\}$ NMR of **3n** (101 MHz, $CDCl_3$)

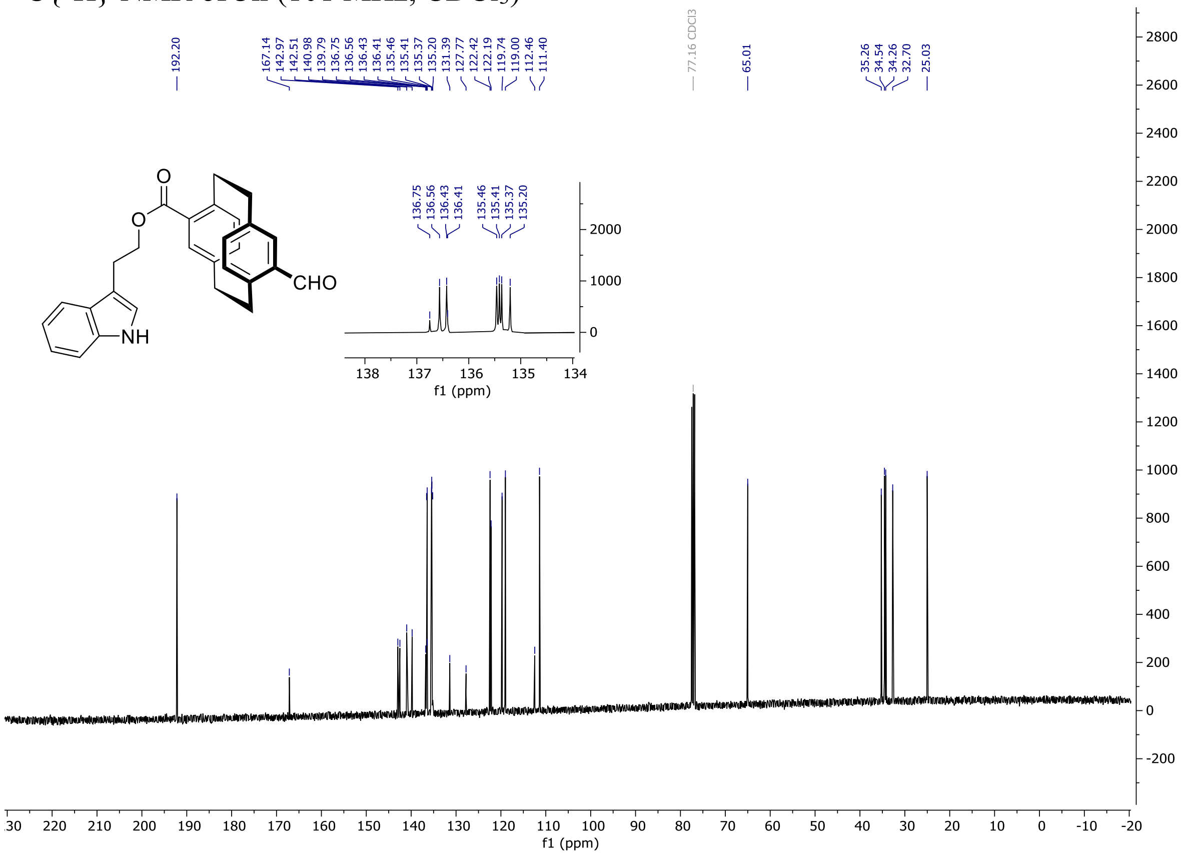

(R_p)-2-(1-(4-Chlorobenzoyl)-6-methoxy-2-methyl-1*H*-indol-3-yl)ethyl 4^3-formyl-1,4(1,4)-dibenzenacyclohexaphane-1^2-carboxylate (3o)

^{1}H NMR of **3o** (400 MHz, $CDCl_3$)

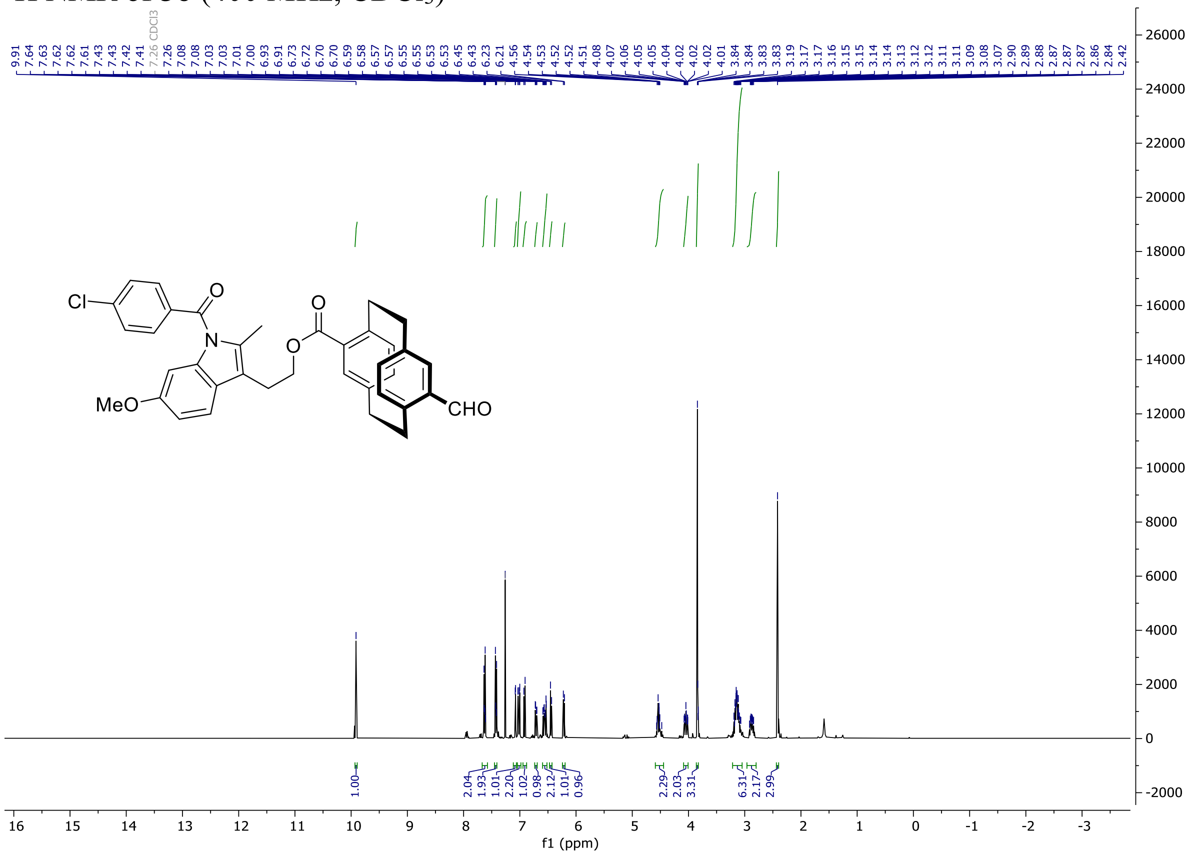

$^{13}C\{^1H\}$ NMR of **3o** (101 MHz, $CDCl_3$)

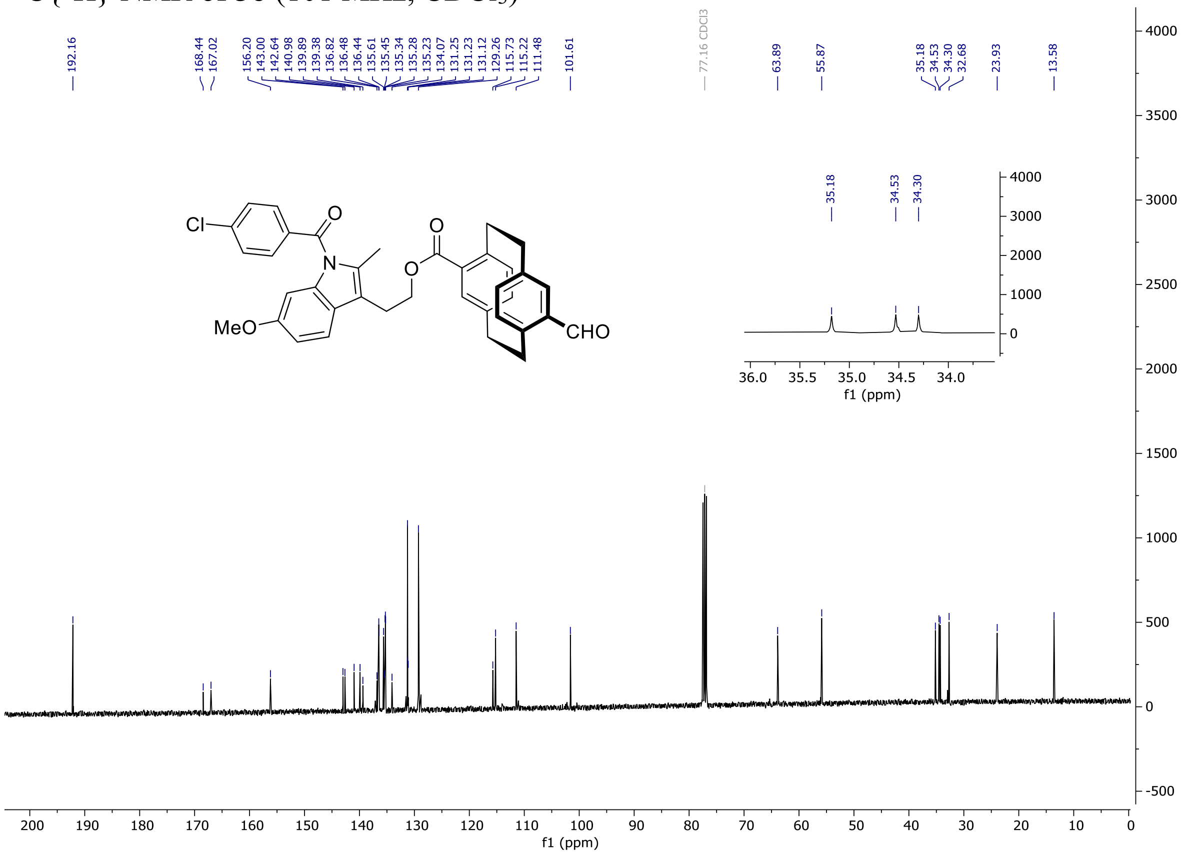

(R_p)-2-(4-Methylthiazol-5-yl)ethyl 4³-formyl-1,4(1,4)-dibenzenacyclohexaphane-1²-carboxylate (3p)

^{1}H NMR of **3p** (400 MHz, CDCl$_3$)

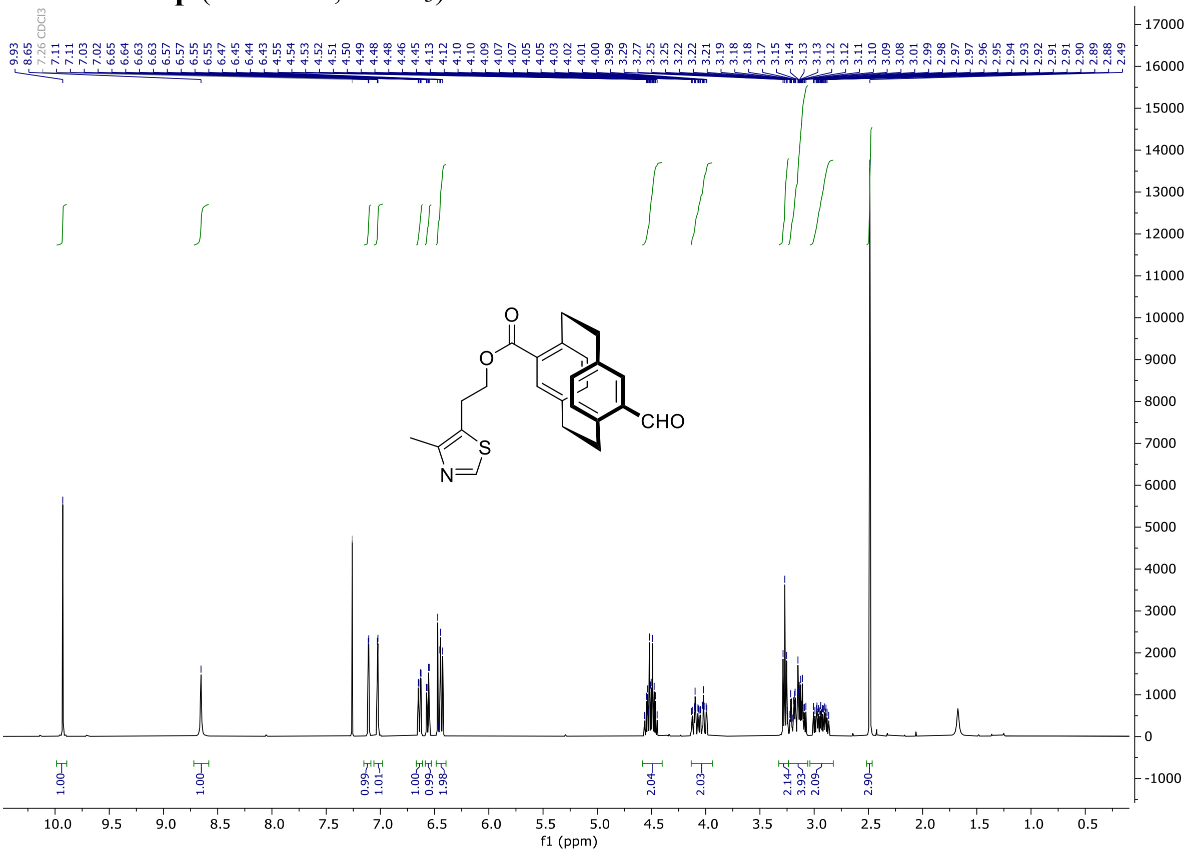

^{13}C{^{1}H} NMR of **3p** (101 MHz, CDCl$_3$)

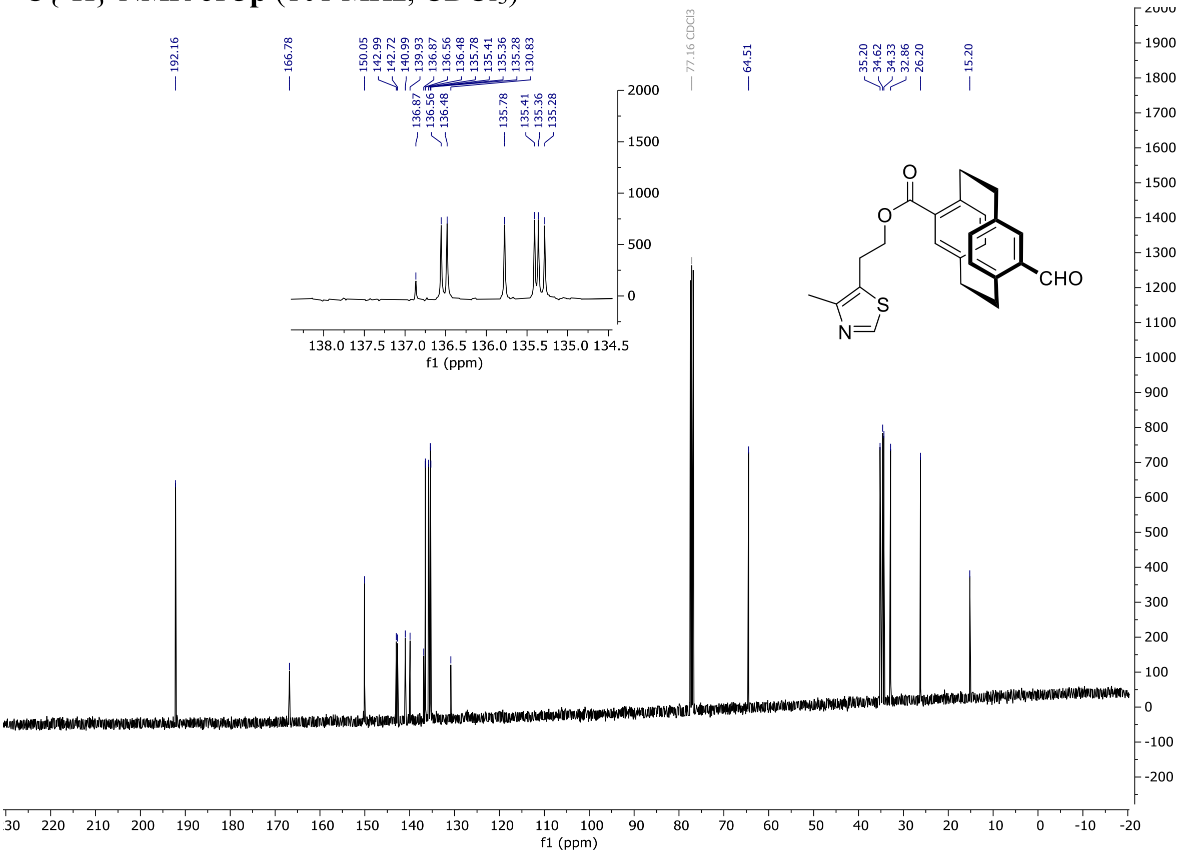

(R_p)-*tert*-Butyl (*S*)-2-(((4^3-formyl-1,4(1,4)-dibenzenacyclohexaphane-1^2-carbonyl)oxy)methyl)pyrrolidine-1-carboxylate (3q)

^{1}H NMR of **3q** (400 MHz, CDCl$_3$)

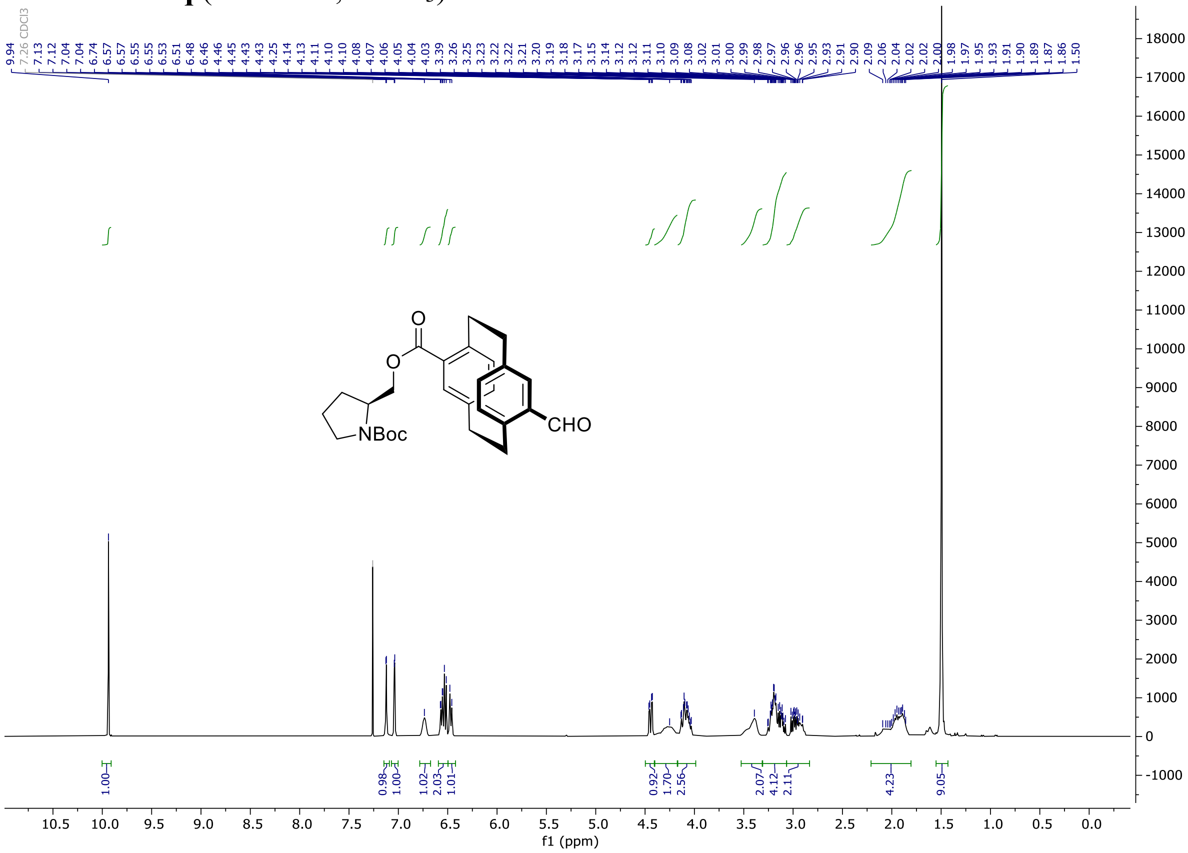

^{13}C{^{1}H} NMR of **3q** (101 MHz, CDCl$_3$)

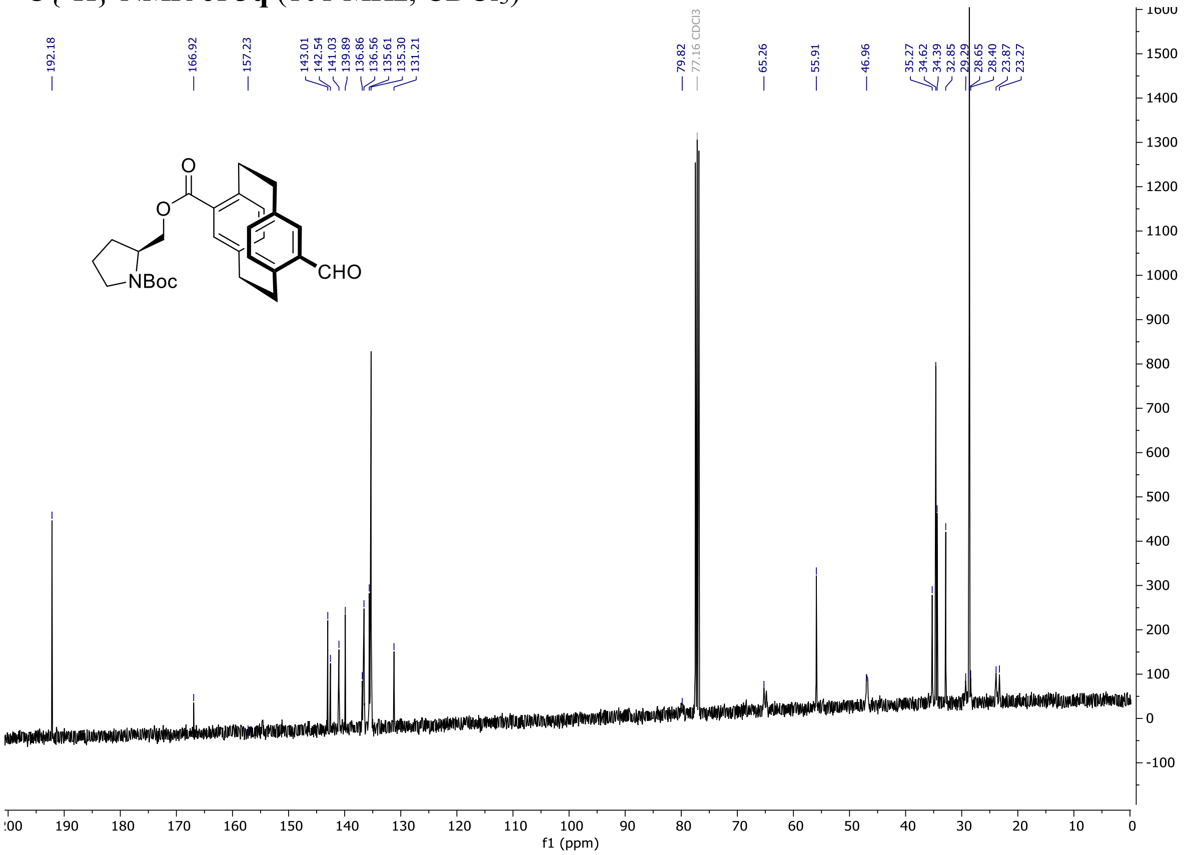

(R_p)-(4R)-4-((3R,7R,8R,9S,10S,13R,14S,17R)-3,7-dihydroxy-10,13-dimethylhexadecahydro-1H-cyclopenta[a]phenanthren-17-yl)pentyl 4[3]-formyl-1,4(1,4)-dibenzenacyclohexaphane-1[2]-carboxylate (3r)

^{1}H NMR of **3r** (400 MHz, $CDCl_3$)

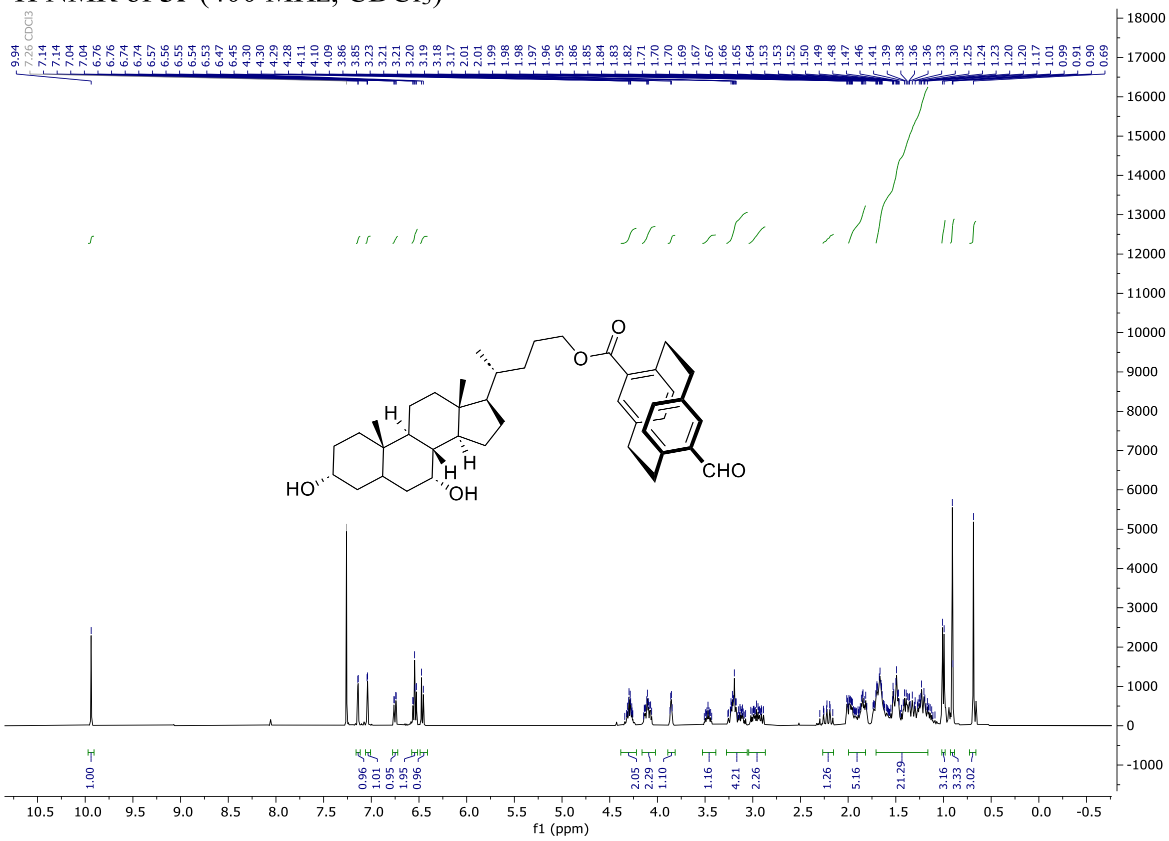

^{13}C{^{1}H} NMR of **3r** (101 MHz, $CDCl_3$)

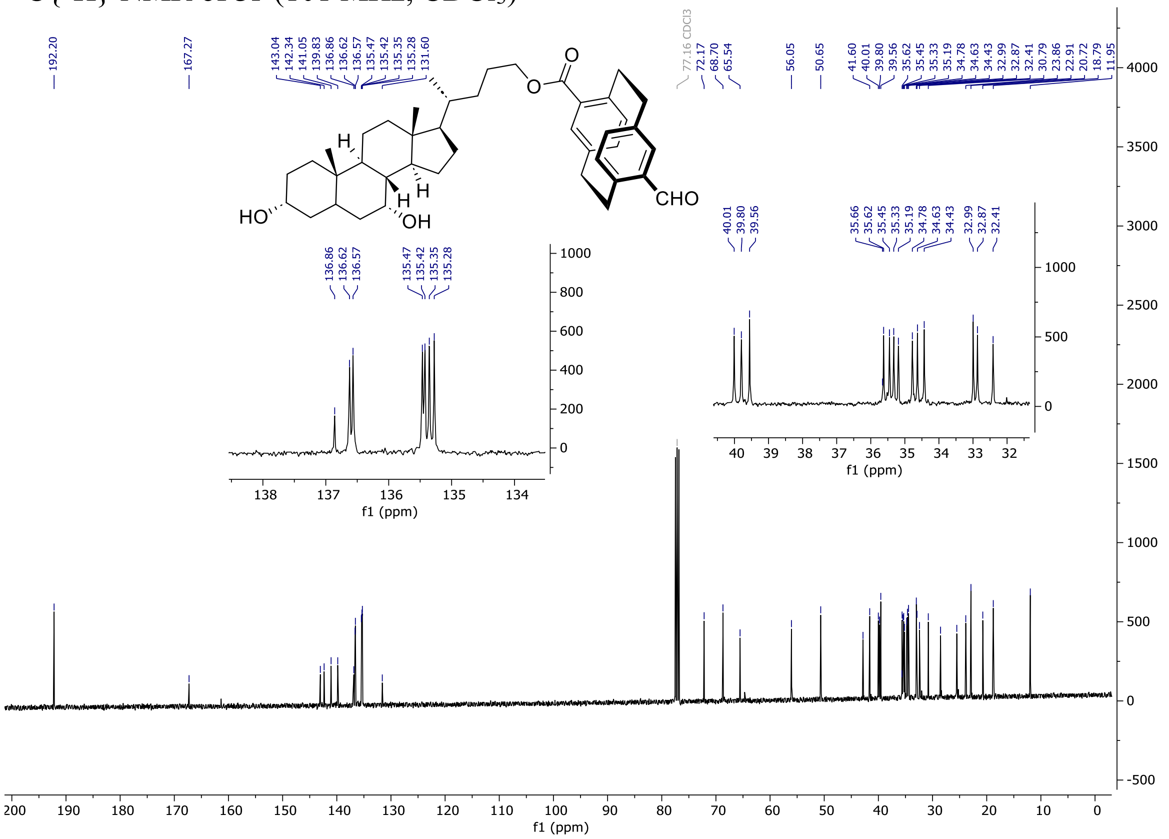

(R_p)-(4R)-4-((3R,7R,8R,9S,10S,13R,14S,17R)-3,7-*bis*(Methoxymethoxy)-10,13-dimethylhexadecahydro-1H-cyclopenta[a]phenanthren-17-yl)pentyl 4^3-formyl-1,4(1,4)-dibenzenacyclohexaphane-1^2-carboxylate (3s)

^{1}H NMR of **3s** (400 MHz, $CDCl_3$)

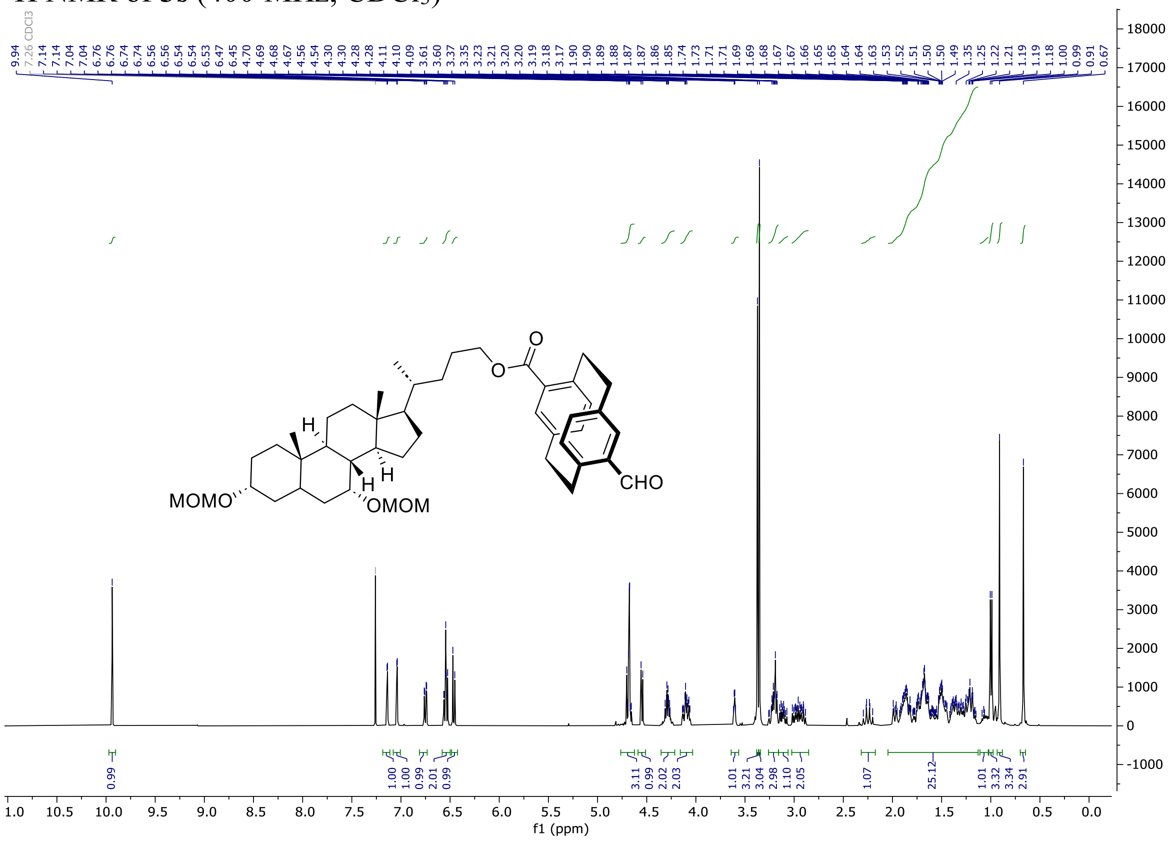

$^{13}C\{^1H\}$ NMR of **3s** (101 MHz, $CDCl_3$)

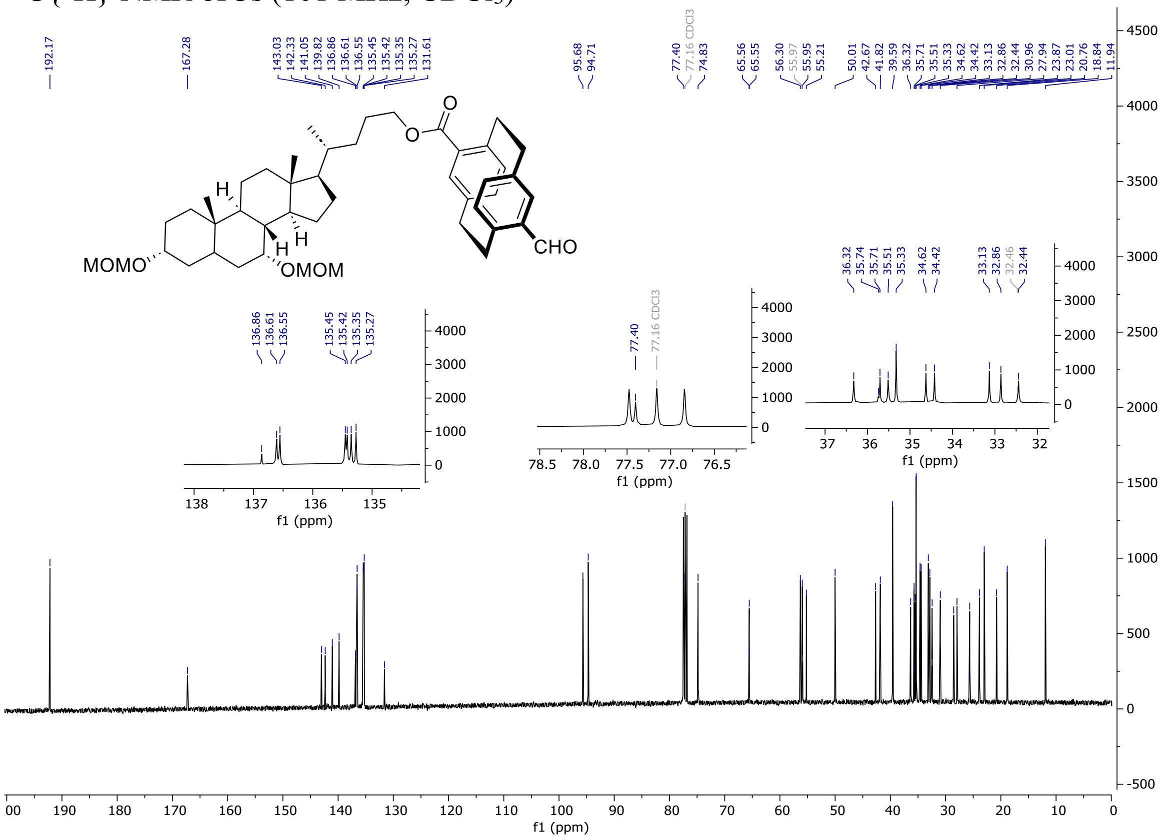

(R_p)-((2R,3R,4S,5R,6S)-3,4,5,6-tetramethoxytetrahydro-2H-pyran-2-yl)methyl 4³-formyl-1,4(1,4)-dibenzenacyclohexaphane-1²-carboxylate (3t)

^{1}H NMR of **3s** (400 MHz, CDCl$_3$)

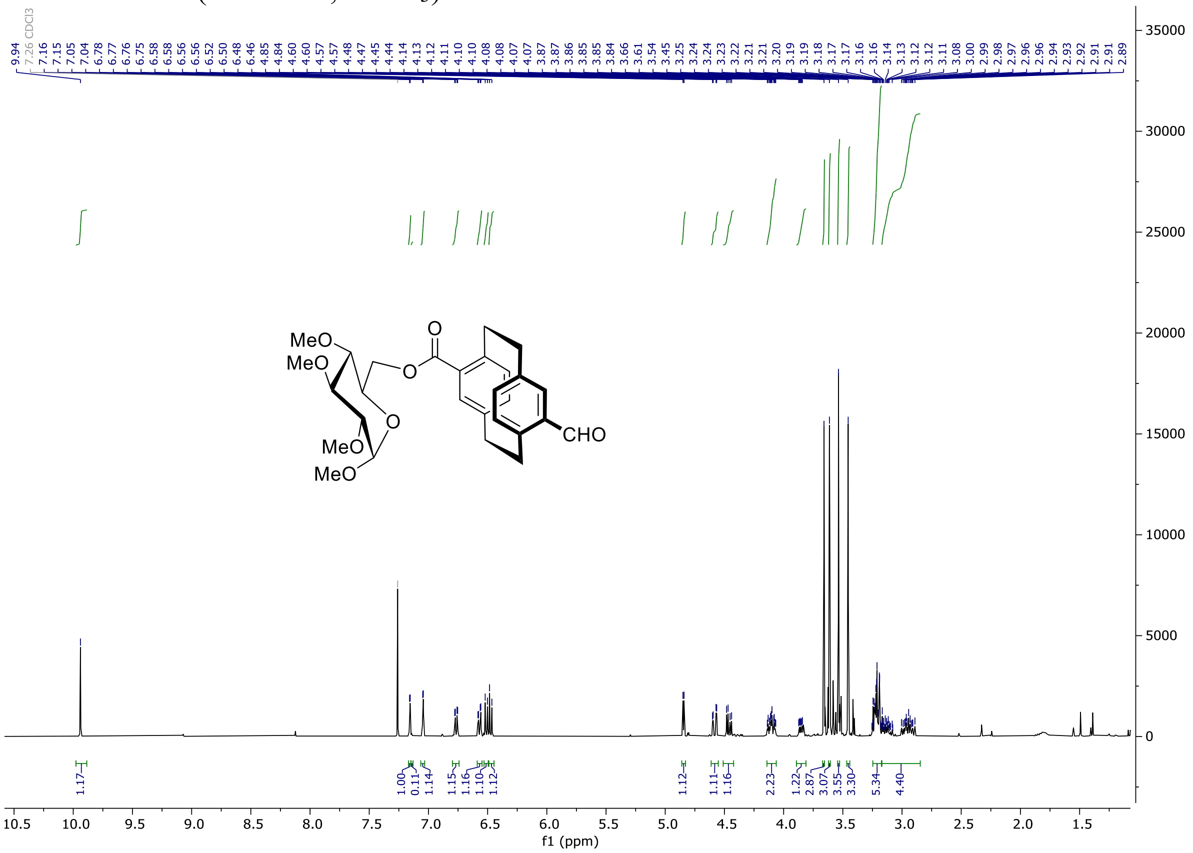

^{13}C{^{1}H} NMR of **3t** (101 MHz, CDCl$_3$)

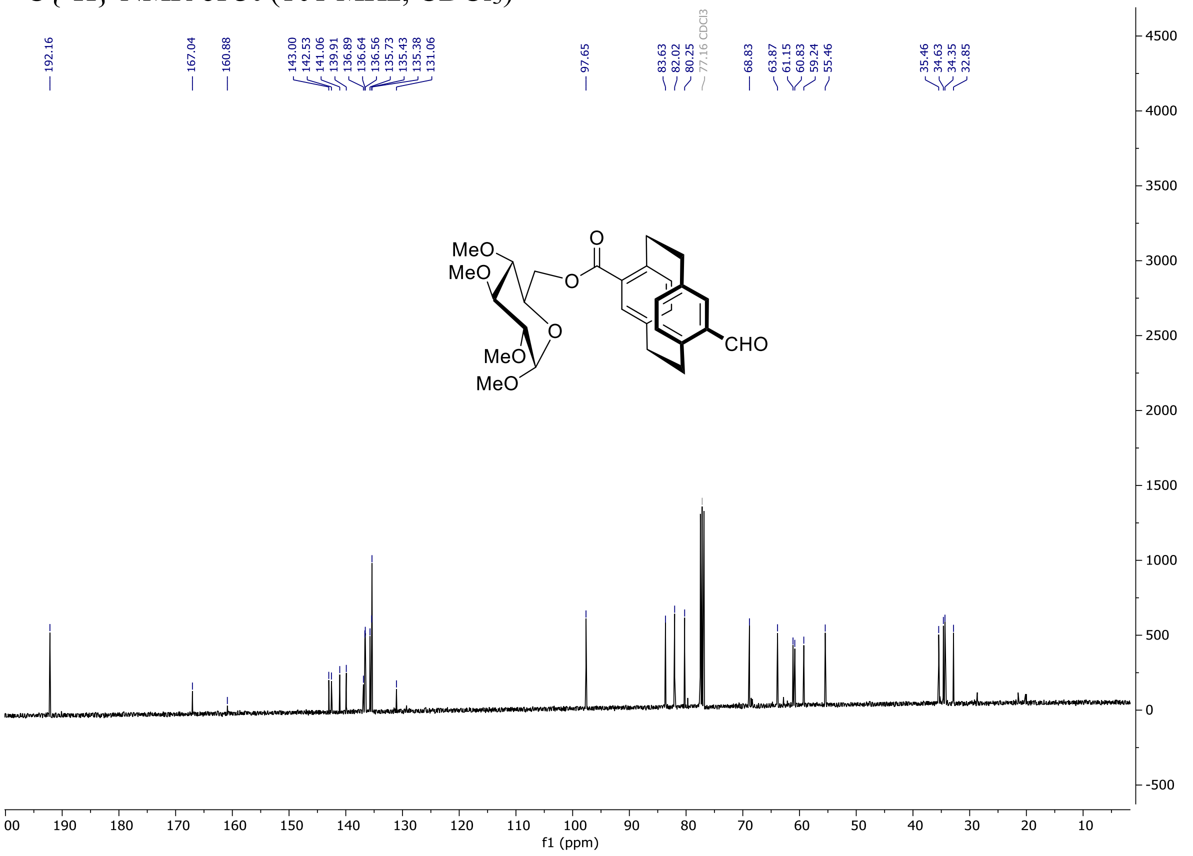

(R_p)-5-((3a*S*,4*S*,6a*R*)-1,3-dibenzyl-2-oxohexahydro-1*H*-thieno[3,4-d]imidazol-4-yl)pentyl 4³-formyl-1,4(1,4)-dibenzenacyclohexaphane-1²-carboxylate (3t)

^{1}H NMR of **3u** (400 MHz, $CDCl_3$)

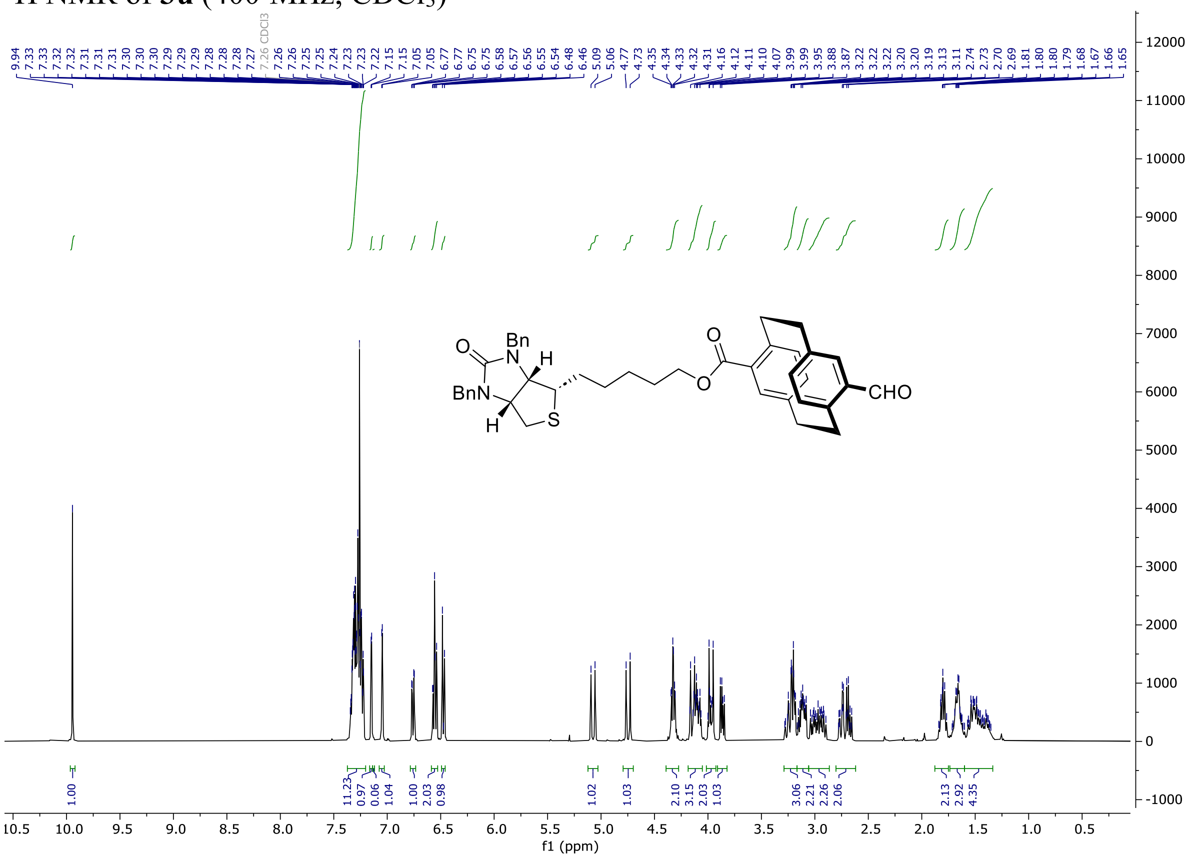

^{13}C{^{1}H} NMR of **3u** (101 MHz, $CDCl_3$)

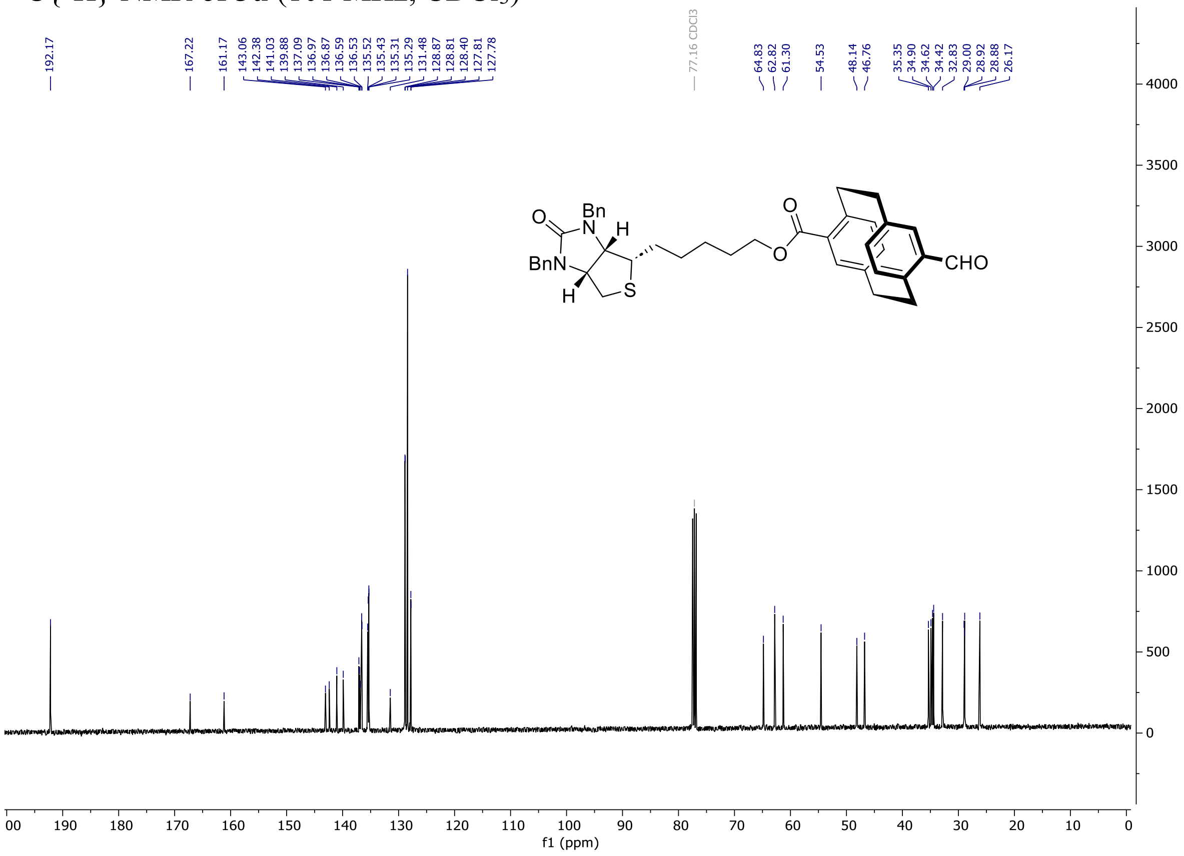

(R_p)-(S)-3,7-Dimethyloct-6-en-1-yl 4[3]-formyl-1,4(1,4)-dibenzenacyclohexaphane-1[2]-carboxylate (3u)

^{1}H NMR of **3v** (400 MHz, $CDCl_3$)

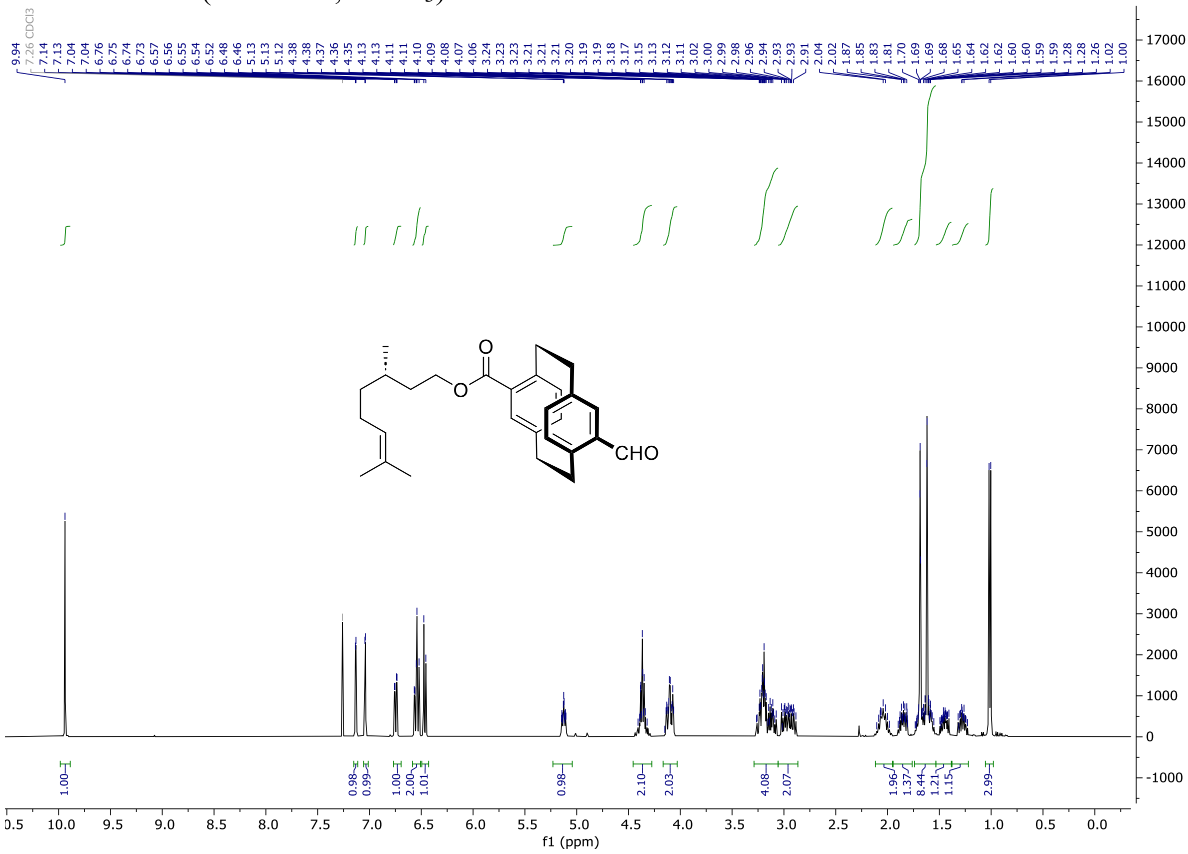

$^{13}C\{^1H\}$ NMR of **3v** (101 MHz, $CDCl_3$)

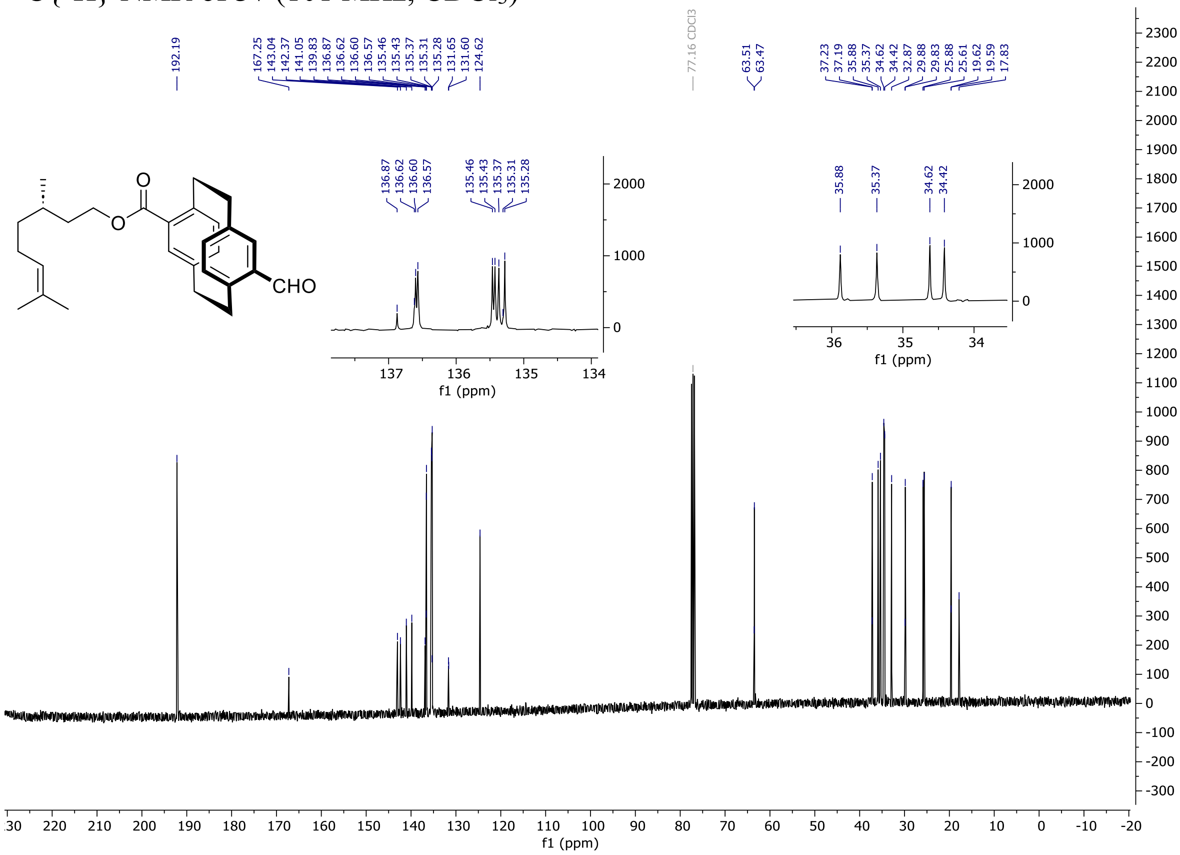

(R_p)-*S*-Ethyl 4^3-formyl-1,4(1,4)-dibenzenacyclohexaphane-1^2-carbothioate (3v)

^{1}H NMR of **3w** (400 MHz, $CDCl_3$)

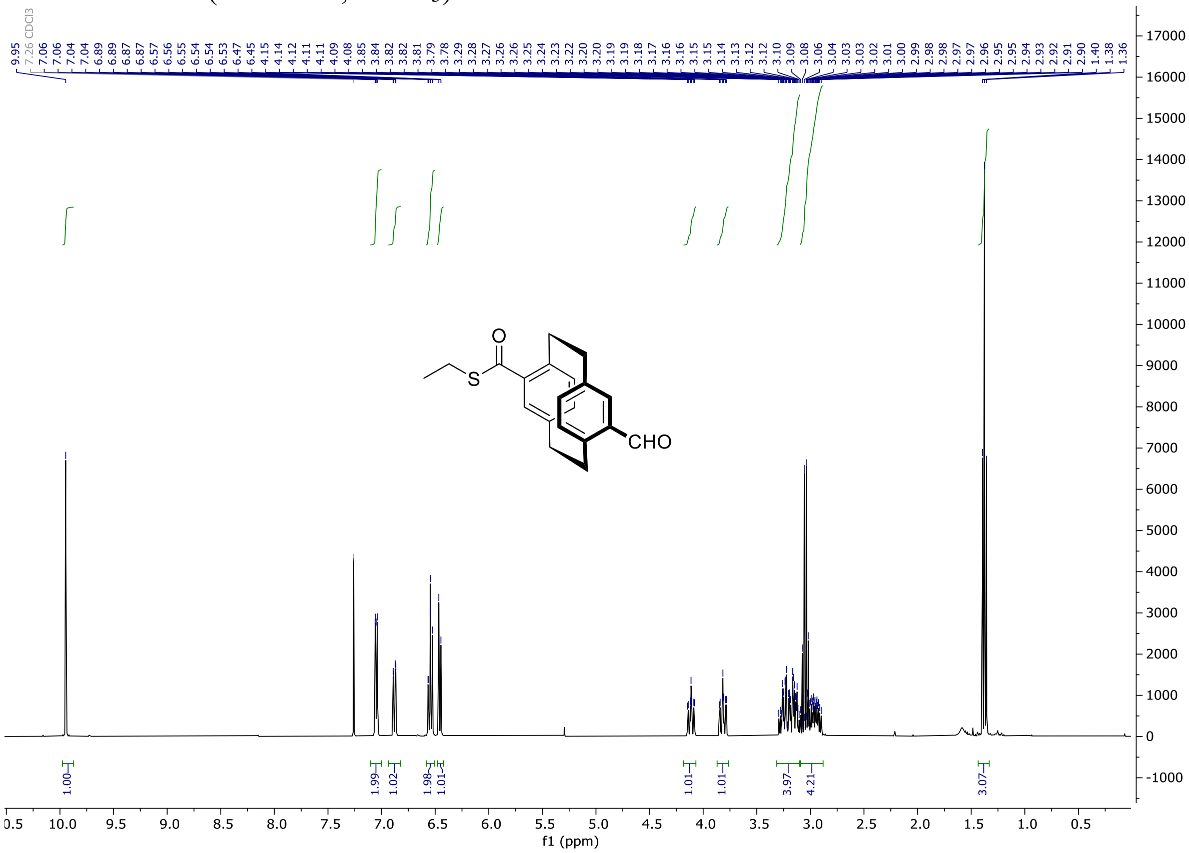

^{13}C{^{1}H} NMR of **3w** (101 MHz, $CDCl_3$)

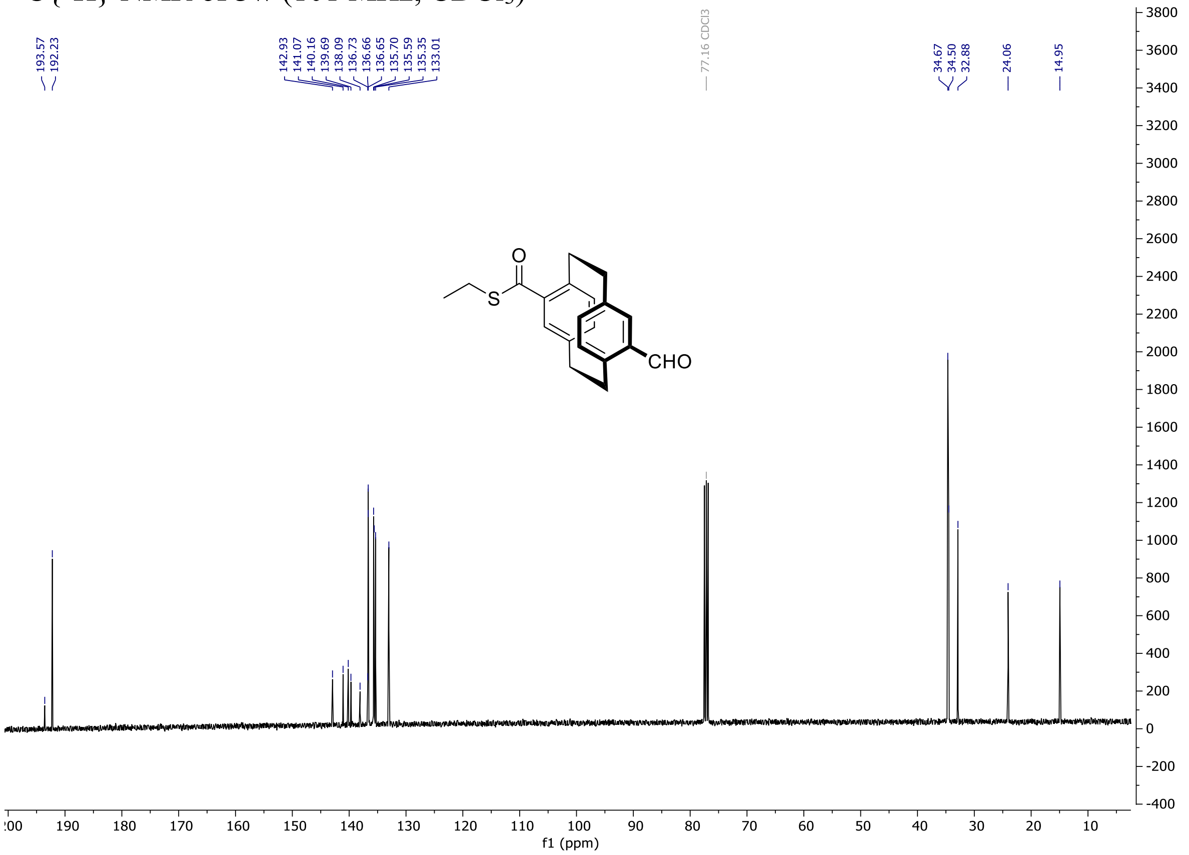

(R_p)-*S*-Phenethyl 4^3-formyl-1,4(1,4)-dibenzenacyclohexaphane-1^2-carbothioate (3w)

^{1}H NMR of **3x** (400 MHz, $CDCl_3$)

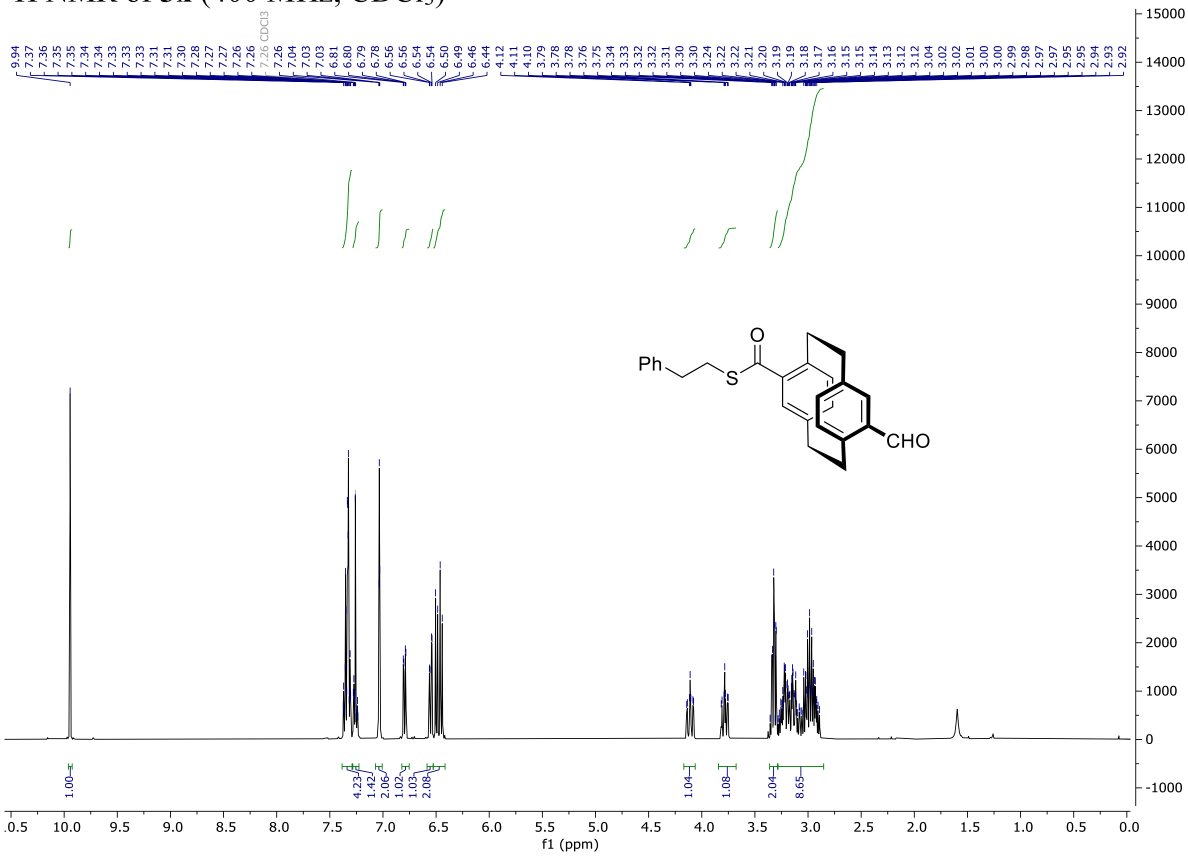

^{13}C{^{1}H} NMR of **3x** (101 MHz, $CDCl_3$)

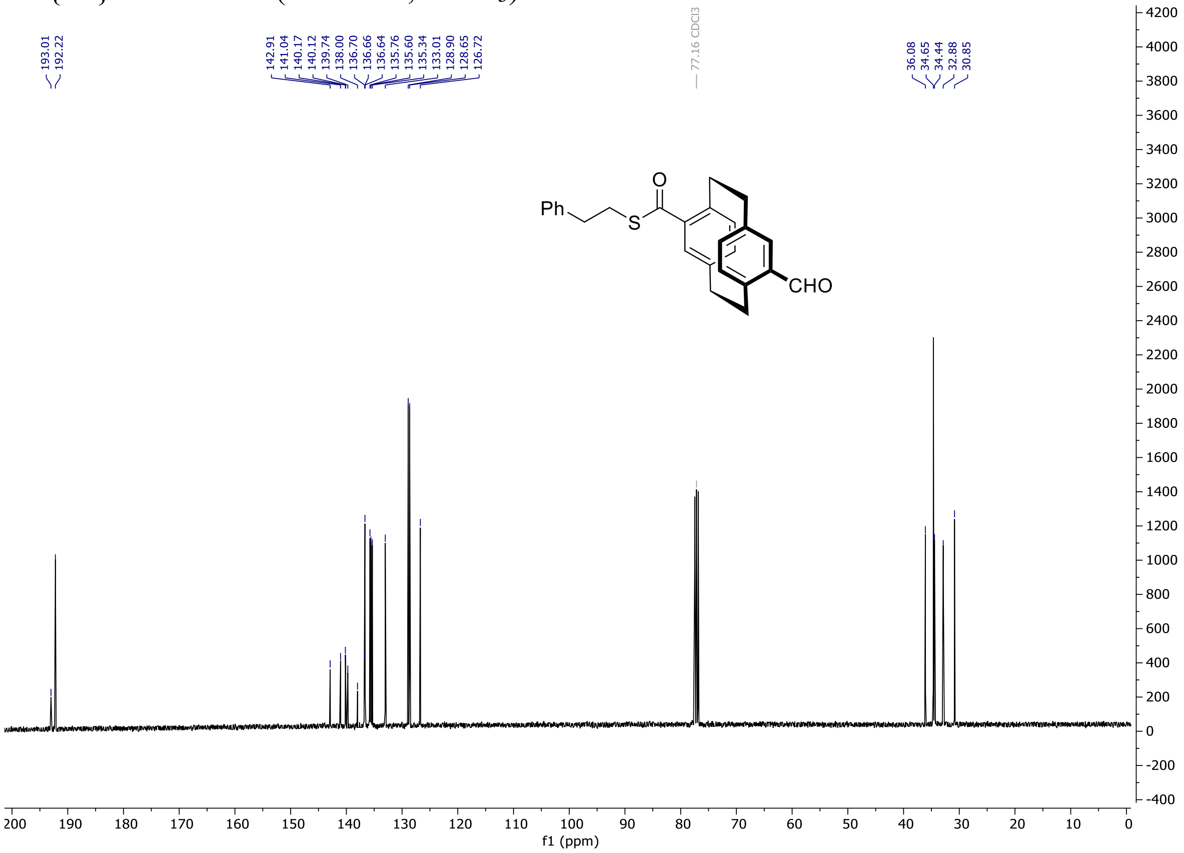

(R_p)-Methyl 4²-formyl-1,4(1,4)-dibenzenacyclohexaphane-1²-carboxylate (5a)

^{1}H NMR of **5a** (400 MHz, $CDCl_3$)

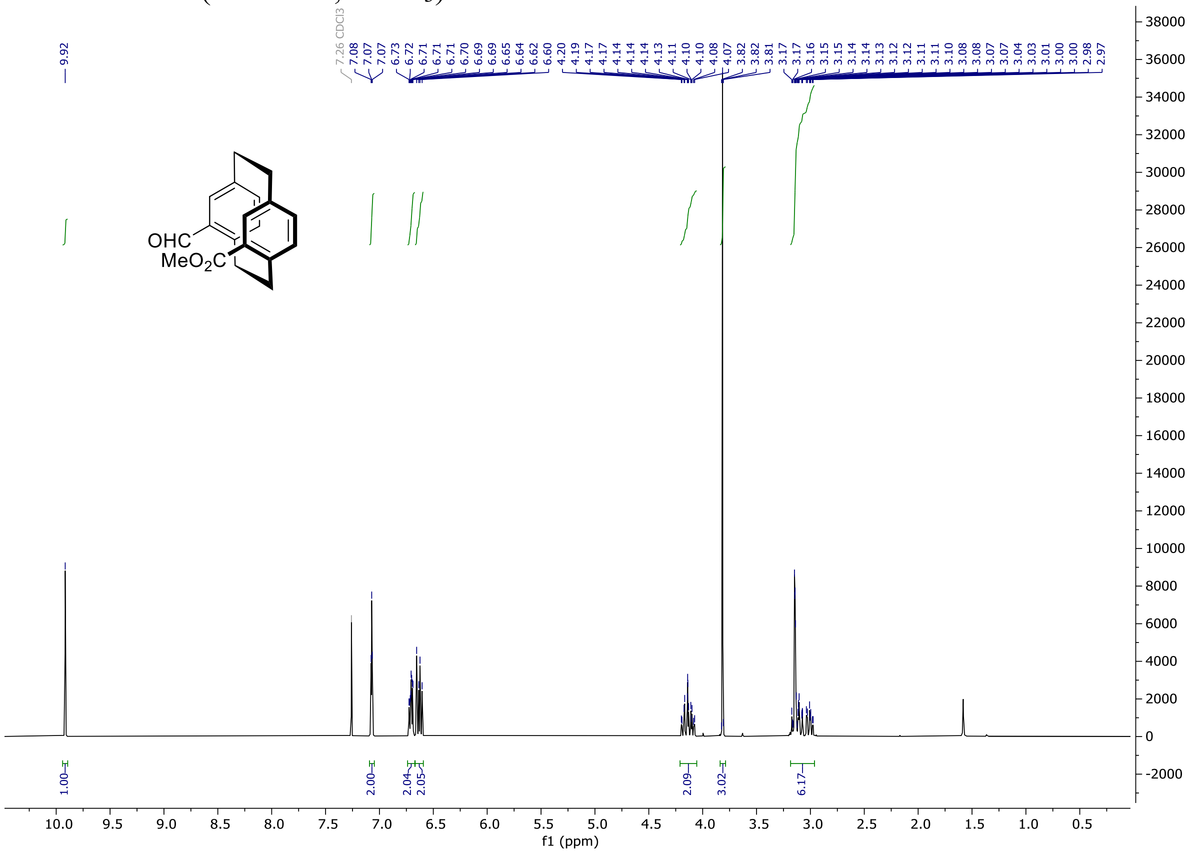

^{13}C{^{1}H} NMR of **5a** (101 MHz, $CDCl_3$)

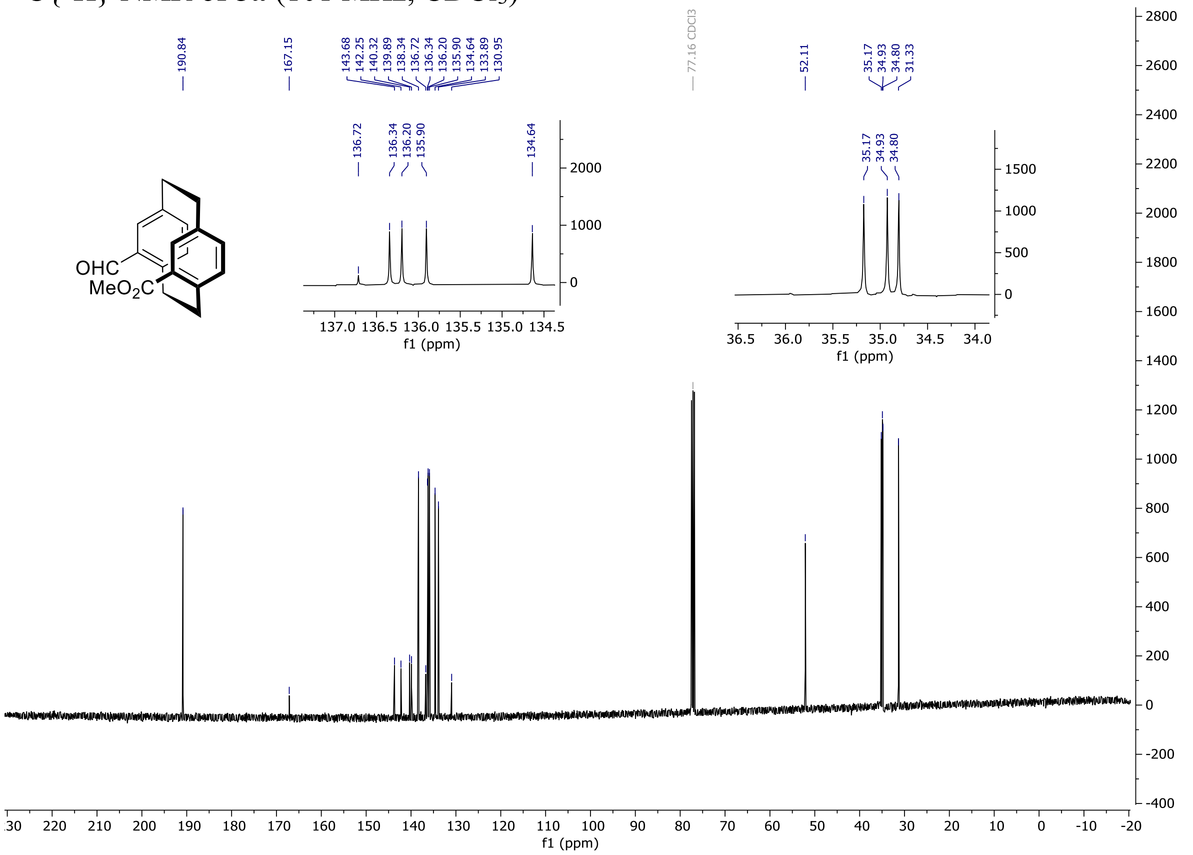

(R_p)-Ethyl 4²-formyl-1,4(1,4)-dibenzenacyclohexaphane-1²-carboxylate (5b)

^{1}H NMR of **5b** (400 MHz, $CDCl_3$)

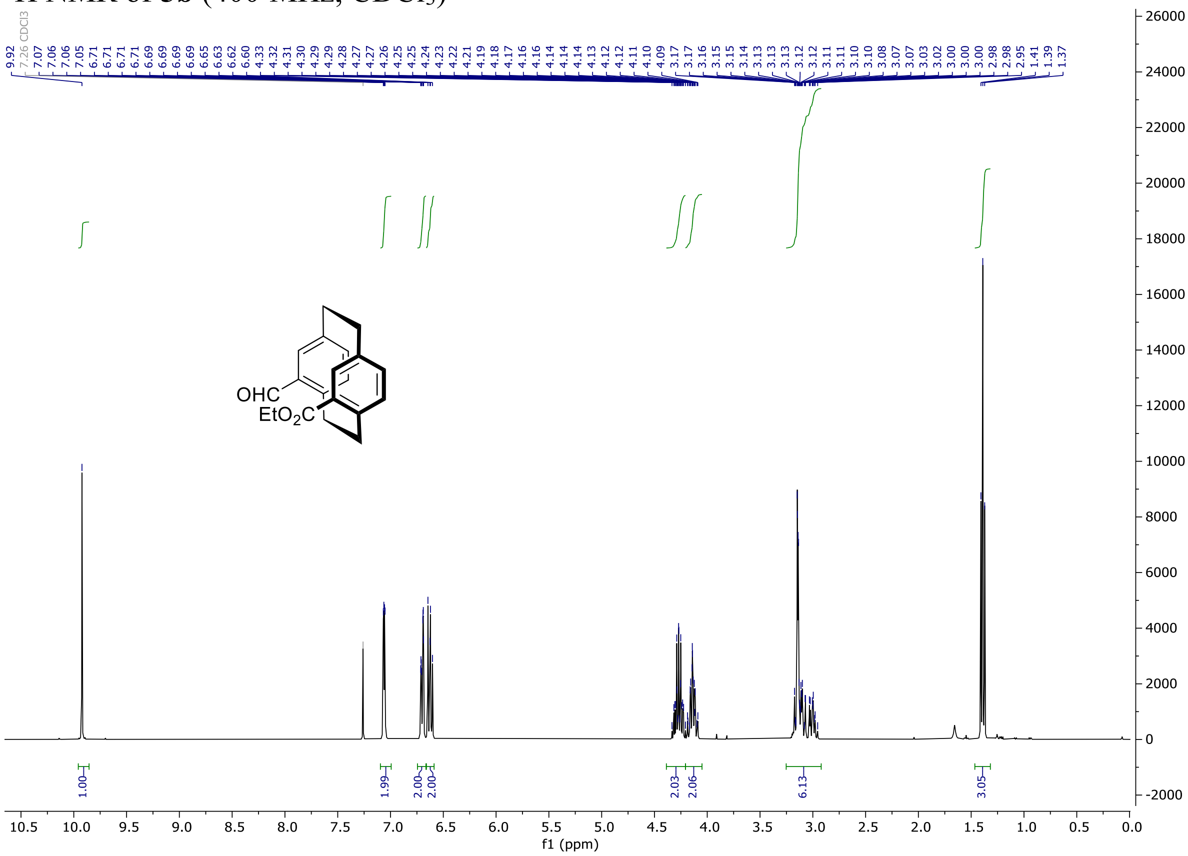

^{13}C{^{1}H} NMR of **5b** (101 MHz, $CDCl_3$)

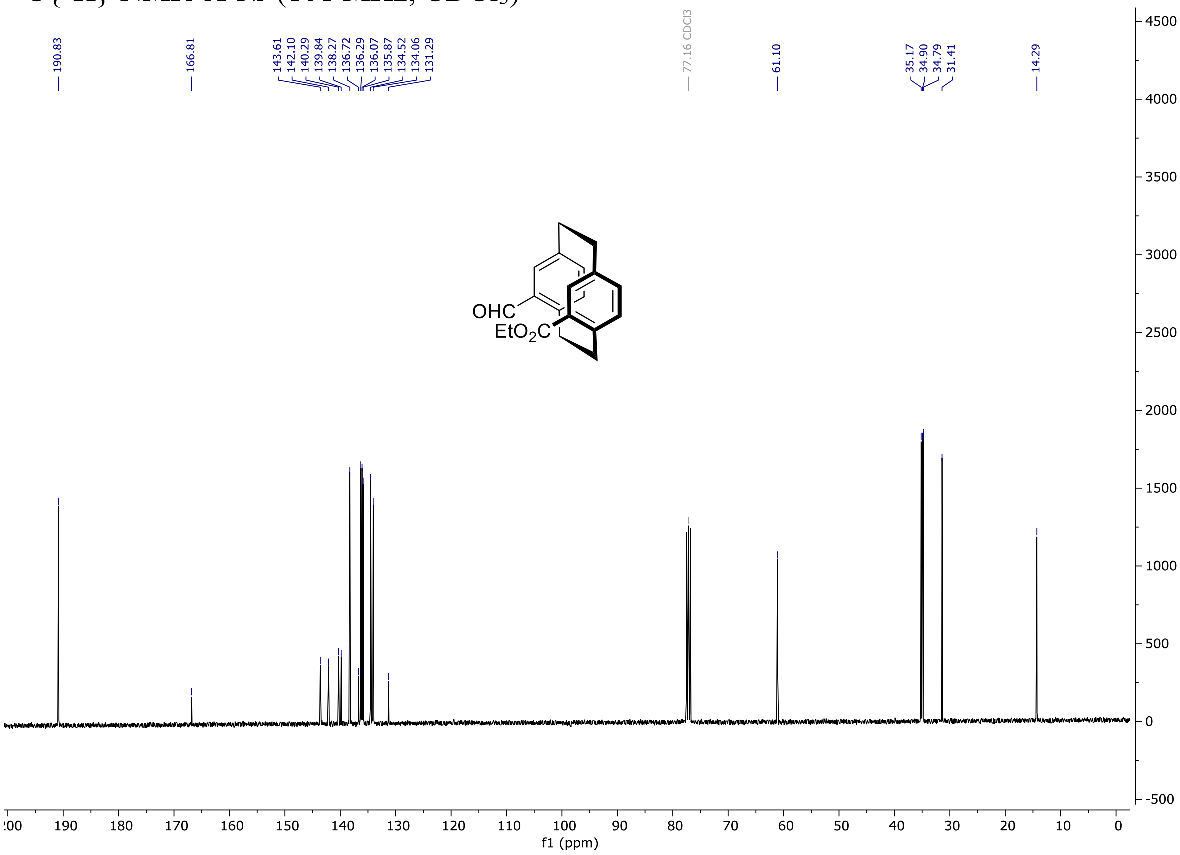

(R_p)-Isopropyl 4^2-formyl-1,4(1,4)-dibenzenacyclohexaphane-1^2-carboxylate (5c)

^{1}H NMR of **5c** (400 MHz, CDCl$_3$)

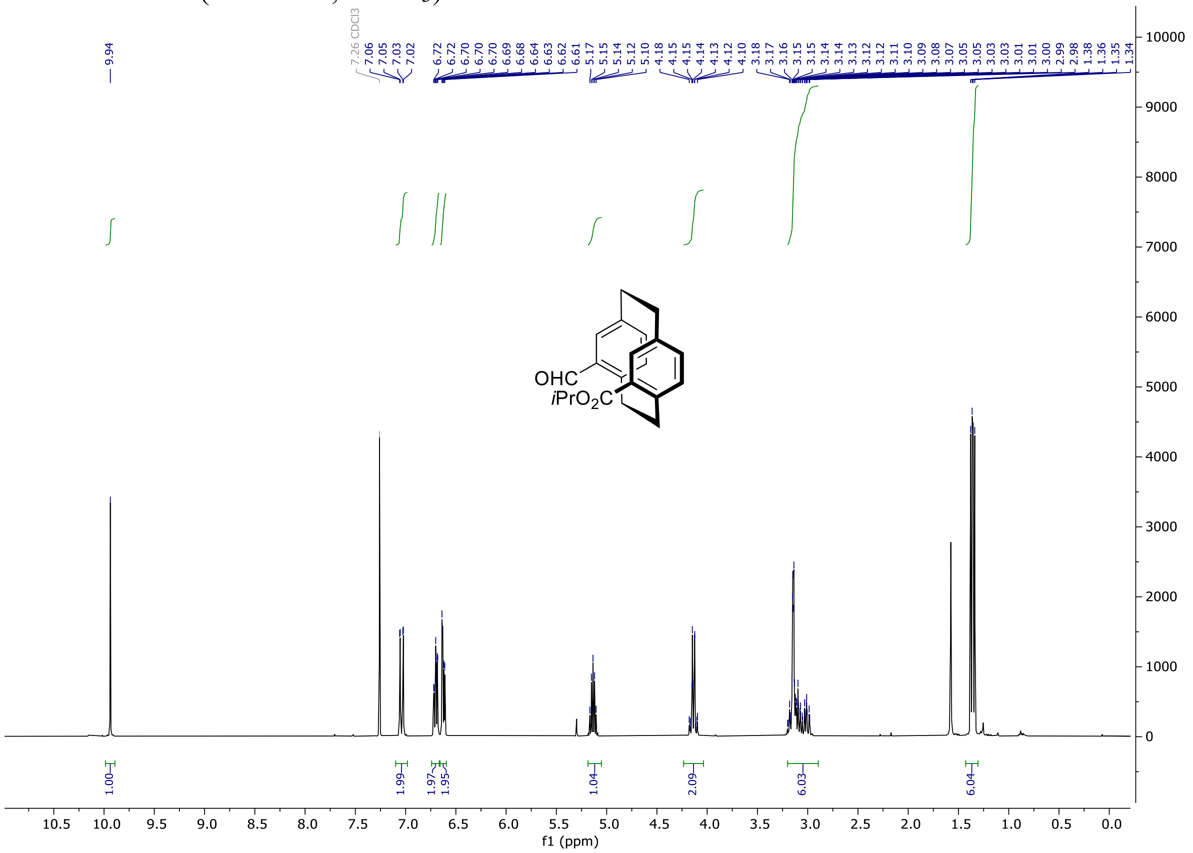

^{13}C{^{1}H} NMR of **5c** (101 MHz, CDCl$_3$)

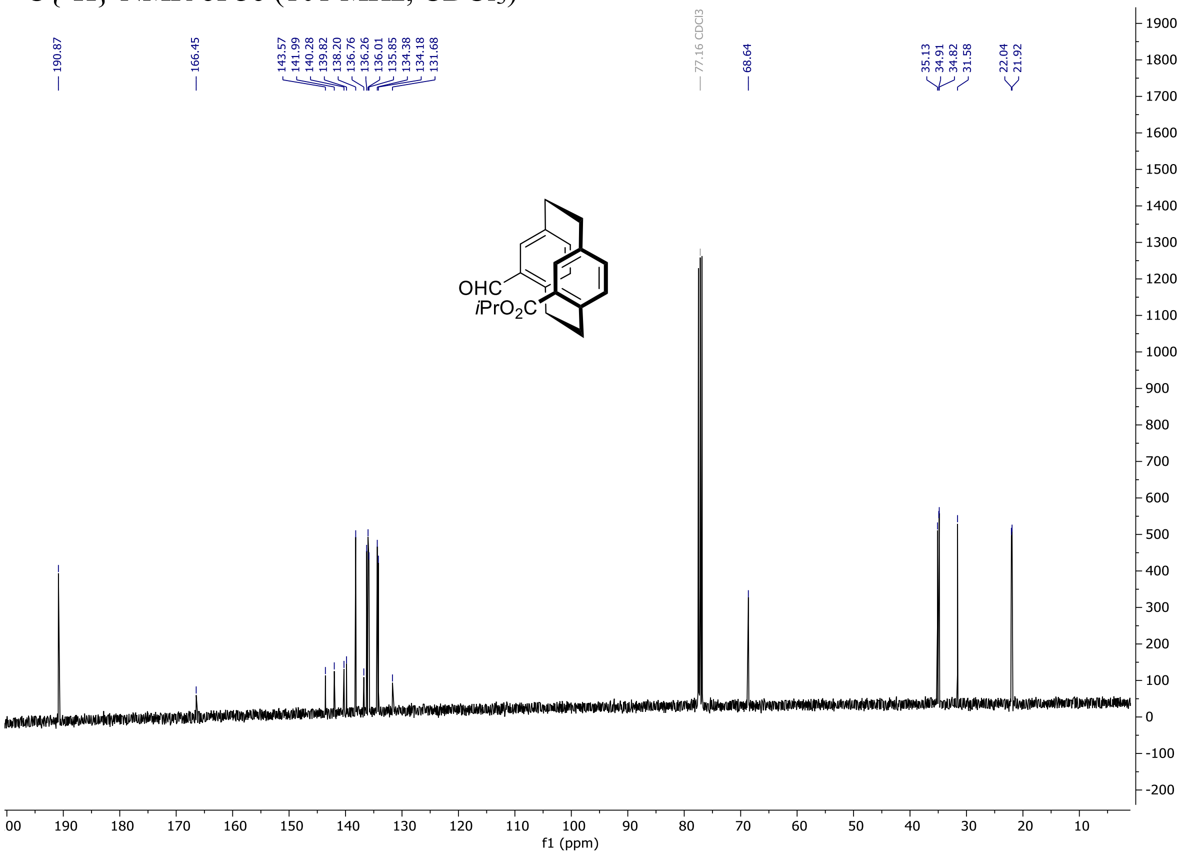

(R_p)-2-Methoxyethyl 4²-formyl-1,4(1,4)-dibenzenacyclohexaphane-1²-carboxylate (5d)

^{1}H NMR of **5d** (400 MHz, $CDCl_3$)

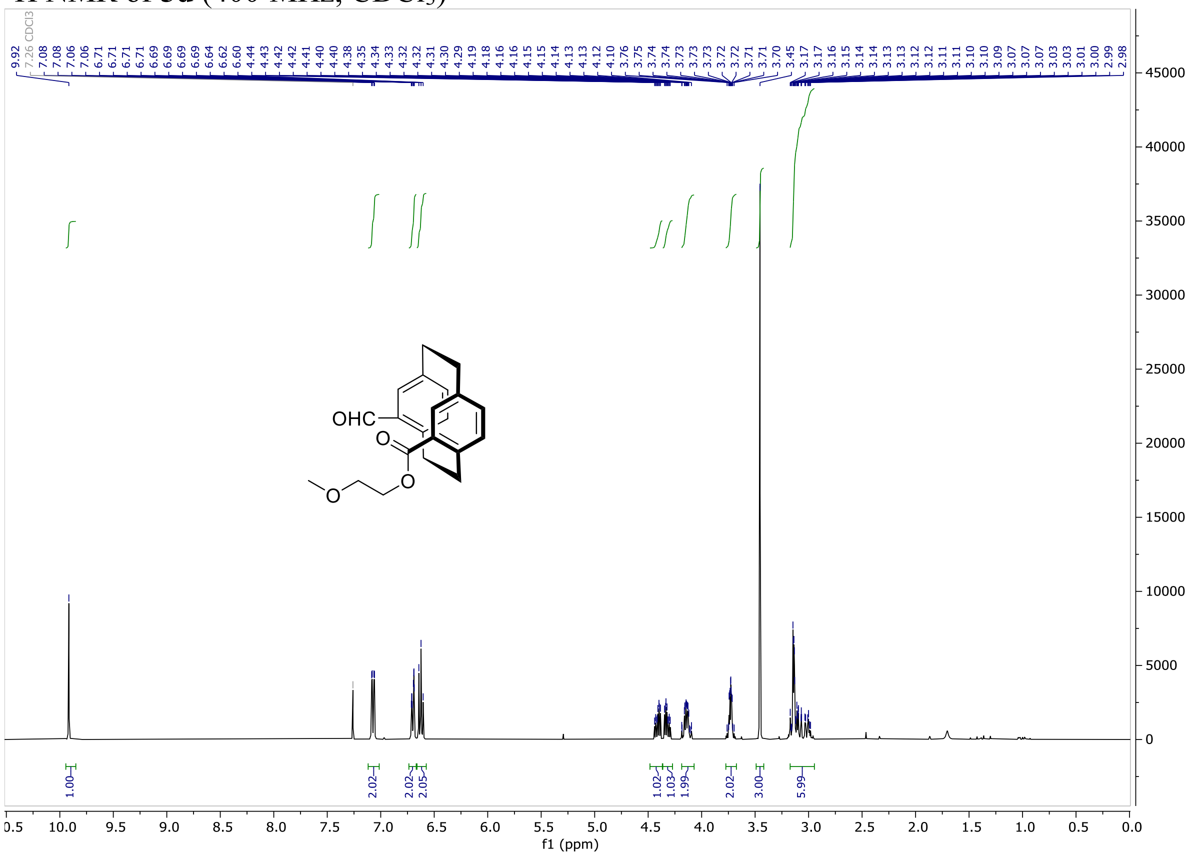

$^{13}C\{^1H\}$ NMR of **5d** (101 MHz, $CDCl_3$)

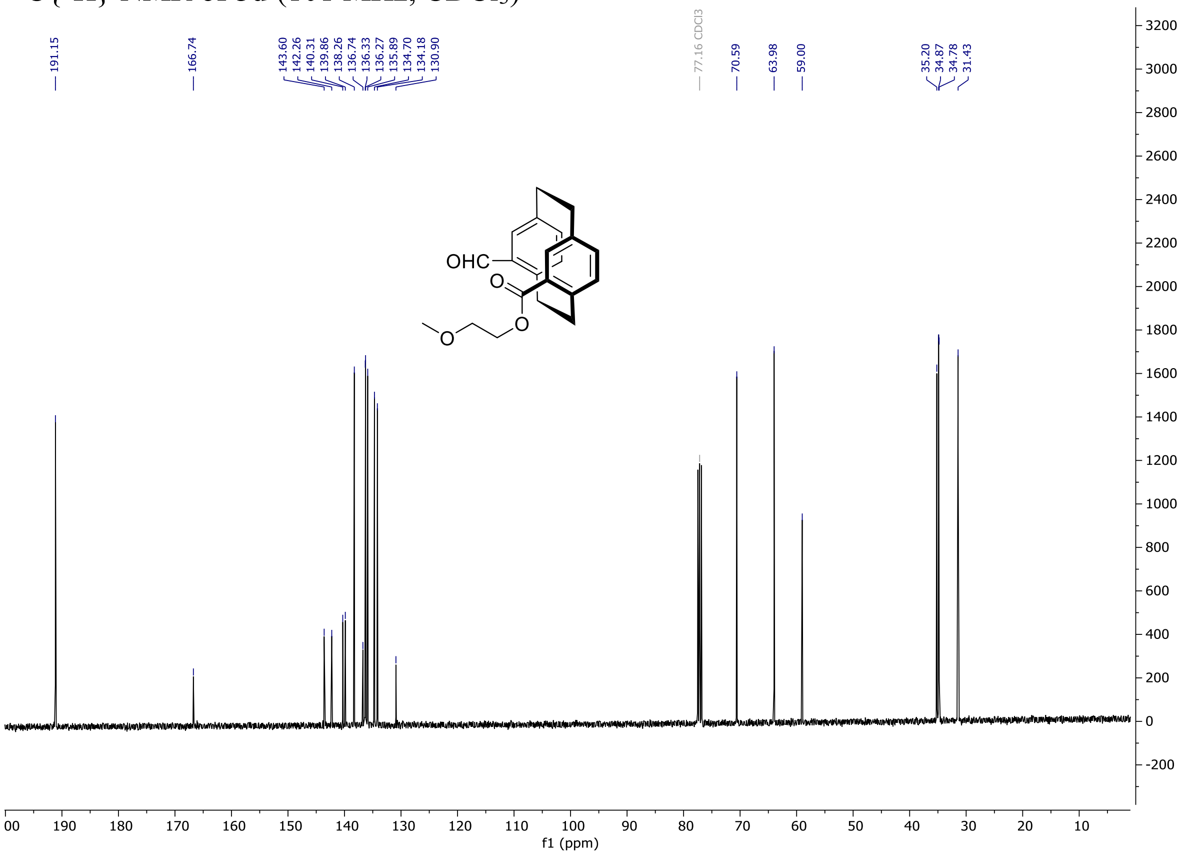

(R_p)-Pent-3-yn-1-yl 4²-formyl-1,4(1,4)-dibenzenacyclohexaphane-1²-carboxylate (5e)

^{1}H NMR of **5e** (400 MHz, $CDCl_3$)

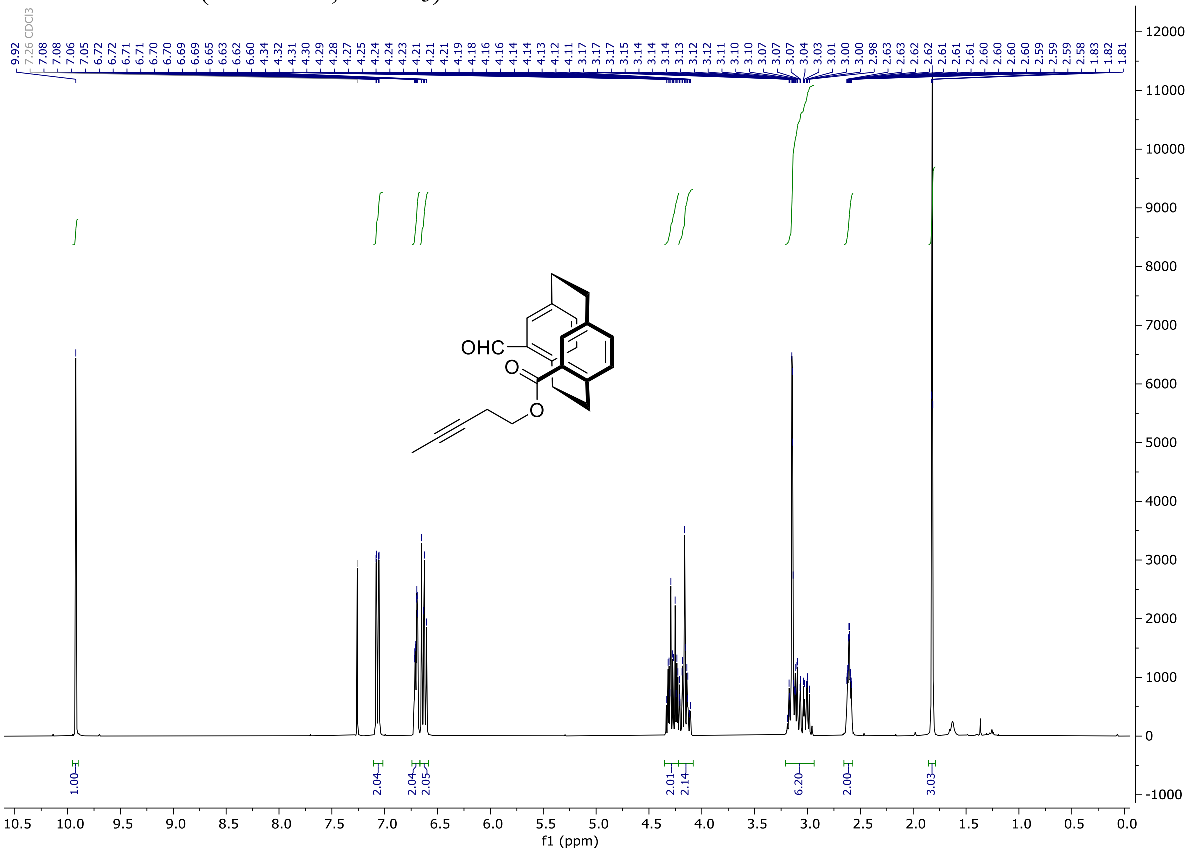

$^{13}C\{^1H\}$ NMR of **5e** (101 MHz, $CDCl_3$)

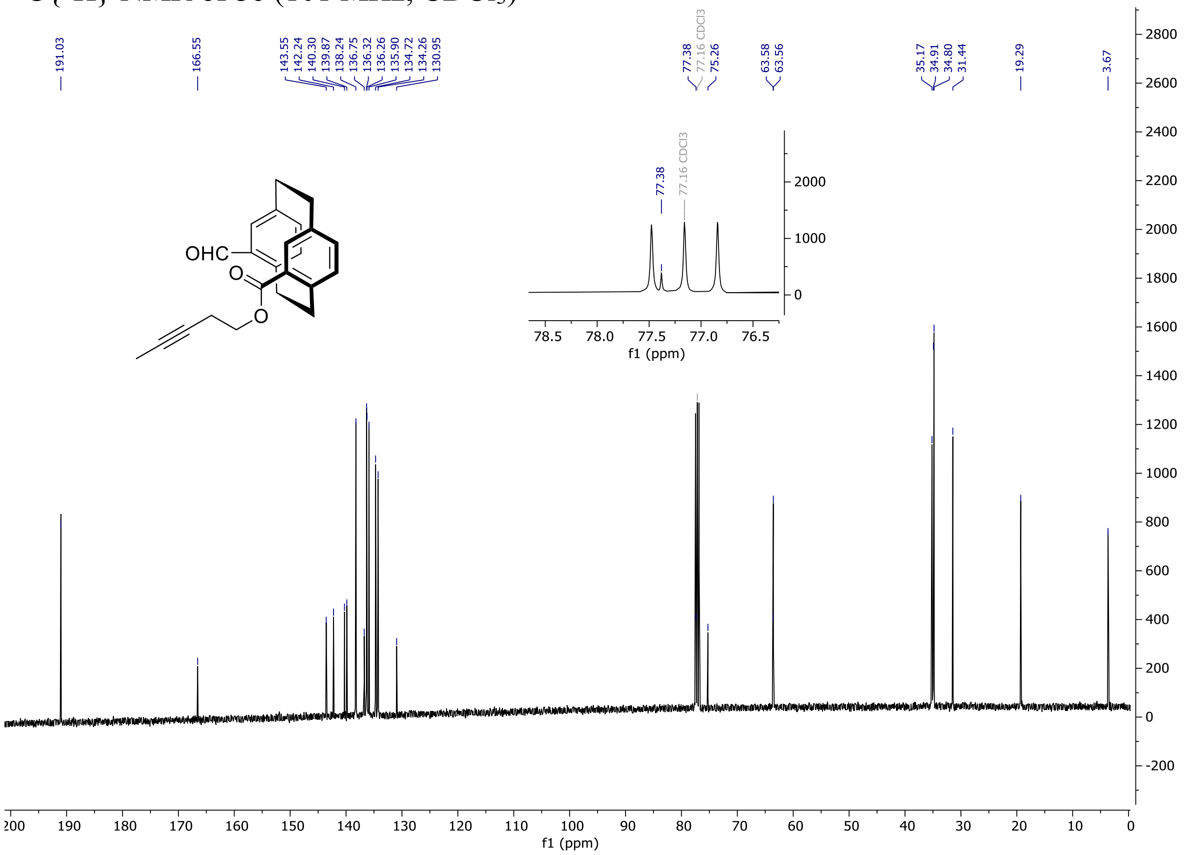

(R_p)-Phenethyl 4²-formyl-1,4(1,4)-dibenzenacyclohexaphane-1²-carboxylate (5f)

^{1}H NMR of **5f** (400 MHz, $CDCl_3$)

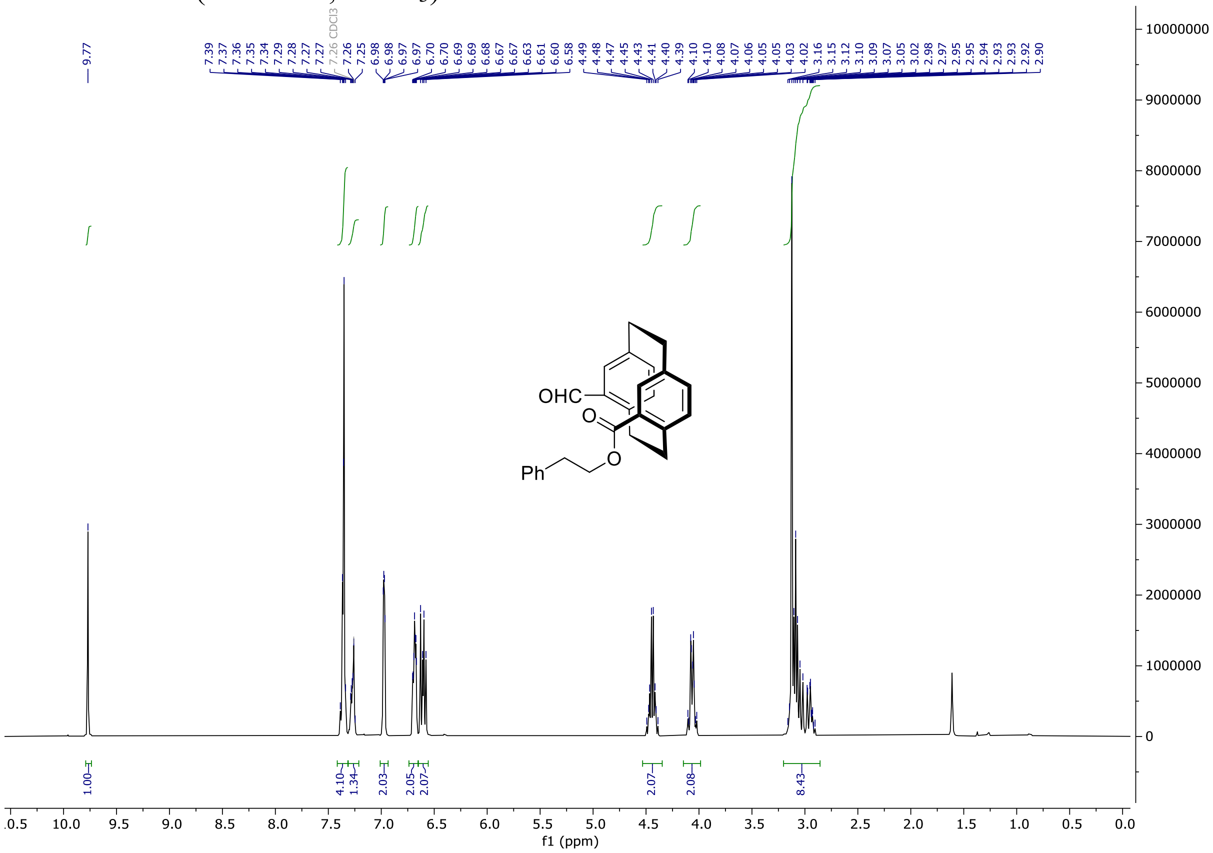

^{13}C{^{1}H} NMR of **5f** (101 MHz, $CDCl_3$)

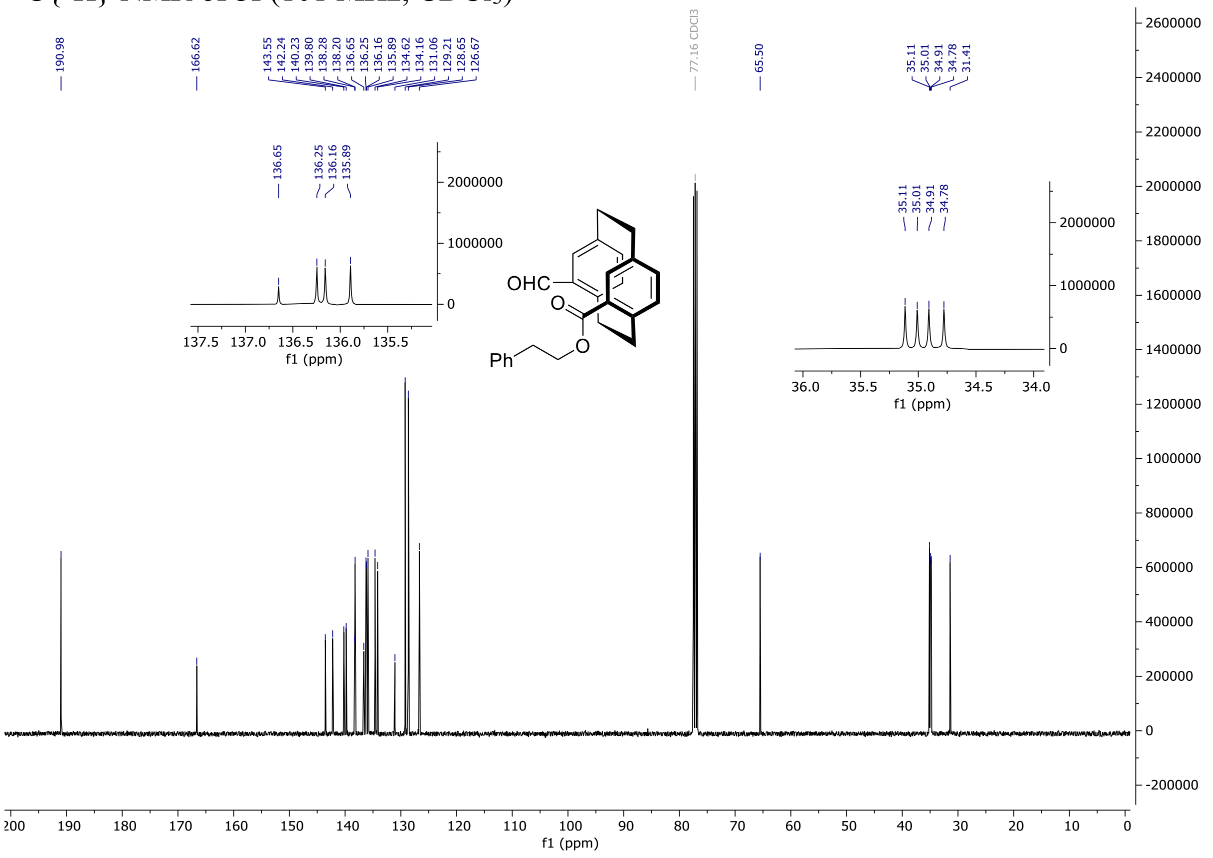

(R_p)-2-(4-Methylthiazol-5-yl)ethyl 4²-formyl-1,4(1,4)-dibenzenacyclohexaphane-1²-carboxylate (5g)

^{1}H NMR of **5g** (400 MHz, CDCl$_3$)

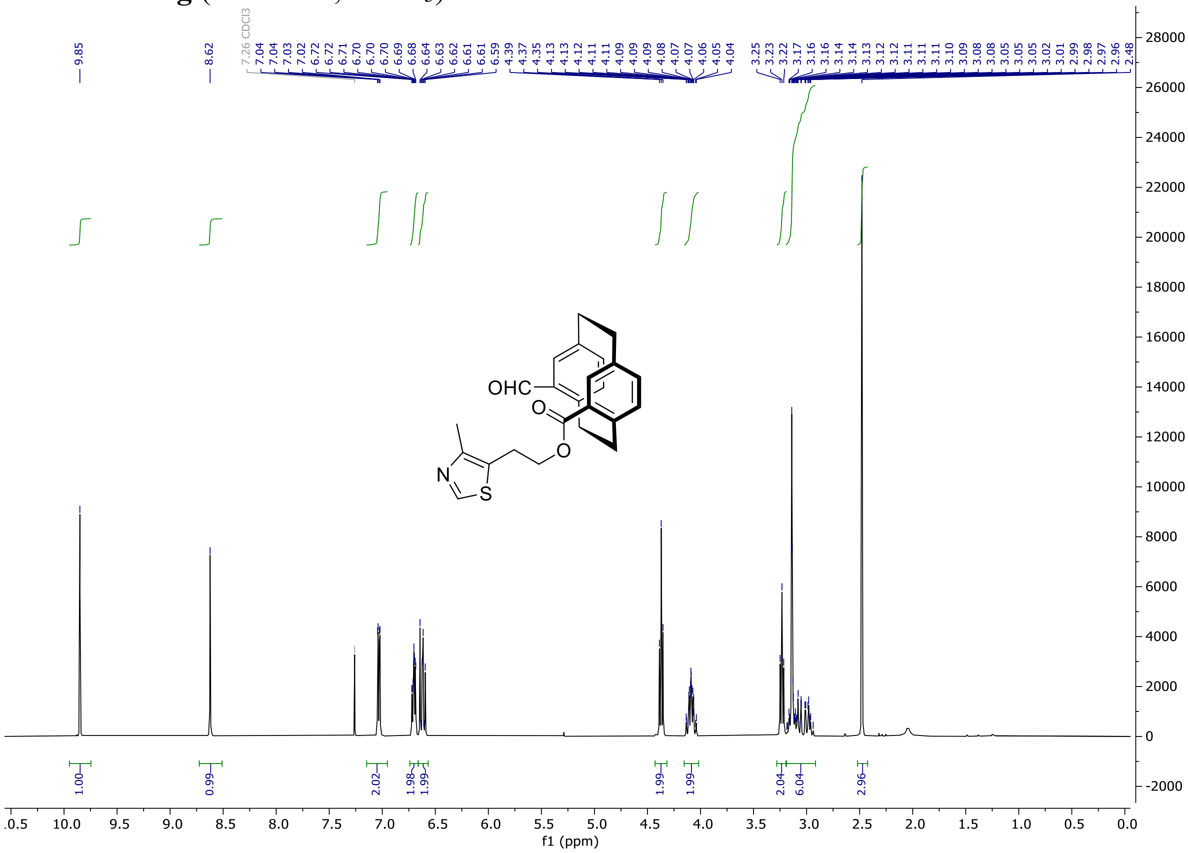

^{13}C{^{1}H} NMR of **5g** (101 MHz, CDCl$_3$)

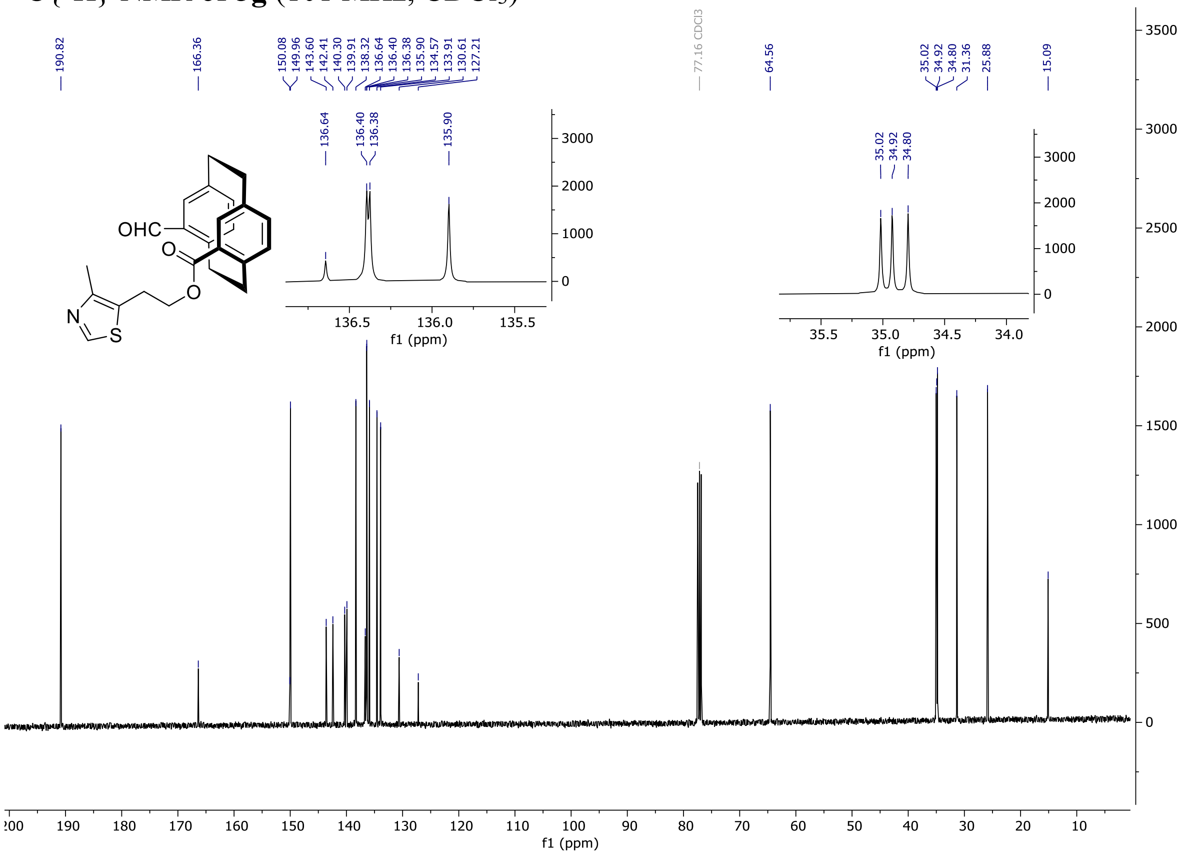

(*R*$_p$)-*S*-Ethyl 4^2-formyl-1,4(1,4)-dibenzenacyclohexaphane-1^2-carbothioate (5h)

^{1}H NMR of **5h** (400 MHz, $CDCl_3$)

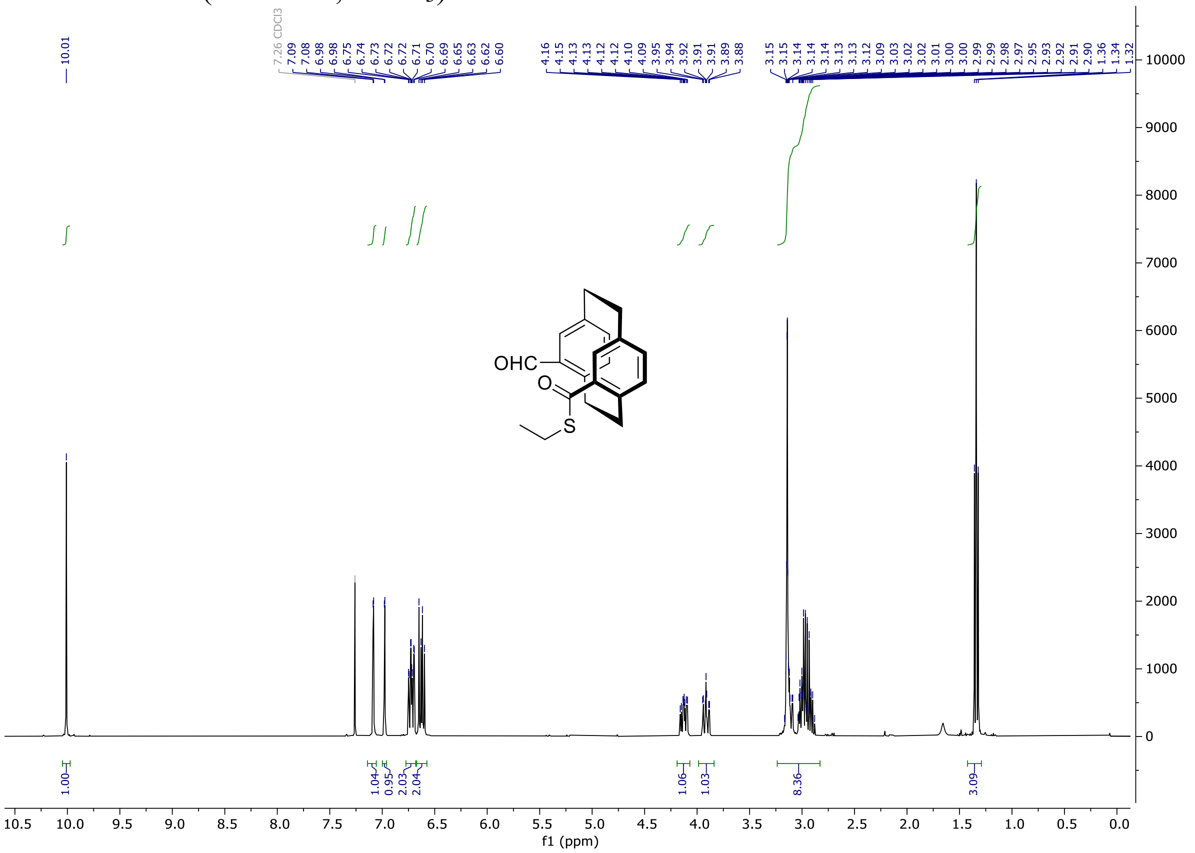

$^{13}C\{^1H\}$ NMR of **5h** (101 MHz, $CDCl_3$)

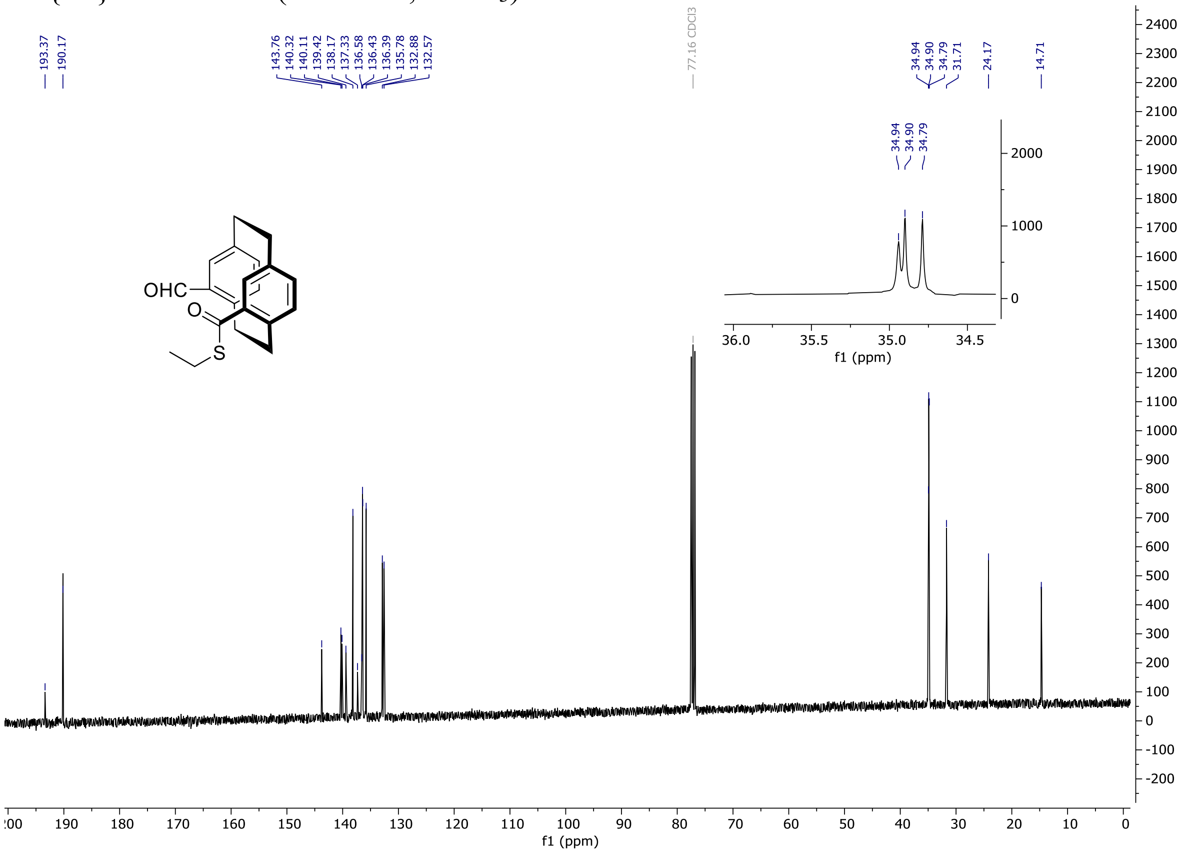

(S_p)-Methyl 4²-((ethylthio)carbonyl)-1,4(1,4)-dibenzenacyclohexaphane-1²-carboxylate (7)

^{1}H NMR of **7** (400 MHz, CDCl$_3$)

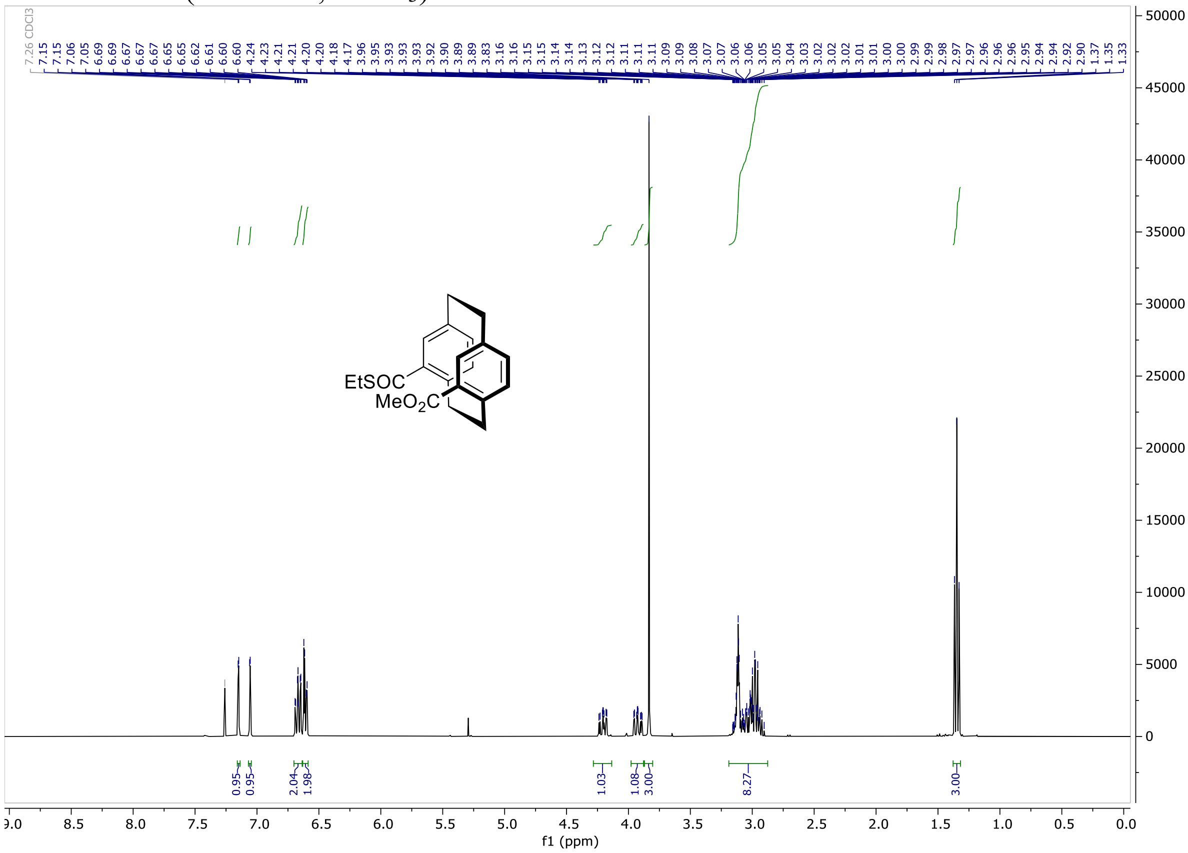

^{13}C{^{1}H} NMR of **7** (101 MHz, CDCl$_3$)

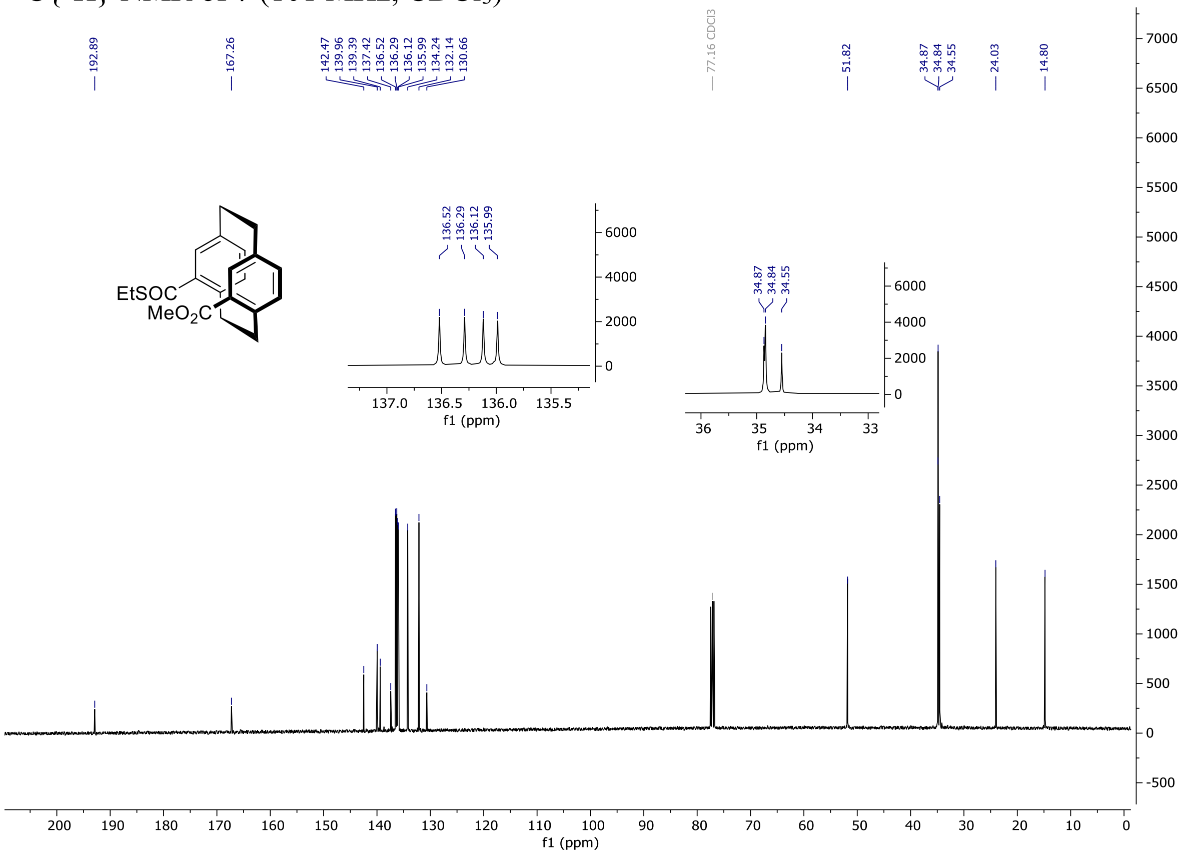

(R_p)-Methyl (E)-4²-(3-ethoxy-3-oxoprop-1-en-1-yl)-1,4(1,4)-dibenzenacyclohexaphane-1²-carboxylate (8)

^{1}H NMR of **8** (400 MHz, $CDCl_3$)

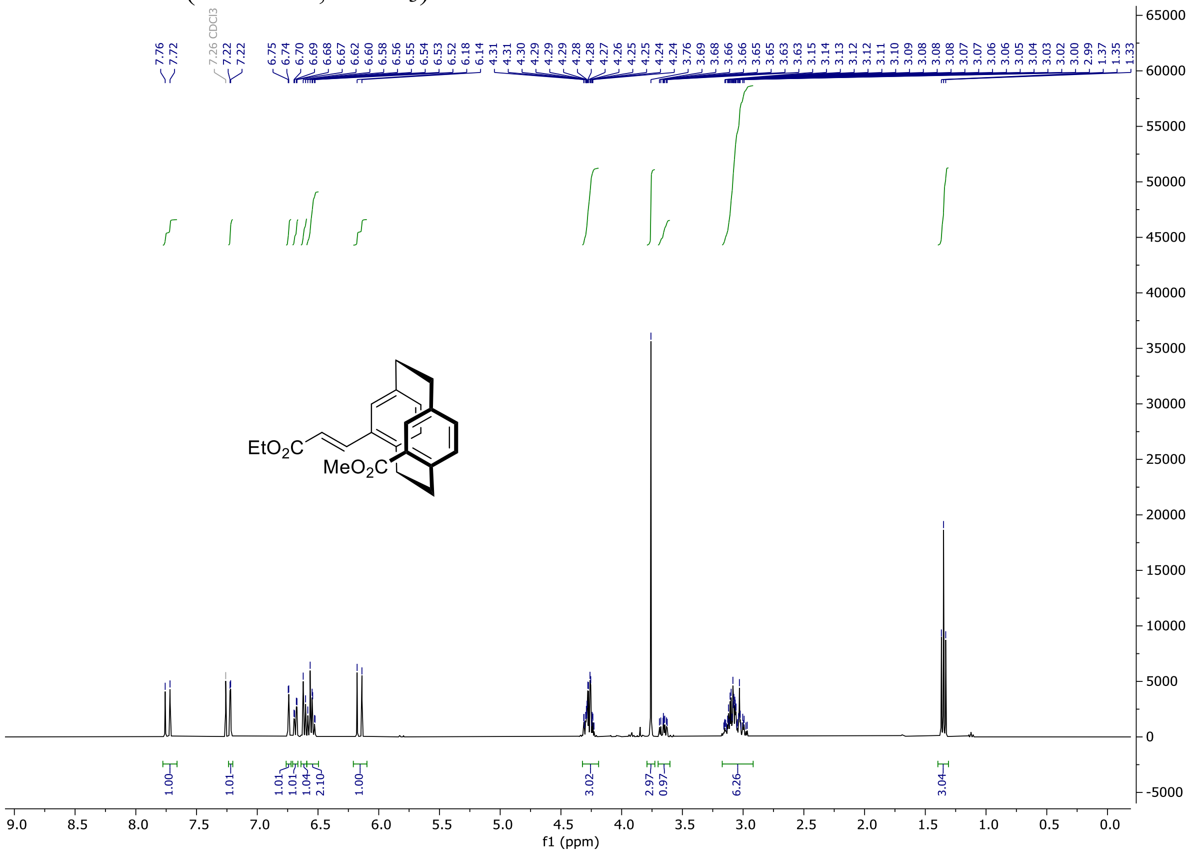

$^{13}C\{^1H\}$ NMR of **8** (101 MHz, $CDCl_3$)

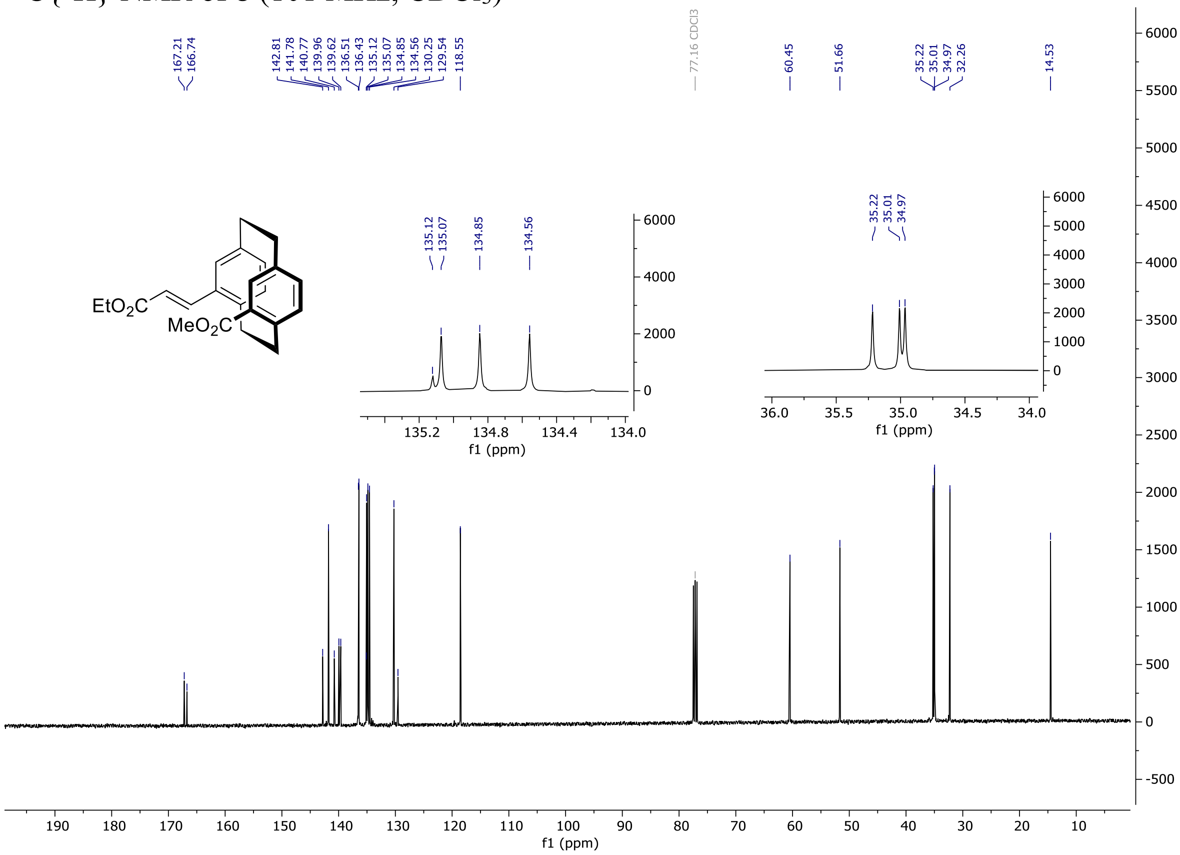

(R_p)-Methyl 4²-((benzylamino)methyl)-1,4(1,4)-dibenzenacyclohexaphane-1²-carboxylate (9)

^{1}H NMR of **9** (400 MHz, $CDCl_3$)

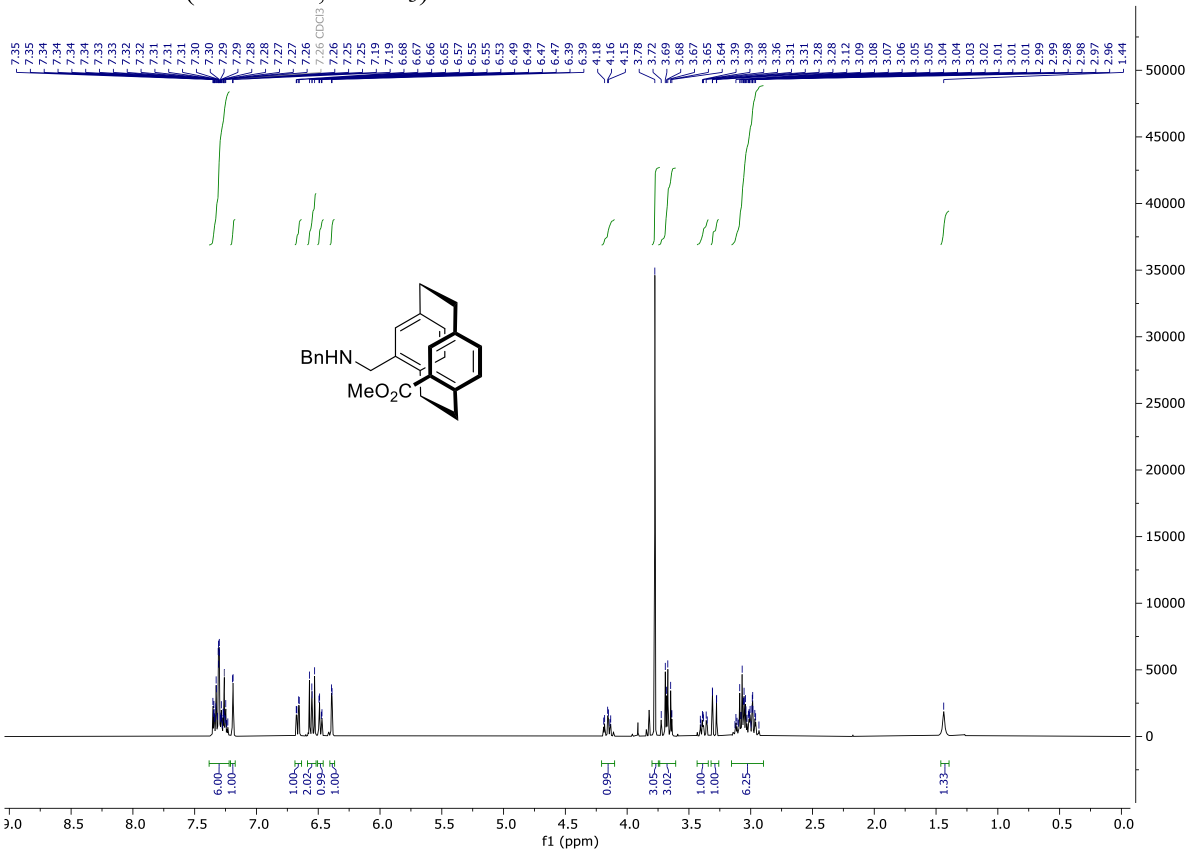

^{13}C{^{1}H} NMR of **9** (101 MHz, $CDCl_3$)

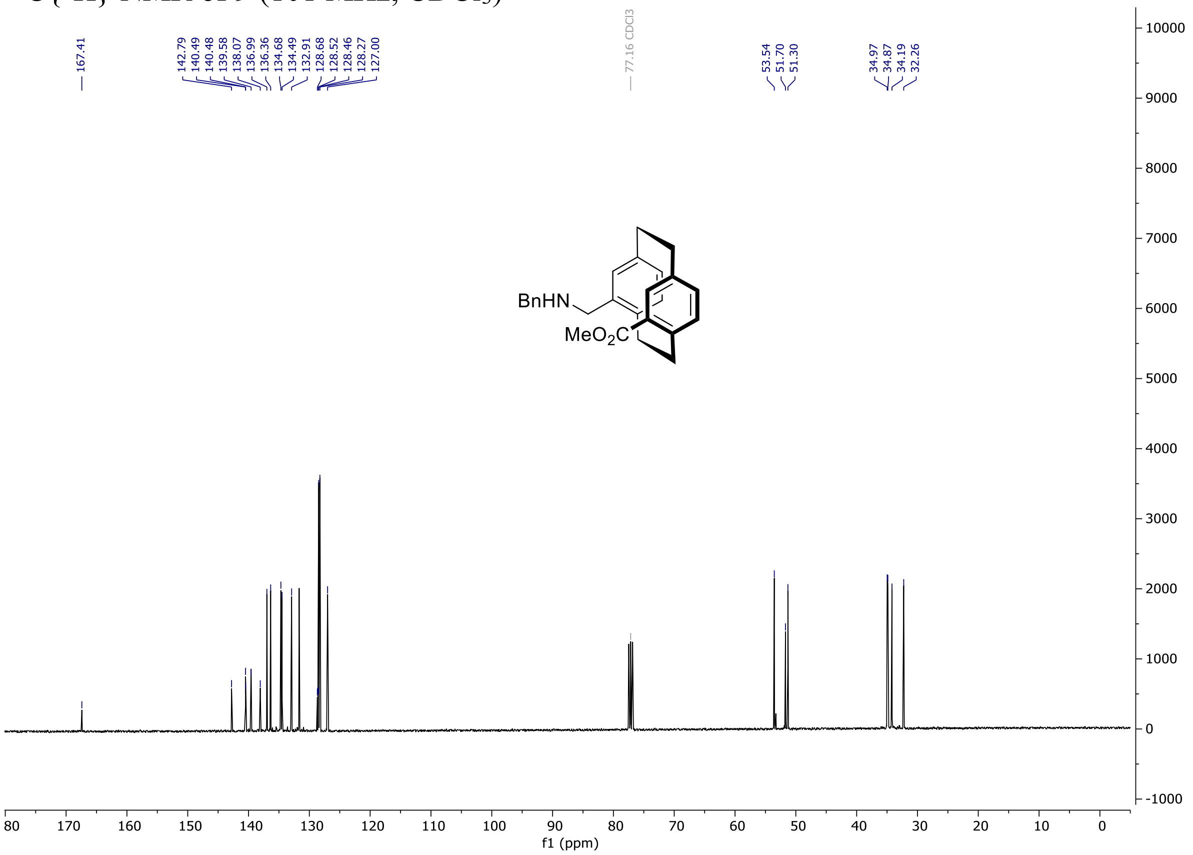

(R_p)-Methyl 4²-(hydroxymethyl)-1,4(1,4)-dibenzenacyclohexaphane-1²-carboxylate (10)

^{1}H NMR of **10** (400 MHz, $CDCl_3$)

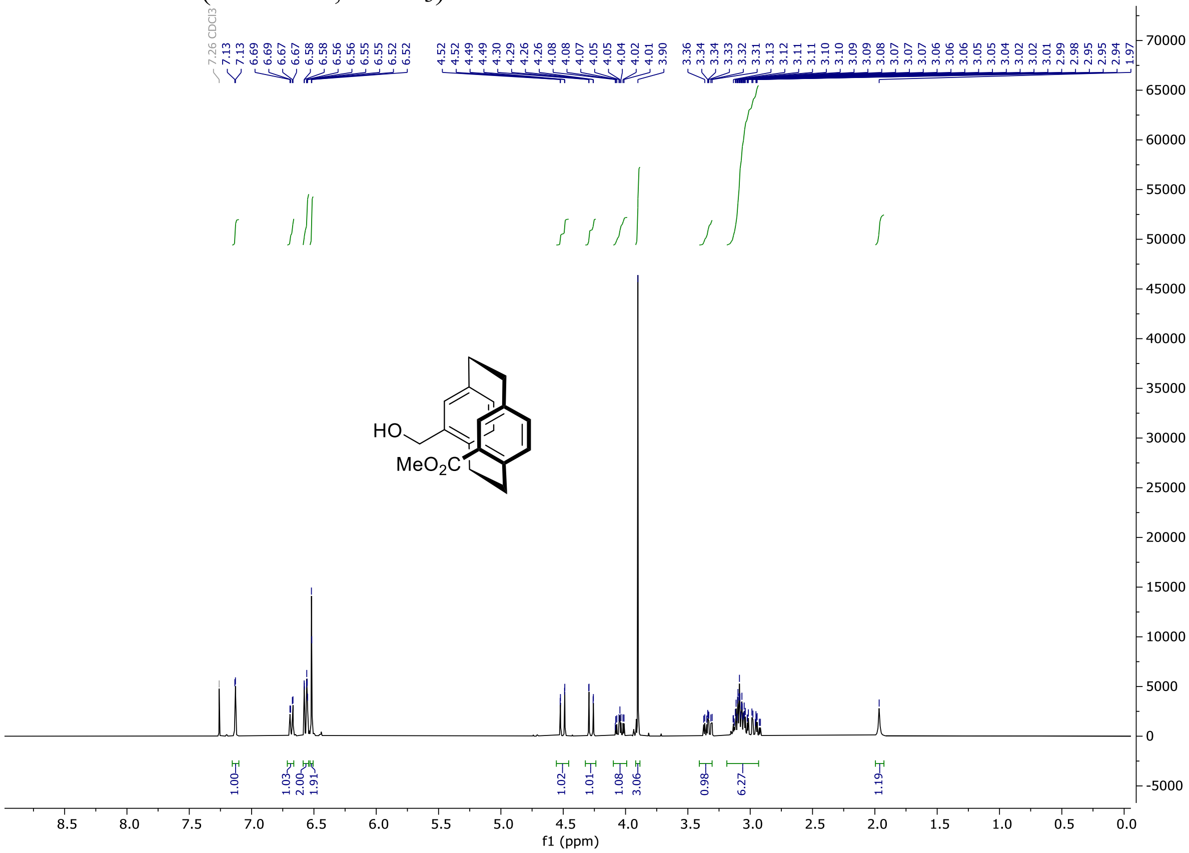

$^{13}C\{^{1}H\}$ NMR of **10** (101 MHz, $CDCl_3$)

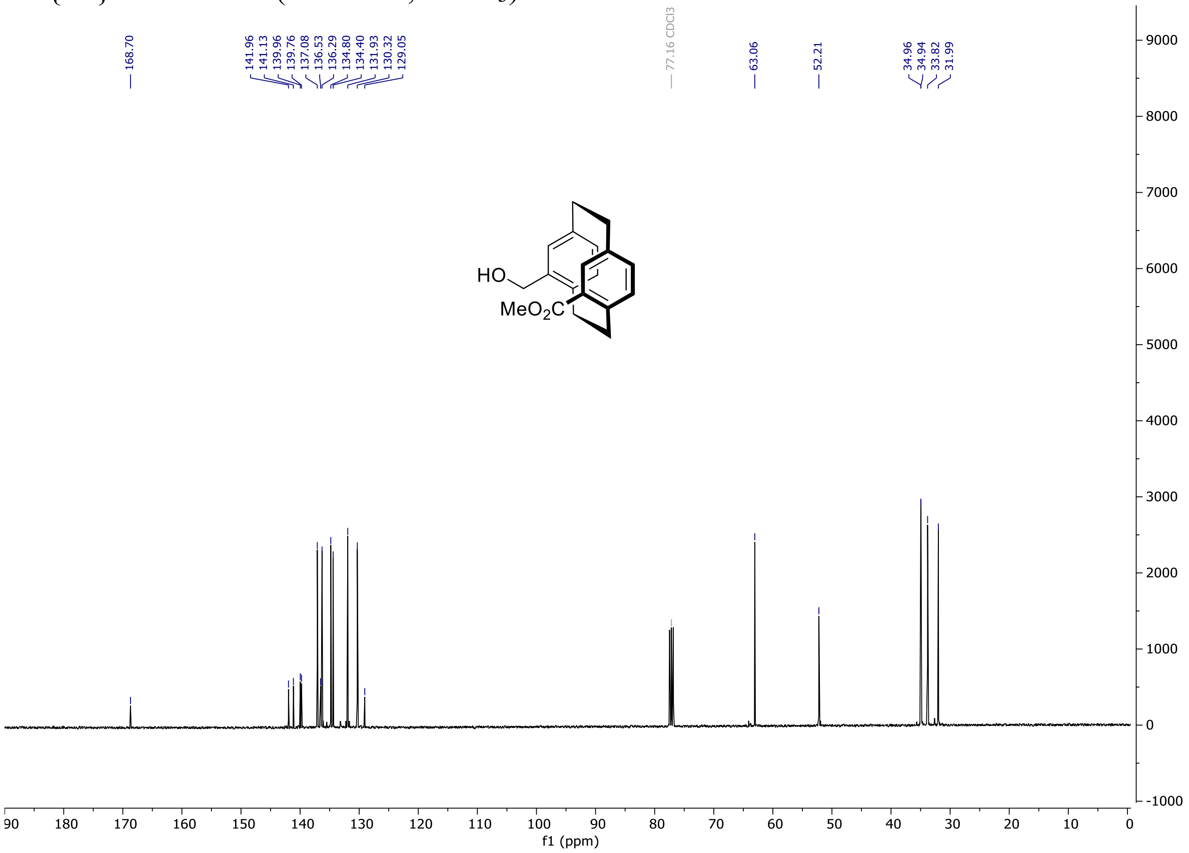

(S_p)-4²-(Methoxycarbonyl)-1,4(1,4)-dibenzenacyclohexaphane-1²-carboxylic acid (11)

^{1}H NMR of **11** (400 MHz, MeOD)

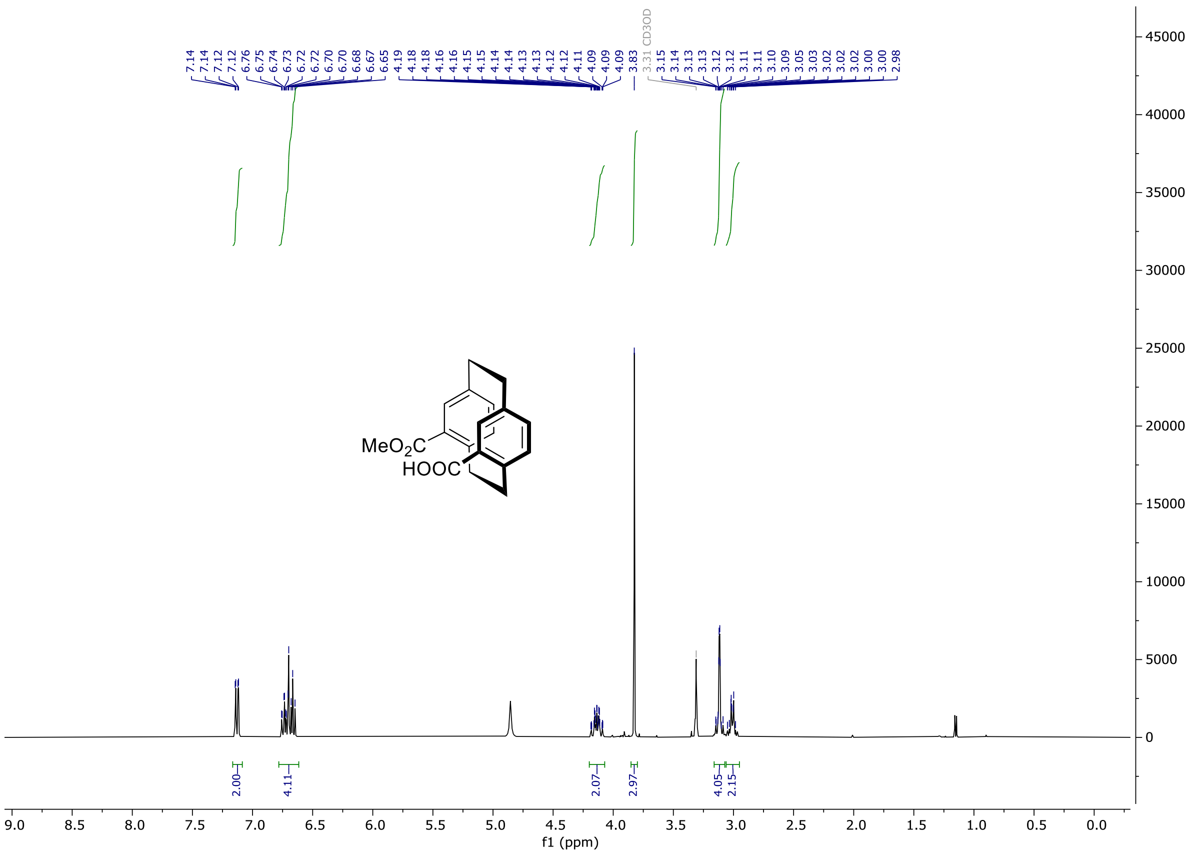

^{13}C{^{1}H} NMR of **11** (101 MHz, MeOD)

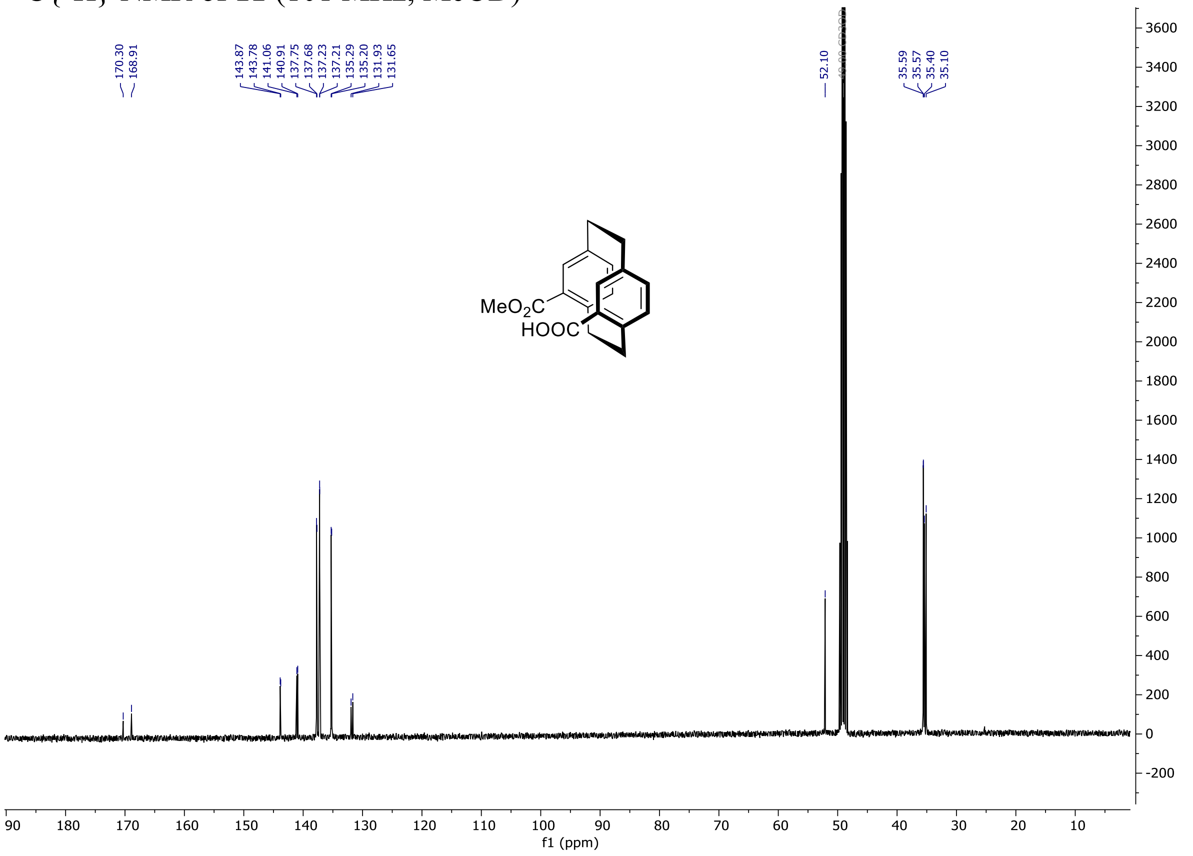

(S_p)-4²-Hydroxy-1,4(1,4)-dibenzenacyclohexaphane-1²-carbaldehyde (12)

^{1}H NMR of **12** (400 MHz, CDCl$_3$)

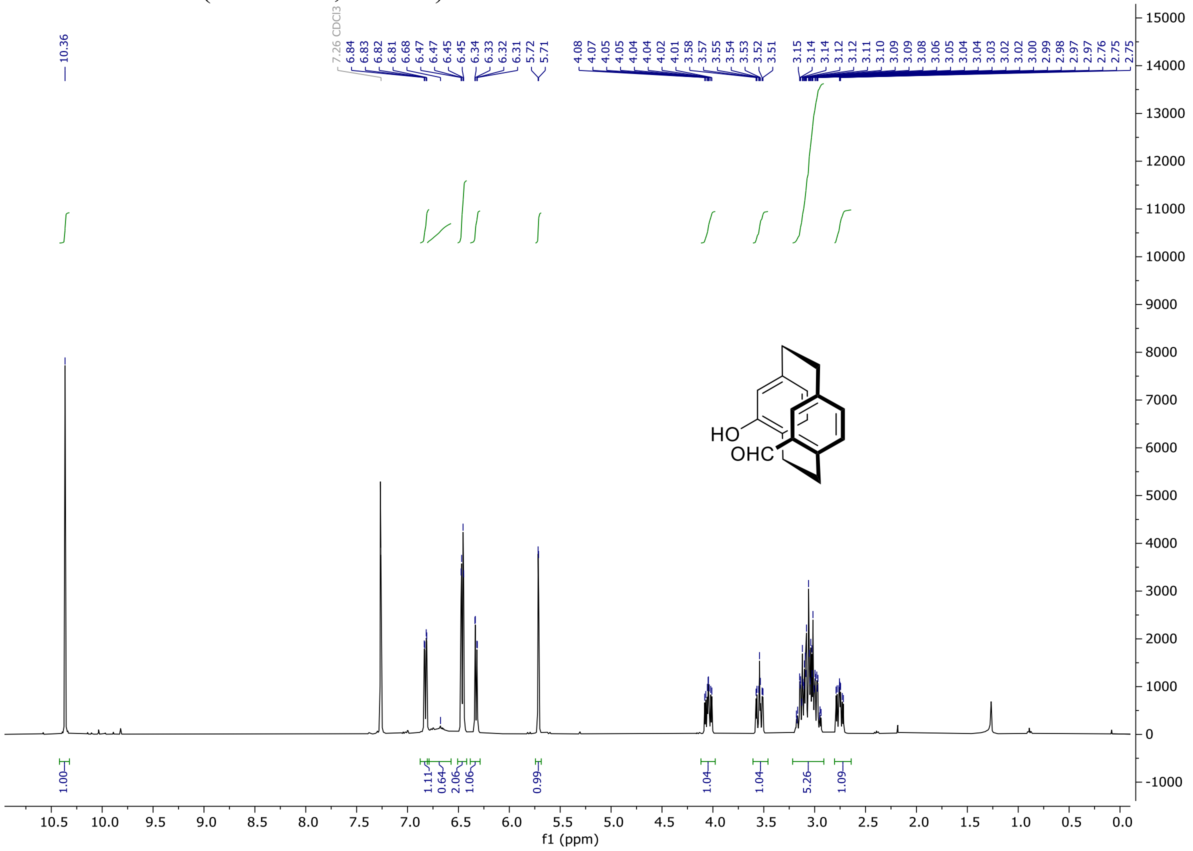

^{13}C{^{1}H} NMR of **12** (101 MHz, CDCl$_3$)

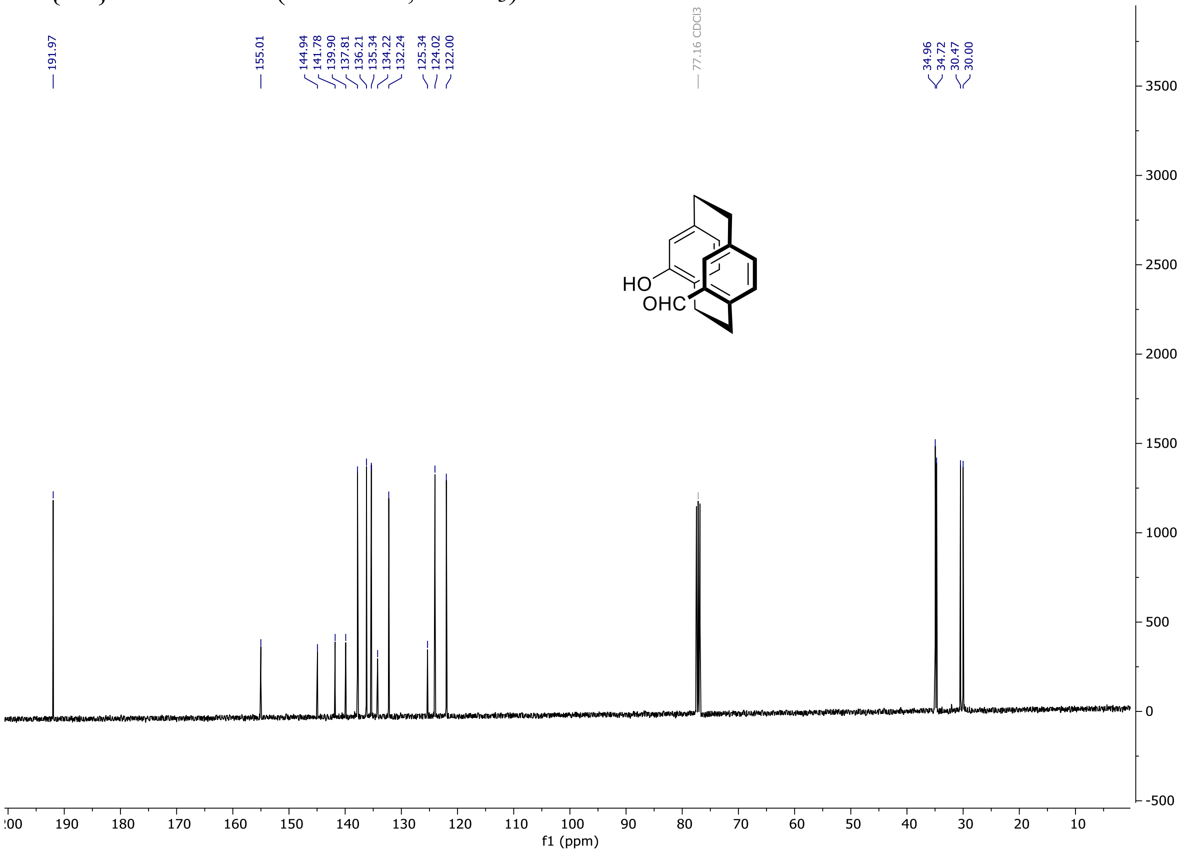

(S_p)-4²-Hydroxy-1,4(1,4)-dibenzenacyclohexaphane-1²-carboxylic acid (13)

^{1}H NMR of **13** (400 MHz, MeOD)

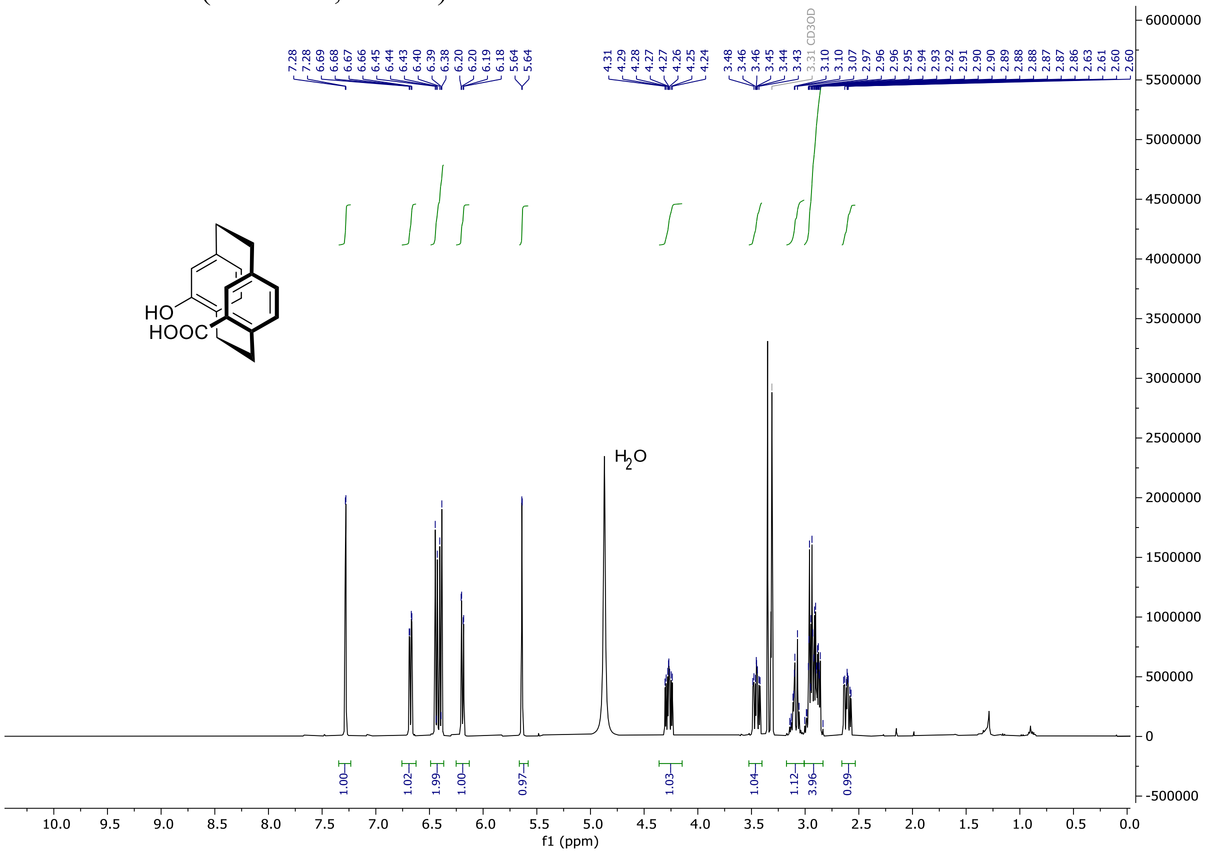

^{13}C{^{1}H} NMR of **13** (101 MHz, MeOD)

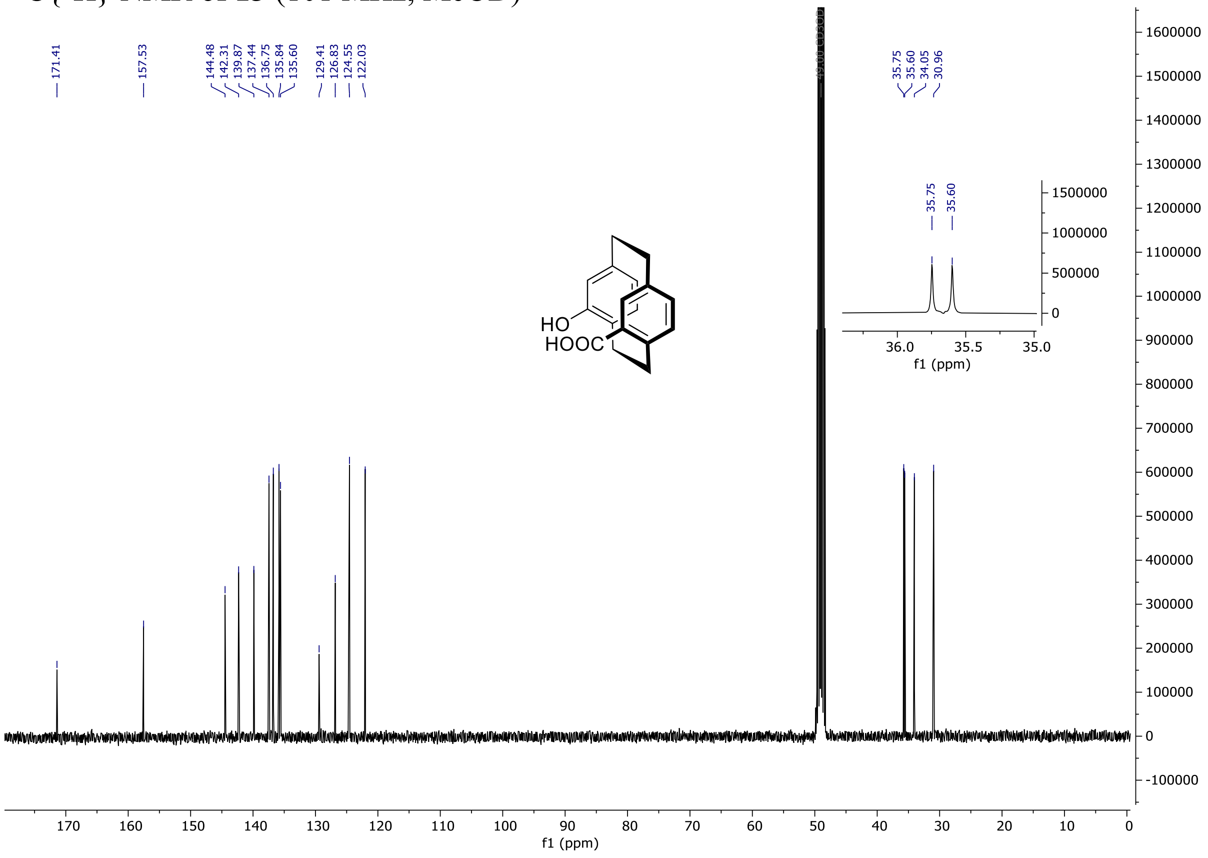

3-Isobutyl-3,4-dihydro-2*H*-benzo[*e*][1,2,4]thiadiazine 1,1-dioxide (16)

^{1}H NMR of **16** (400 MHz, $CDCl_3$)

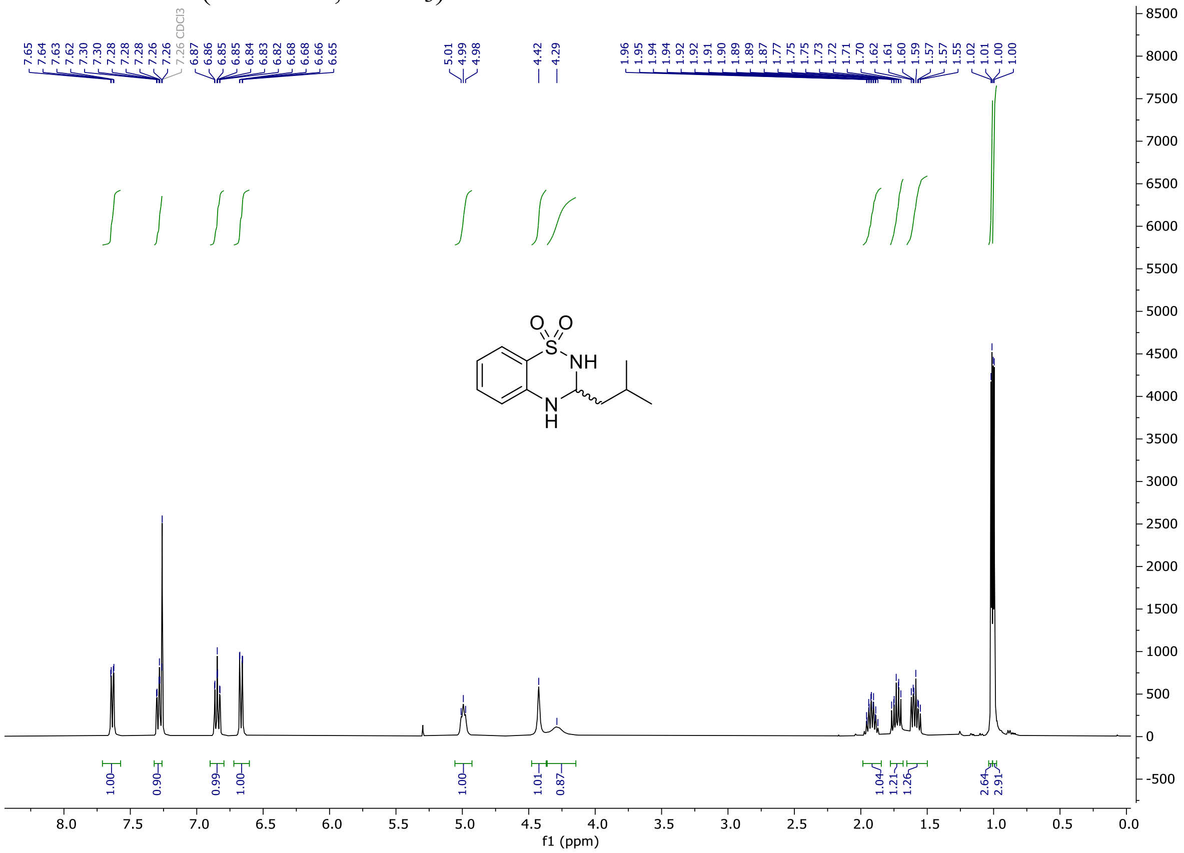

$^{13}C\{^{1}H\}$ NMR of **16** (101 MHz, $CDCl_3$)

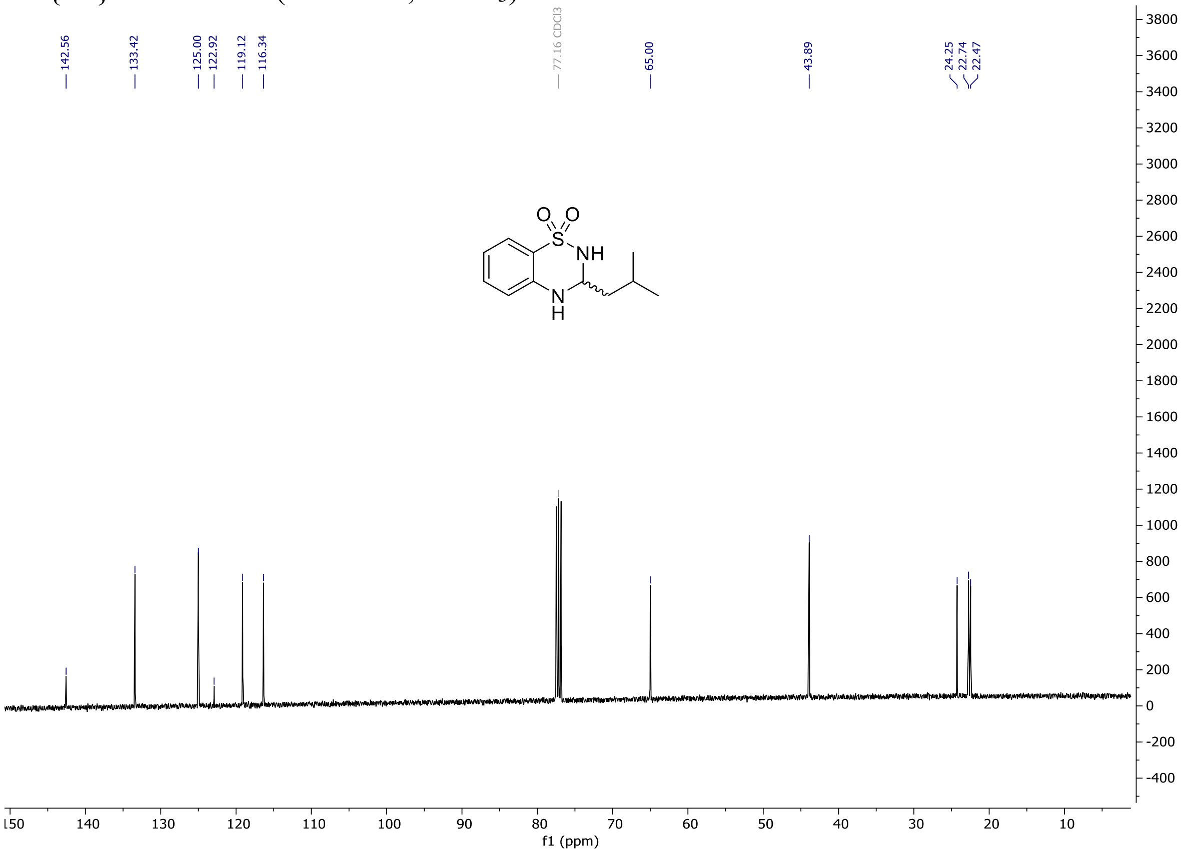

2-Nitro-1-phenylethan-1-ol (19)

^{1}H NMR of **14** (400 MHz, $CDCl_3$)

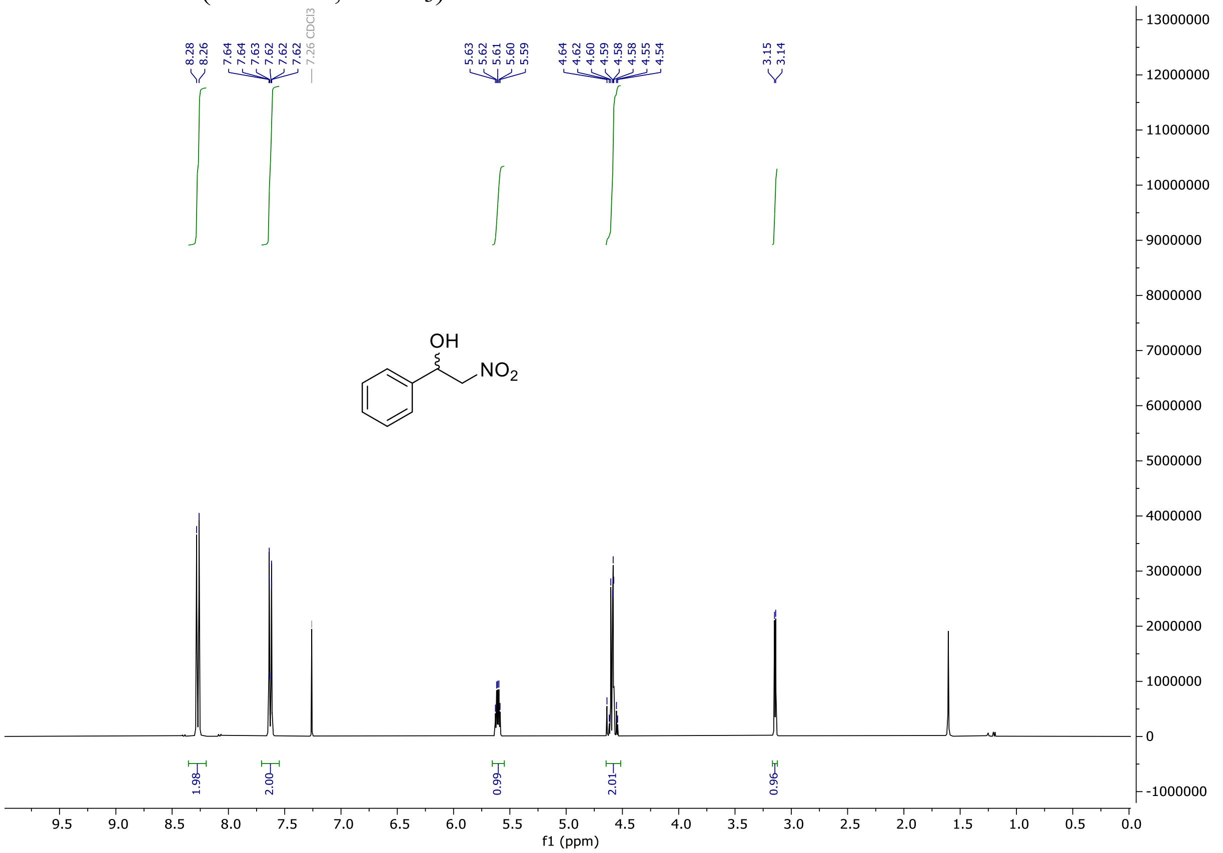

^{13}C{^{1}H} NMR of **19** (101 MHz, $CDCl_3$)

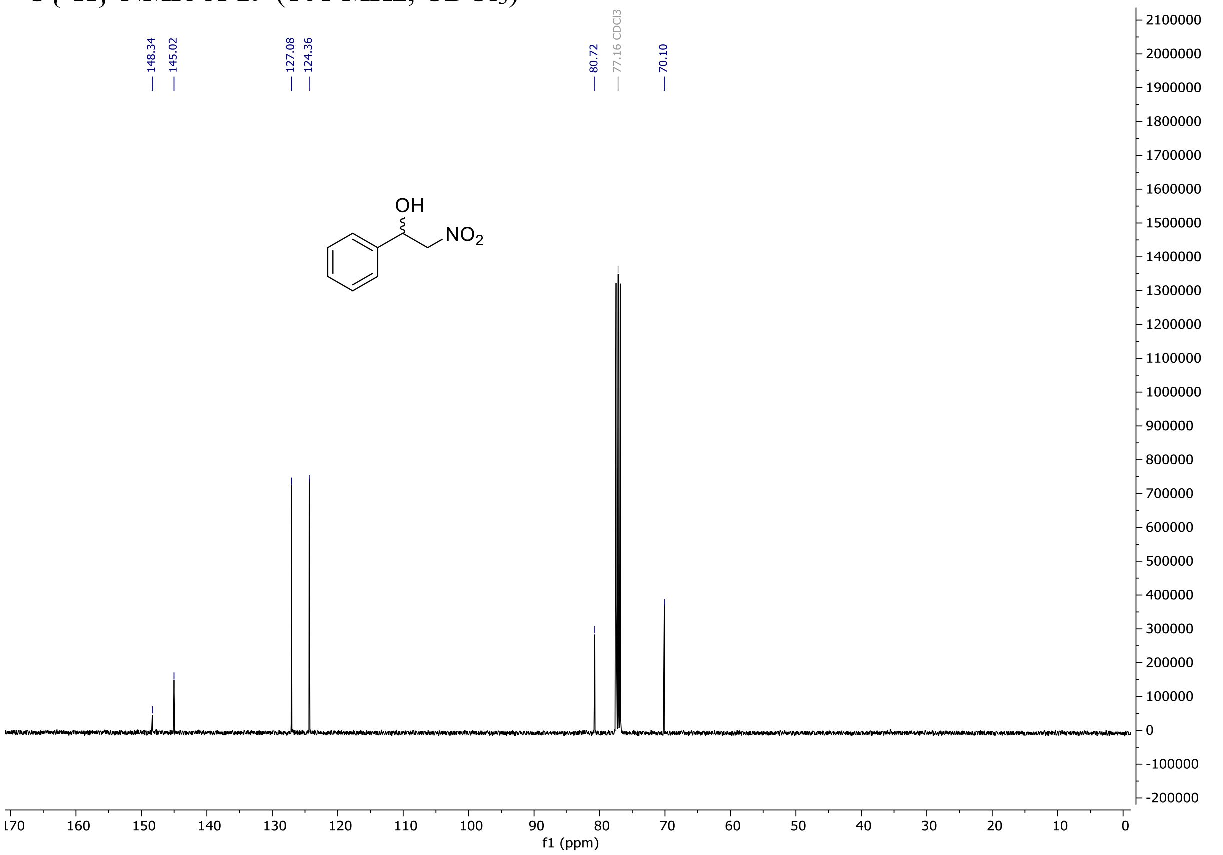

1,4(1,4)-Dibenzenacyclohexaphane-1²,4³-dicarbaldehyde-*d₂* (1a-*d₂*)

^{1}H NMR of **1a**-d_2 (400 MHz, $CDCl_3$)

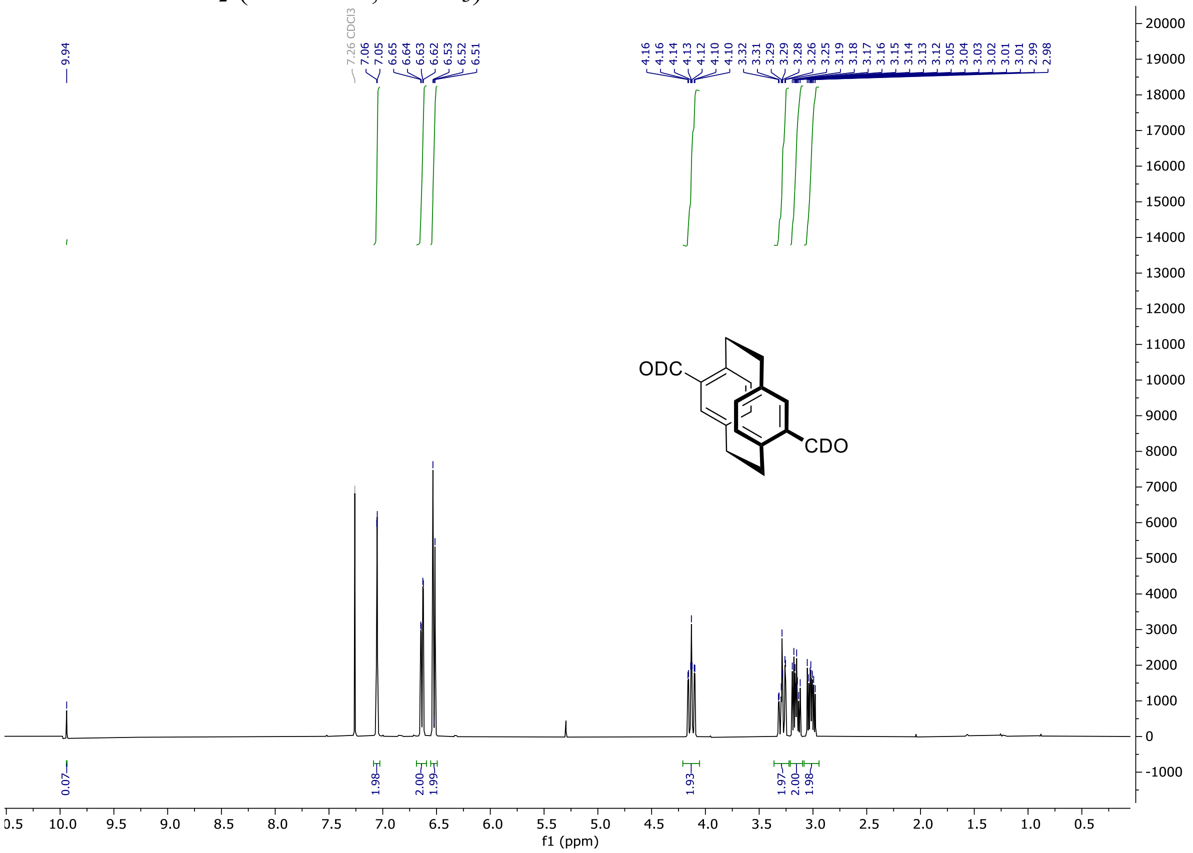

^{2}H NMR of **1a**-d_2 (92 MHz, $CDCl_3$)

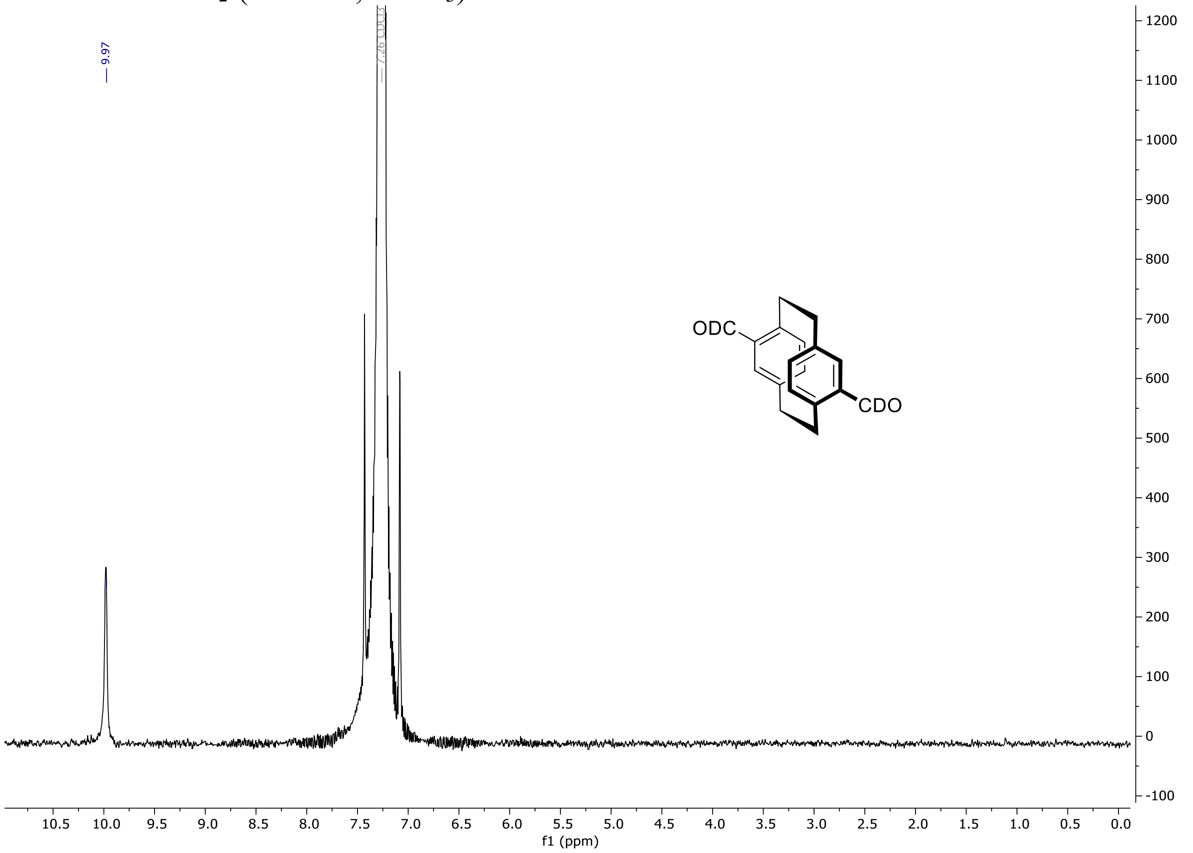

$^{13}C\{^1H\}$ NMR of **1a**-d_2 (101 MHz, $CDCl_3$)

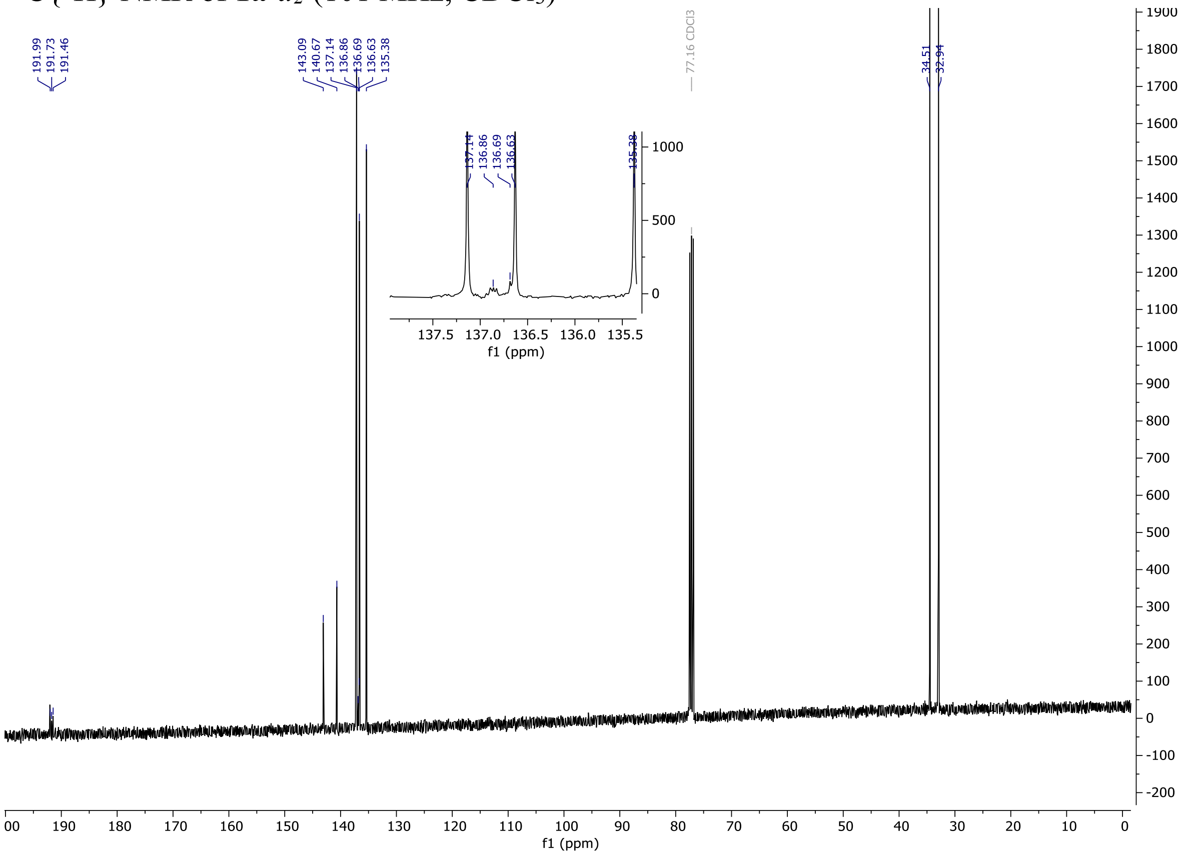

1,4(1,4)-Dibenzenacyclohexaphane-1^2,4^2-dicarbaldehyde-d_2 (1b-d_2)

^{1}H NMR of **1b**-d_2 (400 MHz, $CDCl_3$)

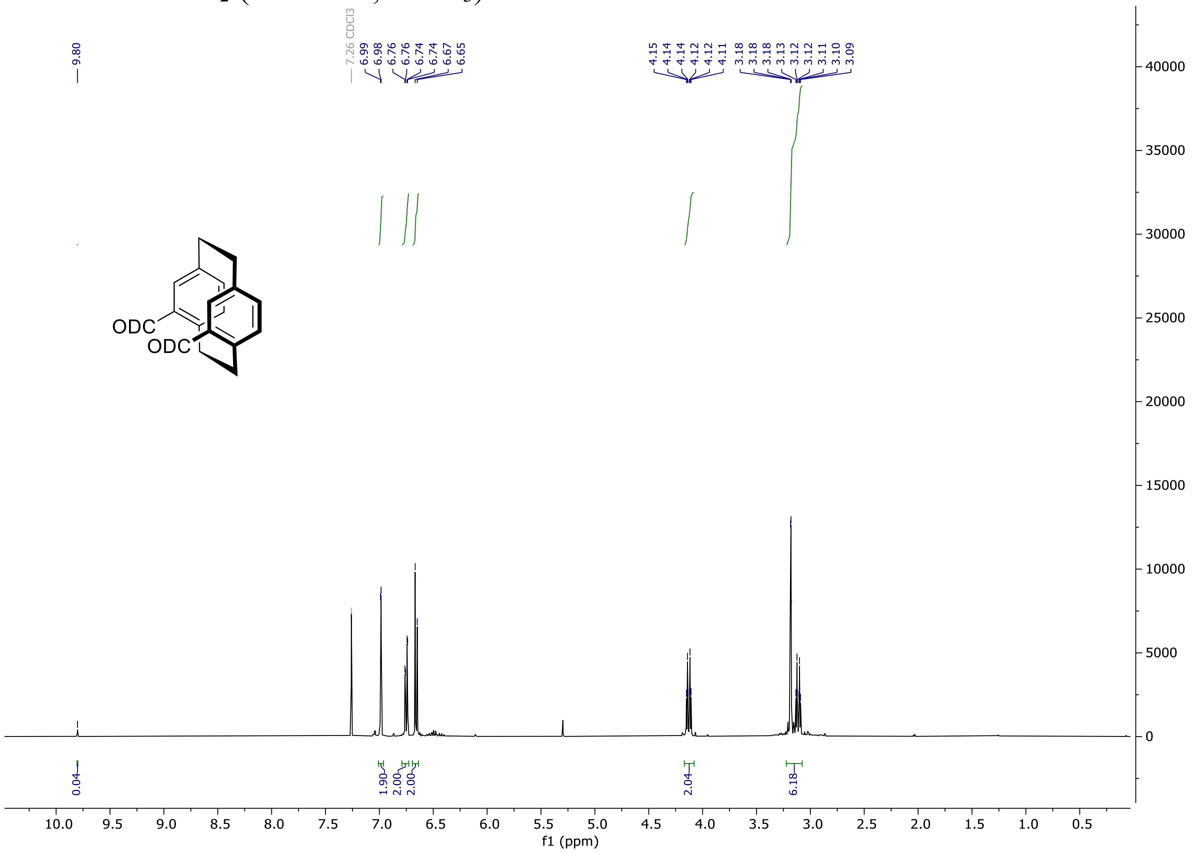

^{2}H NMR of **1b**-d_2 (92 MHz, $CDCl_3$)

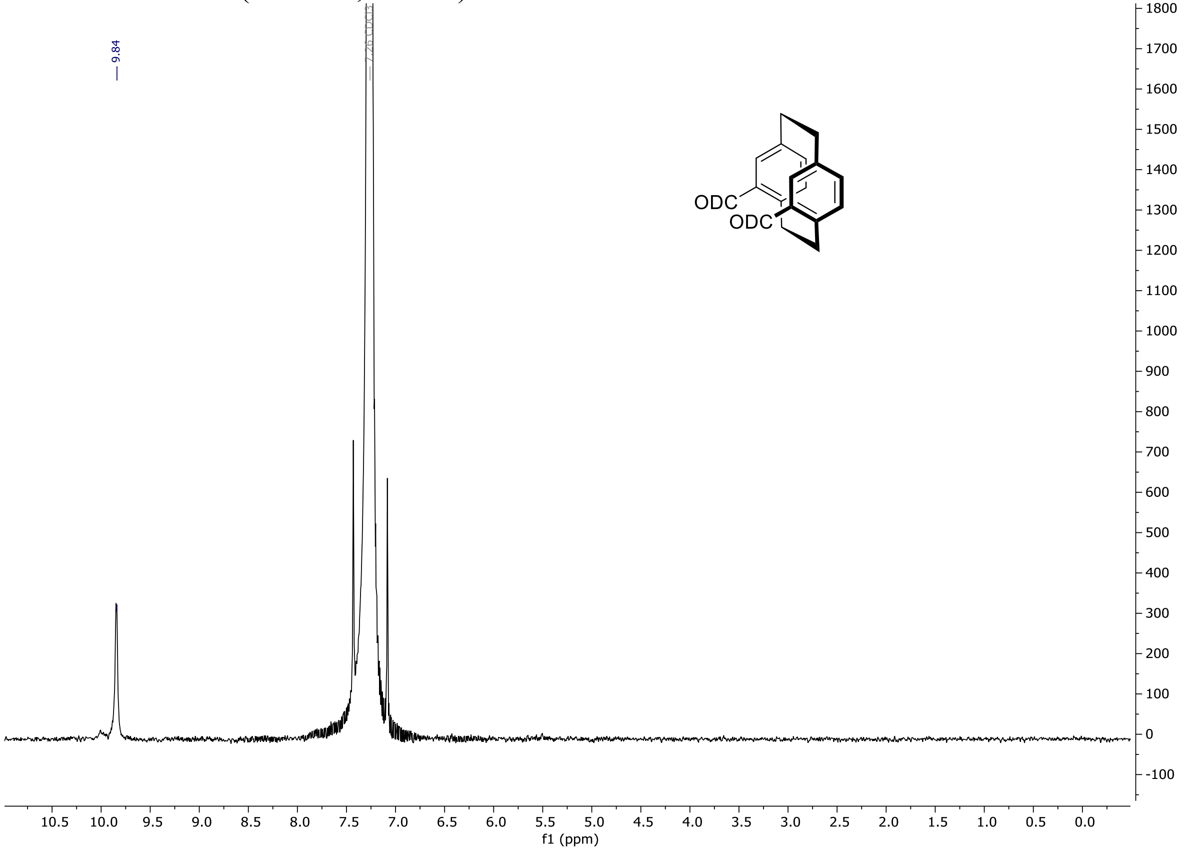

$^{13}C\{^{1}H\}$ NMR of **1b**-d_2 (101 MHz, $CDCl_3$)

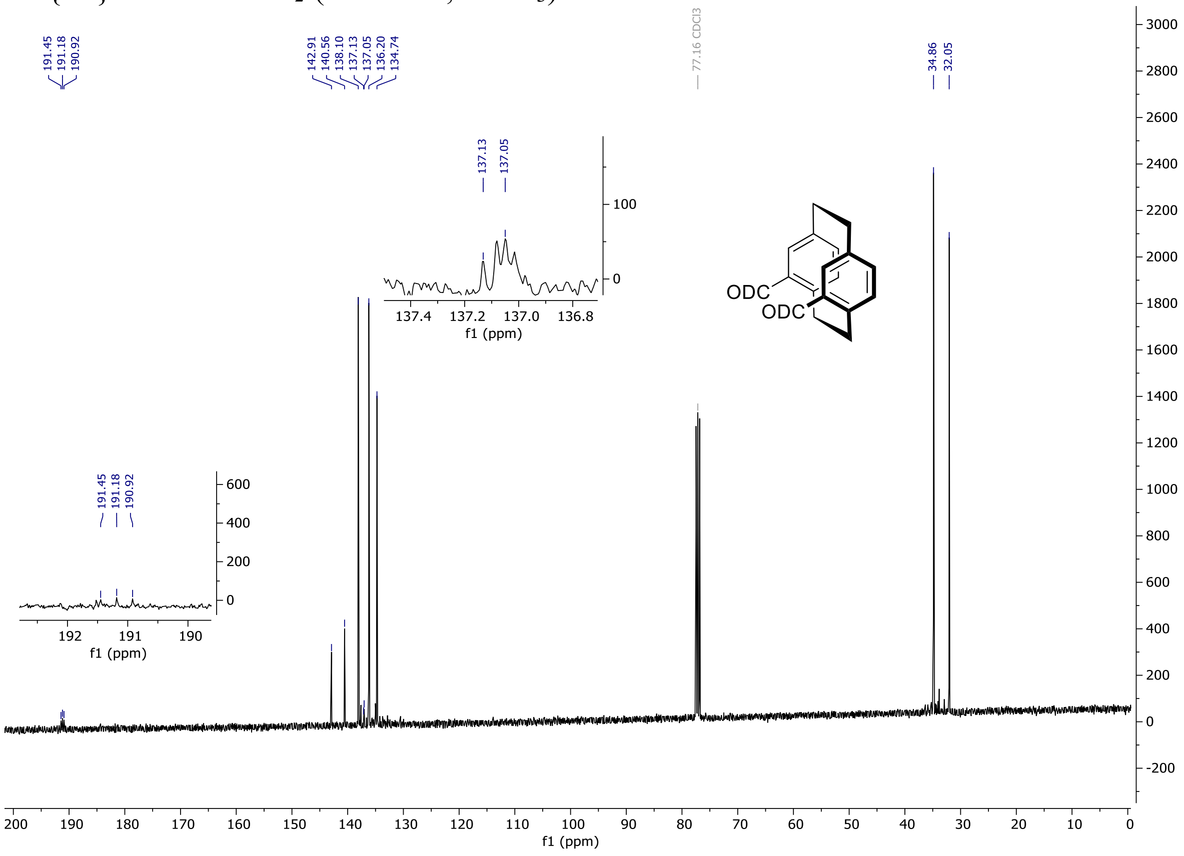

Chiral HPLC

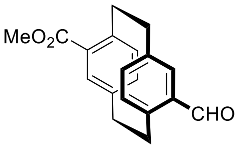

Conditions: IB column
mobile phase: *n*-heptane/*i*-PrOH – 80:20
λ = 190 nm, V = 1.0 ml/min, t = 25 °C
for **3a**: t_R = 7.8 min (major), t_R = 10.1 min (minor)

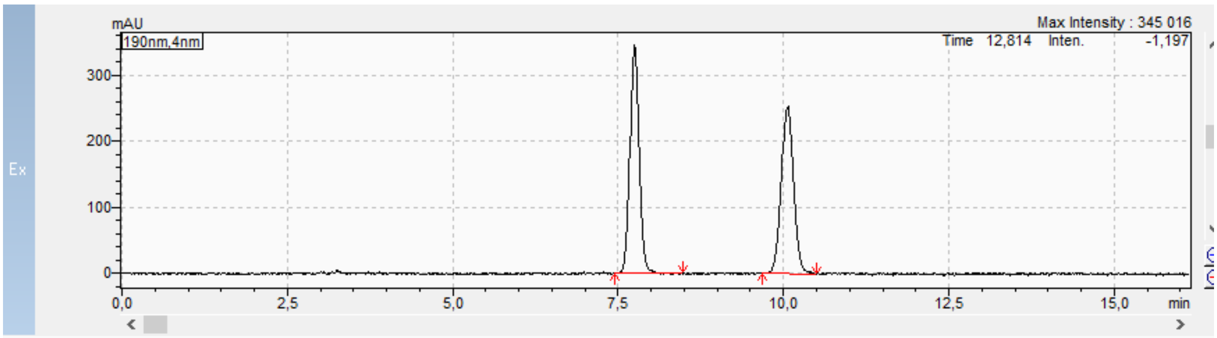

Results View - Peak Table

Peak Table | Compound | Group | Calibration Curve

Peak#	Ret. Time	Area	Height	Mark	Conc.	Unit	ID#	Name	Area%
1	7,746	3286877	344528	M	50,417				50,417
2	10,065	3232535	252455	M	49,583				49,583
Total		6519413	596983		100,000				100,000

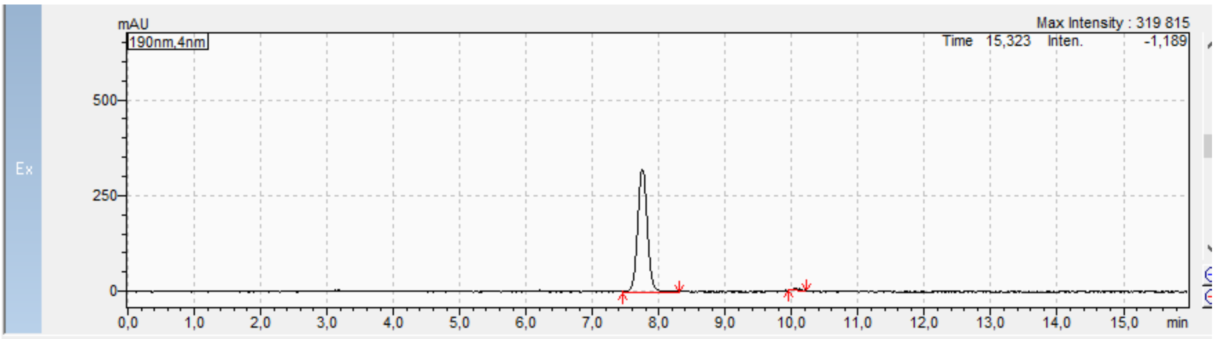

Results View - Peak Table

Peak Table | Compound | Group | Calibration Curve

Peak#	Ret. Time	Area	Height	Mark	Conc.	Unit	ID#	Name	Area%
1	7,751	3151167	321340	M	98,836				98,836
2	10,068	37122	5011	M	1,164				1,164
Total		3188290	326351		100,000				100,000

for **3a**: *er* = 99:1 (*ee* = 98%)

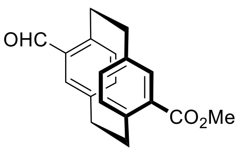

Conditions: IB column
mobile phase: *n*-heptane/*i*-PrOH – 80:20
λ = 190 nm, V = 1.0 ml/min, t = 25 °C
for *ent*-**3a**: t_R = 7.8 min (minor), t_R = 10.1 min (major)

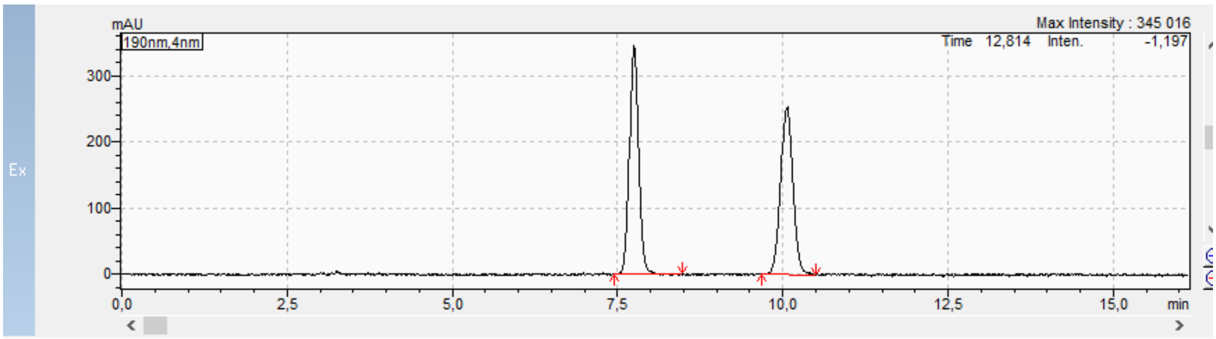

Results View - Peak Table

Peak Table | Compound | Group | Calibration Curve

Peak#	Ret. Time	Area	Height	Mark	Conc.	Unit	ID#	Name	Area%
1	7,746	3286877	344528	M	50,417				50,417
2	10,065	3232535	252455	M	49,583				49,583
Total		6519413	596983		100,000				100,000

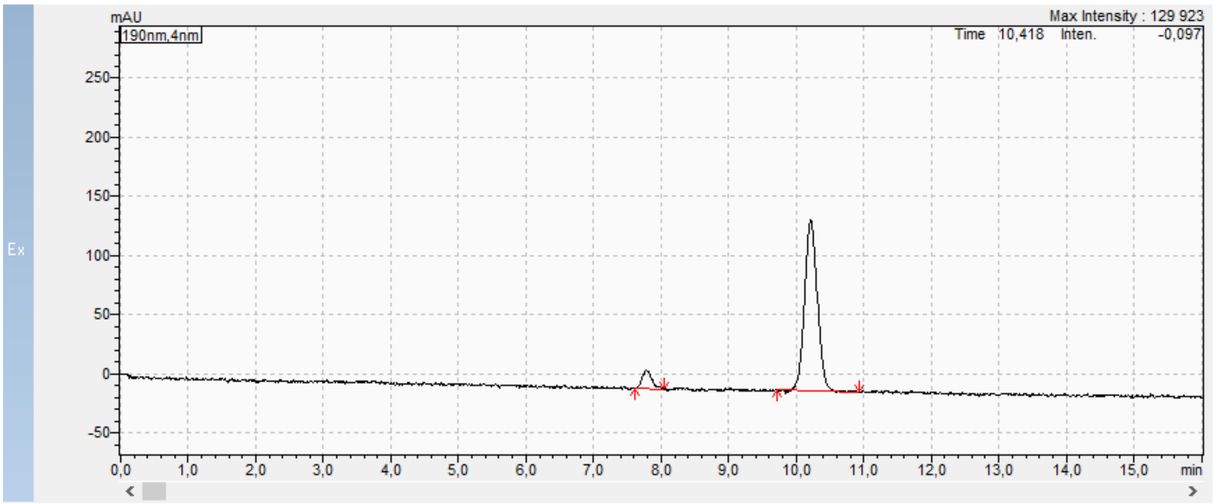

Results View - Peak Table

Peak Table | Compound | Group | Calibration Curve

Peak#	Ret. Time	Area	Height	Peak Start	Peak End	Mark	Conc.	Unit	Area%
1	7,782	153373	15433	7,616	8,043	M	7,468		7,468
2	10,216	1900312	144228	9,717	10,933	M	92,532		92,532
Total		2053686	159661				100,000		100,000

for *ent*-**3a**: *er* = 93:7 (*ee* = 85%)

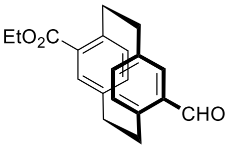

Conditions: IB column
mobile phase: *n*-heptane/*i*-PrOH – 80:20
λ = 190 nm, V = 1.0 ml/min, t = 25 °C
for **3b**: t_R = 6.8 min (major), t_R = 8.8 min (minor)

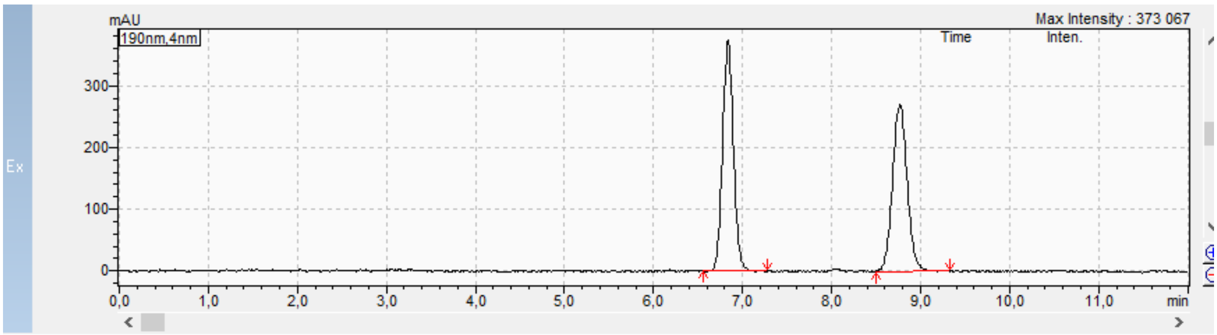

Results View - Peak Table

Peak Table | Compound | Group | Calibration Curve

Peak#	Ret. Time	Area	Height	Mark	Conc.	Unit	ID#	Name	Area%
1	6,834	3150025	373284	M	50,614				50,614
2	8,766	3073619	270333	M	49,386				49,386
Total		6223644	643617		100,000				100,000

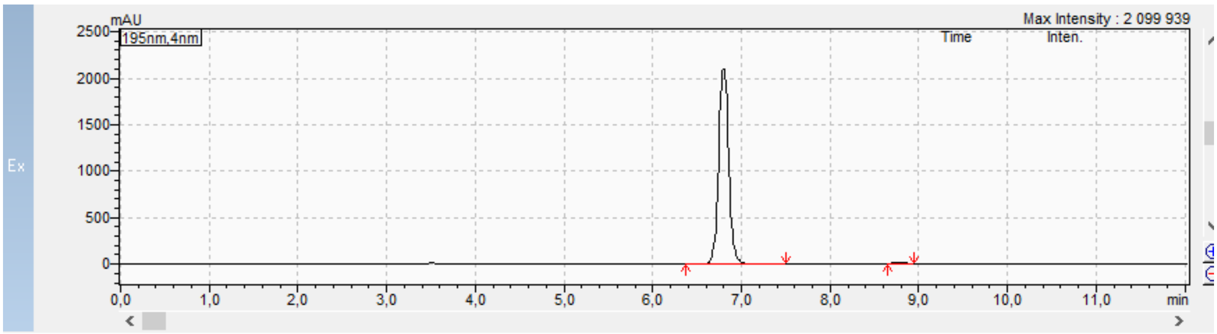

Results View - Peak Table

Peak Table | Compound | Group | Calibration Curve

Peak#	Ret. Time	Area	Height	Mark	Conc.	Unit	ID#	Name	Area%
1	6,799	16978010	2100146	M	98,826				98,826
2	8,779	201751	21158	M	1,174				1,174
Total		17179761	2121305		100,000				100,000

for **3b**: *er* = 99:1 (*ee* = 98%)

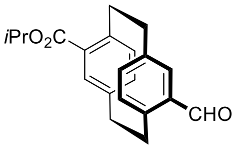

Conditions: IB column
mobile phase: *n*-heptane/*i*-PrOH – 80:20
λ = 190 nm, V = 1.0 ml/min, t = 25 °C
for **3c**: t_R = 5.9 min (major), t_R = 7.6 min (minor)

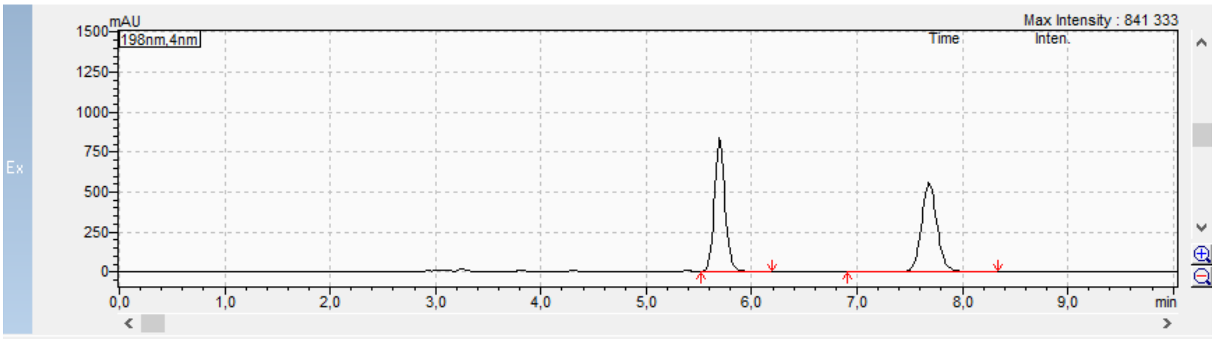

Results View - Peak Table

Peak Table | Compound | Group | Calibration Curve

Peak#	Ret. Time	Area	Height	Mark	Conc.	Unit	ID#	Name	Area%
1	5,696	5858990	839437	M	50,689				50,689
2	7,683	5699726	555156	M	49,311				49,311
Total		11558716	1394592		100,000				100,000

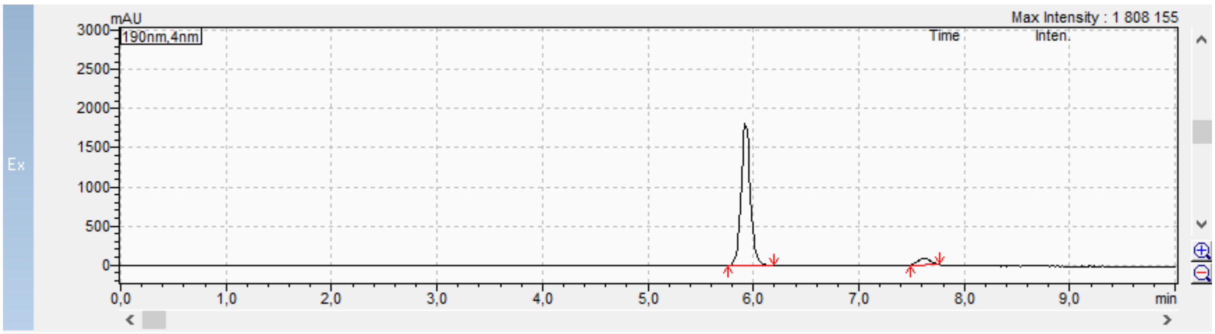

Results View - Peak Table

Peak Table | Compound | Group | Calibration Curve

Peak#	Ret. Time	Area	Height	Mark	Conc.	Unit	ID#	Name	Area%
1	5,924	11369030	1816325	M	94,041				94,041
2	7,613	720458	83786	M	5,959				5,959
Total		12089488	1900111		100,000				100,000

for **3c**: *er* = 94:6 (*ee* = 88%)

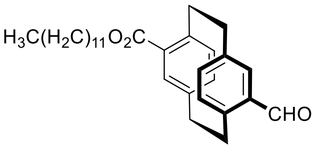

Conditions: IB column
mobile phase: *n*-heptane/*i*-PrOH – 80:20
λ = 190 nm, V = 1.0 ml/min, t = 25 °C
for **3d**: t_R = 5.3 min (major), t_R = 6.3 min (minor)

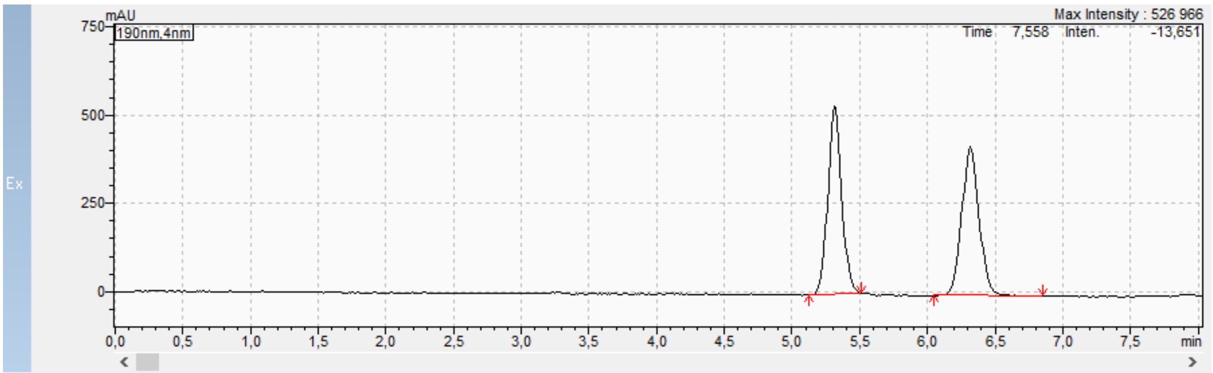

Results View - Peak Table

Peak Table | Compound | Group | Calibration Curve

Peak#	Ret. Time	Area	Height	Mark	Conc.	Unit	ID#	Name	Area%
1	5,315	3837165	534261	M	50,774				50,774
2	6,316	3720228	420466	M	49,226				49,226
Total		7557393	954727		100,000				100,000

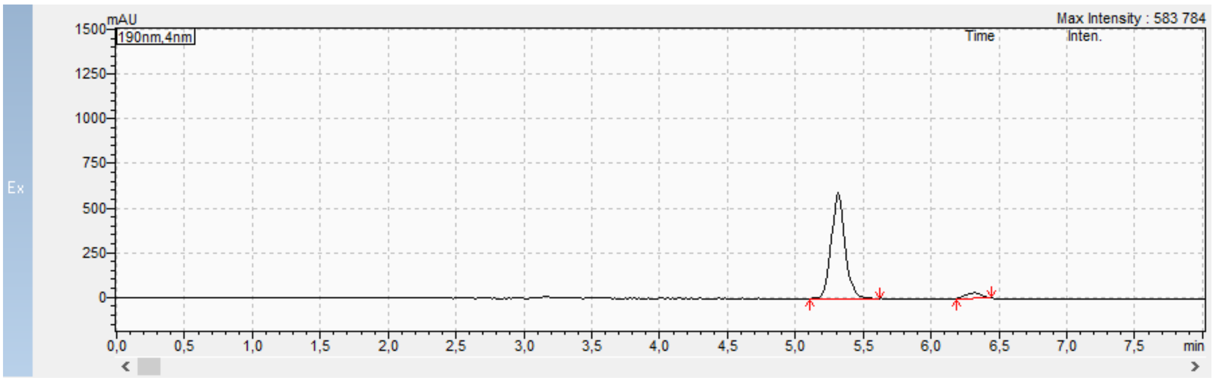

Results View - Peak Table

Peak Table | Compound | Group | Calibration Curve

Peak#	Ret. Time	Area	Height	Mark	Conc.	Unit	ID#	Name	Area%
1	5,315	4162731	590955	M	94,381				94,381
2	6,319	247832	30635	M	5,619				5,619
Total		4410562	621590		100,000				100,000

for **3d**: *er* = 94:6 (*ee* = 89%)

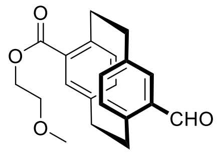

Conditions: IB column
mobile phase: *n*-heptane/*i*-PrOH – 80:20
λ = 190 nm, V = 1.0 ml/min, t = 25 °C
for **3e**: t_R = 9.7 min (major), t_R = 11.1 min (minor)

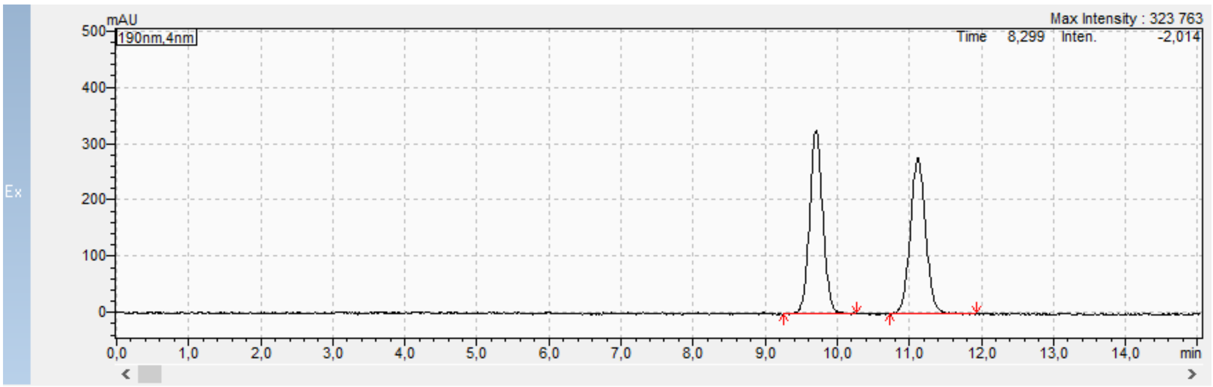

Results View - Peak Table

Peak Table | Compound | Group | Calibration Curve

Peak#	Ret. Time	Area	Height	Mark	Conc.	Unit	ID#	Name	Area%
1	9,708	4123910	325860	M	49,999				49,999
2	11,122	4124104	277182	M	50,001				50,001
Total		8248014	603042		100,000				100,000

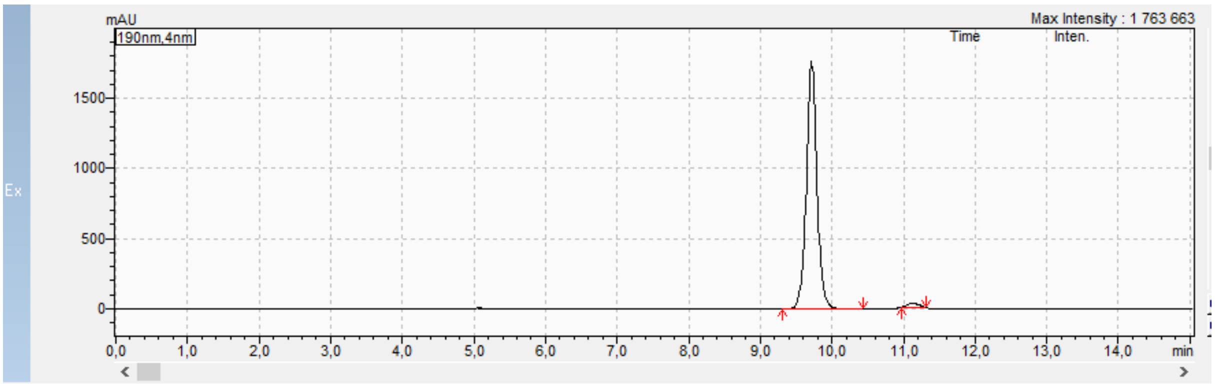

Results View - Peak Table

Peak Table | Compound | Group | Calibration Curve

Peak#	Ret. Time	Area	Height	Mark	Conc.	Unit	ID#	Name	Area%
1	9,717	18138056	1764032	M	97,962				97,962
2	11,119	377391	32230	M	2,038				2,038
Total		18515447	1796262		100,000				100,000

for **3e**: *er* = 98:2 (*ee* = 96%)

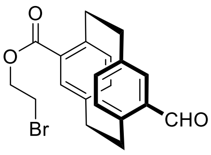

Conditions: IB column
mobile phase: *n*-heptane/*i*-PrOH – 80:20
λ = 244 nm, V = 1.0 ml/min, t = 25 °C
for **3f**: t_R = 10.2 min (major), t_R = 12.6 min (minor)

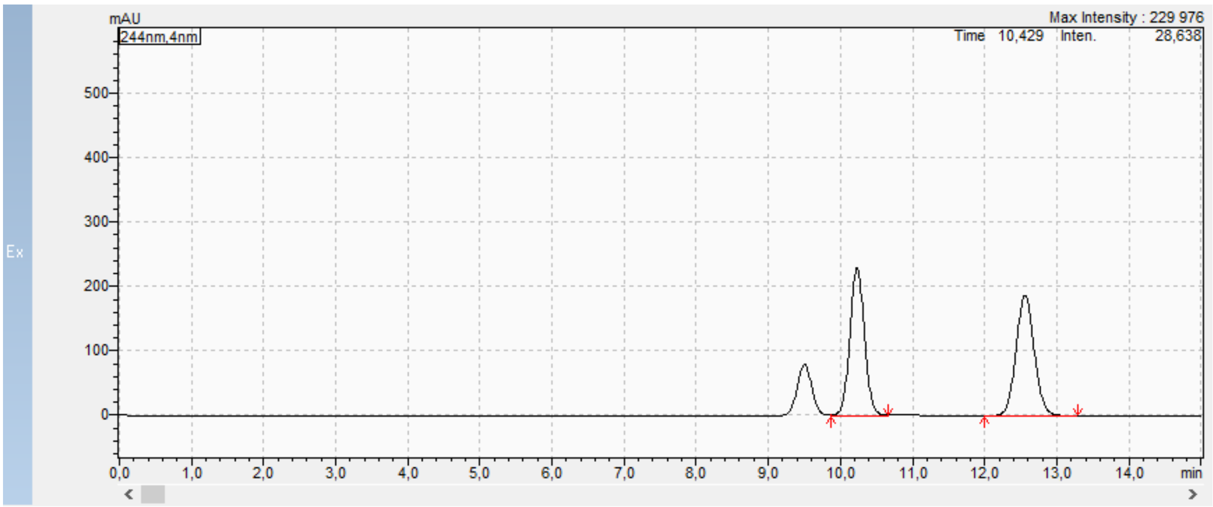

Results View - Peak Table

Peak Table | Compound | Group | Calibration Curve

Peak#	Ret. Time	Area	Height	Peak Start	Peak End	Mark	Conc.	Unit	Area%
1	10,231	3242491	230522	9,867	10,667		49,666		49,666
2	12,559	3286107	187069	12,000	13,291		50,334		50,334
Total		6528598	417591				100,000		100,000

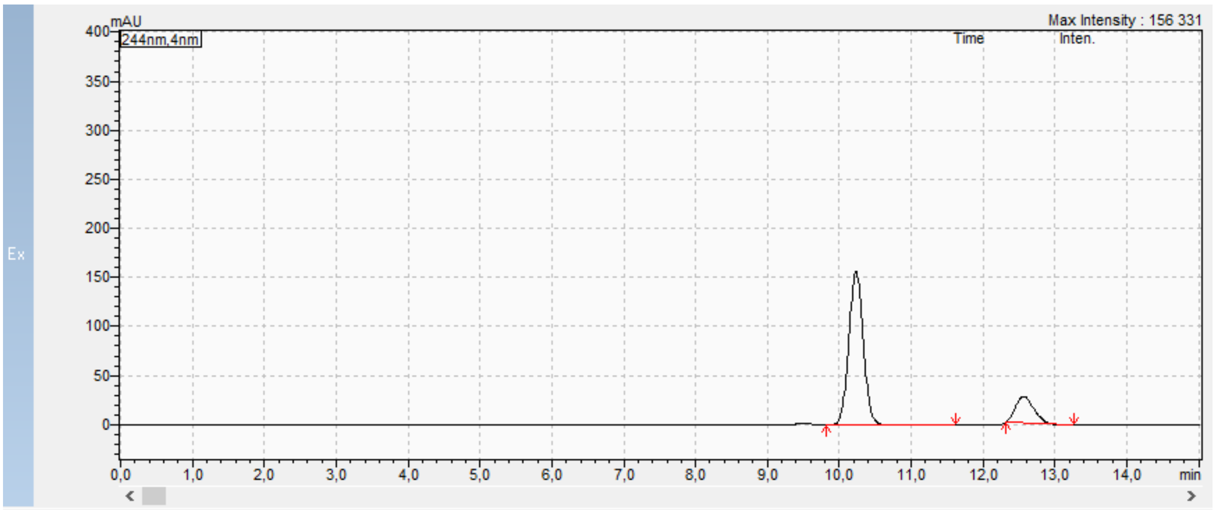

Results View - Peak Table

Peak Table | Compound | Group | Calibration Curve

Peak#	Ret. Time	Area	Height	Peak Start	Peak End	Mark	Conc.	Unit	Area%
1	10,231	2195565	156914	9,813	11,616	M	82,438		82,438
2	12,563	467719	26931	12,320	13,259	M	17,562		17,562
Total		2663284	183845				100,000		100,000

for **3f**: *er* = 82:18 (*ee* = 65%)

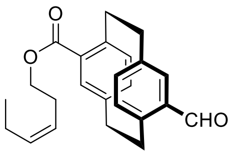

Conditions: IB column
mobile phase: *n*-heptane/*i*-PrOH – 80:20
λ = 190 nm, V = 1.0 ml/min, t = 25 °C
for **3g**: t_R = 6.1 min (major), t_R = 7.3 min (minor).

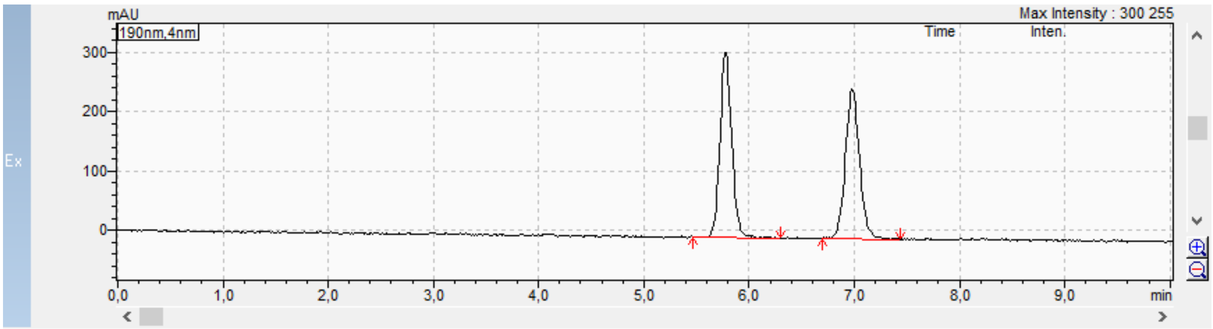

Results View - Peak Table

Peak Table | Compound | Group | Calibration Curve

Peak#	Ret. Time	Area	Height	Mark	Conc.	Unit	ID#	Name	Area%
1	5,776	2390798	311690	M	50,358				50,358
2	6,981	2356761	250734	M	49,642				49,642
Total		4747559	562424		100,000				100,000

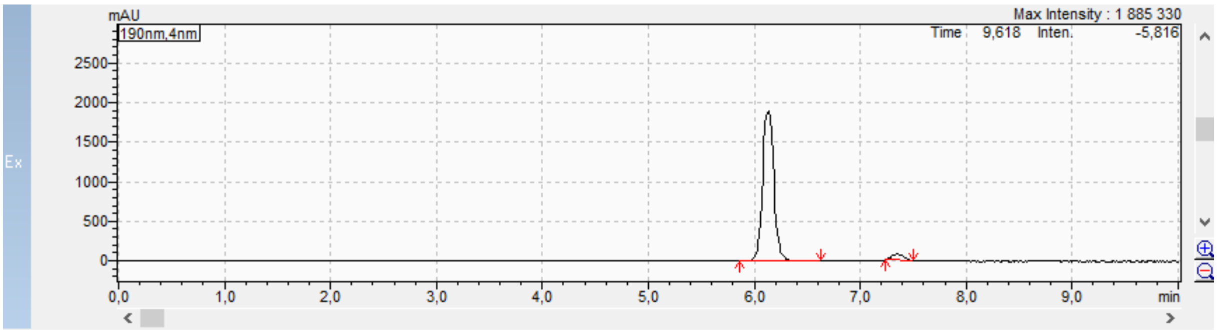

Results View - Peak Table

Peak Table | Compound | Group | Calibration Curve

Peak#	Ret. Time	Area	Height	Mark	Conc.	Unit	ID#	Name	Area%
1	6,139	14309801	1888926	M	96,030				96,030
2	7,347	591616	74236	M	3,970				3,970
Total		14901417	1963163		100,000				100,000

for **3g**: *er* = 96:4 (*ee* = 92%)

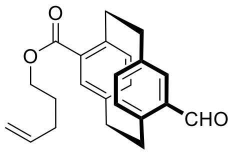

Conditions: IB column
mobile phase: *n*-heptane/*i*-PrOH – 80:20
λ = 190 nm, V = 1.0 ml/min, t = 25 °C
for **3h**: t_R = 6.5 min (major), t_R = 7.7 min (minor)

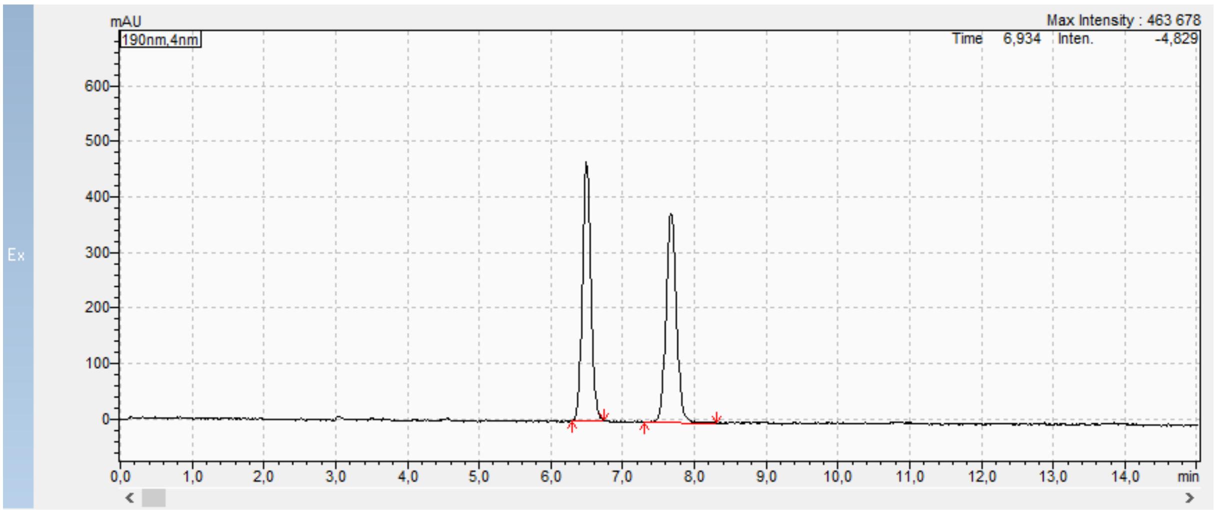

Results View - Peak Table

Peak Table | Compound | Group | Calibration Curve

Peak#	Ret. Time	Area	Height	Peak Start	Peak End	Mark	Conc.	Unit	Area%
1	6,495	3799710	466508	6,293	6,741	M	50,149		50,149
2	7,674	3777114	374823	7,307	8,309	M	49,851		49,851
Total		7576824	841330				100,000		100,000

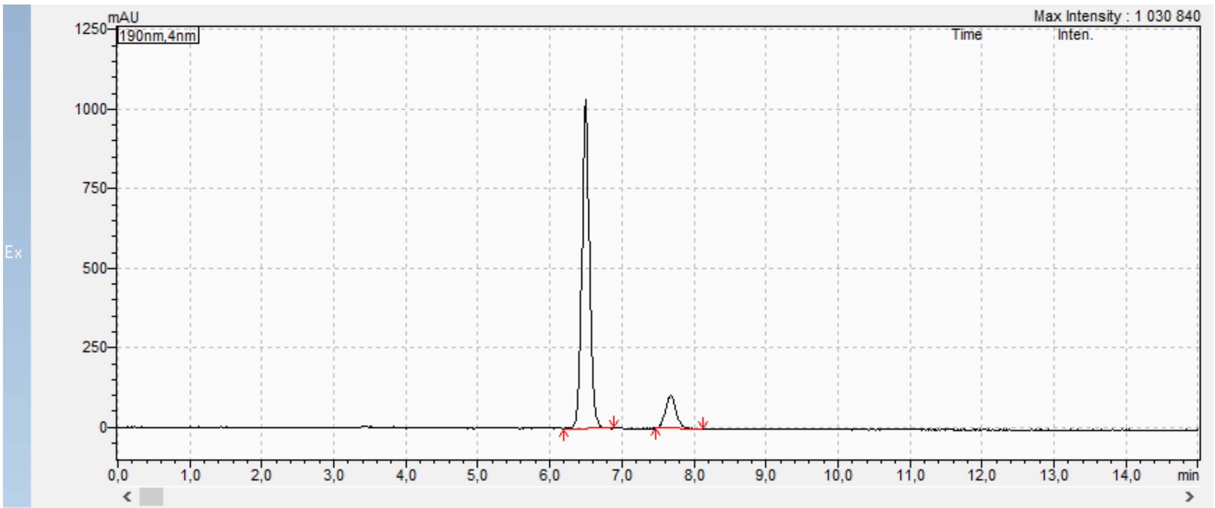

Results View - Peak Table

Peak Table | Compound | Group | Calibration Curve

Peak#	Ret. Time	Area	Height	Peak Start	Peak End	Mark	Conc.	Unit	Area%
1	6,497	7528464	1034039	6,187	6,891	M	88,230		88,230
2	7,677	1004294	100951	7,477	8,128	M	11,770		11,770
Total		8532758	1134990				100,000		100,000

for **3h**: *er* = 88:12 (*ee* = 77%)

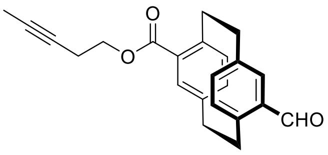

Conditions: IB column
mobile phase: *n*-heptane/*i*-PrOH – 80:20
λ = 190 nm, V = 1.0 ml/min, t = 25 °C
for **3i**: t_R = 7.4 min (major), t_R = 9.4 min (minor)

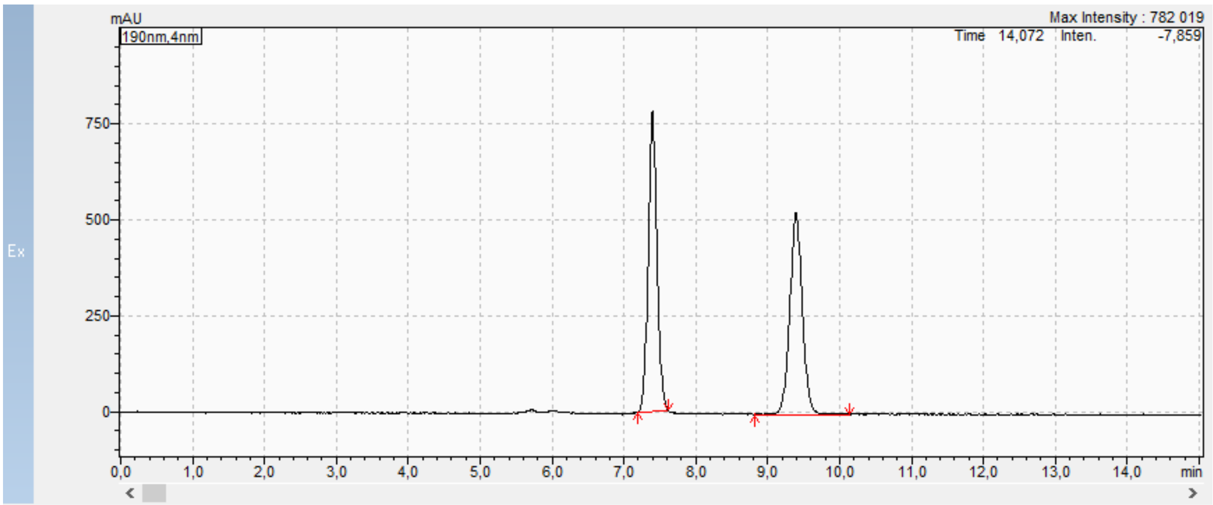

Results View - Peak Table

Peak Table | Compound | Group | Calibration Curve

Peak#	Ret. Time	Area	Height	Peak Start	Peak End	Mark	Conc.	Unit	Area%
1	7,398	6574889	780692	7,189	7,616	M	50,459		50,459
2	9,393	6455170	526971	8,821	10,133	M	49,541		49,541
Total		13030059	1307664				100,000		100,000

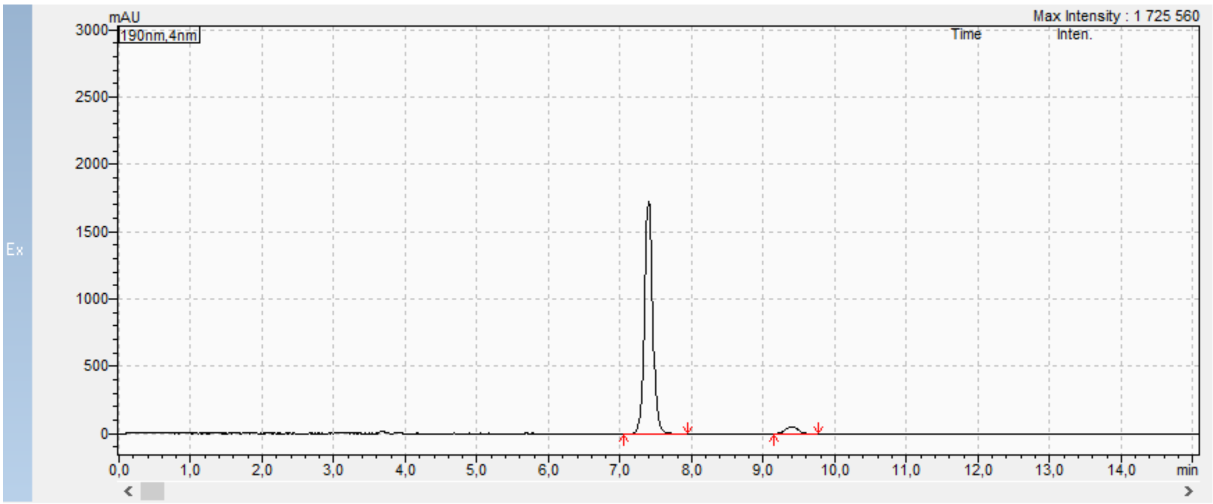

Results View - Peak Table

Peak Table | Compound | Group | Calibration Curve

Peak#	Ret. Time	Area	Height	Peak Start	Peak End	Mark	Conc.	Unit	Area%
1	7,400	13800161	1728050	7,051	7,947	M	95,604		95,604
2	9,396	634516	53340	9,141	9,771	M	4,396		4,396
Total		14434676	1781390				100,000		100,000

for **3i**: *er* = 96:4 (*ee* = 91%)

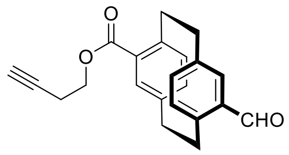

Conditions: IB column
mobile phase: *n*-heptane/*i*-PrOH – 80:20
λ = 199 nm, V = 1.0 ml/min, t = 25 °C
for **3j**: t_R = 8.9 min (major), t_R = 11.5 min (minor)

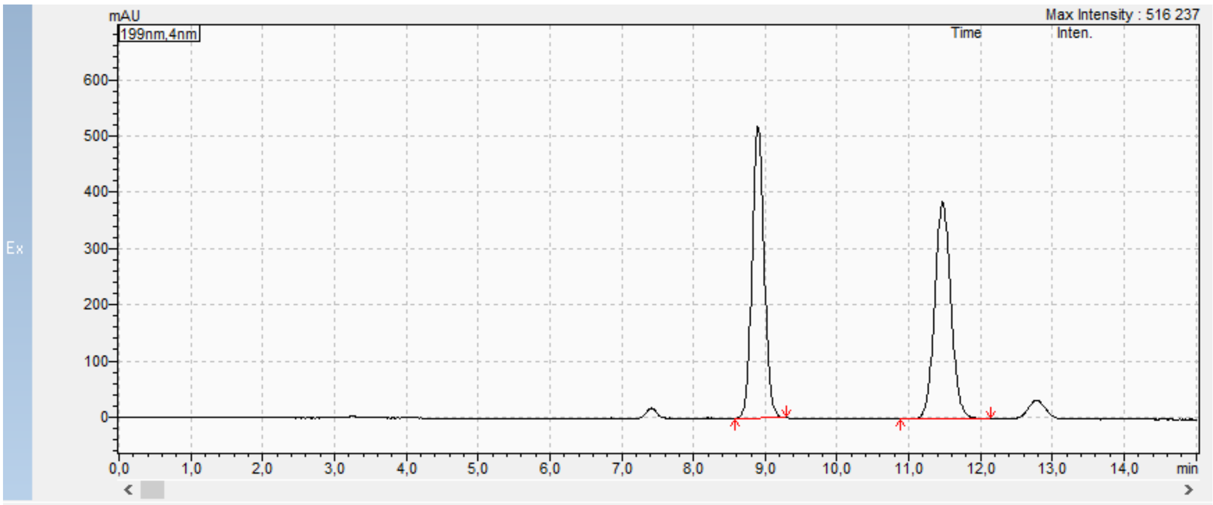

Results View - Peak Table

Peak Table | Compound | Group | Calibration Curve

Peak#	Ret. Time	Area	Height	Peak Start	Peak End	Mark	Conc.	Unit	Area%
1	8,899	6075873	517541	8,576	9,301	M	50,077		50,077
2	11,471	6057240	384913	10,891	12,149	M	49,923		49,923
Total		12133113	902454				100,000		100,000

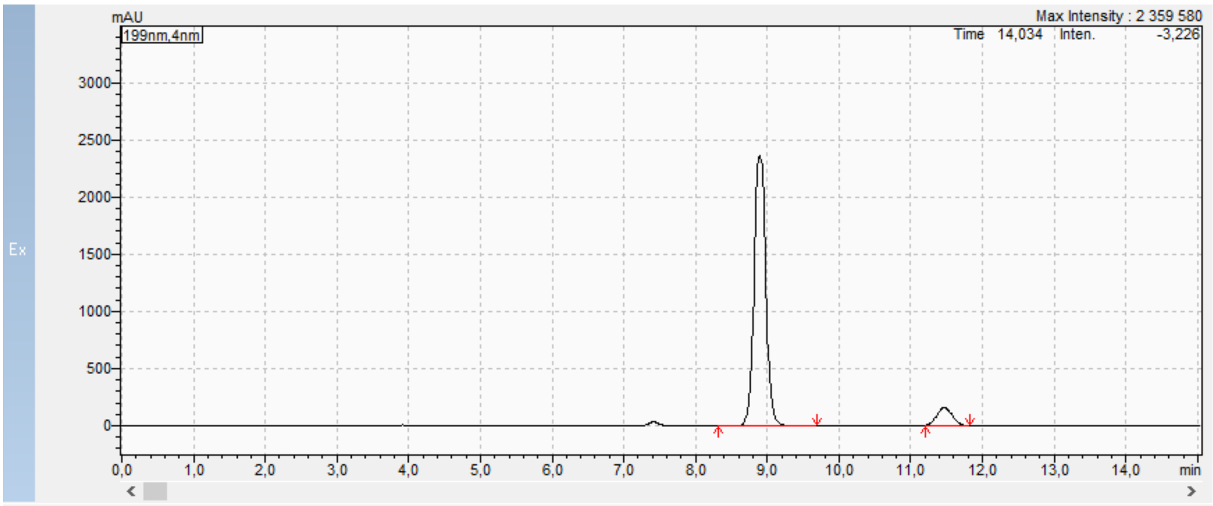

Results View - Peak Table

Peak Table | Compound | Group | Calibration Curve

Peak#	Ret. Time	Area	Height	Peak Start	Peak End	Mark	Conc.	Unit	Area%
1	8,894	27187074	2361677	8,309	9,685	M	92,061		92,061
2	11,468	2344410	155256	11,211	11,829	M	7,939		7,939
Total		29531484	2516933				100,000		100,000

for **3j**: *er* = 92:8 (*ee* = 84%)

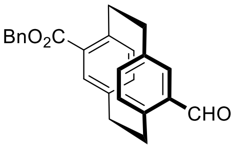

Conditions: IB column
mobile phase: *n*-heptane/*i*-PrOH – 80:20
λ = 190 nm, V = 1.0 ml/min, t = 25 °C
for **3k**: t_R = 8.5 min (major), t_R = 10.2 min (minor)

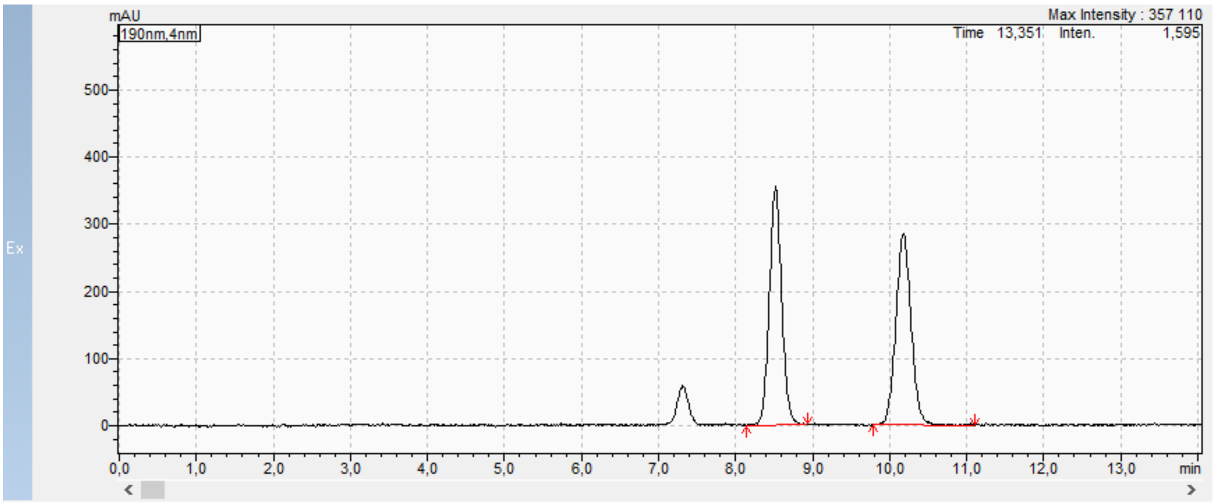

Results View - Peak Table

Peak Table | Compound | Group | Calibration Curve

Peak#	Ret. Time	Area	Height	Peak Start	Peak End	Mark	Conc.	Unit	Area%
1	8,520	3952869	355664	8,139	8,928	M	50,284		50,284
2	10,180	3908284	284792	9,781	11,104	M	49,716		49,716
Total		7861153	640455				100,000		100,000

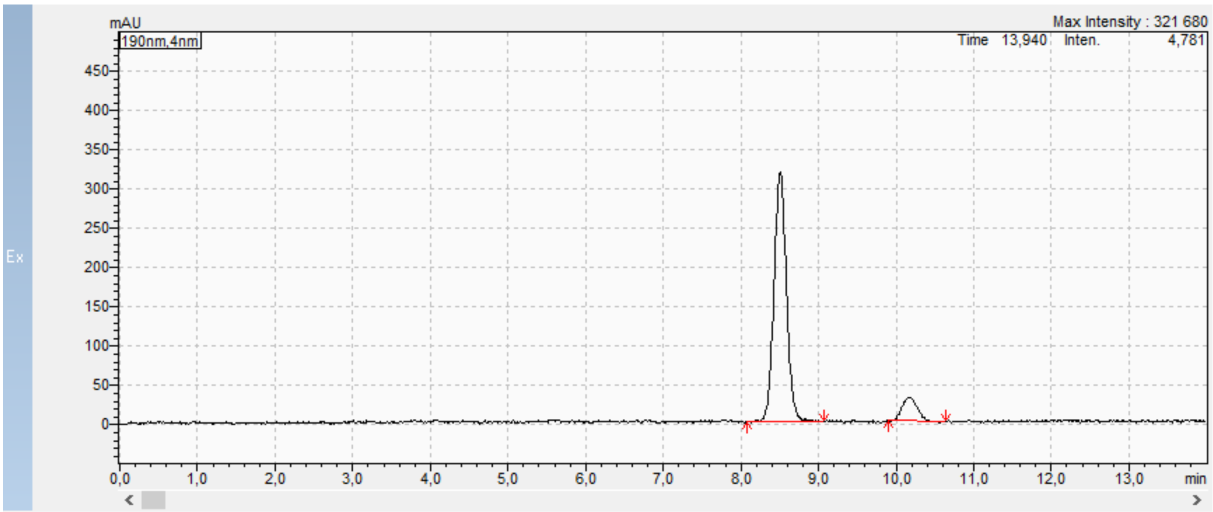

Results View - Peak Table

Peak Table | Compound | Group | Calibration Curve

Peak#	Ret. Time	Area	Height	Peak Start	Peak End	Mark	Conc.	Unit	Area%
1	8,507	3541808	317743	8,085	9,077	M	89,872		89,872
2	10,169	399126	29971	9,909	10,645	M	10,128		10,128
Total		3940934	347714				100,000		100,000

for **3k**: *er* = 90:10 (*ee* = 80%)

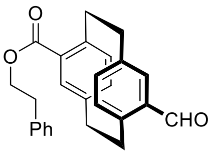

Conditions: IB column
mobile phase: *n*-heptane/*i*-PrOH – 80:20
λ = 190 nm, V = 1.0 ml/min, t = 25 °C
for **3l**: t_R = 9.4 min (major), t_R = 11.2 min (minor)

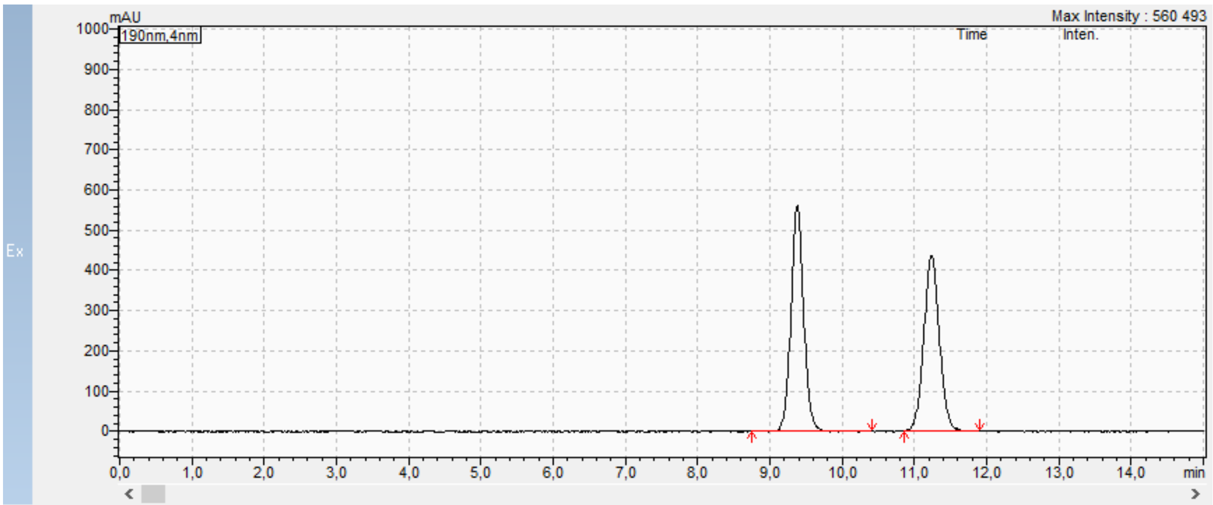

Results View - Peak Table

Peak Table | Compound | Group | Calibration Curve

Peak#	Ret. Time	Area	Height	Peak Start	Peak End	Mark	Conc.	Unit	Area%
1	9,374	6797627	559812	8,747	10,411	M	50,874		50,874
2	11,236	6563993	434660	10,848	11,893	M	49,126		49,126
Total		13361620	994472				100,000		100,000

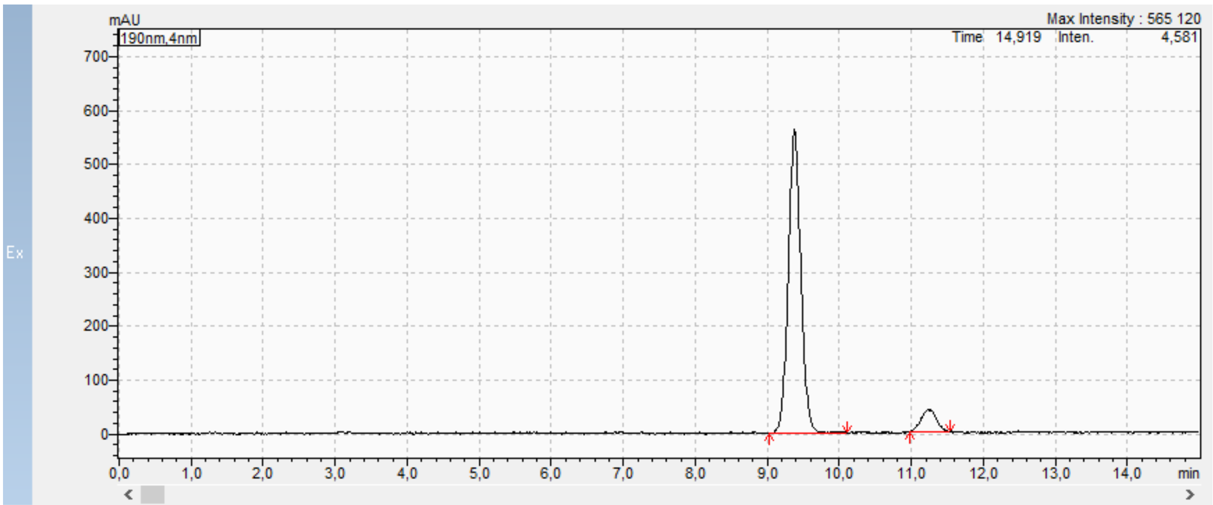

Results View - Peak Table

Peak Table | Compound | Group | Calibration Curve

Peak#	Ret. Time	Area	Height	Peak Start	Peak End	Mark	Conc.	Unit	Area%
1	9,374	6822289	562837	9,024	10,112	M	91,718		91,718
2	11,234	616073	41060	10,976	11,541	M	8,282		8,282
Total		7438362	603898				100,000		100,000

for **3l**: *er* = 92:8 (*ee* = 83%)

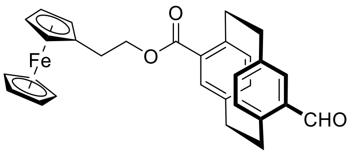

Conditions: IB column
mobile phase: *n*-heptane/*i*-PrOH – 80:20
λ = 194 nm, V = 1.0 ml/min, t = 25 °C
for **3m**: t_R = 13.0 min (major), t_R = 15.2 min (minor)

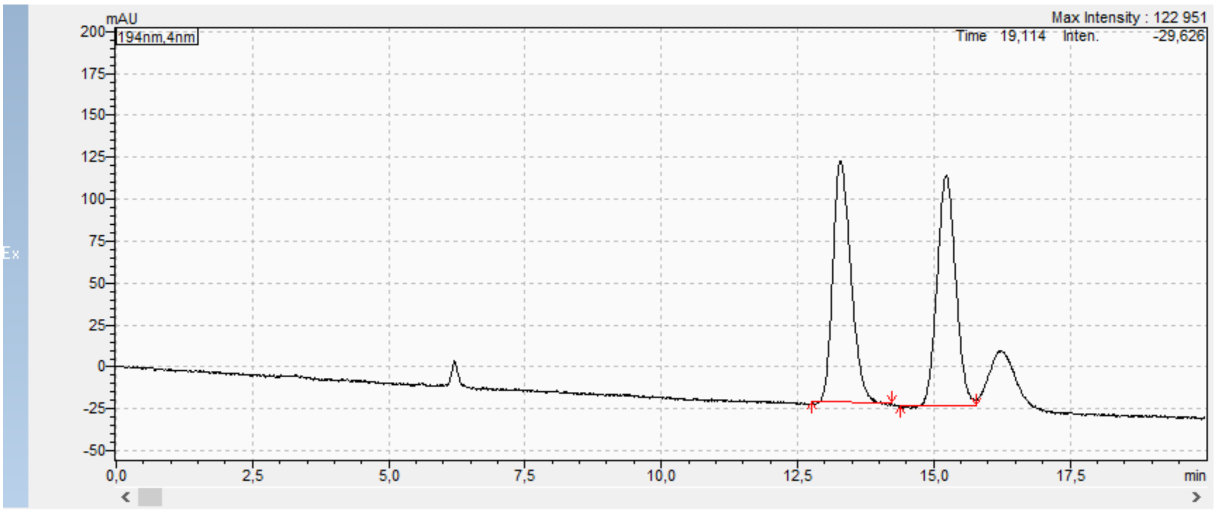

Results View - Peak Table

Peak Table | Compound | Group | Calibration Curve

Peak#	Ret. Time	Area	Height	Peak Start	Peak End	Mark	Conc.	Unit	Area%
1	13,289	3273371	143908	12,757	14,229	M	50,450		50,450
2	15,238	3214919	136855	14,400	15,776	M	49,550		49,550
Total		6488289	280763				100,000		100,000

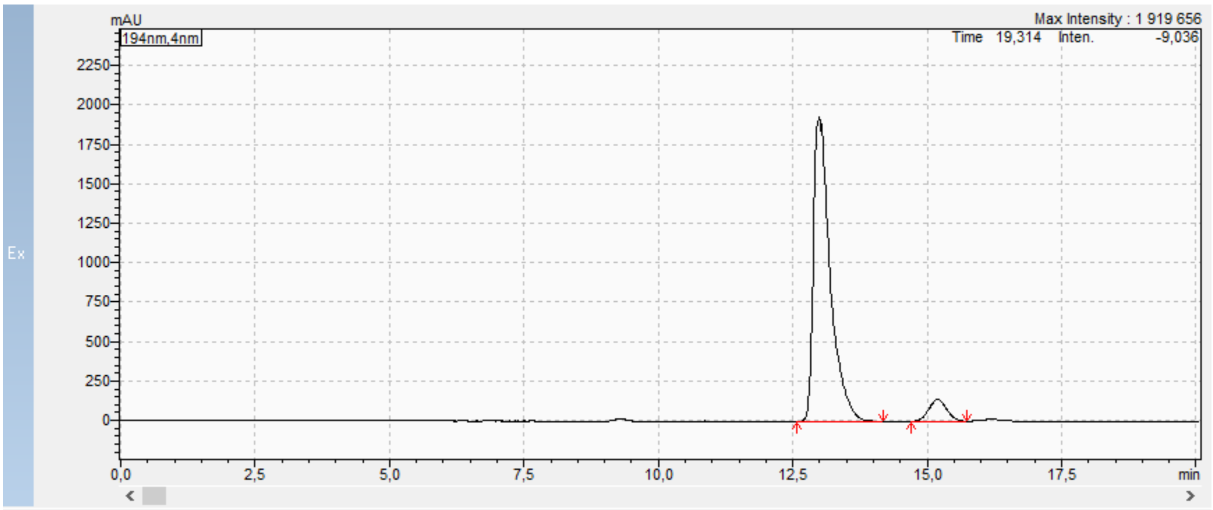

Results View - Peak Table

Peak Table | Compound | Group | Calibration Curve

Peak#	Ret. Time	Area	Height	Peak Start	Peak End	Mark	Conc.	Unit	Area%
1	12,988	41899137	1926402	12,576	14,187	M	92,863		92,863
2	15,192	3219947	138954	14,709	15,733	M	7,137		7,137
Total		45119084	2065357				100,000		100,000

for **3m**: *er* = 93:7 (*ee* = 86%)

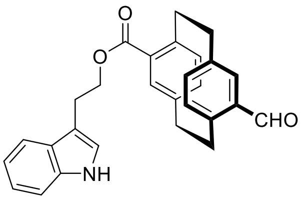

Conditions: IB column
mobile phase: *n*-heptane/*i*-PrOH – 60:40
λ = 190 nm, V = 1.0 ml/min, t = 25 °C
for **3n**: t_R = 14.0 min (major), t_R = 18.9 min (minor)

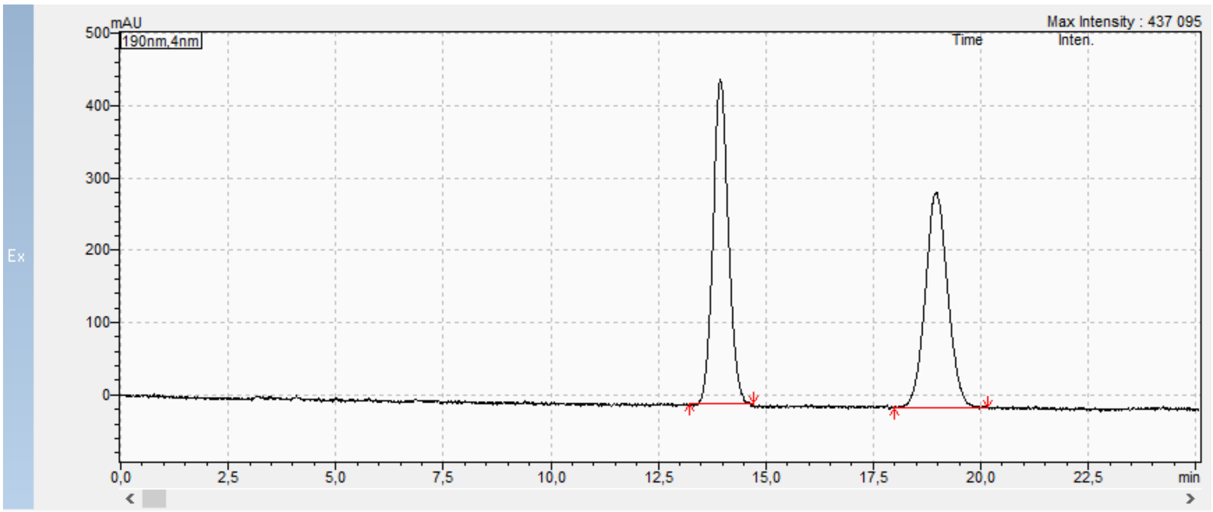

Results View - Peak Table

Peak Table | Compound | Group | Calibration Curve

Peak#	Ret. Time	Area	Height	Peak Start	Peak End	Mark	Conc.	Unit	Area%
1	13,940	11092282	449296	13,227	14,720	M	50,364		50,364
2	18,986	10931732	298874	17,973	20,149	M	49,636		49,636
Total		22024014	748170				100,000		100,000

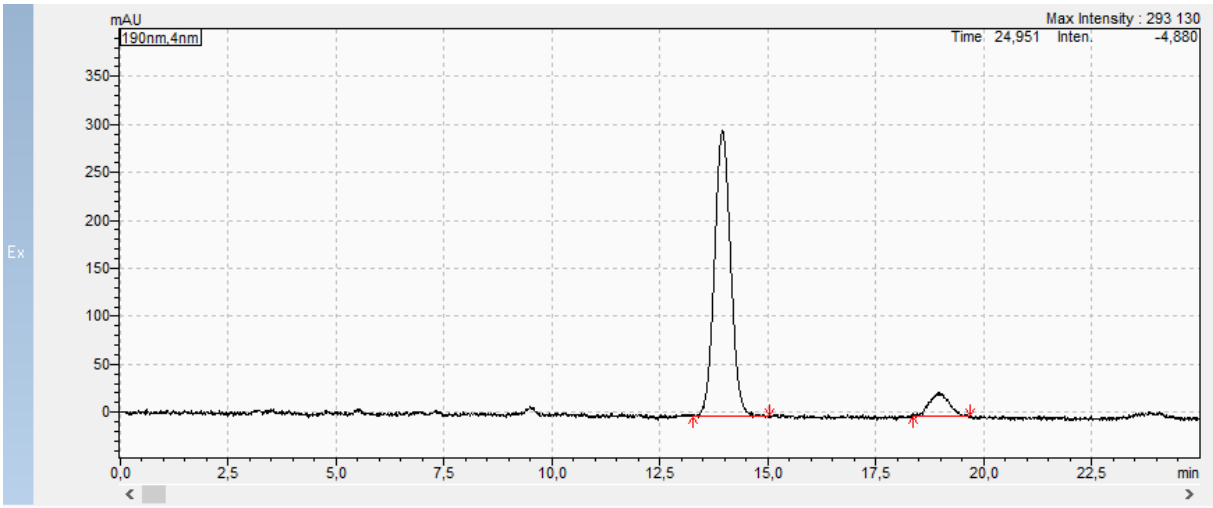

Results View - Peak Table

Peak Table | Compound | Group | Calibration Curve

Peak#	Ret. Time	Area	Height	Peak Start	Peak End	Mark	Conc.	Unit	Area%
1	13,952	7693759	296887	13,259	15,051	M	91,140		91,140
2	18,931	747891	24293	18,379	19,701	M	8,860		8,860
Total		8441651	321180				100,000		100,000

for **3n**: *er* = 91:9 (*ee* = 82%)

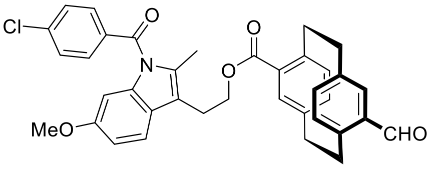

Conditions: IB column
mobile phase: *n*-heptane/*i*-PrOH – 50:50
λ = 209 nm, V = 1.0 ml/min, t = 25 °C
for **3o**: t_R = 20.3 min (major), t_R = 46.9 min (minor)

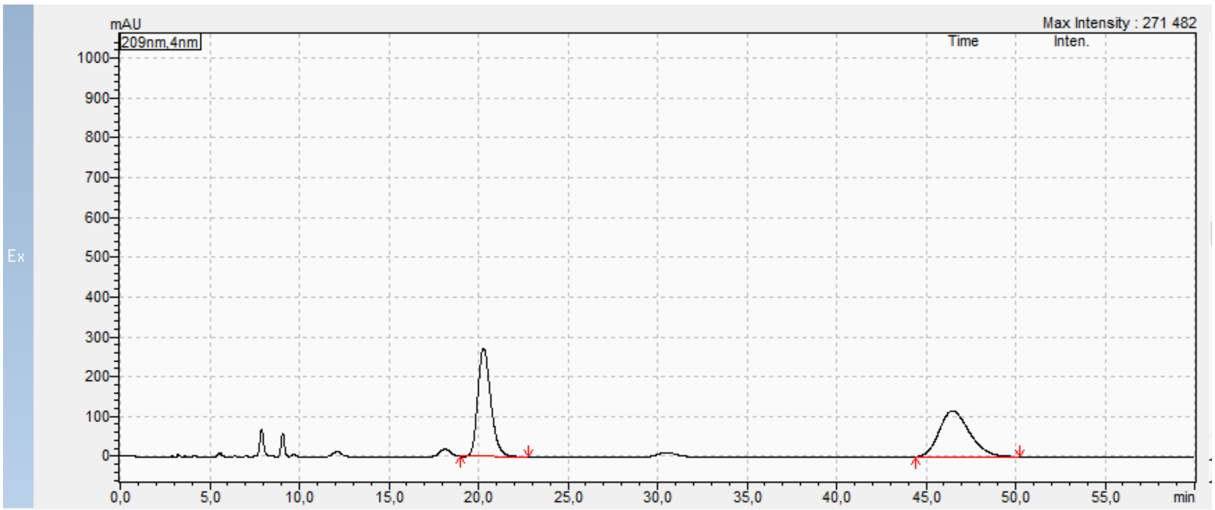

Results View - Peak Table

Peak Table | Compound | Group | Calibration Curve

Peak#	Ret. Time	Area	Height	Peak Start	Peak End	Mark	Conc.	Unit	Area%
1	20,286	13733759	272153	18,997	22,795	M	50,183		50,183
2	46,463	13633466	115125	44,416	50,229	M	49,817		49,817
Total		27367225	387278				100,000		100,000

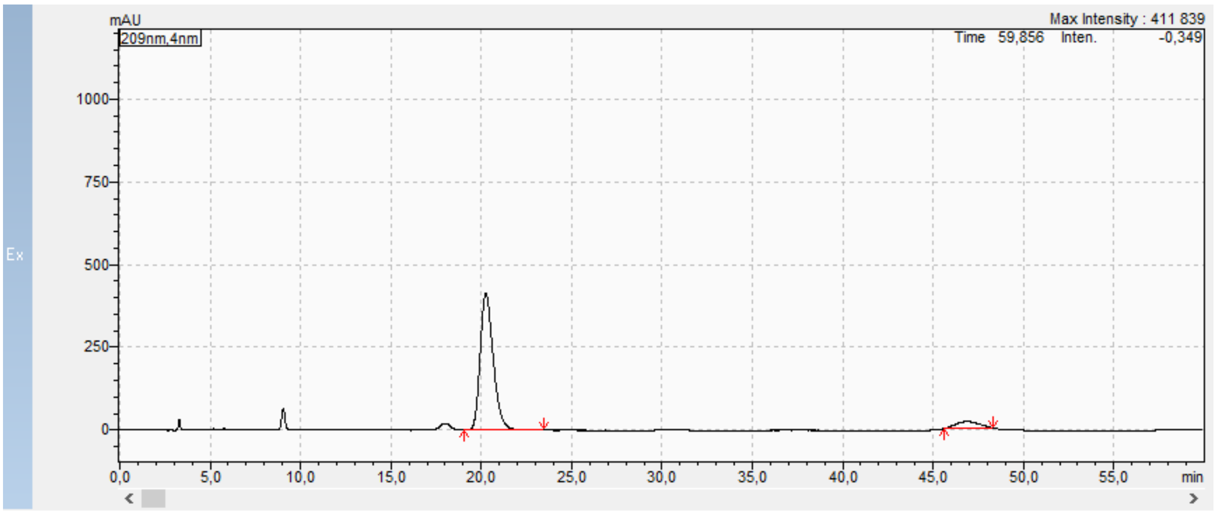

Results View - Peak Table

Peak Table | Compound | Group | Calibration Curve

Peak#	Ret. Time	Area	Height	Peak Start	Peak End	Mark	Conc.	Unit	Area%
1	20,250	20842156	412384	19,072	23,488	M	91,833		91,833
2	46,858	1853674	20233	45,589	48,299	M	8,167		8,167
Total		22695830	432617				100,000		100,000

for **3o**: *er* = 92:8 (*ee* = 84%)

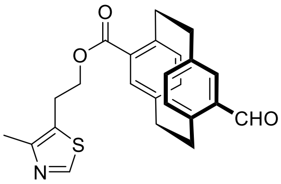

Conditions: IB column
mobile phase: *n*-heptane/*i*-PrOH – 60:40
λ = 197 nm, V = 1.0 ml/min, t = 25 °C
for **3p**: t_R = 13.6 min (major), t_R = 14.9 min (minor)

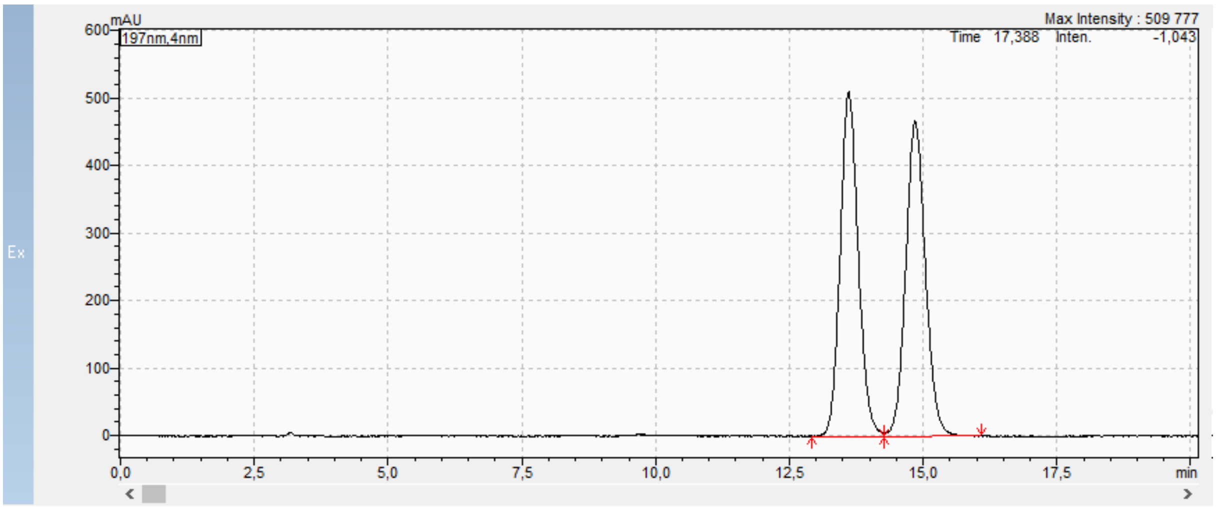

Results View - Peak Table

Peak Table | Compound | Group | Calibration Curve

Peak#	Ret. Time	Area	Height	Peak Start	Peak End	Mark	Conc.	Unit	Area%
1	13,608	12019640	511324	12,907	14,272	M	50,114		50,114
2	14,848	11964922	467172	14,272	16,085	M	49,886		49,886
Total		23984562	978495				100,000		100,000

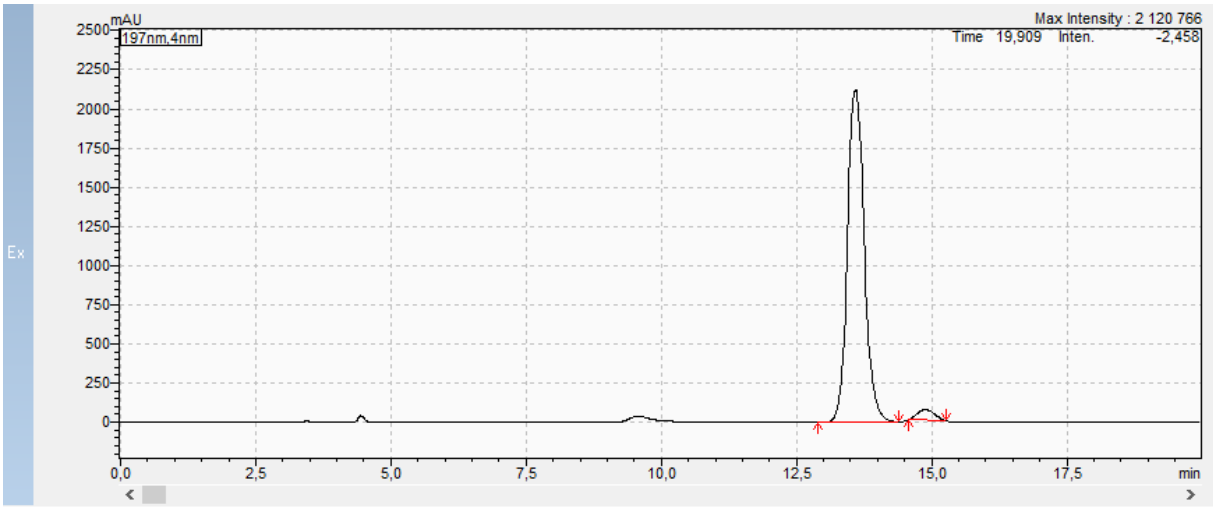

Results View - Peak Table

Peak Table | Compound | Group | Calibration Curve

Peak#	Ret. Time	Area	Height	Peak Start	Peak End	Mark	Conc.	Unit	Area%
1	13,582	46190017	2122895	12,885	14,379		96,818		96,818
2	14,883	1517894	69327	14,571	15,264	M	3,182		3,182
Total		47707912	2192222				100,000		100,000

for **3p**: *er* = 97:3 (*ee* = 94%)

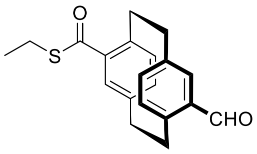

Conditions: IB column
mobile phase: *n*-heptane/*i*-PrOH – 80:20
λ = 190 nm, V = 1.0 ml/min, t = 25 °C
for **3w**: t_R = 6.8 min (major), t_R = 8.3 min (minor)

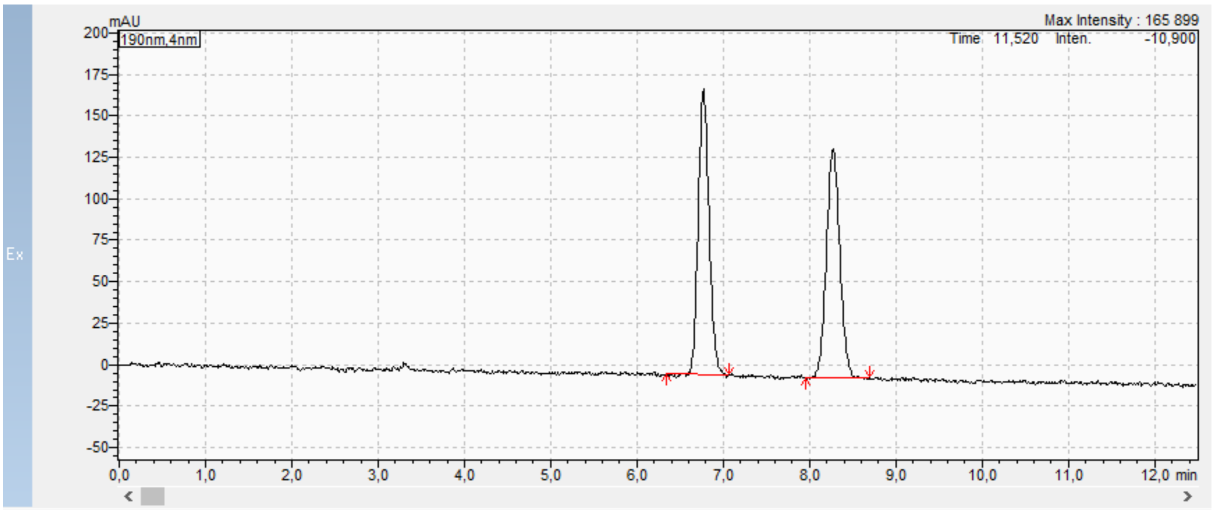

Results View - Peak Table

Peak Table | Compound | Group | Calibration Curve

Peak#	Ret. Time	Area	Height	Peak Start	Peak End	Mark	Conc.	Unit	Area%
1	6,772	1489756	171834	6,347	7,061	M	50,183		50,183
2	8,272	1478901	137719	7,947	8,693	M	49,817		49,817
Total		2968657	309553				100,000		100,000

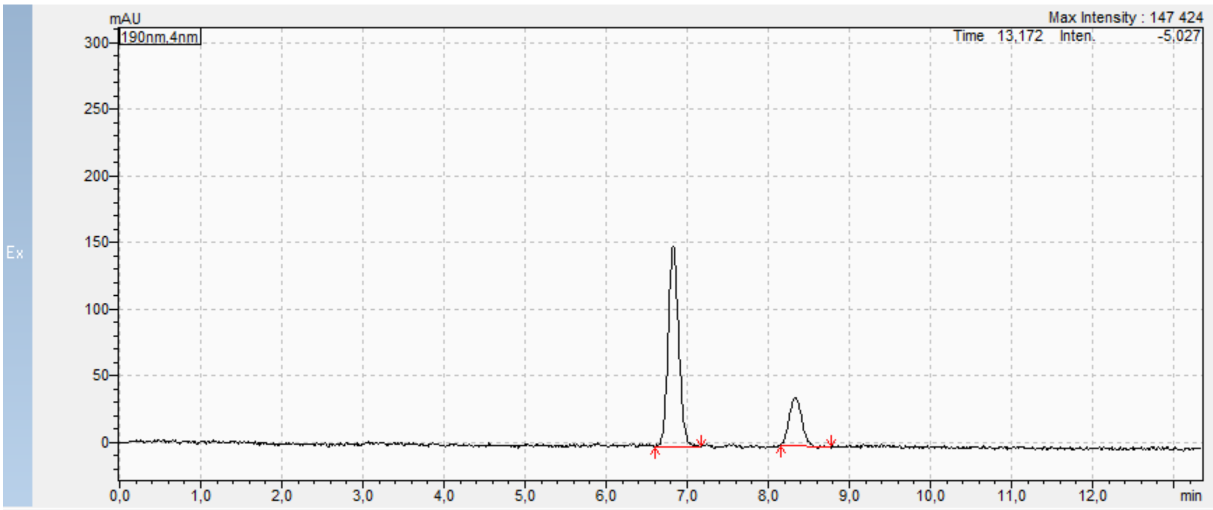

Results View - Peak Table

Peak Table | Compound | Group | Calibration Curve

Peak#	Ret. Time	Area	Height	Peak Start	Peak End	Mark	Conc.	Unit	Area%
1	6,827	1351527	150706	6,603	7,179	M	78,342		78,342
2	8,331	373640	36266	8,160	8,768	M	21,658		21,658
Total		1725166	186972				100,000		100,000

for **3w**: *er* = 78:22 (*ee* = 57%)

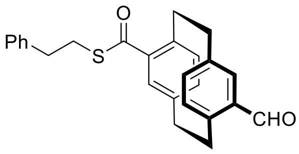

Conditions: IB column
mobile phase: *n*-heptane/*i*-PrOH – 80:20
λ = 190 nm, V = 1.0 ml/min, t = 25 °C
for **3x**: t_R = 9.4 min (major), t_R = 10.9 min (minor)

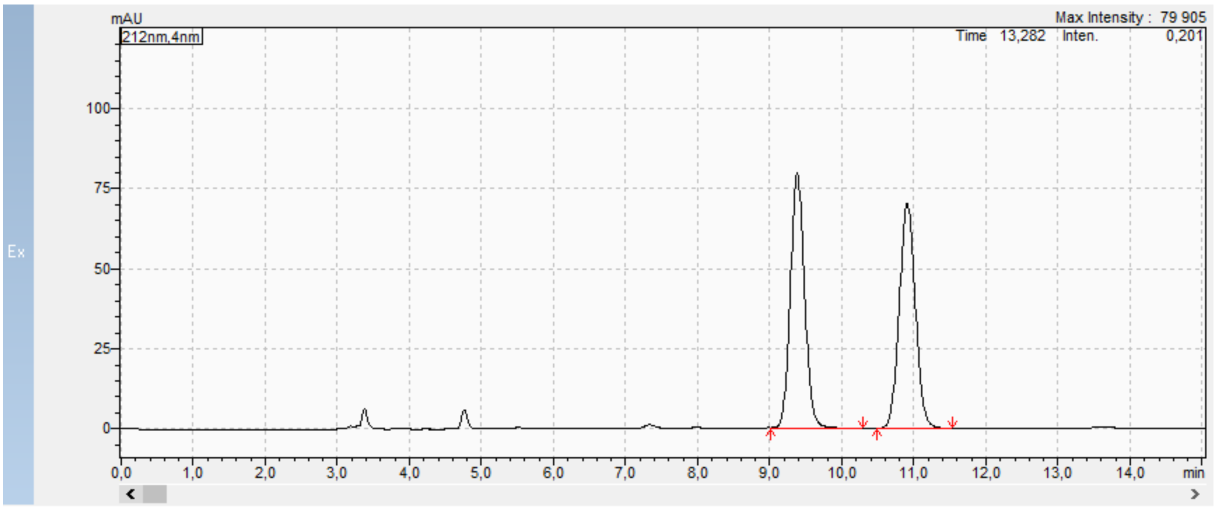

Results View - Peak Table

Peak Table | Compound | Group | Calibration Curve

Peak#	Ret. Time	Area	Height	Peak Start	Peak End	Mark	Conc.	Unit	Area%
1	9,381	1093715	79706	9,013	10,283	S	50,094		50,094
2	10,908	1089620	70161	10,485	11,531	S	49,906		49,906
Total		2183335	149867				100,000		100,000

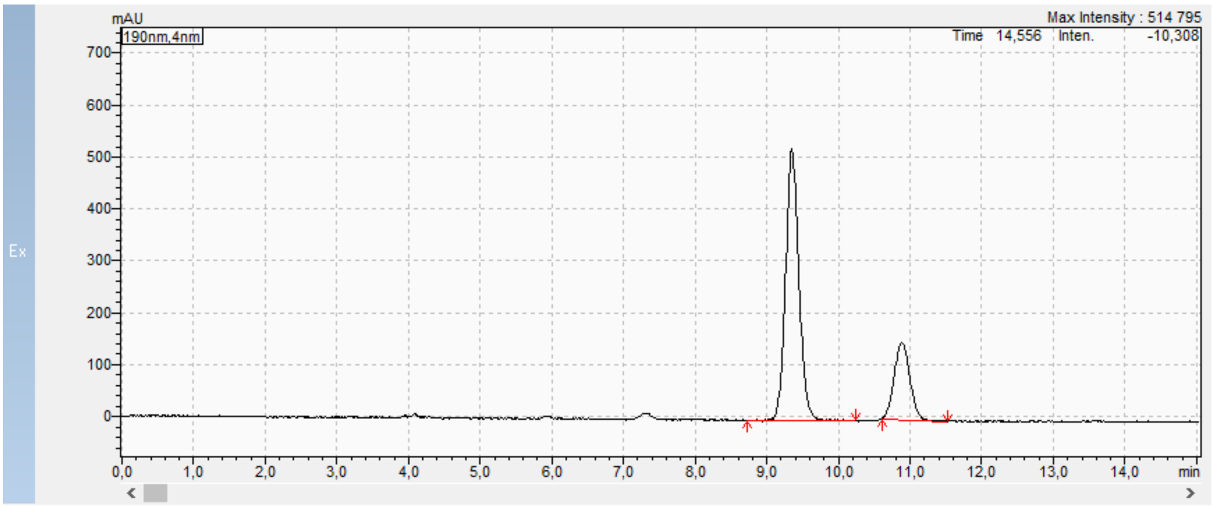

Results View - Peak Table

Peak Table | Compound | Group | Calibration Curve

Peak#	Ret. Time	Area	Height	Peak Start	Peak End	Mark	Conc.	Unit	Area%
1	9,349	6719372	522108	8,725	10,251	M	74,729		74,729
2	10,890	2272221	149846	10,603	11,531	M	25,271		25,271
Total		8991593	671954				100,000		100,000

for **3x**: *er* = 75:25 (*ee* = 50%)

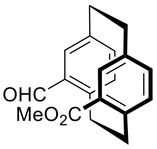

Conditions: IB column
mobile phase: *n*-heptane/*i*-PrOH – 80:20
λ = 190 nm, V = 1.0 ml/min, t = 25 °C
for **5a**: t_R = 9.3 min (major), t_R = 14.3 min (minor).

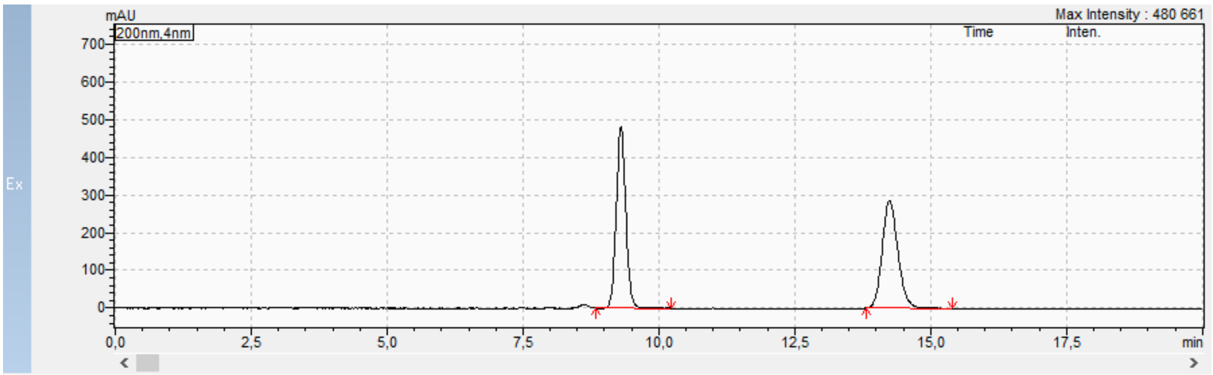

Results View - Peak Table

Peak Table | Compound | Group | Calibration Curve

Peak#	Ret. Time	Area	Height	Mark	Conc.	Unit	ID#	Name	Area%
1	9,301	5844742	481017	M	50,495				50,495
2	14,242	5730224	285783	M	49,505				49,505
Total		11574967	766800		100,000				100,000

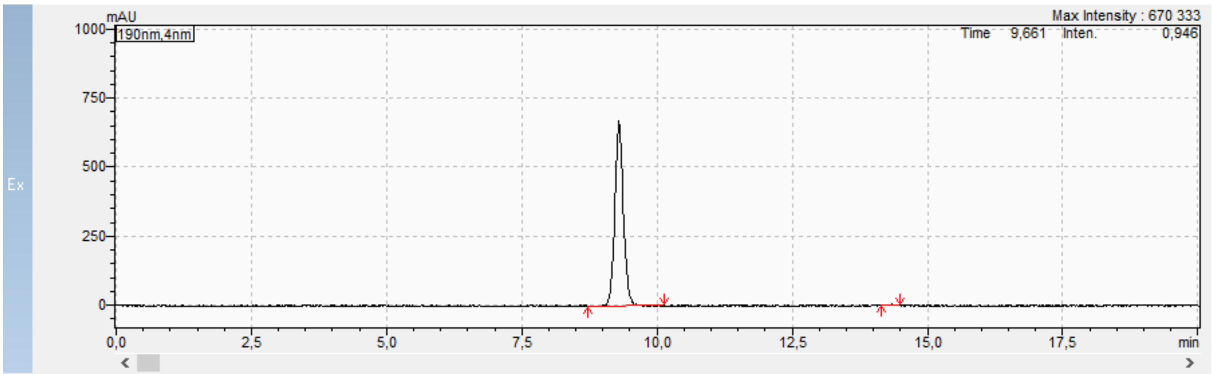

Results View - Peak Table

Peak Table | Compound | Group | Calibration Curve

Peak#	Ret. Time	Area	Height	Mark	Conc.	Unit	ID#	Name	Area%
1	9,300	7232448	671695	M	99,607				99,607
2	14,343	28564	3449	M	0,393				0,393
Total		7261011	675144		100,000				100,000

for **5a**: *er* = 99.5:0.5 (*ee* = 99%)

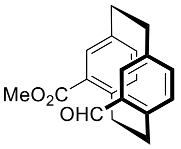

Conditions: IB column
mobile phase: *n*-heptane / *i*-PrOH – 80:20
λ = 190 nm, V = 1.0 ml/min, t = 25 °C
for *ent*-**5a**: t_R = 9.3 min (minor), t_R = 14.3 min (major)

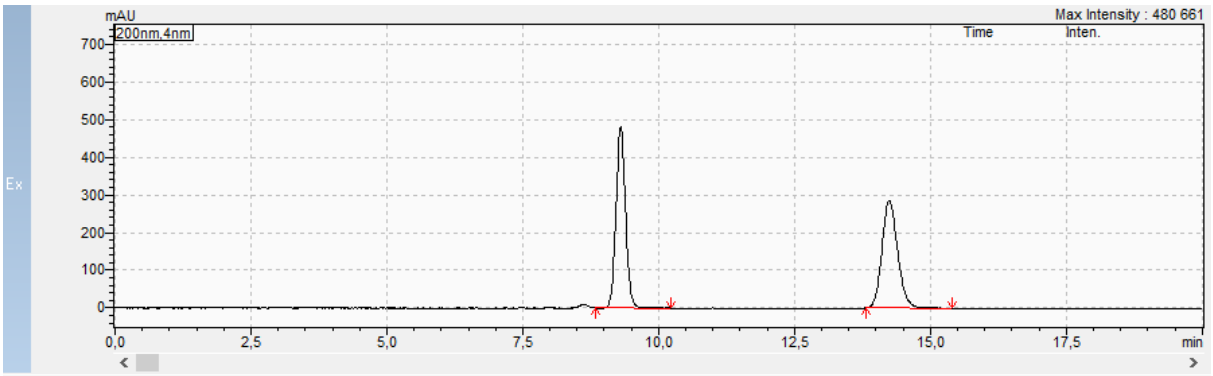

Results View - Peak Table

Peak Table | Compound | Group | Calibration Curve

Peak#	Ret. Time	Area	Height	Mark	Conc.	Unit	ID#	Name	Area%
1	9,301	5844742	481017	M	50,495				50,495
2	14,242	5730224	285783	M	49,505				49,505
Total		11574967	766800		100,000				100,000

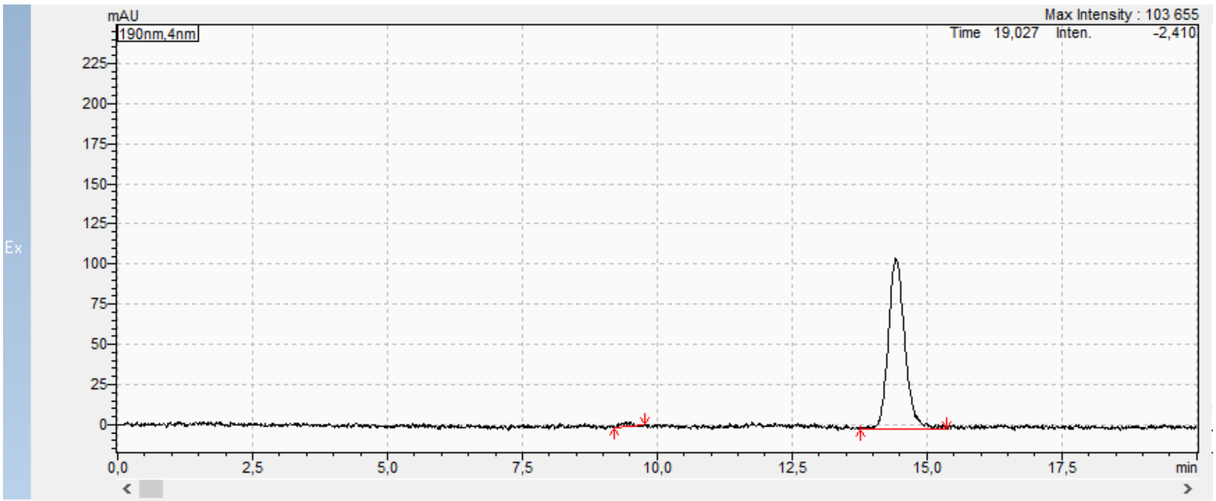

Results View - Peak Table

Peak Table | Compound | Group | Calibration Curve

Peak#	Ret. Time	Area	Height	Peak Start	Peak End	Mark	Conc.	Unit	Area%
1	9,416	32020	2914	9,205	9,781	M	1,402		1,402
2	14,423	2252162	106183	13,760	15,371	M	98,598		98,598
Total		2284183	109096				100,000		100,000

for *ent*-**5a**: *er* = 99:1 (*ee* = 97%)

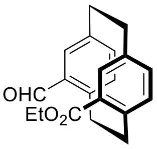

Conditions: IB column
mobile phase: *n*-heptane / *i*-PrOH – 80:20
λ = 196 nm, V = 1.0 ml/min, t = 25 °C
for **5b**: t_R = 7.6 min (major), t_R = 10.9 min (minor)

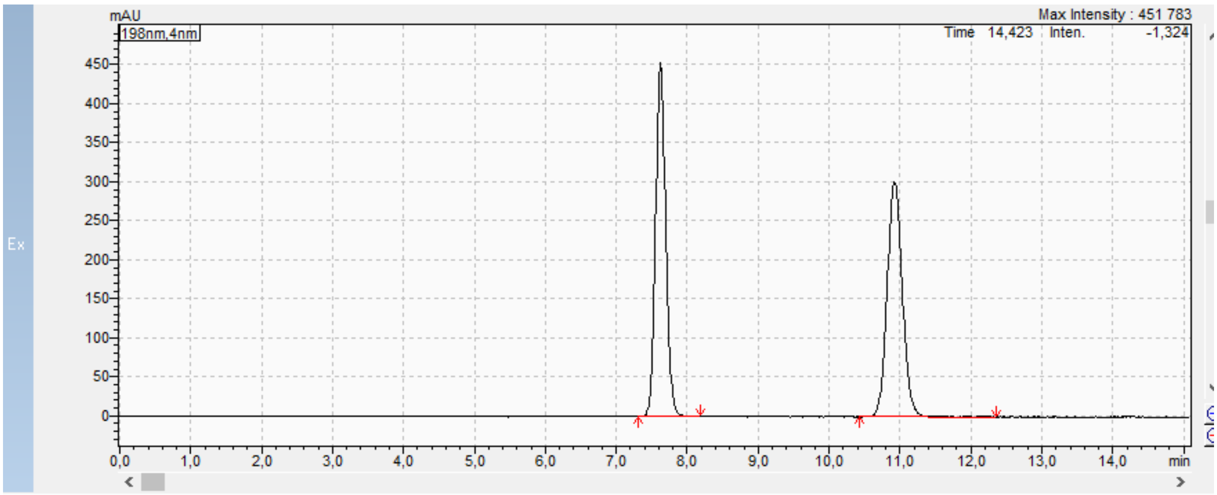

Results View - Peak Table

Peak Table | Compound | Group | Calibration Curve

Peak#	Ret. Time	Area	Height	Peak Start	Peak End	Mark	Conc.	Unit	Area%
1	7,623	4510646	452766	7,307	8,181	M	50,214		50,214
2	10,922	4472265	301490	10,421	12,352	M	49,786		49,786
Total		8982911	754256				100,000		100,000

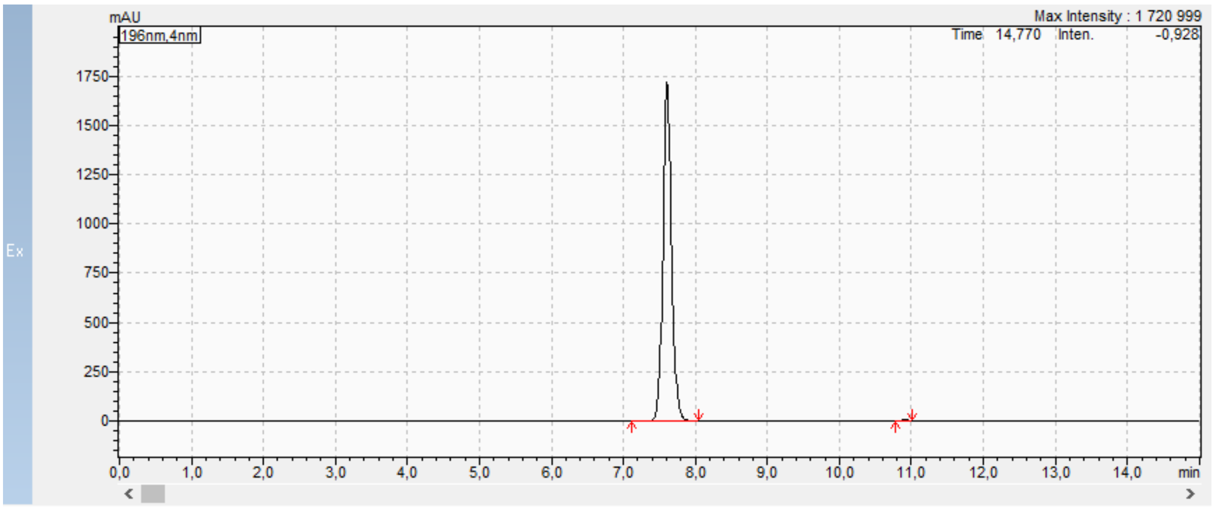

Results View - Peak Table

Peak Table | Compound | Group | Calibration Curve

Peak#	Ret. Time	Area	Height	Peak Start	Peak End	Mark	Conc.	Unit	Area%
1	7,606	14208868	1721625	7,115	8,053	M	99,820		99,820
2	10,911	25591	3205	10,784	11,019	M	0,180		0,180
Total		14234458	1724830				100,000		100,000

for **5b**: *er* = 99.5:0.5 (*ee* = 99%)

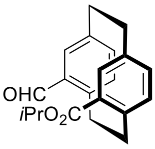

Conditions: IB column
mobile phase: *n*-heptane/*i*-PrOH – 80:20
λ = 215 nm, V = 1.0 ml/min, t = 25 °C
for **5c**: t_R = 6.3 min (major), t_R = 7.8 min (minor)

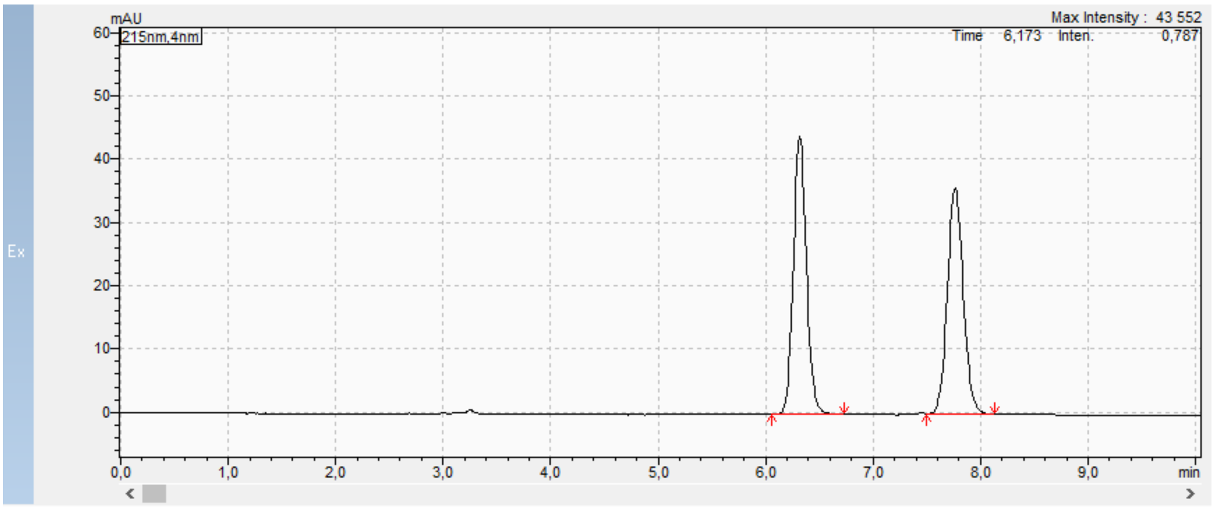

Results View - Peak Table

Peak Table | Compound | Group | Calibration Curve

Peak#	Ret. Time	Area	Height	Peak Start	Peak End	Mark	Conc.	Unit	Area%
1	6,317	372496	43912	6,059	6,731		50,078		50,078
2	7,764	371333	35873	7,499	8,128		49,922		49,922
Total		743828	79785				100,000		100,000

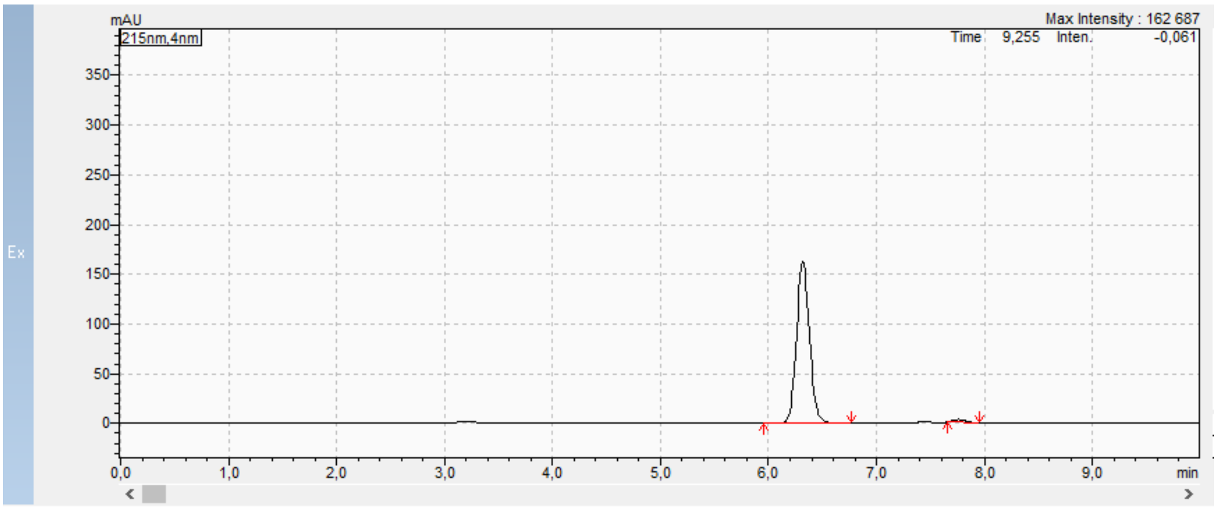

Results View - Peak Table

Peak Table | Compound | Group | Calibration Curve

Peak#	Ret. Time	Area	Height	Peak Start	Peak End	Mark	Conc.	Unit	Area%
1	6,317	1355587	162596	5,952	6,773	M	98,236		98,236
2	7,759	24336	2961	7,659	7,957	M	1,764		1,764
Total		1379923	165557				100,000		100,000

for **5c**: *er* = 98:2 (*ee* = 97%)

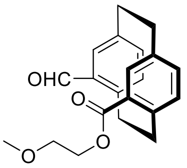

Conditions: IB column
mobile phase: *n*-heptane/*i*-PrOH – 80:20
λ = 215 nm, V = 1.0 ml/min, t = 25 °C
for **5d**: t_R = 17.4 min (major), t_R = 23.4 min (minor)

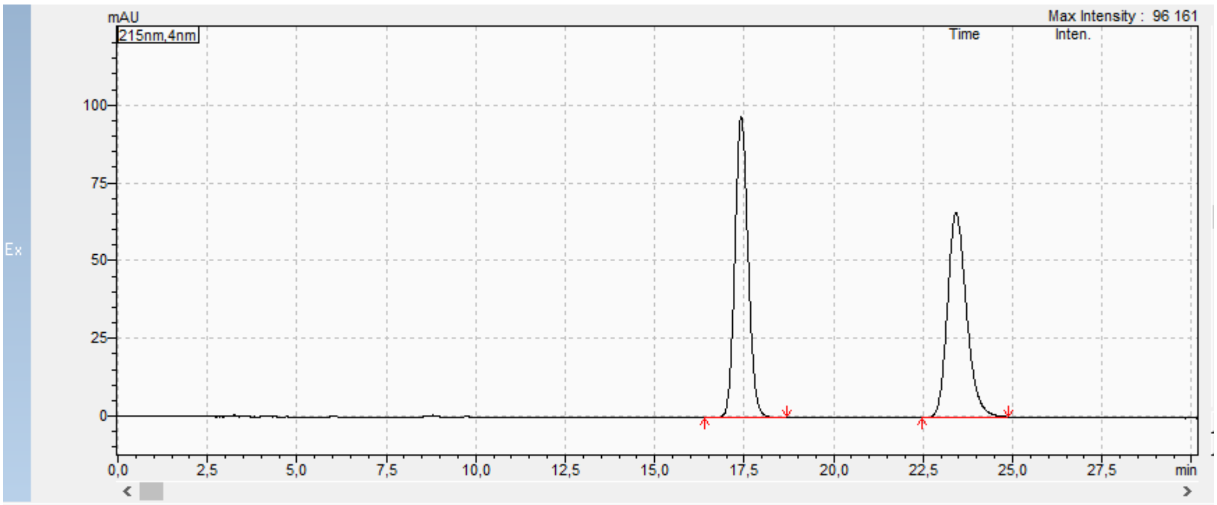

Results View - Peak Table

Peak Table | Compound | Group | Calibration Curve

Peak#	Ret. Time	Area	Height	Peak Start	Peak End	Mark	Conc.	Unit	Area%
1	17,416	2469147	96627	16,405	18,699	M	50,277		50,277
2	23,421	2441926	65882	22,464	24,875	M	49,723		49,723
Total		4911073	162508				100,000		100,000

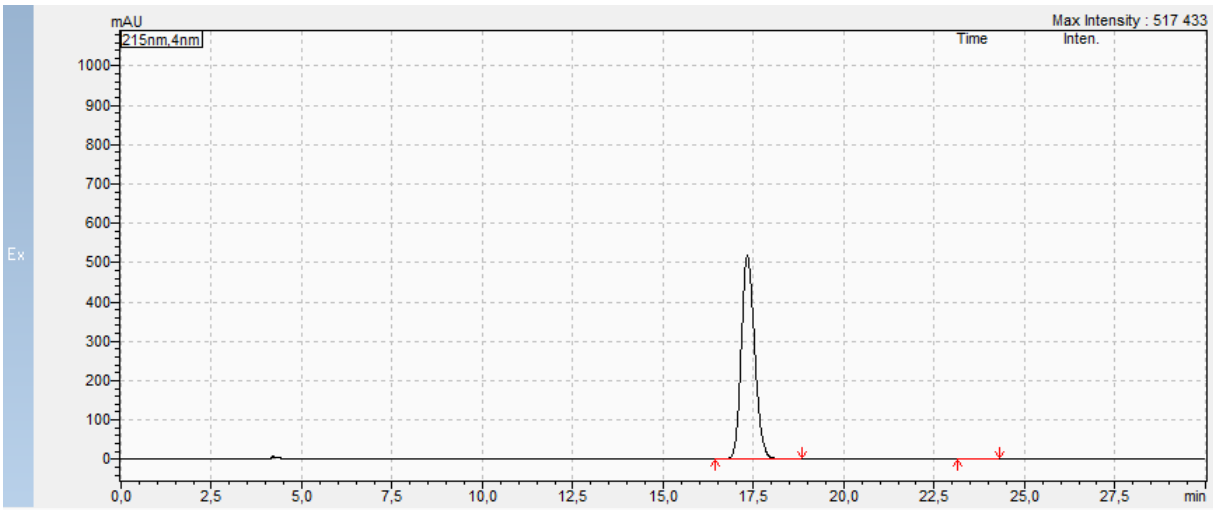

Results View - Peak Table

Peak Table | Compound | Group | Calibration Curve

Peak#	Ret. Time	Area	Height	Peak Start	Peak End	Mark	Conc.	Unit	Area%
1	17,331	13320297	517516	16,427	18,859	M	99,757		99,757
2	23,550	32411	940	23,125	24,299	M	0,243		0,243
Total		13352708	518456				100,000		100,000

for **5d**: *er* = 99.5:0.5 (*ee* = 99%)

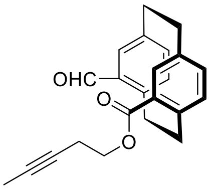

Conditions: IB column
mobile phase: *n*-heptane/*i*-PrOH – 80:20
λ = 201 nm, V = 1.0 ml/min, t = 25 °C
for **5e**: t_R = 9.3 min (major), t_R = 12.4 min (minor)

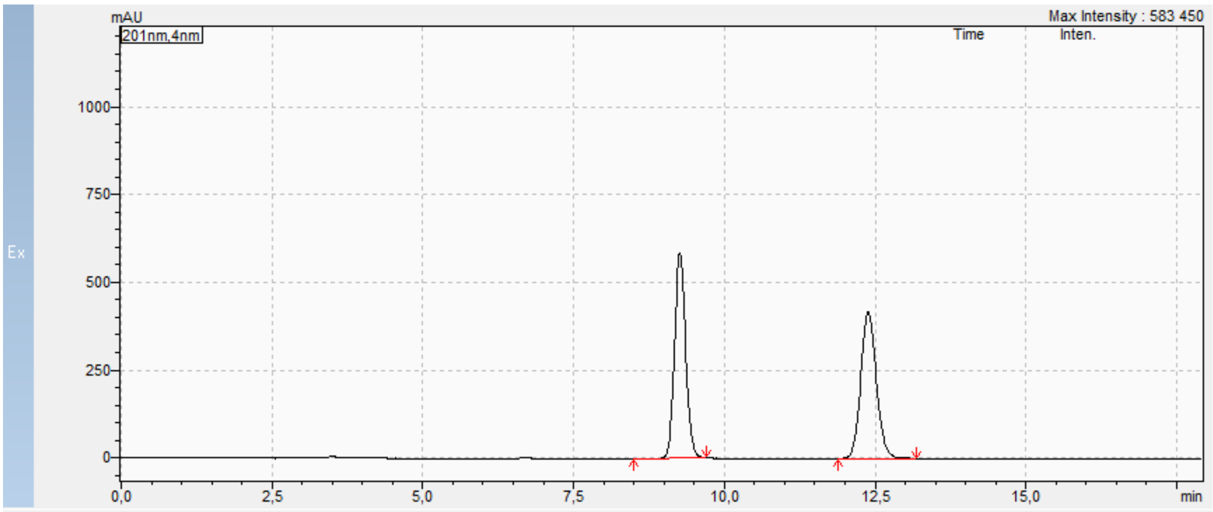

Results View - Peak Table

Peak Table | Compound | Group | Calibration Curve

Peak#	Ret. Time	Area	Height	Peak Start	Peak End	Mark	Conc.	Unit	Area%
1	9,263	7466821	584019	8,501	9,696	M	49,994		49,994
2	12,387	7468648	417652	11,883	13,195	M	50,006		50,006
Total		14935469	1001671				100,000		100,000

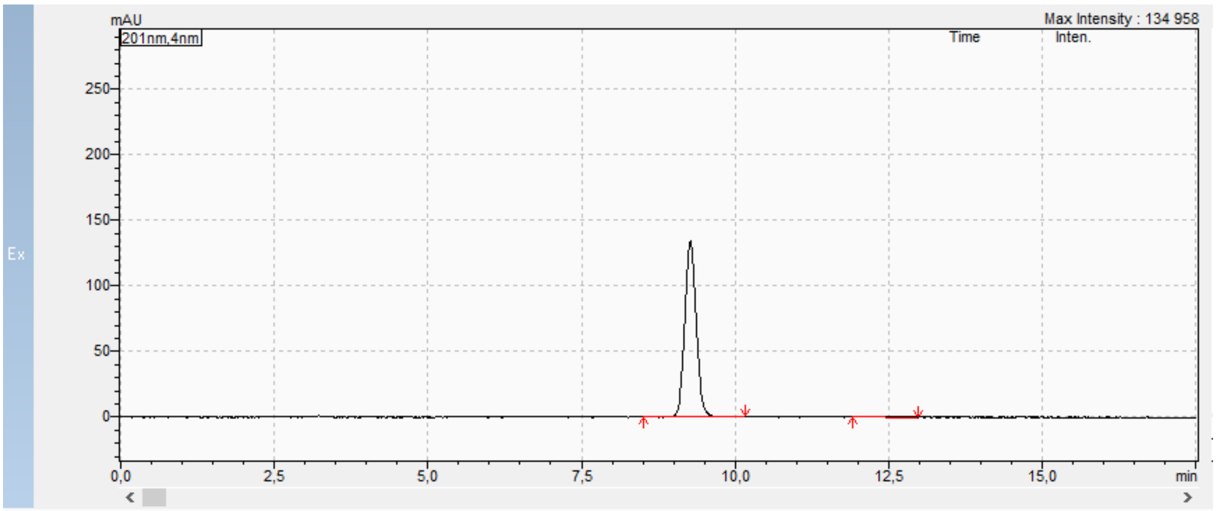

Results View - Peak Table

Peak Table | Compound | Group | Calibration Curve

Peak#	Ret. Time	Area	Height	Peak Start	Peak End	Mark	Conc.	Unit	Area%
1	9,272	1748812	134890	8,501	10,165	M	99,513		99,513
2	12,403	8560	639	11,915	12,971	M	0,487		0,487
Total		1757373	135528				100,000		100,000

for **5e**: *er* = 99.5:0.5 (*ee* = 99%)

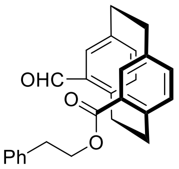

Conditions: IB column
mobile phase: *n*-heptane/*i*-PrOH – 80:20
λ = 213 nm, V = 1.0 ml/min, t = 25 °C
for **5f**: t_R = 10.2 min (major), t_R = 13.0 min (minor)

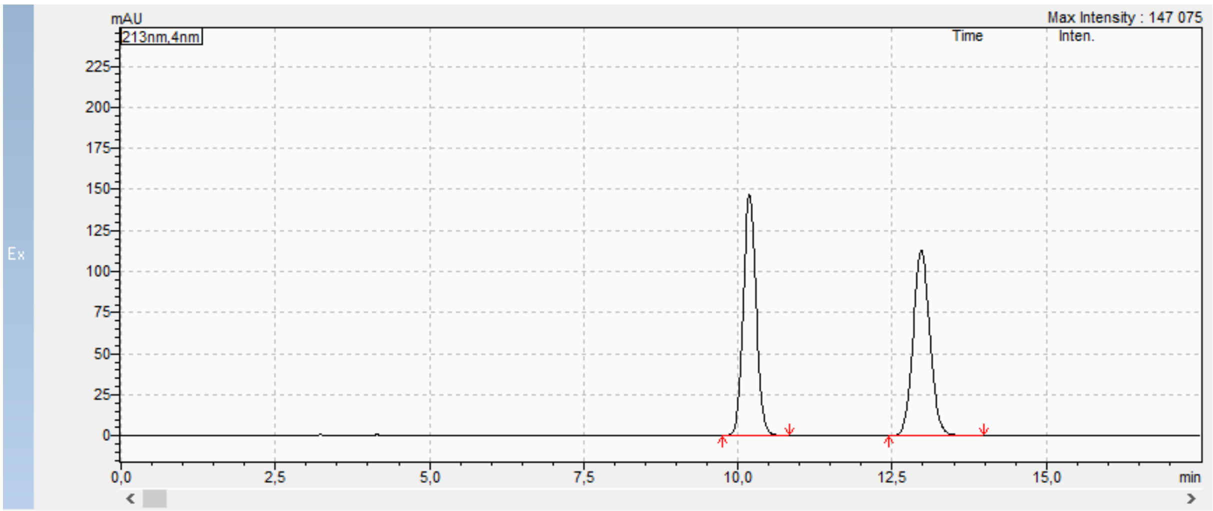

Results View - Peak Table

Peak Table | Compound | Group | Calibration Curve

Peak#	Ret. Time	Area	Height	Peak Start	Peak End	Mark	Conc.	Unit	Area%
1	10,188	2146740	146993	9,749	10,837		50,017		50,017
2	12,974	2145272	112607	12,448	13,995		49,983		49,983
Total		4292012	259599				100,000		100,000

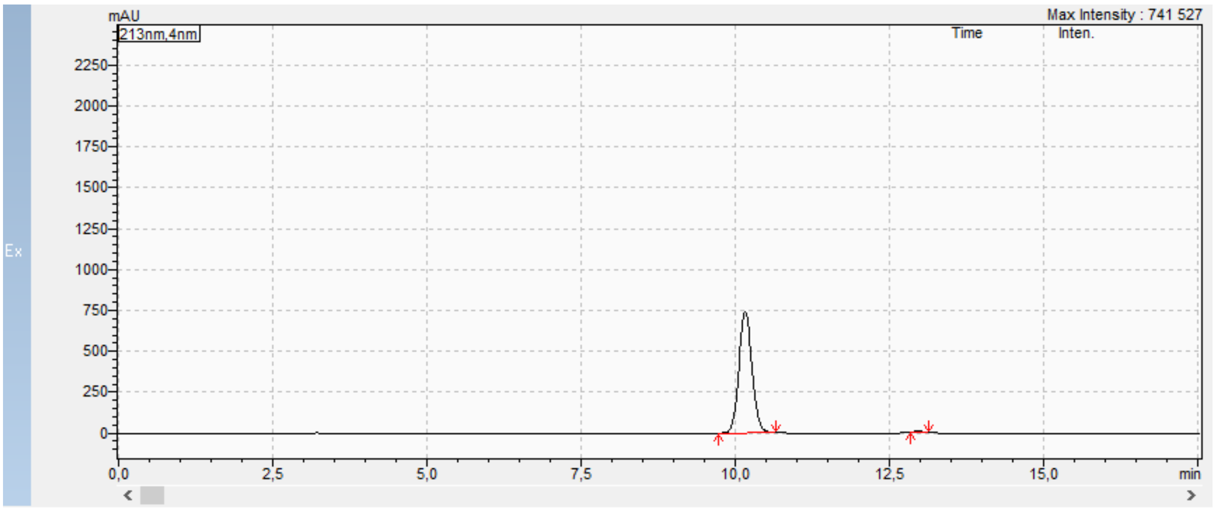

Results View - Peak Table

Peak Table | Compound | Group | Calibration Curve

Peak#	Ret. Time	Area	Height	Peak Start	Peak End	Mark	Conc.	Unit	Area%
1	10,163	10937319	739955	9,739	10,656	M	99,553		99,553
2	12,967	49058	4666	12,853	13,141	M	0,447		0,447
Total		10986377	744621				100,000		100,000

for **5f**: *er* = 99.5:0.5 (*ee* = 99%)

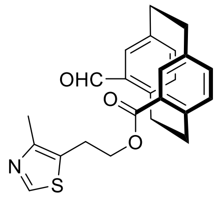

Conditions: IB column
mobile phase: *n*-heptane/*i*-PrOH – 60:40
λ = 252 nm, V = 1.0 ml/min, t = 25 °C
for **5g**: t_R = 10.1 min (major), t_R = 13.1 min (minor)

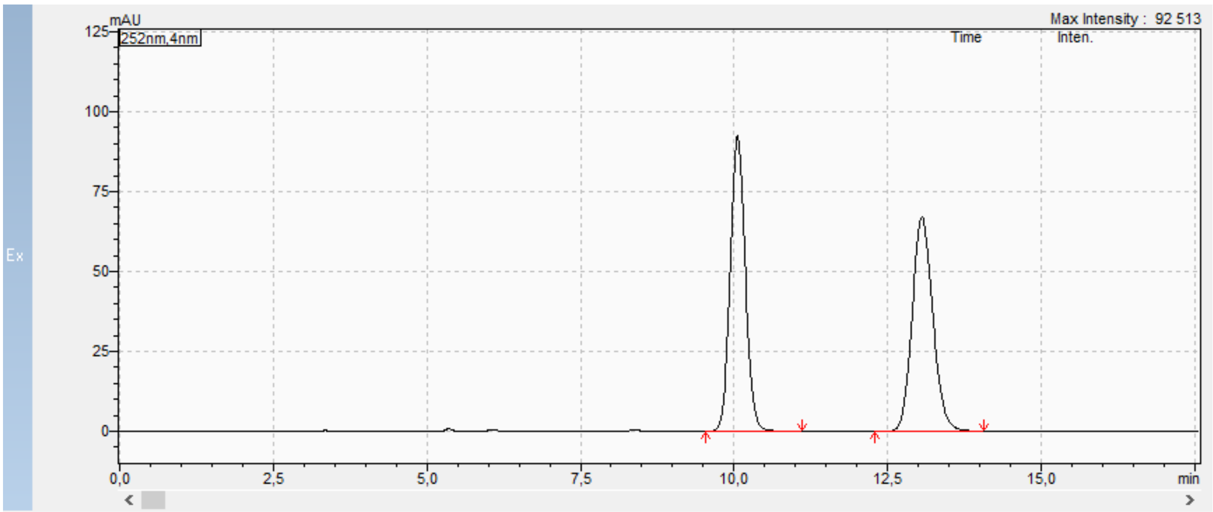

Results View - Peak Table

Peak Table | Compound | Group | Calibration Curve

Peak#	Ret. Time	Area	Height	Peak Start	Peak End	Mark	Conc.	Unit	Area%
1	10,057	1581194	92528	9,536	11,104	M	50,174		50,174
2	13,059	1570213	67088	12,288	14,059	M	49,826		49,826
Total		3151407	159616				100,000		100,000

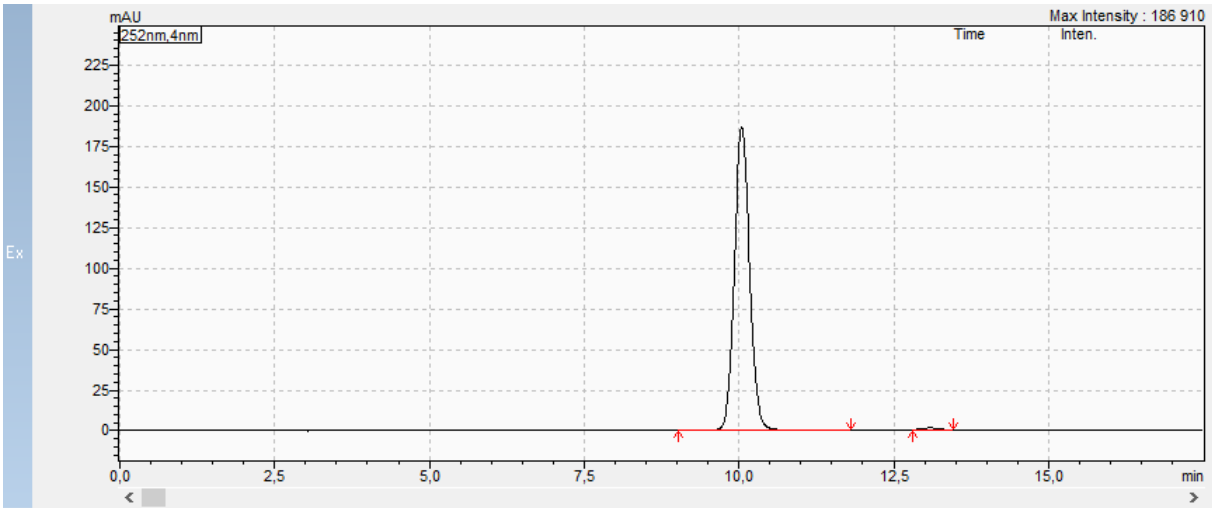

Results View - Peak Table

Peak Table | Compound | Group | Calibration Curve

Peak#	Ret. Time	Area	Height	Peak Start	Peak End	Mark	Conc.	Unit	Area%
1	10,046	3186630	186560	9,024	11,819	M	99,175		99,175
2	13,088	26516	1299	12,800	13,472	M	0,825		0,825
Total		3213146	187859				100,000		100,000

for **5g**: *er* = 99:1 (*ee* = 98%)

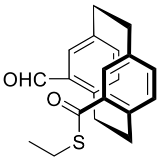

Conditions: IB column
mobile phase: *n*-heptane/*i*-PrOH – 80:20
λ = 211 nm, V = 1.0 ml/min, t = 25 °C
for **5h**: t_R = 8.5 min (major), t_R = 14.3 min (minor)

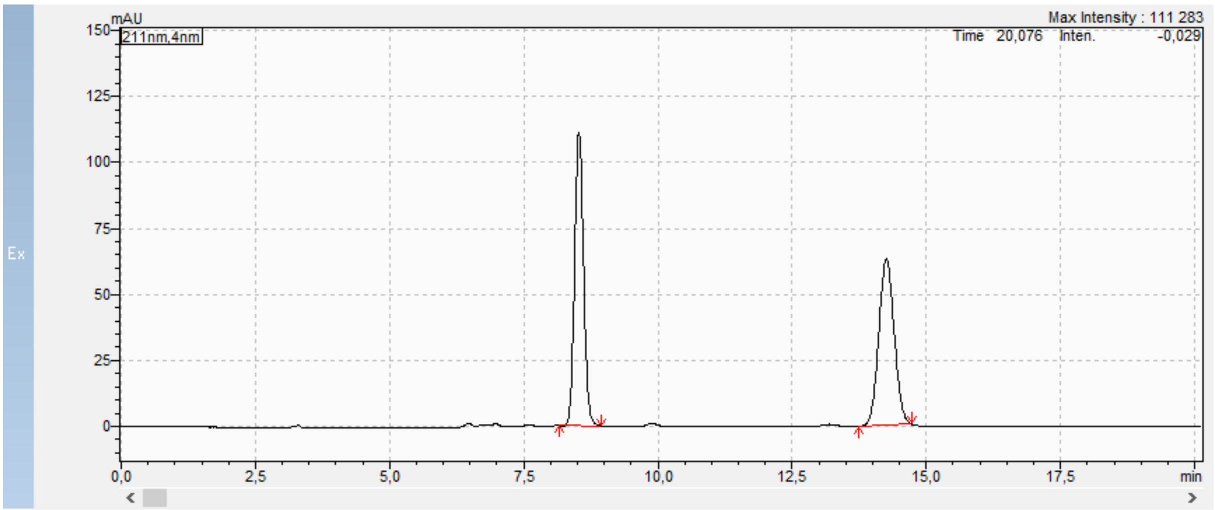

Results View - Peak Table

Peak Table | Compound | Group | Calibration Curve

Peak#	Ret. Time	Area	Height	Peak Start	Peak End	Mark	Conc.	Unit	Area%
1	8,528	1285050	111008	8,160	8,939	M	50,454		50,454
2	14,256	1261942	63065	13,739	14,720	M	49,546		49,546
Total		2546992	174073				100,000		100,000

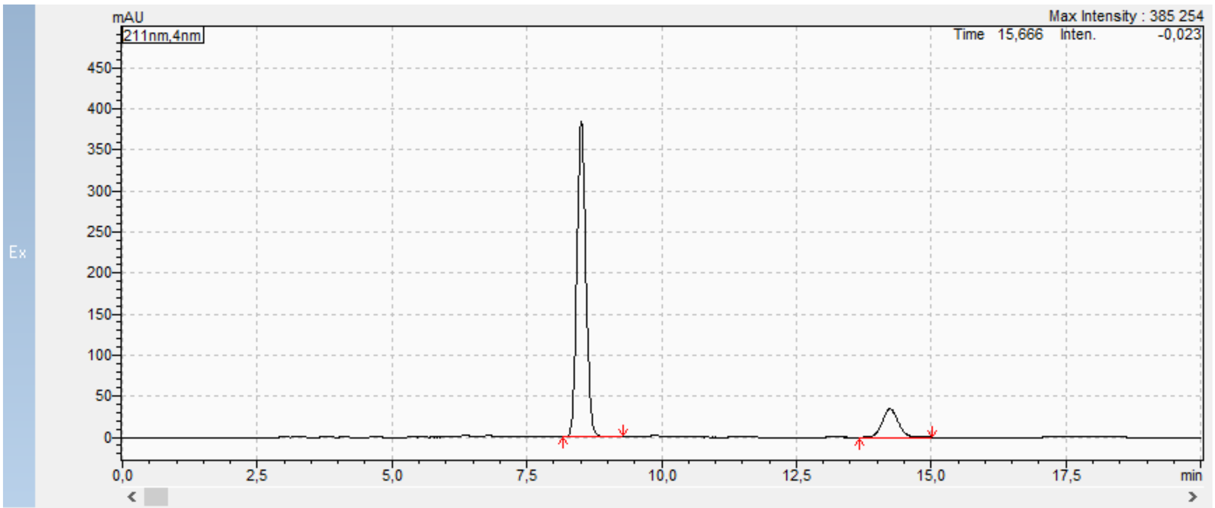

Results View - Peak Table

Peak Table | Compound | Group | Calibration Curve

Peak#	Ret. Time	Area	Height	Peak Start	Peak End	Mark	Conc.	Unit	Area%
1	8,509	4445416	385069	8,181	9,280		86,256		86,256
2	14,238	708329	34536	13,675	15,019	S	13,744		13,744
Total		5153746	419605				100,000		100,000

for **5h**: *er* = 86:14 (*ee* = 73%)

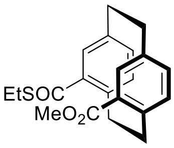

Conditions: IC column
mobile phase: *n*-heptane/*i*-PrOH – 80:20
λ = 212 nm, V = 1.0 ml/min, t = 25 °C
for **7**: t_R = 7.6 min (major), t_R = 16.7 min (minor)

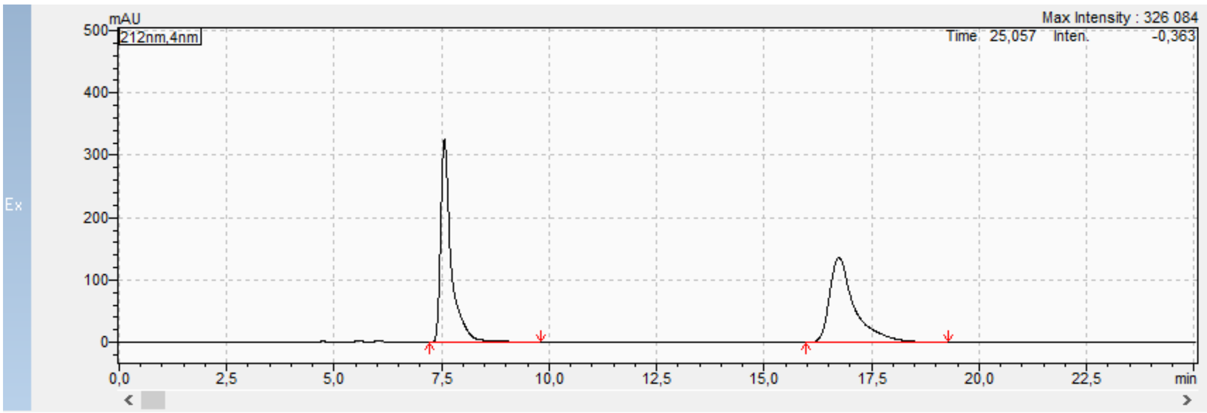

Results View - Peak Table

Peak Table | Compound | Group | Calibration Curve

Peak#	Ret. Time	Area	Height	Peak Start	Peak End	Mark	Conc.	Unit	Area%
1	7,561	5609751	325984	7,221	9,792		50,645		50,645
2	16,739	5466781	135893	15,968	19,285		49,355		49,355
Total		11076532	461877				100,000		100,000

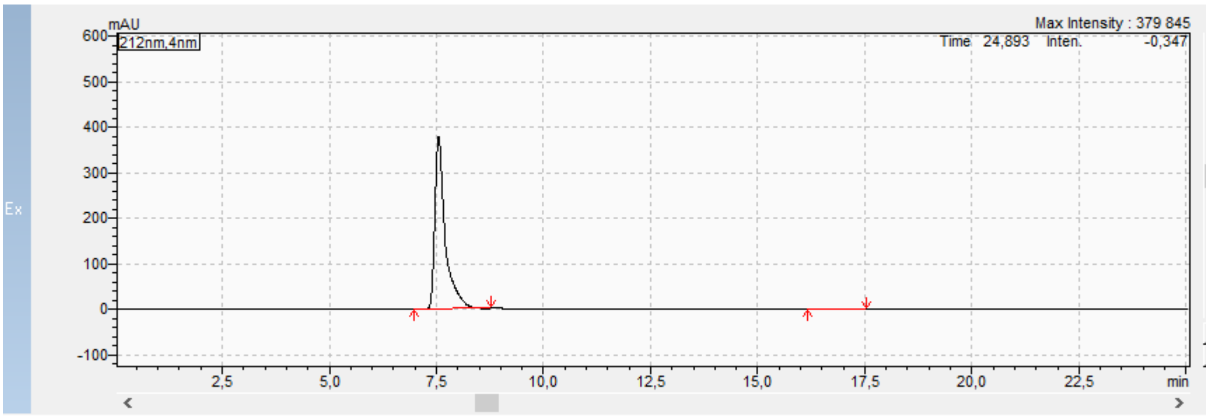

Results View - Peak Table

Peak Table | Compound | Group | Calibration Curve

Peak#	Ret. Time	Area	Height	Peak Start	Peak End	Mark	Conc.	Unit	Area%
1	7,549	6339031	379031	6,976	8,779	M	99,143		99,143
2	16,716	54801	1650	16,171	17,536		0,857		0,857
Total		6393832	380682				100,000		100,000

for **7**: *er* = 99:1 (*ee* = 98%)

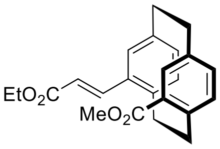

Conditions: IC column
mobile phase: *n*-heptane/*i*-PrOH – 80:20
λ = 262 nm, V = 1.0 ml/min, t = 25 °C
for **8**: t_R = 13.0 min (minor), t_R = 20.6 min (major)

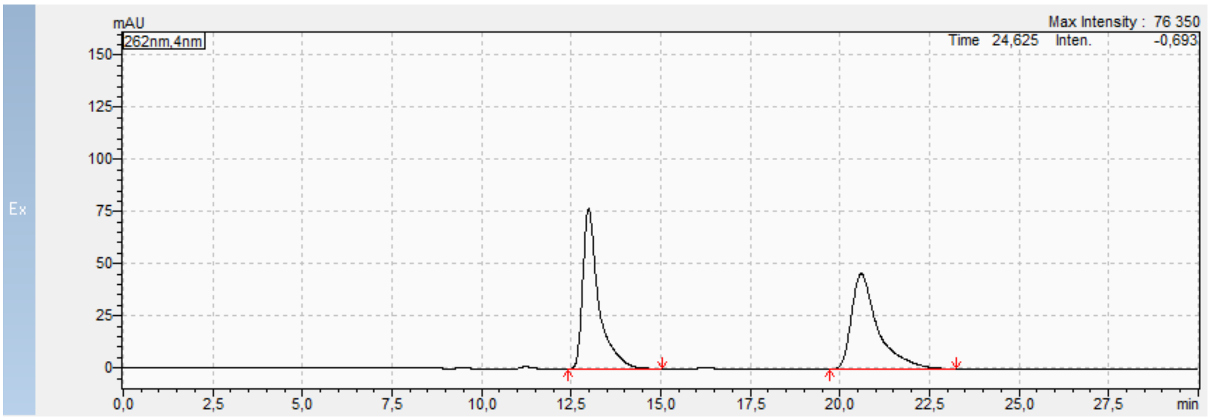

Results View - Peak Table

Peak Table | Compound | Group | Calibration Curve

Peak#	Ret. Time	Area	Height	Peak Start	Peak End	Mark	Conc.	Unit	Area%
1	12,988	2379787	76844	12,427	15,051		50,266		50,266
2	20,595	2354582	45867	19,723	23,232		49,734		49,734
Total		4734369	122711				100,000		100,000

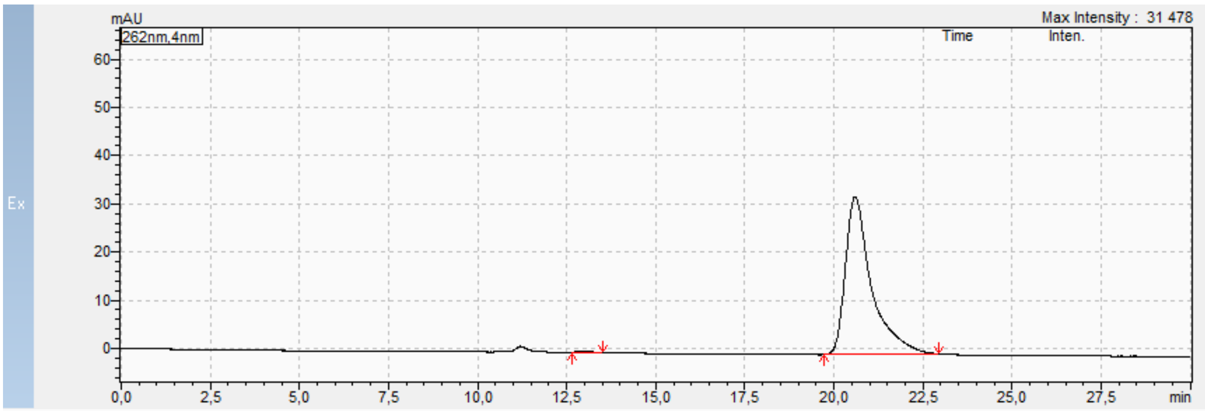

Results View - Peak Table

Peak Table | Compound | Group | Calibration Curve

Peak#	Ret. Time	Area	Height	Peak Start	Peak End	Mark	Conc.	Unit	Area%
1	12,950	8472	358	12,640	13,504	S	0,505		0,505
2	20,596	1668566	32731	19,723	22,955		99,495		99,495
Total		1677038	33090				100,000		100,000

for **8**: *er* = 99.5:0.5 (*ee* = 99%)

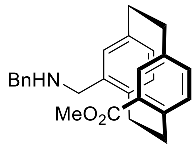

Conditions: IG column
mobile phase: *n*-heptane/*i*-PrOH – 80:20
λ = 209 nm, V = 1.0 ml/min, t = 25 °C
for **9**: t_R = 12.4 min (minor), t_R = 13.5 min (major)

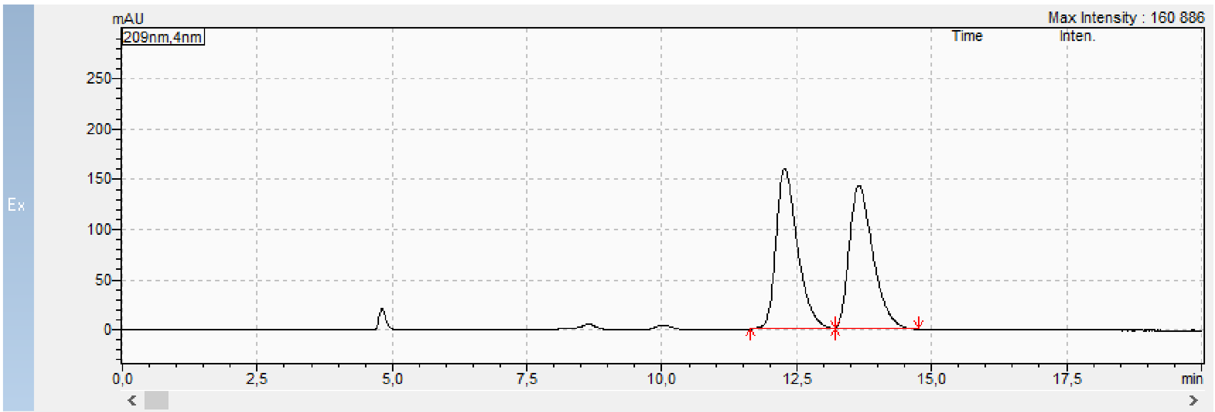

Results View - Peak Table

Peak Table | Compound | Group | Calibration Curve

Peak#	Ret. Time	Area	Height	Peak Start	Peak End	Mark	Conc.	Unit	Area%
1	12,278	4385051	159283	11,637	13,216	M	50,442		50,442
2	13,654	4308218	142357	13,216	14,763	M	49,558		49,558
Total		8693269	301639				100,000		100,000

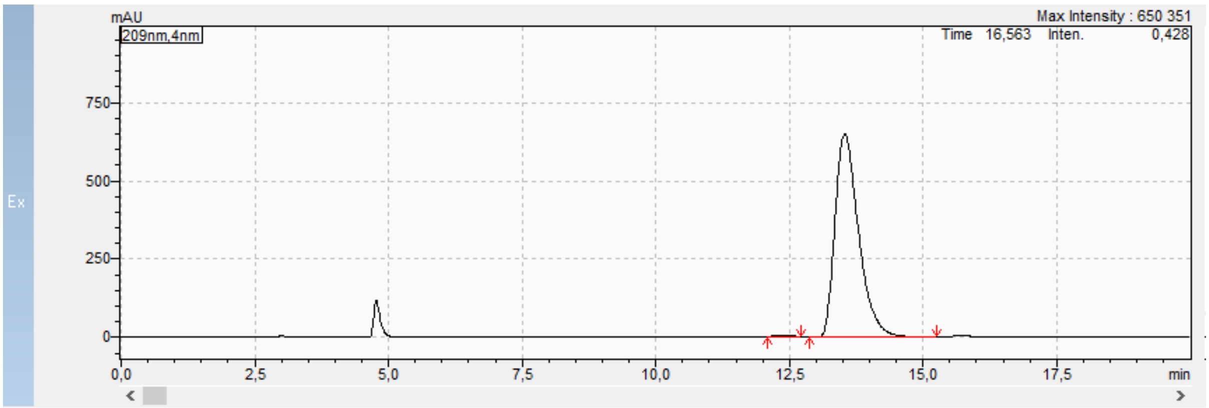

Results View - Peak Table

Peak Table | Compound | Group | Calibration Curve

Peak#	Ret. Time	Area	Height	Peak Start	Peak End	Mark	Conc.	Unit	Area%
1	12,365	122830	5743	12,096	12,725	M	0,609		0,609
2	13,534	20033789	649800	12,885	15,243	M	99,391		99,391
Total		20156619	655542				100,000		100,000

for **9**: *er* = 99:1 (*ee* = 99%)

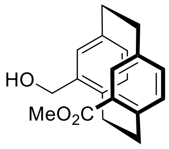

Conditions: IC column
mobile phase: *n*-heptane/*i*-PrOH – 80:20
λ = 232 nm, V = 1.0 ml/min, t = 25 °C
for **10**: t_R = 13.6 min (major), t_R = 16.9 min (minor)

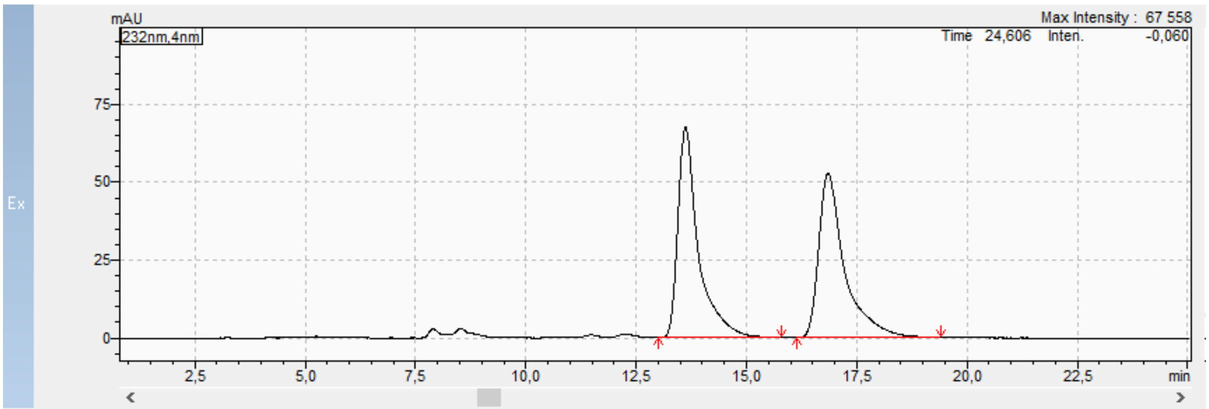

Results View - Peak Table

Peak Table | Compound | Group | Calibration Curve

Peak#	Ret. Time	Area	Height	Peak Start	Peak End	Mark	Conc.	Unit	Area%
1	13,617	2155384	67422	13,013	15,787		50,205		50,205
2	16,854	2137781	52752	16,139	19,392		49,795		49,795
Total		4293165	120175				100,000		100,000

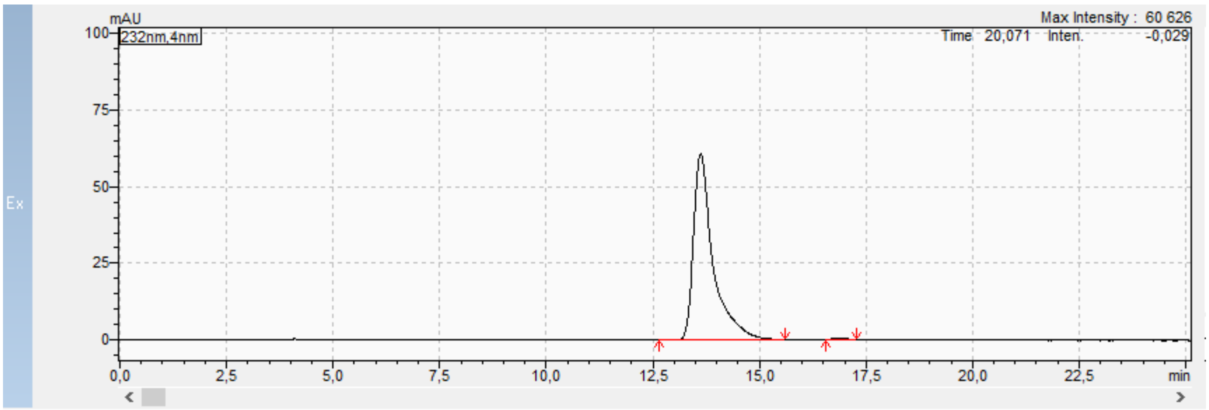

Results View - Peak Table

Peak Table | Compound | Group | Calibration Curve

Peak#	Ret. Time	Area	Height	Peak Start	Peak End	Mark	Conc.	Unit	Area%
1	13,623	1939077	60566	12,661	15,595	M	99,537		99,537
2	16,876	9011	374	16,565	17,280	M	0,463		0,463
Total		1948088	60940				100,000		100,000

for **10**: *er* = 99.5:0.5 (*ee* = 99%)

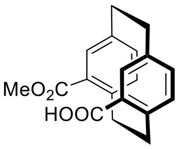

Conditions: IC column
mobile phase: *n*-heptane/*i*-PrOH – 40:60
λ = 238 nm, V = 1.0 ml/min, t = 25 °C
for **11**: t_R = 16.7 min (minor), t_R = 29.0 min (major)

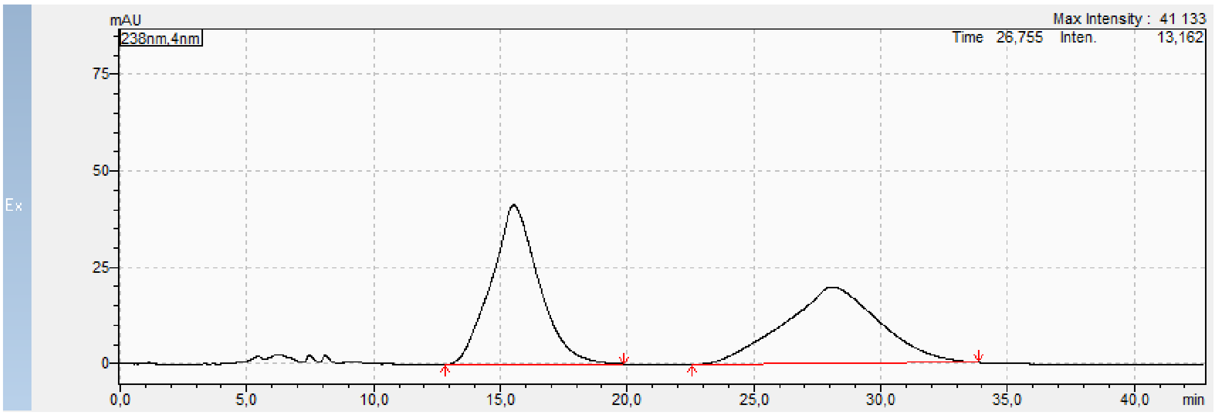

Results View - Peak Table

Peak Table | Compound | Group | Calibration Curve

Peak#	Ret. Time	Area	Height	Peak Start	Peak End	Mark	Conc.	Unit	Area%
1	15,525	5424588	41355	12,800	19,893	S	50,938		50,938
2	28,077	5224847	19983	22,560	33,867		49,062		49,062
Total		10649435	61338				100,000		100,000

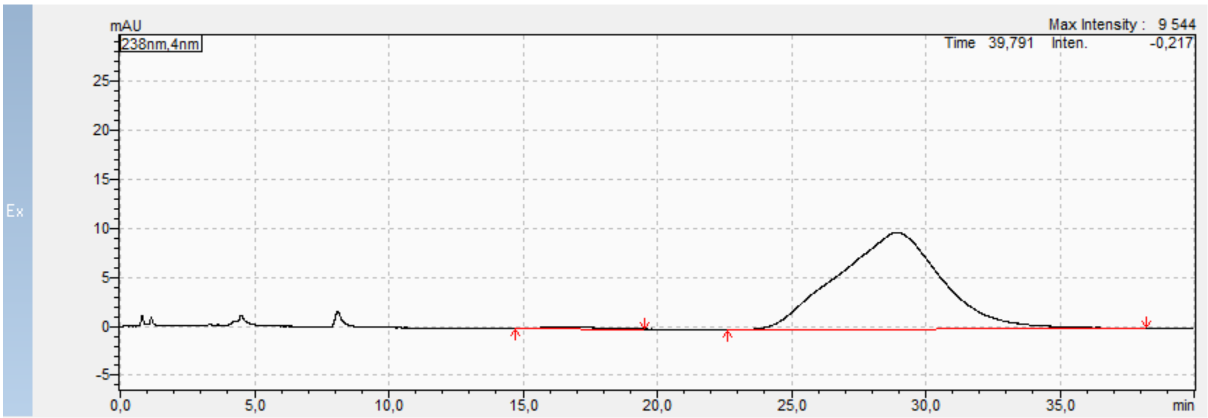

Results View - Peak Table

Peak Table | Compound | Group | Calibration Curve

Peak#	Ret. Time	Area	Height	Peak Start	Peak End	Mark	Conc.	Unit	Area%
1	16,736	25749	198	14,709	19,531	M	0,963		0,963
2	28,947	2647800	9832	22,613	38,219	M	99,037		99,037
Total		2673549	10030				100,000		100,000

for **11**: *er* = 99.1:1 (*ee* = 98%)

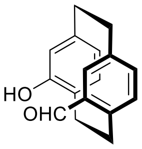

Conditions: IB column
mobile phase: *n*-heptane/*i*-PrOH – 80:20
λ = 257 nm, V = 1.0 ml/min, t = 25 °C
for **12**: t_R = 7.4 min (minor), t_R = 8.4 min (major)

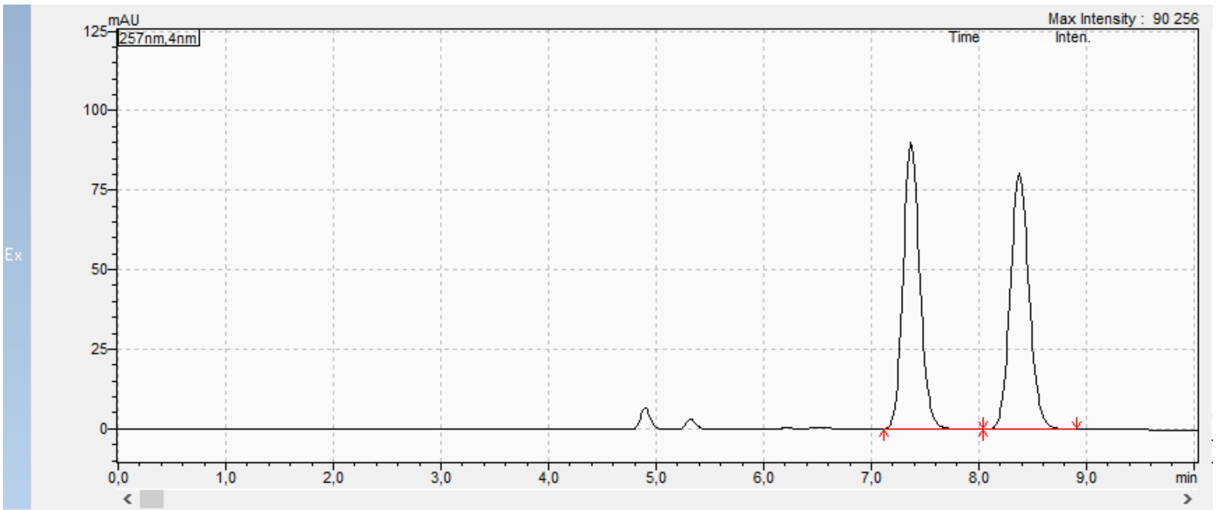

Results View - Peak Table

Peak Table | Compound | Group | Calibration Curve

Peak#	Ret. Time	Area	Height	Peak Start	Peak End	Mark	Conc.	Unit	Area%
1	7,369	977194	90449	7,115	8,043		49,931		49,931
2	8,377	979889	80499	8,043	8,907	V	50,069		50,069
Total		1957083	170948				100,000		100,000

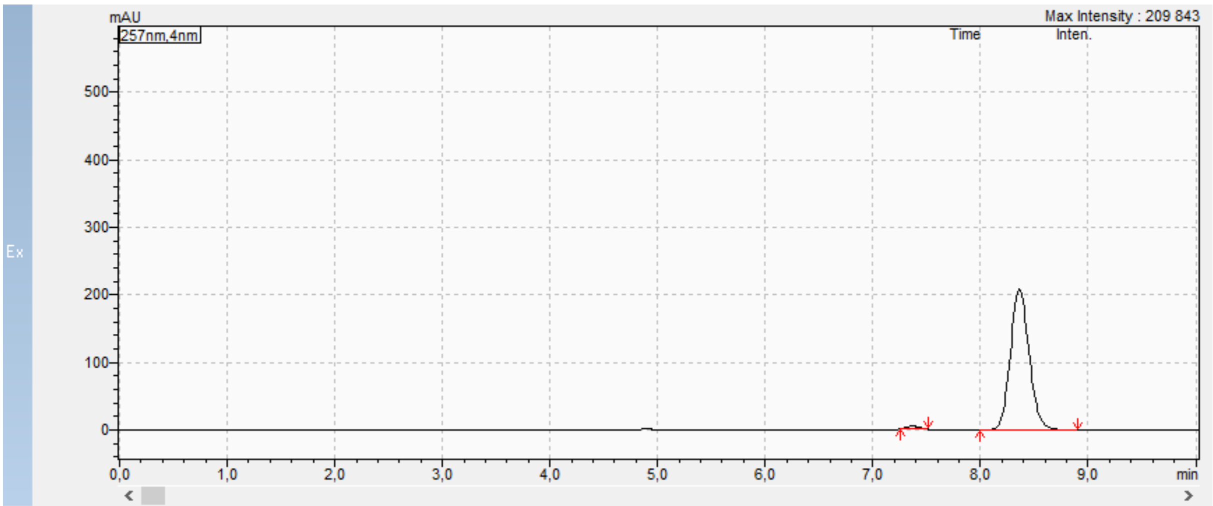

Results View - Peak Table

Peak Table | Compound | Group | Calibration Curve

Peak#	Ret. Time	Area	Height	Peak Start	Peak End	Mark	Conc.	Unit	Area%
1	7,372	35230	4194	7,253	7,509	M	1,377		1,377
2	8,361	2523197	209478	8,000	8,907	M	98,623		98,623
Total		2558427	213672				100,000		100,000

for **12**: *er* = 99:1 (*ee* = 97%)

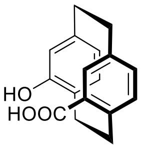

Conditions: IC column
mobile phase: *n*-heptane/*i*-PrOH – 60:40
λ = 208 nm, V = 1.0 ml/min, t = 25 °C
for **13**: t_R = 6.4 min (minor), t_R = 12.7 min (major)

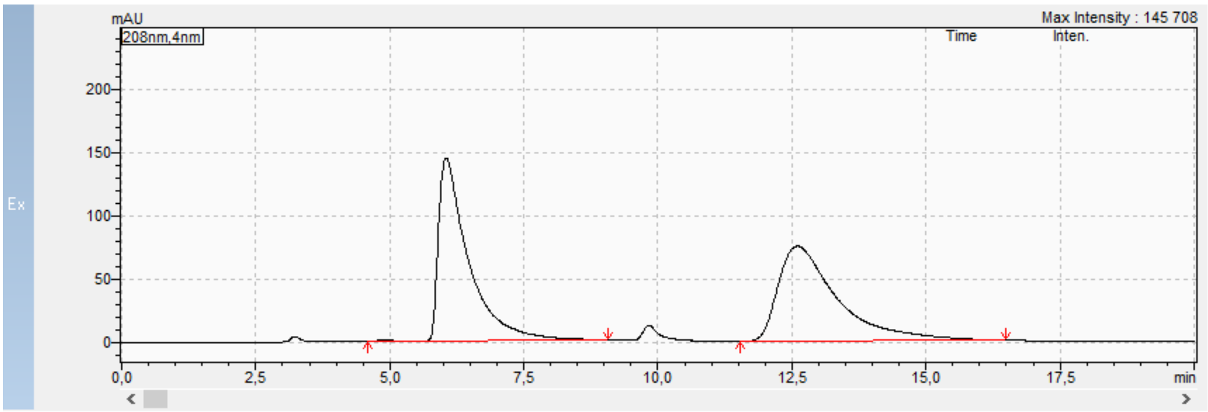

Results View - Peak Table

Peak Table | Compound | Group | Calibration Curve

Peak#	Ret. Time	Area	Height	Peak Start	Peak End	Mark	Conc.	Unit	Area%
1	6,052	5666733	144584	4,597	9,067	M	49,498		49,498
2	12,602	5781623	75188	11,541	16,501		50,502		50,502
Total		11448356	219772				100,000		100,000

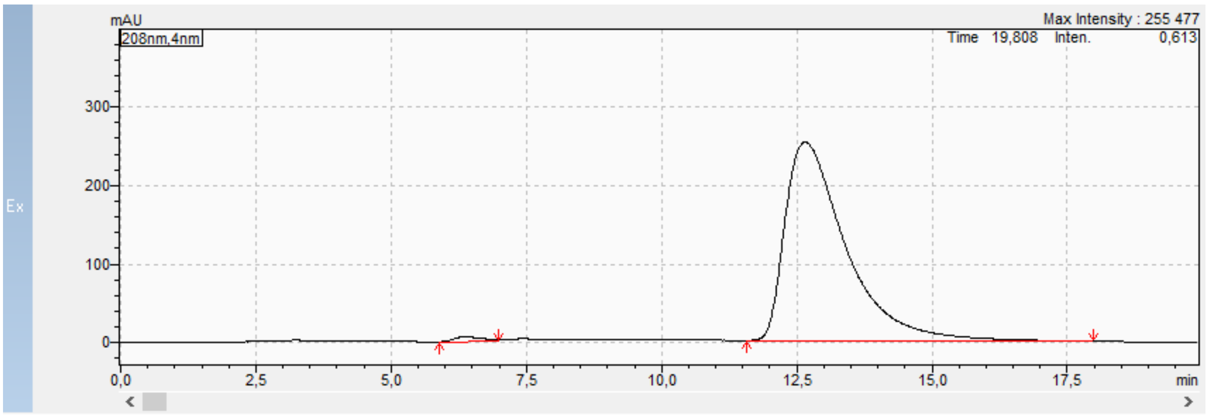

Results View - Peak Table

Peak Table | Compound | Group | Calibration Curve

Peak#	Ret. Time	Area	Height	Peak Start	Peak End	Mark	Conc.	Unit	Area%
1	6,368	220044	5864	5,899	6,997		1,098		1,098
2	12,654	19816739	252838	11,573	17,973		98,902		98,902
Total		20036783	258702				100,000		100,000

for **13**: *er* = 99:1 (*ee* = 98%)

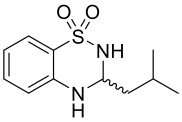

Conditions: ODH column
mobile phase: *n*-heptane/*i*-PrOH – 80:20
λ = 249 nm, V = 1.0 ml/min, t = 25 °C
for **16**: t_R = 7.0 min (minor), t_R = 14.8 min (major)

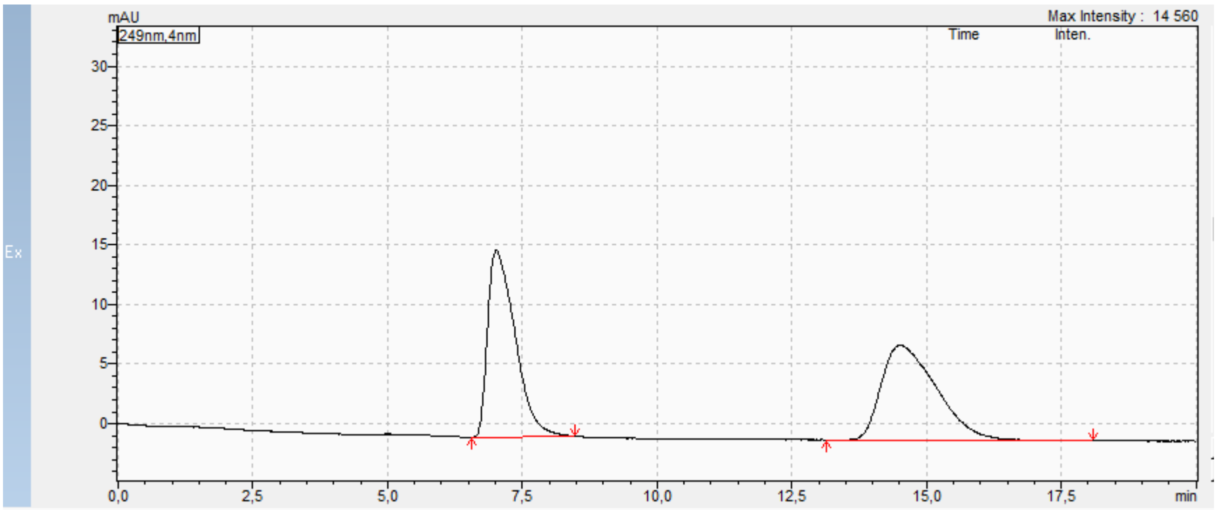

Results View - Peak Table

Peak Table | Compound | Group | Calibration Curve

Peak#	Ret. Time	Area	Height	Peak Start	Peak End	Mark	Conc.	Unit	Area%
1	7,015	554336	15674	6,560	8,469		49,573		49,573
2	14,504	563889	7958	13,141	18,091	M	50,427		50,427
Total		1118225	23633				100,000		100,000

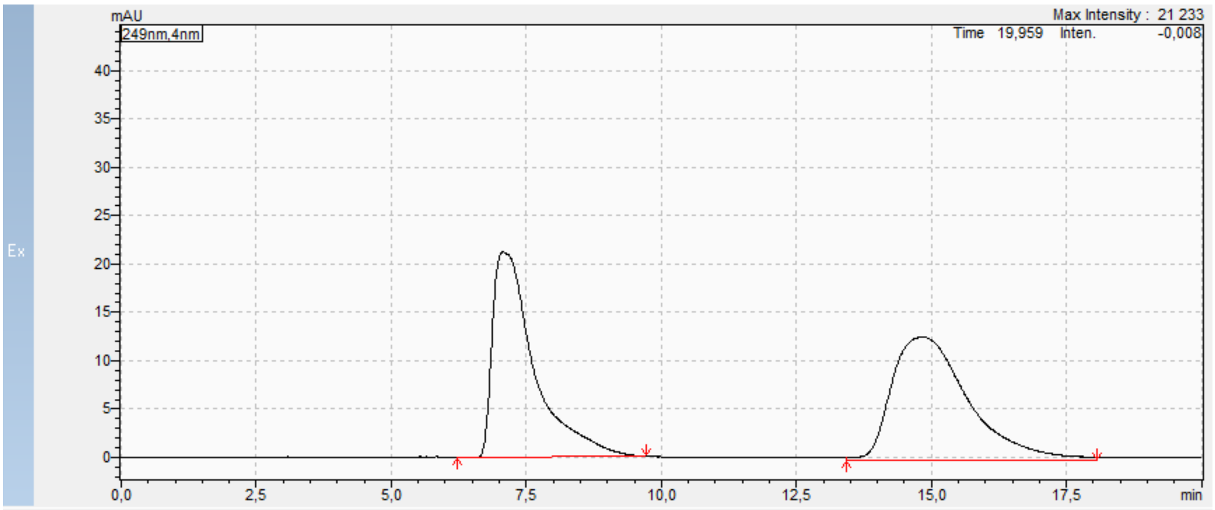

Results View - Peak Table

Peak Table | Compound | Group | Calibration Curve

Peak#	Ret. Time	Area	Height	Peak Start	Peak End	Mark	Conc.	Unit	Area%
1	7,070	1136668	21175	6,208	9,717	M	47,467		47,467
2	14,828	1258002	12735	13,419	18,069	M	52,533		52,533
Total		2394670	33910				100,000		100,000

for **16**: *er* = 53:47 (*ee* = 5%)

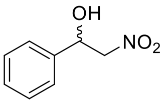

Conditions: ODH column
mobile phase: *n*-heptane/*i*-PrOH – 80:20
λ = 212 nm, V = 1.0 ml/min, t = 25 °C
for **19**: t_R = 10.0 min (major), t_R = 12.0 min (minor)

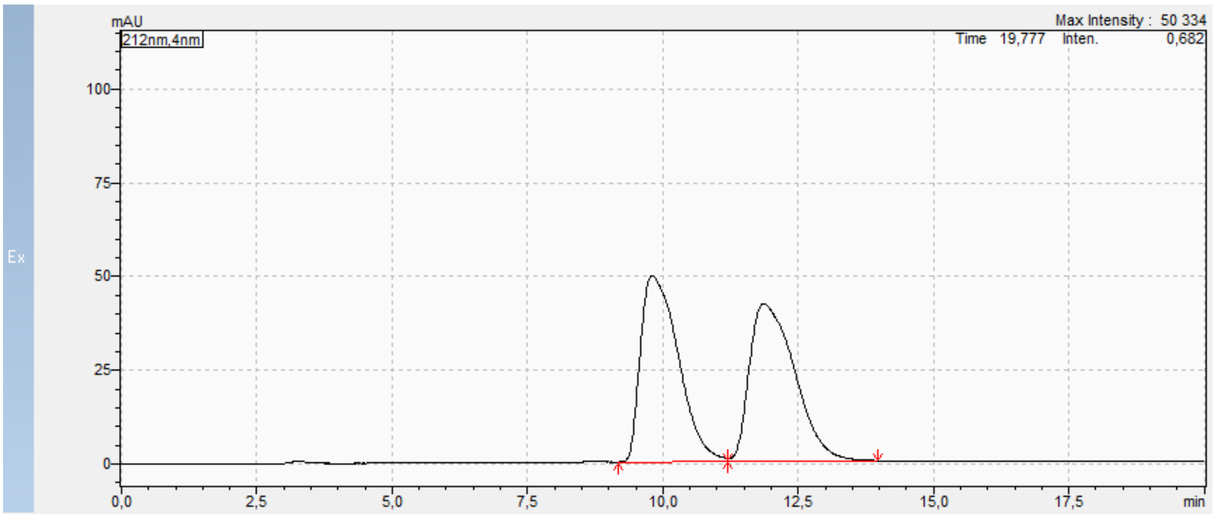

Results View - Peak Table

Peak Table | Compound | Group | Calibration Curve

Peak#	Ret. Time	Area	Height	Peak Start	Peak End	Mark	Conc.	Unit	Area%
1	9,801	2409531	49708	9,184	11,200		49,733		49,733
2	11,860	2435369	42037	11,200	13,973	SV	50,267		50,267
Total		4844900	91746				100,000		100,000

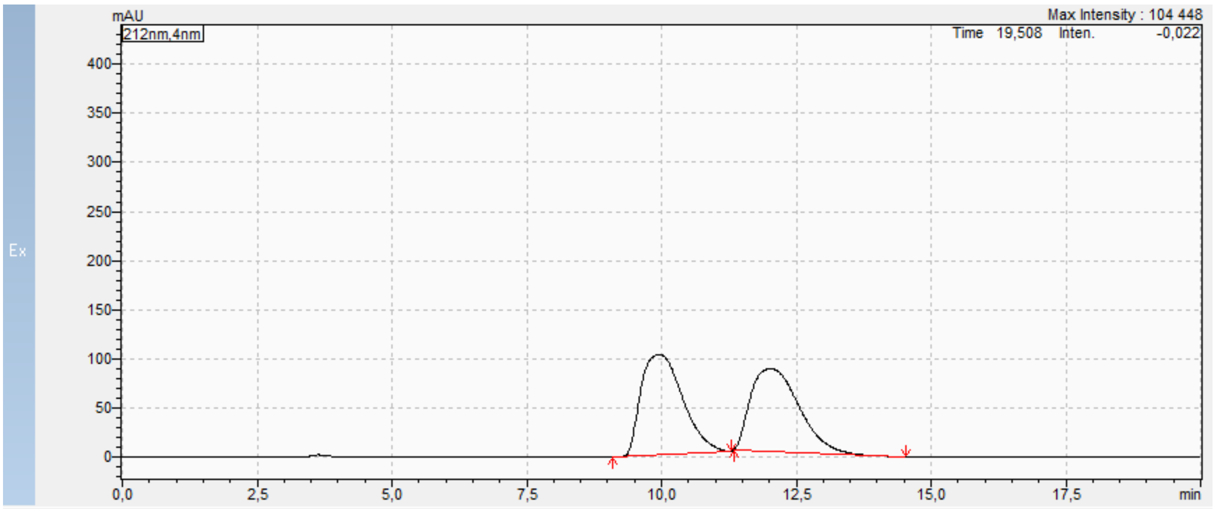

Results View - Peak Table

Peak Table | Compound | Group | Calibration Curve

Peak#	Ret. Time	Area	Height	Peak Start	Peak End	Mark	Conc.	Unit	Area%
1	9,969	5473448	101864	9,088	11,296	M	51,313		51,313
2	12,021	5193325	83642	11,349	14,539	M	48,687		48,687
Total		10666773	185507				100,000		100,000

for **19**: *er* = 51:49 (*ee* = 3%)

References

1. Delcourt, M.-L., Felder, S., Benedetti, E. & Micouin, L. Highly Enantioselective Desymmetrization of Centrosymmetric *pseudo*-*para*-Diformyl[2.2]paracyclophane via Asymmetric Transfer Hydrogenation. *ACS Catal.* **8**, 6612-6616 (2018).
2. Liu, L., Guo, D. & Wang, J. NHC-Catalyzed Asymmetric *α*-Regioselective [4 + 2] Annulation to Construct α-Alkylidene-δ-Lactones. *Org. Lett.* **22**, 7025-7029 (2020).
3. Lewtak, J. P., Landman, M., Fernández, I., & Swarts, J. C. A DFT-Elucidated Comparison of the Solution-Phase and SAM Electrochemical Properties of Short-Chain Mercaptoalkylferrocenes: Synthetic and Spectroscopic Aspects, and the Structure of Fc–CH2CH2–S–S–CH2CH2–Fc. *Inorg. Chem.* **55**, 2584-2596 (2016).
4. Alexander, C., Smith, C. R., Whitcombe, M. J., & Vulfson, E. N. Imprinted Polymers as Protecting Groups for Regioselective Modification of Polyfunctional Substrates. *J. Am. Chem. Soc.* **121**, 6640–6651 (1999).
5. Devlin, A. & Adhikari, A. Small molecule modulators of gut bacterial metabolism. *WO2020231776A1* (2020).
6. Pinilla, I. M., Martínez, M. B. & Galbis, J. A. Synthesis of 2,3,4,5-tetra-*O*-methyl-D-glucono-1,6-lactone as a monomer for the preparation of copolyesters. *Carbohydr. Res.* **338**, 549-555 (2003).
7. Oh, K. An efficient epimerization of biotin sulfone derivatives to 2-*epi*-biotin analogs. *Tetrahedron Lett.* **48**, 3685-3688 (2007).
8. Dahmen, S. & Bräse, S. Preparation of planar chiral amino phenols based on the [2.2]paracyclophane backbone. *Tetrahedron: Asymm.* **12**, 2845-2850 (2001).
9. Rozenberg, V. I. et al. Enantiomerically pure (*R*)- and (*S*)-15-Hydroxy[2.2]paracyclophane-4-carbaldehyde (*iso*-FHPC): A Novel Parent Compound for Planar Chiral Ligands. *Eur. J. Org. Chem.* **2003**, 2056-2061 (2003).
10. Sui, Y. et al. Highly Enantioselective Synthesis of Cyclic Aminals with a Cyclopentadiene-Based Chiral Carboxylic Acid. *European J. Org. Chem.* **2018**, 215-218 (2018).
11. Kitagaki, S., Ueda, T. & Mukai, C. Planar chiral [2.2]paracyclophane-based bis(thiourea) catalyst: application to asymmetric Henry reaction. *Chem. Commun.* **49**, 4030–4032 (2013).
12. Wessels, A., Klussmann, M., Breugst, M., Schlörer, E. N. & Berkessel, A. Formation of Breslow Intermediates from *N*-Heterocyclic Carbenes and Aldehydes Involves Autocatalysis by the Breslow Intermediate, and a Hemiacetal. *Angew. Chem. Int. Ed.* **61**, e202117682 (2022).
13. Moore, J. L., Silvestri, A. P., de Alaniz, J. R., DiRocco, D. A. & Rovis, T. Mechanistic Investigation of the Enantioselective Intramolecular Stetter Reaction: Proton Transfer Is the First Irreversible Step. *Org. Lett.* **13**, 1742-1745 (2011)
14. Collett, C. J., Young, C. M., Massey, R. S., O'Donoghue, A. C. & Smith, A. D. Kinetic and Structure-Activity Studies of the Triazolium Ion- Catalyzed Intramolecular Stetter Reaction. *European J. Org. Chem.* **2021**, 3670-3675 (2021).
15. Edwards, D. R., Montoya-Peleaz, P., & Crudden, C. M. Experimental Investigation into the Mechanism of the Epoxidation of Aldehydes with Sulfur Ylides. *Org. Lett.* **9**, 5481-5484 (2007).
16. Sheldrick, G. M. SHELXT-Integrated Space-Group and Crystal-Structure Determination. *Acta Crystallogr. Sect. A Found. Crystallogr.* **A71**, 3-8 (2015).
17. Sheldrick, G. M. Crystal structure refinement with SHELXL. *Acta Crystallogr. Sect. C Struct. Chem.* **C71**, 3-8 (2015).
18. Parsons, S., Flack, H. D. & Wagner, T. Use of intensity quotients and differences in absolute structure refinement. *Acta Crystallogr. Sect. B Struct. Sci. Cryst. Eng. Mater.* **B69**, 249-259 (2013).
